# Chemodivergence in Pd-catalyzed desymmetrization of allenes: enantioselective [4+3] cycloaddition, desymmetric allenylic substitution and enynylation†

**DOI:** 10.1039/d3sc04581a

**Published:** 2023-09-21

**Authors:** Pengfei Luo, Long Li, Xinfang Mao, Zheng Sun, Yingcheng Wang, Fangzhi Peng, Zhihui Shao

**Affiliations:** a Key Laboratory of Medicinal Chemistry for Natural Resource, Ministry of Education, School of Chemical Science and Technology, Yunnan Provincial Center for Research & Development of Natural Products, State Key Laboratory for Conservation and Utilization of Bio-Resources in Yunnan, Yunnan University Kunming 650091 China zhihui_shao@hotmail.com

## Abstract

A class of prochiral allenylic di-electrophiles have been introduced for the first time as three-atom synthons in cycloadditions, and a new type of [4+3] cycloaddition involving transition metal-catalyzed enantioselective sequential allenylic substitution has been successfully developed, enabling challenging seven-membered exocyclic axially chiral allenes to be accessed in good yields with good enantioselectivity. Through the addition of a catalytic amount of *ortho*-aminoanilines or *ortho*-aminophenols, the racemization of the [4+3] cycloaddition products is effectively suppressed. Mechanistic studies reveal that elusive Pd-catalyzed enantioselective intramolecular allenylic substitution rather than intermolecular allenylic substitution is the enantio-determining step in this cycloaddition. By tuning the ligands, a Pd-catalyzed enantioselective desymmetric allenylic substitution leading to linear axially chiral tri-substituted allenes or a Pd-catalyzed tandem desymmetric allenylic substitution/β-vinylic hydrogen elimination (formal enynylation) leading to multi-functionalized 1,3-enynes is achieved chemodivergently.

## Abstract 2

Pd-catalyzed enantioselective allenylic [4+3] cycloaddition, desymmetric allenylic substitution or tandem desymmetric allenylic substitution/β-vinylic hydrogen elimination (formal enynylation) can be realized chemodivergently by ligand control.

## Graphical abstract

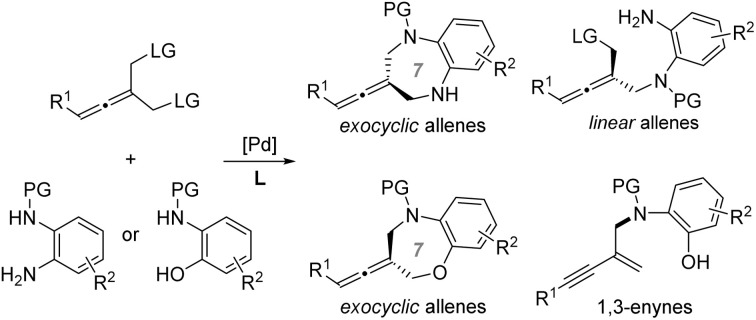

## Introduction

The development of efficient methods to construct medium-sized ring systems is of great interest. In particular, seven-membered ring systems are ubiquitous in natural products, bioactive molecules, and pharmaceuticals.^1^ However, these ring systems are more challenging to construct, due to unfavorable entropic effects and transannular interactions.^2^ The intermolecular cycloaddition reaction is one of the most straightforward and powerful methods for the construction of structurally diverse ring systems.^3^ However, in contrast with well-developed [3+2] and [3+3] cycloadditions, [4+3] cycloadditions especially in a catalytic asymmetric manner, are under-developed.^4,5^ Thus, developing new three-atom or four-atom synthons and designing new strategies for catalytic asymmetric [4+3] cycloaddition to construct seven-membered cyclic compounds, especially those which are difficult to access by existing methods, are highly desirable. In contrast with the previously reported studies focusing on the construction of seven-membered ring systems bearing *central chirality*, catalytic asymmetric [4+3] cycloaddition to construct seven-membered ring systems bearing *axial chirality* remains elusive.

**Fig. 1:**
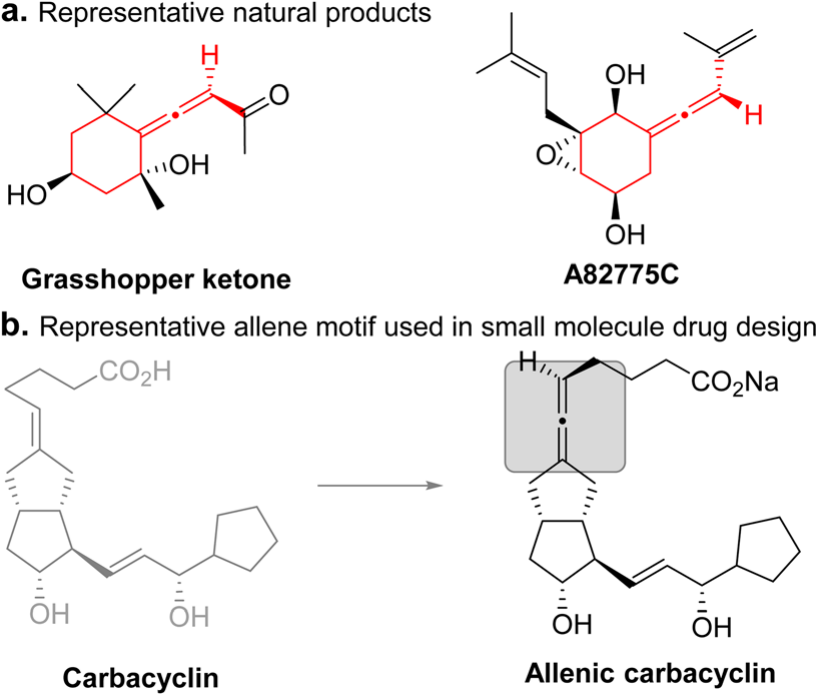
Representative examples of chiral exocyclic allenes in natural products and the allene motif used in small molecule drug design.

Axially chiral allenes are ubiquitous in natural products, bioactive molecules, and functional materials, and also serve as versatile chiral building blocks in organic synthesis due to their unique structure and diverse reactivities.^6^ Thus, developing general methods for the efficient catalytic enantioselective synthesis of axially chiral allene-containing compounds has become an active area of research in organic chemistry.^7^ However, studies on the synthesis of exocyclic allenes largely lag behind those on linear allenes. As a major subclass, exocyclic allenes are present in many natural products and pharmaceuticals (Fig. 1a).^8^ By introducing an allene moiety into the existing exocyclic backbone of the molecule, the biological and pharmacological properties could be tuned (Fig. 1b).^9^ To date, only a few methods have been reported to construct exocyclic axially chiral allenes. However, these methods are largely limited to the construction of five- or six-membered rings.^10^ Catalytic enantioselective construction of seven-membered exocyclic axially chiral allenes is highly challenging. To our knowledge, general methods for the catalytic enantioselective construction of seven-membered exocyclic axially chiral allenes in good yields and enantioselectivity remain elusive. Therefore, their potential applications have been largely unexplored. Therefore, the development of an efficient and general method to construct such synthetically valuable compounds from simple starting materials in a single step is highly desirable.

**Fig. 2:**
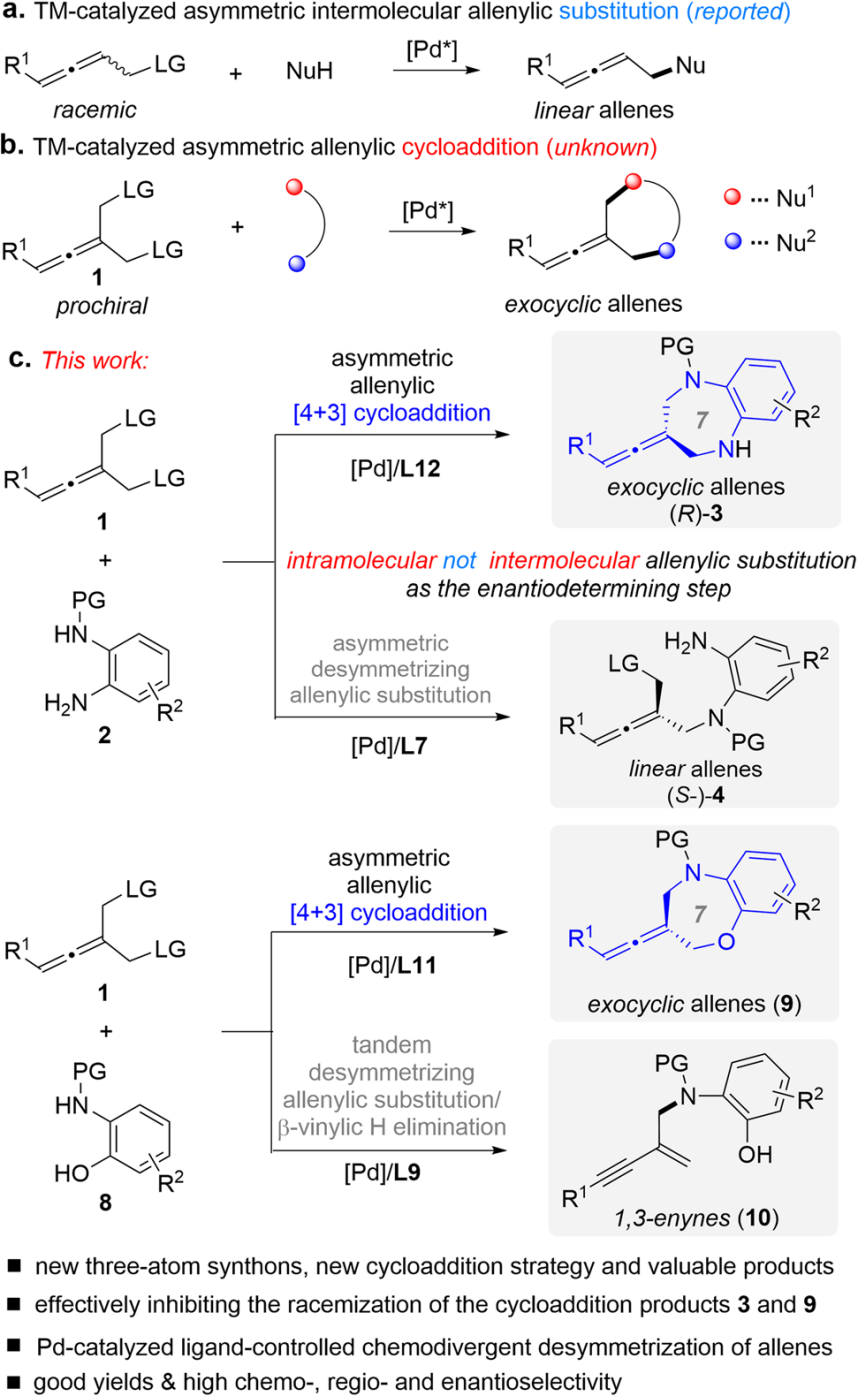
Design of a new type of TM-catalyzed asymmetric [4+3] cycloaddition and chemodivergent desymmetric reaction of prochiral allenes 1. LG = leaving group.

In line with our interest in cycloaddition chemistry^11^ and allene chemistry,^12*f*,*g*^ herein we introduce an intriguing class of substrates, prochiral allenes 1, for the first time as three-atom synthons in cycloadditions, design a type of cycloaddition strategy (Fig. 2b), and demonstrate their utility in the context of Pd-catalyzed asymmetric desymmetric [4+3] cycloaddition (Fig. 2c).^13^ This cycloaddition reaction is a new sequence process that involves intermolecular/intramolecular allenylic substitution reactions, in which the enantio-determining step was found to be the elusive intramolecular allenylic substitution rather than intermolecular allenylic substitution. This represents an important addition to the armory of [4+3] cycloadditions, and also enables general access to difficult-to-access seven membered exocyclic axially chiral allenes in good yields with good enantioselectivity. Using this protocol, we have produced a range of axially chiral allene-containing 1,5-benzodiazepines and 1,5-benzoxazepines. Both 1,5-benzodiazepines and 1,5-benzoxazepines are important scaffolds in medicinal chemistry and organic chemistry which exist ubiquitously in biologically active molecules and pharmaceuticals.^14^ Thus, it would be of high interest to combine an allene and 1,5-benzodiazepine or 1,5-benzoxazepine into one molecule.

**Fig. 3:**
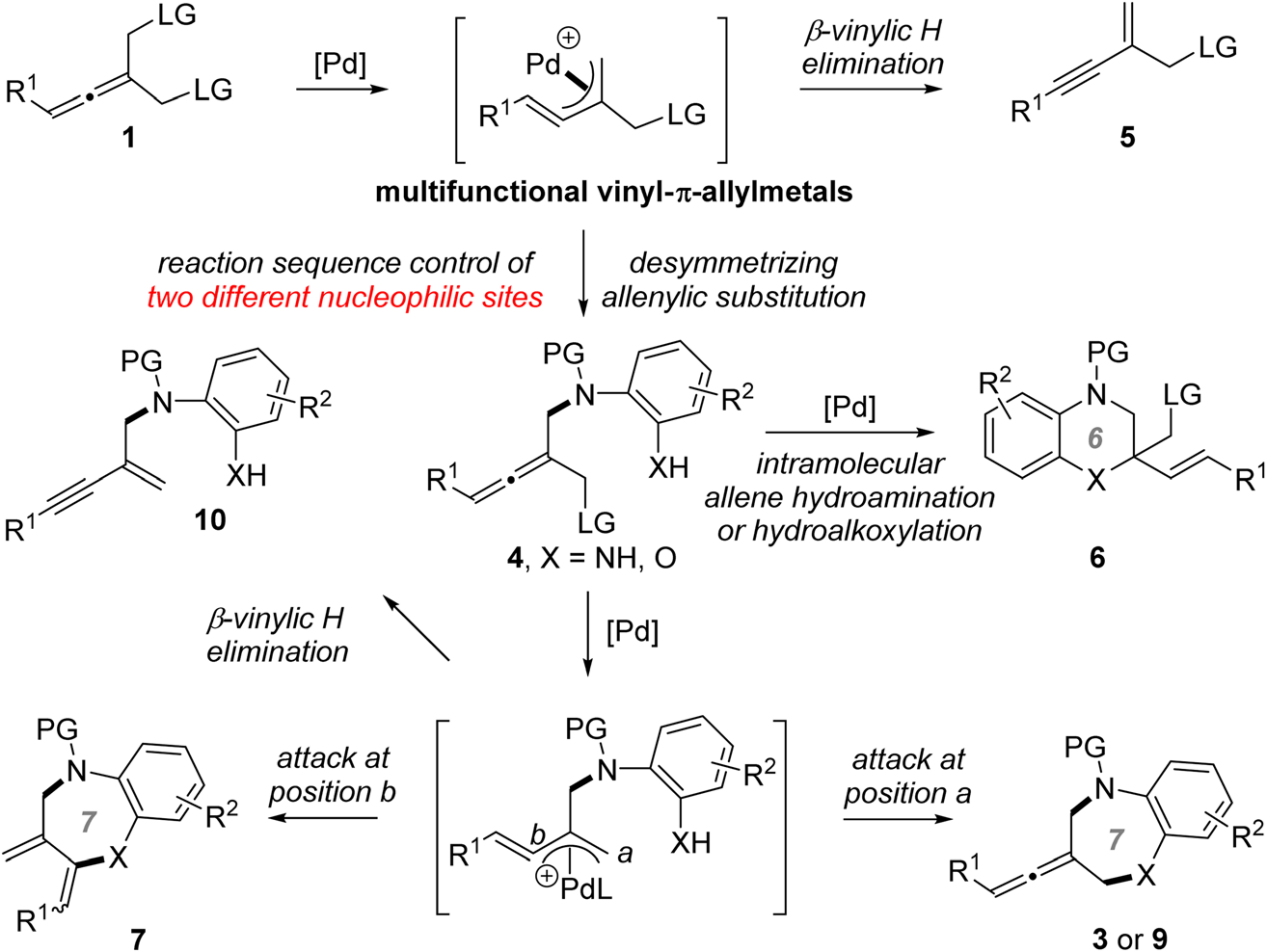
Versatile reactivity of prochiral allenes 1 & complex chemo- and regioselectivity in the reaction of 1 with di-nucleophiles. LG = leaving group.

Transition metal (TM)-catalyzed intermolecular enantioselective allenylic substitution, which involves vinyl-π-allylmetal intermediates, has grown into a valuable approach for chemical bond formation (Fig. 2a).^12^ However, TM-catalyzed enantioselective allenylic cycloaddition, which involves sequential intermolecular/intramolecular allenylic substitution, has not been developed (Fig. 2b), although such a transformation would offer a type of cycloaddition and provide a general platform for the straightforward synthesis of structurally diverse chiral cyclic allenes in a one-pot manner from simple and readily available starting materials. Meanwhile, different from racemic allenes bearing a single leaving group, prochiral allenes 1 bearing two allenylic leaving groups can participate in multiple competitive reaction pathways (see Fig. 3). Thus, achieving TM-catalyzed enantioselective allenylic cycloaddition requires a multifunctional chiral Pd catalyst that not only needs to be active in all the steps and can provide high levels of chemo- and regioselectivity to precisely promote both the intermolecular and the intramolecular allenylic substitution in a one-pot procedure, but can also effectively control the enantioselectivity of the whole process, particularly the intramolecular allenylic substitution step. It is noted that TM-catalyzed enantioselective intramolecular allenylic substitution has remained elusive yet challenging. To achieve high enantiocontrol, the chiral Pd catalyst is additionally required to be capable of much faster racemization of tri-substituted allenes (produced during the TM-catalyzed intermolecular allenylic substitution step) than that of the subsequent intramolecular allenylic substitution (cyclization) as well as uniquely effective enantiocontrol of the unknown intramolecular allenylic substitution. Yet, the intramolecular cyclization process is usually faster than the corresponding intermolecular process. In addition, the intramolecular process may require a specific property of the palladium catalyst or the chiral ligand which is different from the intermolecular process. Taken together, it is a difficult task to find a suitable catalyst that meets all the demands in the sequential reaction to effect a high-yielding and high enantioselective allenylic cycloaddition. Fortunately, an enabling Pd catalyst system with an electron-deficient chiral bidentate phosphite-type ligand, which was previously not utilized in the TM-catalyzed asymmetric allenylic substitution reaction, was successfully identified.

Besides, [4+3] allenylic cycloaddition products 3 and 9 easily undergo reversible C–N or C–O bond activation under Pd catalysis to lead to racemization. We found that through the addition of a catalytic amount of *ortho*-aminoanilines 2 or *ortho*-aminophenols 8, adverse racemization was completely controlled.

Prochiral allenes 1 possess versatile reactivities to be explored (Fig. 3), which provides an opportunity for the development of chemodivergent synthesis by a catalytic method. By switching the chiral ligand from L12 to L7, a Pd-catalyzed asymmetric desymmetric allenylic substitution reaction was developed, leading to axially chiral tri-substituted linear allenes 4. To our knowledge, this is the first example of TM-catalyzed enantioselective desymmetric transformations *via* an allenylic substitution in which two identical enantiotopic allenylic leaving groups were effectively differentiated. Reaction strategies allowing chemodivergence represent one of the most cutting-edge developments in synthetic organic chemistry and medicinal chemistry.^15^ To our knowledge, examples of TM-catalyzed enantioselective and chemodivergent desymmetric synthesis of allenes have remained elusive. Notably, it enables catalytic asymmetric and chemodivergent synthesis of two different types of axially chiral allenes, linear allenes and exocyclic allenes, from the same set of starting materials. Despite extensive efforts in axially chiral allene synthesis, such a method has remained elusive. On changing a nucleophilic site of double nucleophiles, we have achieved a previously unreported tandem desymmetric allenylic substitution/β-vinylic hydrogen elimination (formal enynylation) that provides a new method for multi-functionalized 1,3-enynes, which are subunits widely present in natural products and biologically active molecules, and are also versatile building blocks in organic synthesis.^16^ It is noted that β-hydrogen elimination of palladium complexes from C(sp^2^) rather than C(sp^3^) (*i.e.* β-vinylic hydrogen elimination) in palladium catalysis is scarce.

## Results and discussion

We began our studies by selecting prochiral allene 1a and *ortho*-aminoaniline 2a′ or 2a as model substrates under Pd catalysis. Chiral ligands, which have previously proven optimal in catalyzing enantioselective allenylic substitution reactions, were screened. Among them, only Trost ligand L7^12*a*^ was reactive, but it only promoted the intermolecular desymmetric allenylic substitution reaction and exclusively provided tri-substituted axially chiral linear allenes 4a′ in 75% yield and 90% ee (entry 8). Increasing the reaction temperature could not promote the subsequent intramolecular allenylic substitution to effect the desired [4+3] cycloaddition. Next, we screened other chiral ligands which have previously not been used in Pd-catalyzed allenylic substitution reactions. Most chiral ligands tested were unreactive. Chiral BABIBOP ligand^17^L5 provided the formal [3+4] cycloaddition product; however, no enantioinduction was observed (entry 5). We finally found that chiral bidentate phosphite-type ligand L12 can not only promote both the intermolecular and the intramolecular allenylic substitution in a one-pot procedure, but can also effectively control the enantioselectivity of the whole process (entry 14). Replacing the base K_2_CO_3_ with Na_2_CO_3_ led to 81% yield and 95% ee (entry 15) (Table 1).

**Table 1:** Selected optimization of the Pd-catalyzed chemo-divergent asymmetric reaction of 1a and 2a′ or 2a^a^

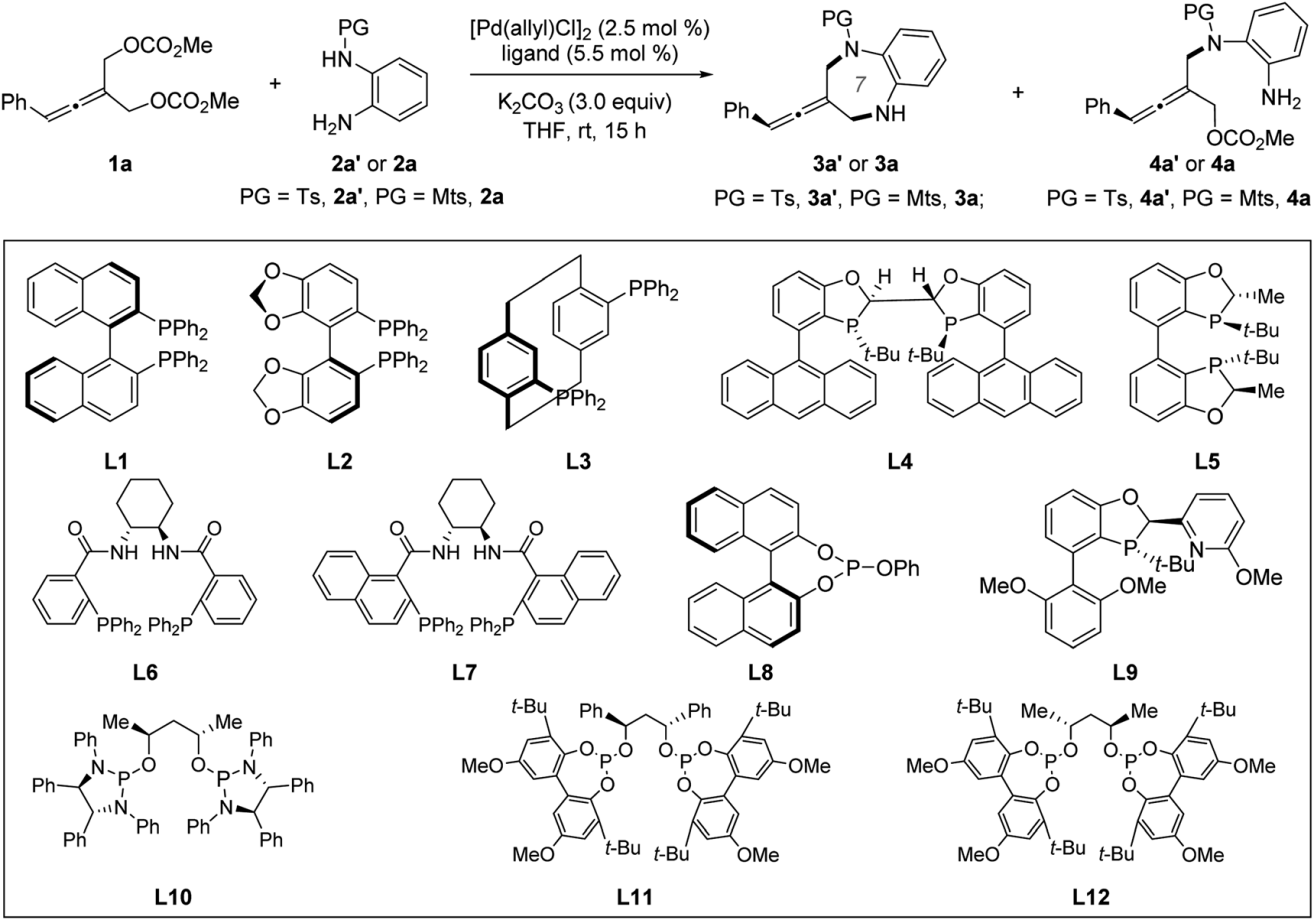						
Entry	Ligand	PG	3		4	
			Yield^b^ (%)	ee^c^ (%)	Yield^b^ (%)	ee^c^ (%)
1	L1	Ts	0	—	0	—
2	L2	Ts	0	—	0	—
3	L3	Ts	0	—	0	—
4	L4	Ts	0	—	0	—
5	L5	Ts	67	0	0	—
6	L6	Ts	0	—	Trace	—
7	L7	Ts	0	—	24	45
8^d^	L7	Mts	0	—	75	90
9^e^	L8	Ts	0	—	0	—
10	L9	Ts	0	—	0	—
11	L10	Ts	75	−55	0	—
12	L11	Ts	71	−69	0	—
13	L12	Ts	70	73	0	—
14	L12	Mts	91	85	0	—
15^f^	L12	Mts	81	95	0	—

a Reaction conditions: 1a (0.1 mmol), 2a′ or 2a (0.13 mmol), [Pd(allyl)Cl]_2_ (2.5 mol%), ligand (5.5 mol%), K_2_CO_3_ (3.0 equiv.) and THF (0.8 mL).

b Yield of the isolated product.

c Determined by chiral HPLC analysis.

d In TMB (1,3,5-trimethylbenzene) at 46 °C for 20 h.

e L8 (11.0 mol%) was used.

f Na_2_CO_3_ was used as the base. Ts = 4-methylbenzenesulfonyl. Mts = 2,4,6-trimethylbenzenesulfonyl.

With the optimized chiral catalyst systems in hand, we first investigated the scope of the Pd-catalyzed asymmetric allenylic [4+3] cycloaddition reaction (Table 2). A range of prochiral allenes underwent Pd-catalyzed [4+3] cycloaddition smoothly to provide seven-membered N-heterocycle-containing exocyclic allenes in good yields with good enantioselectivities (3a–3i). When R^1^ = primary alkyl, the corresponding [4+3] cycloaddition product 3j was obtained in good yield (86% yield) with moderate enantioselectivity (72% ee). In addition, several *ortho*-aminoanilines were also examined, providing the desired [4+3] cycloaddition products in good yields with high ee values (3k–3o). The absolute configuration of a seven-membered N-heterocycle-containing exocyclic axially chiral allene was determined by X-ray crystallographic analysis of the product 3n. Next, the Pd-catalyzed desymmetric asymmetric allenylic substitution reaction of prochiral allenes was investigated. The corresponding tri-substituted axially chiral linear allenes 4a–4e were obtained in good yields with good enantioselectivities in all cases examined.

**Table 2:** Pd-catalyzed chemodivergent asymmetric [4+3] cycloaddition and desymmetric allenylic substitution of 1 with 2^a^

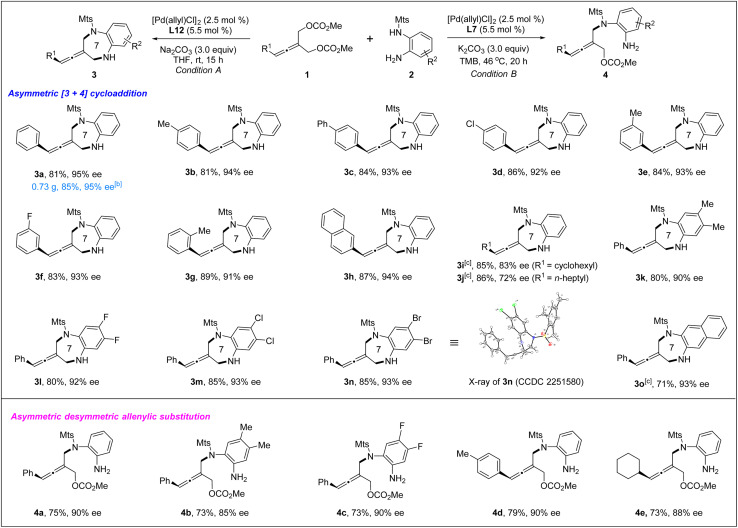

a Reaction conditions A: 1 (0.1 mmol), 2 (0.13 mmol), [Pd(allyl)Cl]_2_ (2.5 mol%), L12 (5.5 mol%), Na_2_CO_3_ (3.0 equiv.) in THF (0.8 mL) at rt for 15 h. Reaction conditions B: 1 (0.1 mmol), 2 (0.13 mmol), [Pd(allyl)Cl]_2_ (2.5 mol%), L7 (5.5 mol%), K_2_CO_3_ (3.0 equiv.) in TMB (1.0 mL) at 46 °C for 20 h. Isolated yields were reported and the enantiomeric excess was determined by chiral HPLC analysis.

b Scale-up reaction: 1a (2.0 mmol), 2a (2.6 mmol), [Pd(allyl)Cl]_2_ (2.5 mol%), L12 (5.5 mol%), Na_2_CO_3_ (3.0 equiv.) in THF (10 mL) at rt for 62 h.

c Li_2_CO_3_ (3.0 equiv.) as the base.

The absolute configuration of tri-substituted axially chiral linear allenes was determined by X-ray crystallographic analysis of the product 4f (eqn (1)).1
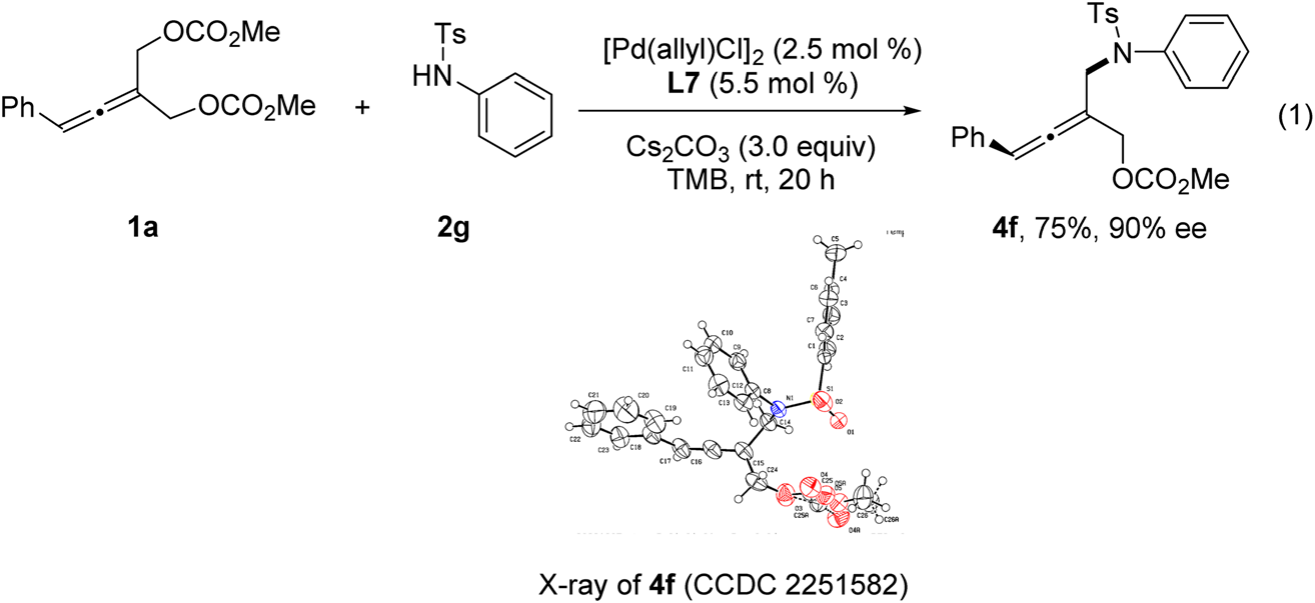


Next, we investigated the Pd-catalyzed chemodivergent reaction of prochiral allenes with *ortho*-aminophenols. Extension of allenylic [4+3] cycloaddition to *ortho*-aminophenols is interesting as TM-catalyzed enantioselective allenylic substitution with OH nucleophiles *via* C–O bond formation (to form axially chiral allenylic ethers) has remained elusive. The use of the ligand L12 offered the [4+3] cycloaddition product 9 in 80% yield with 53% ee (Table 3, entry 1). On changing the ligand from L12 to L11, an increase in enantioselectivity was observed (entry 2). We found that the N-protective group of *ortho*-aminophenol 8 had an important effect on the enantiocontrol of the [4+3] cycloaddition (see the ESI† for the details). When the N-protective group was replaced by 3,5-bis-trifluoromethyl-benzonesulfonyl, the [4+3] cycloaddition product 9 can be obtained in 94% yield and 88% ee (entry 3). Adjusting the ratio of substrates slightly improved the enantioselectivity to 90% ee (entry 4). Remarkably, a wide range of allene-containing 1,5-benzoxazepines could be obtained in good yields and enantioselectivities in general (Table 4).^18^

**Table 3:** Optimization of the Pd-catalyzed chemodivergent reaction of 1a with 8^a^

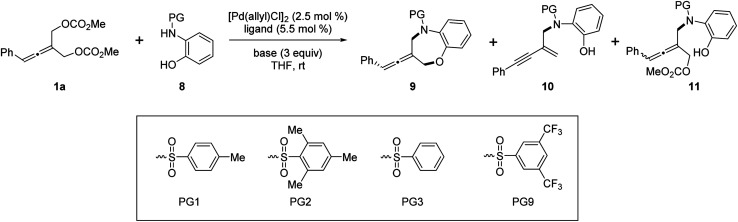						
Entry	Ligand	PG	Base	9		Yield of 10^b^
				Yield^b^ (%)	ee^c^ (%)	
1	L12	PG2	Na_2_CO_3_	80	−53	0
2	L11	PG2	Na_2_CO_3_	82	58	0
3	L11	PG9	Na_2_CO_3_	94	88	0
4^d^	L11	PG9	Na_2_CO_3_	87	90	0
5^e^	L7	PG9	K_2_CO_3_	Trace	—	0
6^e^	L7	PG2	K_2_CO_3_	Trace	—	0
7^f^^,^^g^	L9	PG3	K_2_CO_3_	10	—	38
8^f^^,^^h^	L9	PG3	K_2_CO_3_	—	—	85

a Reaction conditions: 1a (0.1 mmol), 8 (0.13 mmol), [Pd(allyl)Cl]_2_ (2.5 mol%), ligand (5.5 mol%), base (3.0 equiv.), in THF (0.8 mL) at rt for 5 h.

b Isolated yield.

c Determined by chiral HPLC analysis.

d 1a (0.25 mmol), 8 (0.1 mmol).

e In TMB (1.0 mL) at 46 °C for 20 h.

f Ligand (11.0 mol%) was used.

g At rt for 15 h.

h At 60 °C for 15 h.

**Table 4:** Pd-catalyzed chemodivergent [4+3] cycloaddition and enynylation of 1 and 8^a^

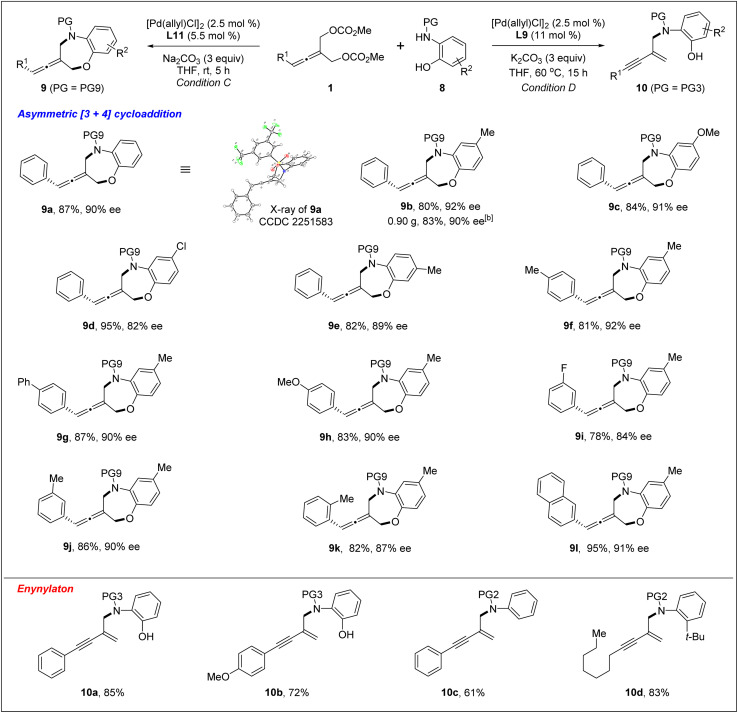

a Reaction conditions C: 1 (0.25 mmol), 8 (0.1 mmol), [Pd(allyl)Cl]_2_ (2.5 mol%), L11 (5.5 mol%), Na_2_CO_3_ (3.0 equiv.) in THF (0.8 mL) at rt for 5 h. Reaction conditions D: 1 (0.1 mmol), 8 (0.13 mmol), [Pd(allyl)Cl]_2_ (2.5 mol%), L9 (11 mol%), K_2_CO_3_ (3.0 equiv.), in THF (0.8 mL) at 60 °C for 15 h. Isolated yields were reported and the enantiomeric excess was determined by chiral HPLC analysis.

b Scale-up reaction: 1a (0.25 mmol), 8b (0.1 mmol), [Pd(allyl)Cl]_2_ (2.5 mol%), L11 (5.5 mol%), Na_2_CO_3_ (3.0 equiv.) in THF (10 mL) at rt for 32 h.

Very surprisingly, unlike *ortho*-aminoanilines, *ortho*-aminophenols could not undergo desymmetric allenylic substitution, indicating the interesting effect of the free hydroxyl group in the *ortho*-aminophenols 8 on the reaction. Unexpectedly, through utilizing the ligand L9,^19^ we achieved previously unreported tandem desymmetric allenylic substitution/β-vinylic hydrogen elimination (formal enynylation), leading to multi-functionalized 1,3-enyne 10 in 85% yield (Table 3, entry 8). This tandem reaction provided a useful method to synthesize 1,3-enynes (Table 4).

**Scheme 1:**
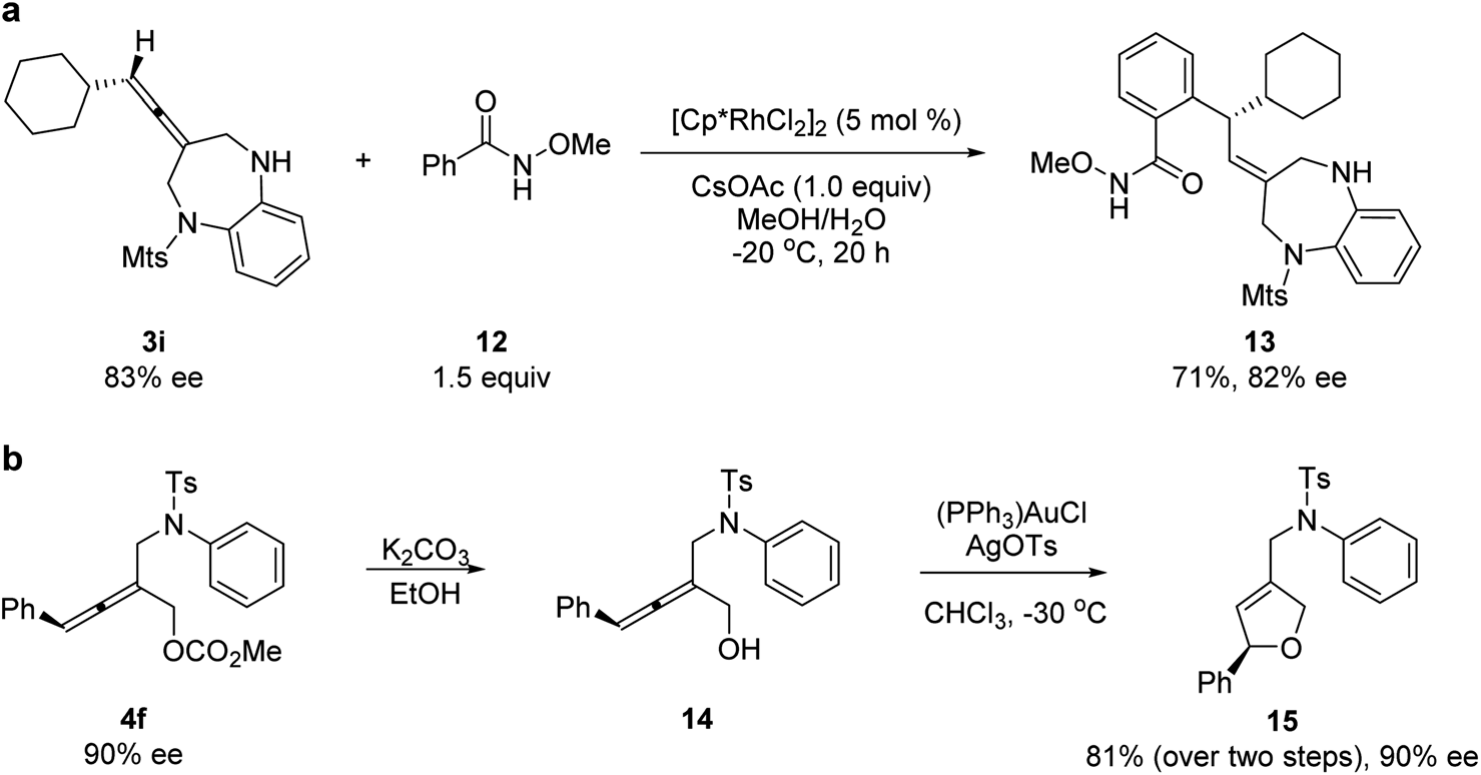
Synthetic transformations.

To demonstrate the utilities of the allene unit of the products, several transformations were conducted. A rhodium-catalyzed hydroarylation reaction of allene 3i with *N*-methoxybenzamide 12 provided chiral 1,5-benzodiazepine 13 (Scheme 1a).^20^ A chemoselective de-protection and a subsequent gold-catalyzed intramolecular hydroalkoxylation led to chiral dihydrofuran product 15 with complete axial-to-central chirality transfer (Scheme 1b).

**Scheme 2:**
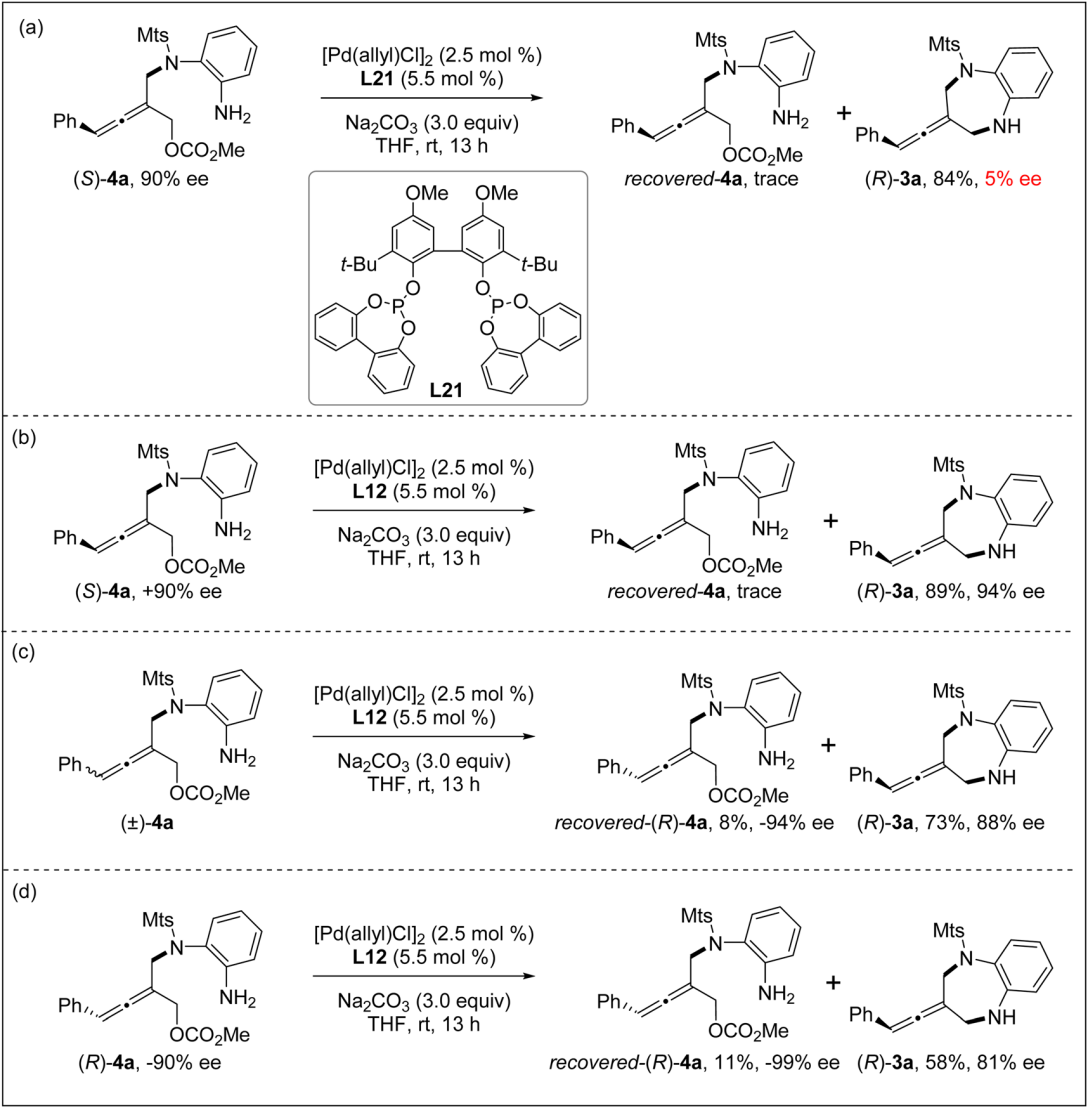
Control experiments.

In order to understand the reaction process, particularly the chiral control step of the formal [4+3] cycloaddition, several control experiments were conducted under the standard reaction conditions. The intramolecular allenylic substitution reaction of the linear allene intermediate 4a could not occur without Pd salt or a ligand. When the axially chiral tri-substituted linear allene intermediate 4a obtained through the intermolecular allenylic substitution by using chiral Trost ligand L7 was subjected to the Pd catalyst system with achiral bidentate phosphite ligand L21, the cyclization (*i.e.* intramolecular allenylic substitution) product 3a was almost completely racemic (Scheme 2a). These results indicate that the intramolecular allenylic substitution step of the linear allene intermediate 4a is not a stereoretentive or stereospecific process. In other words, the axial chirality of the linear allene intermediate 4a could not be preserved or transferred into the cyclization product 3a. Next, the intramolecular allenylic substitution reaction with (*S*)-4a, (±)-4a and (*R*)-4a was conducted, respectively (Schemes 2b–d). The results indicate that the absolute configuration of the cyclic allene product 3a is mainly controlled by the chirality of the ligand and the intramolecular allenylic substitution is the enantio-determining step. The relatively lower yield and enantioselectivity of the product 3a by using (*R*)-4a suggests that a combination of (*R*)-allene intermediate 4a and (*R*,*R*)-ligand L12 would be a mismatched pair, while the combination of (*S*)-allene intermediate 4a and (*R*,*R*)-ligand L12 would be a matched pair in the intramolecular allenylic substitution. Importantly, the stereochemical outcome of the allene intermediate 4a generated during the step of the intermolecular desymmetric allenylic substitution catalyzed by Pd/(*R*,*R*)-L12 was consistent with the subsequent Pd/(*R*,*R*)-L12-catalyzed asymmetric intramolecular allenylic substitution, leading to enantioselectivity enhancement.

**Scheme 3:**
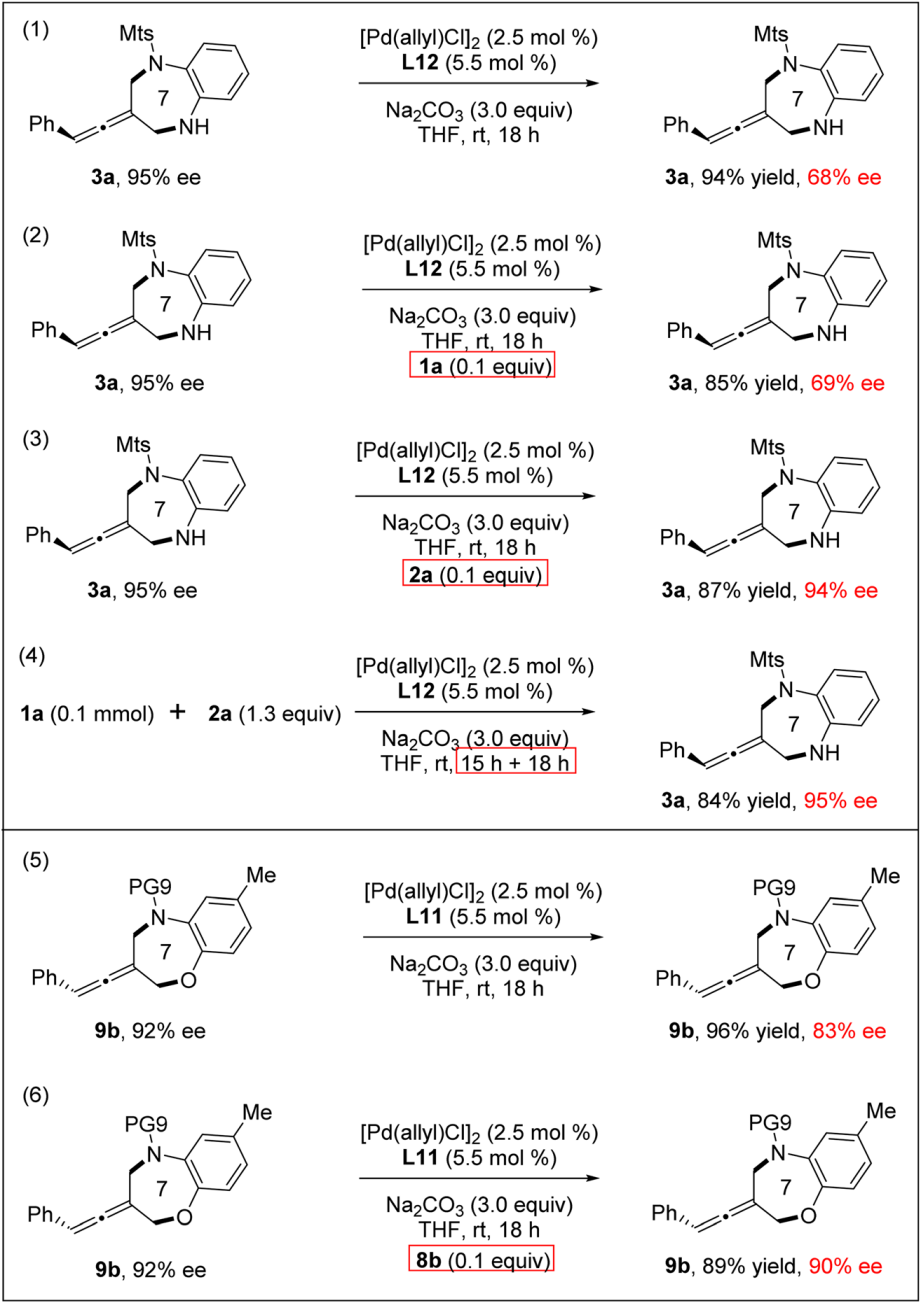
Racemization experiments of [4+3] cycloaddition products.

Next, reversibility and racemization experiments of the products 3a and 9b were explored, respectively, under standard conditions (Scheme 3). We found that 3a (95% ee) underwent the undesired racemization and 3a was recovered with a much decreased 68% ee. Interestingly, through the addition of a catalytic amount of *ortho*-aminoaniline 2a, the racemization of the product 3a was completely inhibited.^21^ Notably, the reaction between 1a and 2a did not result in racemization even on extending the reaction time, probably due to the presence of *ortho*-aminoaniline 2a in the reaction system inhibiting product racemization. Meanwhile, racemization also occurred in the reaction of the product 9b under standard reaction conditions. Through the addition of a catalytic amount of 8b, we can also effectively control the racemization of the product 9b.

**Fig. 4:**
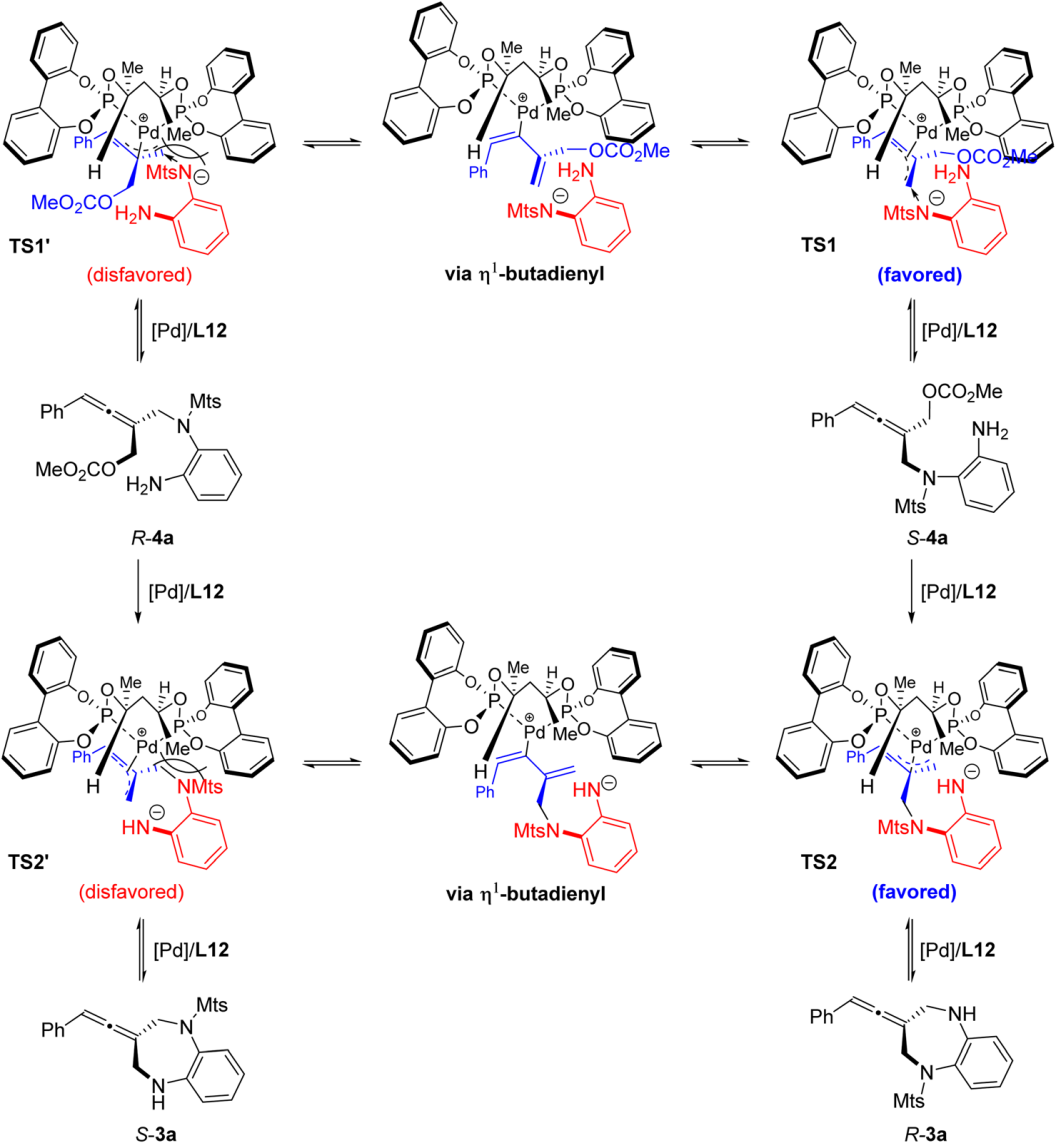
Proposed transition state model.

Based on the above experimental results and the absolute configuration of the product, a possible transition metal model is proposed (Fig. 4). The absolute configuration of the cyclic allene product 3a is mainly controlled by the chirality of the ligand *via* asymmetric intramolecular allenylic substitution which involves a dynamic kinetic resolution or asymmetric transformation. The stereochemical outcome of the allene intermediate 4a generated during the step of the intermolecular desymmetric allenylic substitution catalyzed by Pd/(*R*,*R*)-L12 was consistent with the subsequent Pd/(*R*,*R*)-L12-catalyzed asymmetric intramolecular allenylic substitution, leading to enantioselectivity enhancement.

## Conclusions

In summary, we have introduced prochiral allenes of type 1 as a new class of C3 synthons in cycloadditions and developed a new type of [4+3] cycloaddition involving previously unknown Pd-catalyzed enantioselective sequential allenylic substitution. This protocol provides a general method for chiral cyclic allenes enabling difficult-to-access seven-membered exocyclic axially chiral allenes. Despite multiple reactivity and selectivity issues and complex stereocontrol, an enabling Pd catalyst system was successfully identified to provide high levels of chemo-, regio-, and enantioselectivity in the reactions of prochiral allenes 1 with *ortho*-aminoanilines 2 and *ortho*-aminophenols 8, exclusively providing axially chiral allene-containing 1,5-benzodiazepines 3 and 1,5-benzoxazepines 9 in good yields with good enantioselectivity. Interestingly, the enantio-determining step in this allenylic cycloaddition was the elusive Pd-catalyzed enantioselective intramolecular allenylic substitution. Moreover, through the addition of a catalytic amount of *ortho*-aminoanilines or *ortho*-aminophenols, racemization of the [4+3] cycloaddition products was effectively controlled. Interestingly, a switch in chiral ligands from L12 to L7 resulted in a previously unreported Pd-catalyzed enantioselective desymmetric allenylic substitution, thus demonstrating the divergent reactivity of prochiral allenes 1. This work also constitutes an unprecedented catalytic enantioselective chemodivergent desymmetrization, and provides an elusive example of catalytic asymmetric chemodivergent synthesis of chiral cyclic allenes and linear allenes from the same set of starting materials. We believe that prochiral allenes 1 as C3 synthons and the newly developed cycloaddition strategy will find more applications in other cycloaddition reactions.

## Data availability

All experimental and characterization data in this manuscript are available in the ESI.† Crystallographic data for compounds 3n, 4f and 9a have been deposited at the CCDC and assigned the numbers 2251580, 2251582 and 2251583,† respectively.

## Author contributions

Z. S. conceived and directed the project. P. L., L. L., X. M. and Z. S. performed the experiments. Y. W., F. P., and Z. S. co-wrote the manuscript. All the authors discussed the results and commented on the manuscript.

## Conflicts of interest

There are no conflicts to declare.

## Supplementary Material

SC-014-D3SC04581A-s001

SC-014-D3SC04581A-s002
