# Supplementary material for: Chemodivergence in Pd-catalyzed desymmetrization of allenes: enantioselective [4+3] cycloaddition, desymmetric allenylic substitution and enynylation
Source: Chem Sci. 2023 Sep 21;14(39):10812–23. doi: 10.1039/d3sc04581a (PMC10566515; doi:10.1039/d3sc04581a)
Supplement: SC-014-D3SC04581A-s001 [file SC-014-D3SC04581A-s001.pdf]

Electronic Supplementary Material (ESI) for Chemical Science.
This journal is © The Royal Society of Chemistry 2023

Supporting Information

Chemodivergence in Pd-Catalyzed Desymmetrization of Allenes: Enantioselective [4+3] Cycloaddition, Desymmetric Allenylic Substitution and Enynylation

Pengfei Luo,[a] Long Li,[a] Xinfang Mao, Zheng Sun, Yingcheng Wang, Fangzhi Peng, and Zhihui Shao*

Key Laboratory of Medicinal Chemistry for Natural Resource, Ministry of Education, School of Chemical Science and Technology, Yunnan Provincial Center for Research & Development of Natural Products, and State Key Laboratory for Conservation and Utilization of Bio-Resources in Yunnan, Yunnan University, Kunming 650091, China
[a] These authors contributed equally.
E-mail: zhihui_shao@hotmail.com

Table of Contents

1. General information 1

2. Substrate synthesis 1

3. Optimization of reaction conditions 5

4. General condition 13

5. Transformations of the products 15

6. Procedures of control experiments 17

7. Single crystal X-ray diffraction data 23

8. References 27

9. Spectral data of products 28

10. NMR spectra 57

11. HPLC spectra 128

1. General information

Unless otherwise noted, all reactions in standard conditions were carried out under an argon atmosphere. Solvents were dried by standard methods under argon atmosphere. ^{1}H NMR, ^{13}C NMR and ^{19}F NMR spectra were recorded on 400 MHz, 500 MHz, 600 MHz instruments using $CDCl_3$ or CD_3OD as solvent. Chemical shifts of ^{1}H NMR were recorded in parts per million (ppm, *δ (ppm)*) relative to tetramethylsilane (*δ (ppm)* = 0.00 ppm) with the solvent resonance as an internal standard ($CDCl_3$: *δ (ppm)* = 7.26 ppm, CD_3OD: *δ (ppm)* = 3.31 ppm). NMR multiplicities are abbreviated as follows: s = singlet, d = doublet, t = triplet, m = multiple. High-resolution mass spectral analysis (HRMS) data were measured on a spectrometer by means of the ESI technique. The enantiomeric excess was determined by chiral HPLC with *n*-hexane and *i*-propanol as eluents. Optical rotations were measured on a polarimeter. Column chromatography was performed on silica gel (200–300 mesh).

2. Substrate synthesis

Synthesis of allenylic dicarbonates

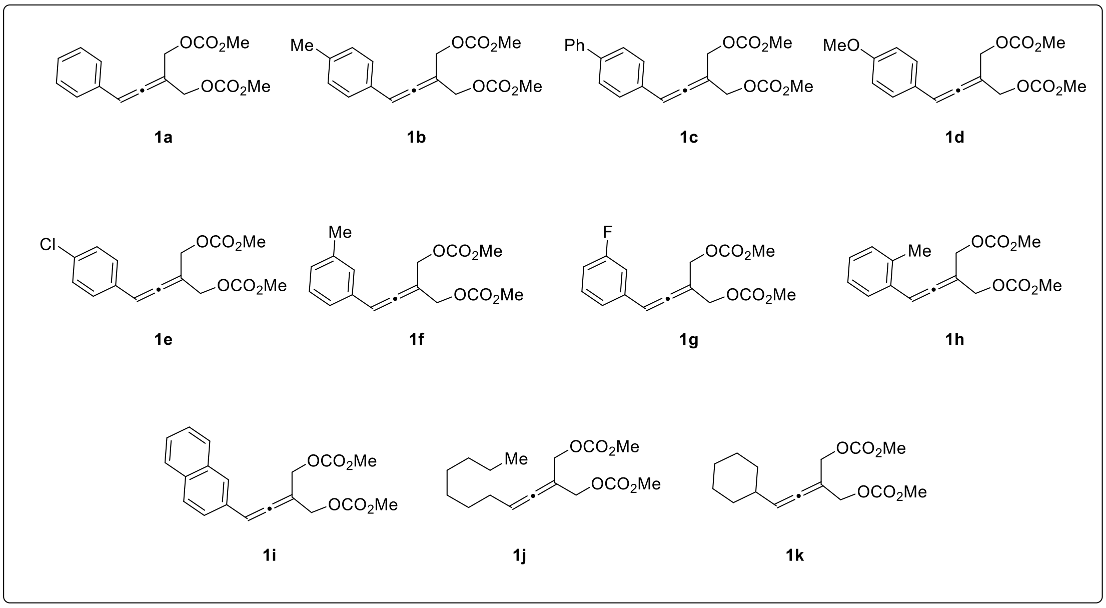

The substrates of **1a-1k** were synthesized according to modified procedure from Jan Deska and Ma et al.[1,2]

Procedure for synthesizing 1a-1i

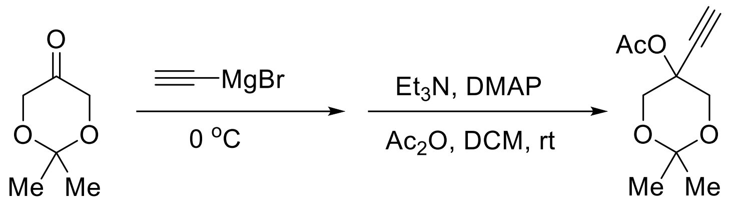

To an ice-cold solution of ethynylmagnesium bromide (0.5 M in THF, 250 mL, 125 mmol) a solution of 2,2-dimethyl-1,3-dioxan-5-one (13.0 g, 100 mmol, in 100 mL dry THF) was added dropwise at 0 °C over a period of one hour. After complete addition, the reaction mixture was carefully hydrolyzed with saturated aqueous NH_4Cl and ethyl acetate (200 mL) was added. The aqueous layer was extracted with ethyl acetate, the combined organic layers washed with sat. NaCl and dried over Na_2SO_4. After removal of the solvent in vacuo, the crude alcohol was dissolved in CH_2Cl_2 (10 mL), and triethylamine (16.7 mL, 120 mmol), DMAP (1.2 g, 10.0 mmol) and acetic anhydride (20 mL, 212 mmol) were added and the reaction mixture was stirred at rt overnight. After removal of triethylamine and excess acetic anhydride in vacuo, the residue was dissolved in ethyl acetate (200 mL), washed with 0.5 N HCl, saturated $NaHCO_3$ and brine and the organic layer was dried over Na_2SO_4. After concentration in vacuo, the crude acetate was purified by column chromatography (SiO_2, cyclohexane/ethyl acetate = 80:20) yielding as pale white solid (82% yield).

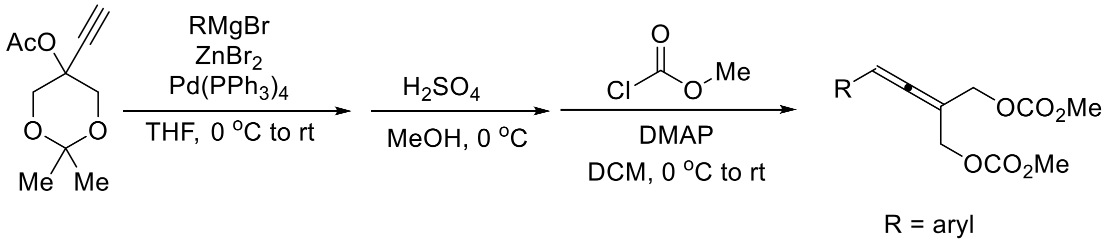

ZnBr$_2$ (2.44 g, 4.4 mmol) was carefully dried with a heatgun in vacuo until a fine powder formed. After cooling to rt, under Ar-atmosphere, Pd(PPh$_3$)$_4$ (20 mg, 0.06 mmol) was added, catalyst and zinc salt were dissolved in dry THF (15 mL) and cooled to 0 °C. A solution of the corresponding Grignard reagent (4 mmol, 0.5-1M in THF) was added dropwise and stirring at 0 °C was continued for 20 min where upon a white precipate formed. In a second flask, 5-acetoxy-2,2-dimethyl-5-ethynyl-1,3-dioxane (396 mg, 2.0 mmol) was dissolved in dry THF (4 mL) and added to the solution of the

organozinc reagent at 0 °C. The reaction mixture was allowed to warm to rt overnight. After addition of saturated NH_4Cl (10 mL) and ethyl acetate (20 mL), the aqueous phase was extracted with ethyl acetate (20 mL) and the combined organic layers were dried over Na_2SO_4 and filtered through a small plug of silica. The solvents were removed in vacuo, the residue redissolved in methanol (10 mL) and cooled to 0 ^{o}C. A drop of conc. sulfuric acid was added and the reaction mixture was stirred for approx. 30 min at 0 °C (TLC-control). Saturated $NaHCO_3$ (5 mL) and ethyl acetate (30 mL) were added, the aqueous layer was extracted with ethyl acetate (2 x 10 mL) and the combined organic layers were dried over Na_2SO_4. After removal of the solvents in vacuo, column chromatography (SiO_2, cyclohexane/ethyl acetate = 70:30 to 40:60) delivered the pure allendiols (72-95% yield). To a three-neck flask were added allendiols (1.64 mmol) prepared above, CH_2Cl_2 (30 mL), and DMAP (251.3 mg, 2.06 mmol) sequentially. The resulting mixture was stirred at 0 ^{o}C for 10 min followed by the dropwise addition of a solution of methyl chloroformate (0.32 mL, 4.1 mmol) in CH_2Cl_2 (5 mL) within 10 min at 0 ^{o}C. The resulting mixture was stirred at this temperature for 10 min, removed from the cooling bath, allowed to warm up to rt gradually, and continued to react at rt. After 6.0 h, the reaction was complete as monitored by TLC and quenched with H_2O (30 mL). The organic layer was separated, washed with an aqueous solution of hydrochloric acid (1M, 2 × 30 mL) and brine (30 mL) sequentially, and dried over anhydrous Na_2SO_4. After filtration, evaporation of the solvent and chromatography on silica gel (eluent: petroleum ether/ethyl acetate = 20:1) afforded allenylic dicarbonates **1a-1i** (76-92% yield).

Procedure for synthesizing 1g、1k

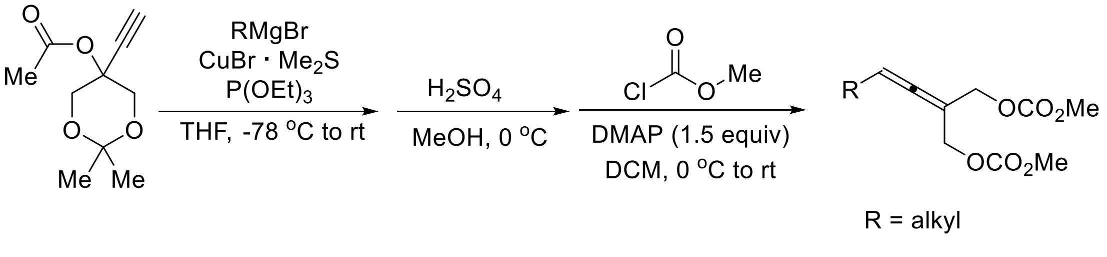

In an oven-dried Schlenk-flask under Ar-atmosphere, CuBr•Me_2S (41 mg, 0.2 mmol) was dissolved in dry THF (10 mL). Triethylphosphite (67 mg, 0.4 mmol) was added at rt whereupon the solid copper salt slowly dissolved. After addition of 5-acetoxy-2,2-dimethyl-5-ethynyl-1,3-dioxane (396 mg, 2.0 mmol) the solution was

cooled to -78 °C and a solution of the corresponding Grignard-reagent (4 mmol, 0.5-1M in THF) was added dropwise over a period of 2 hours. The reaction mixture was allowed to warm up to rt overnight. After addition of saturated NH_4Cl (10 mL) and ethyl acetate (20 mL), the aqueous phase was extracted with ethyl acetate (20 mL) and the combined organic layers were dried over Na_2SO_4 and filtered through a small plug of silica. The solvents were removed in vacuo, the residue redissolved in methanol (10 mL) and cooled to 0 °C. A drop of conc. sulfuric acid was added and the reaction mixture was stirred for approx. 30 min at 0 °C (TLC-control). Saturated $NaHCO_3$ (5 mL) and ethyl acetate (30 mL) were added, the aqueous layer was extracted with ethyl acetate (2 x 10 mL) and the combined organic layers were dried over Na_2SO_4. After removal of the solvents in vacuo, column chromatography (SiO_2, cyclohexane/ethyl acetate 7/3 to 4/6) delivered the pure allendiols (57-71% yield). To a three-neck flask were added allendiols (1.64 mmol) prepared above, CH_2Cl_2 (30 mL), and DMAP (251.3 mg, 2.06 mmol) sequentially. The resulting mixture was stirred at 0 ^{o}C for 10 min followed by the dropwise addition of a solution of methyl chloroformate (0.32 mL, 4.1 mmol) in CH_2Cl_2 (5 mL) within 10 min at 0 ^{o}C. The resulting mixture was stirred at this temperature for 10 min, removed from the cooling bath, allowed to warm up to rt gradually, and continued to react at rt. After 6 h, the reaction was complete as monitored by TLC and quenched with H_2O (30 mL). The organic layer was separated, washed with an aqueous solution of hydrochloric acid (1M, 2 × 30 mL) and brine (30 mL) sequentially, and dried over anhydrous Na_2SO_4. After filtration, evaporation of the solvent and chromatography on silica gel (eluent: petroleum ether/ethyl acetate = 30:1) afforded allenylic dicarbonates **1g**、**1k** (81-87% yield).

3. Optimization of reaction conditions

Optimization of reaction conditions of allenylic dicarbonate 1a with 2

Table S1: Chiral ligand catalyst screening[a]

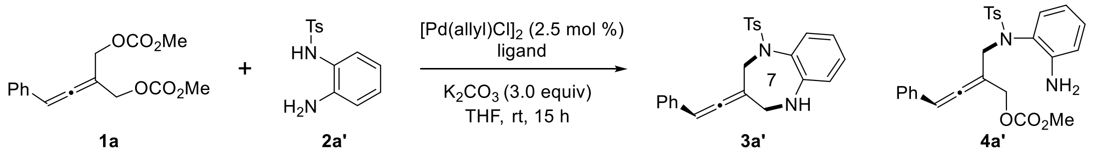

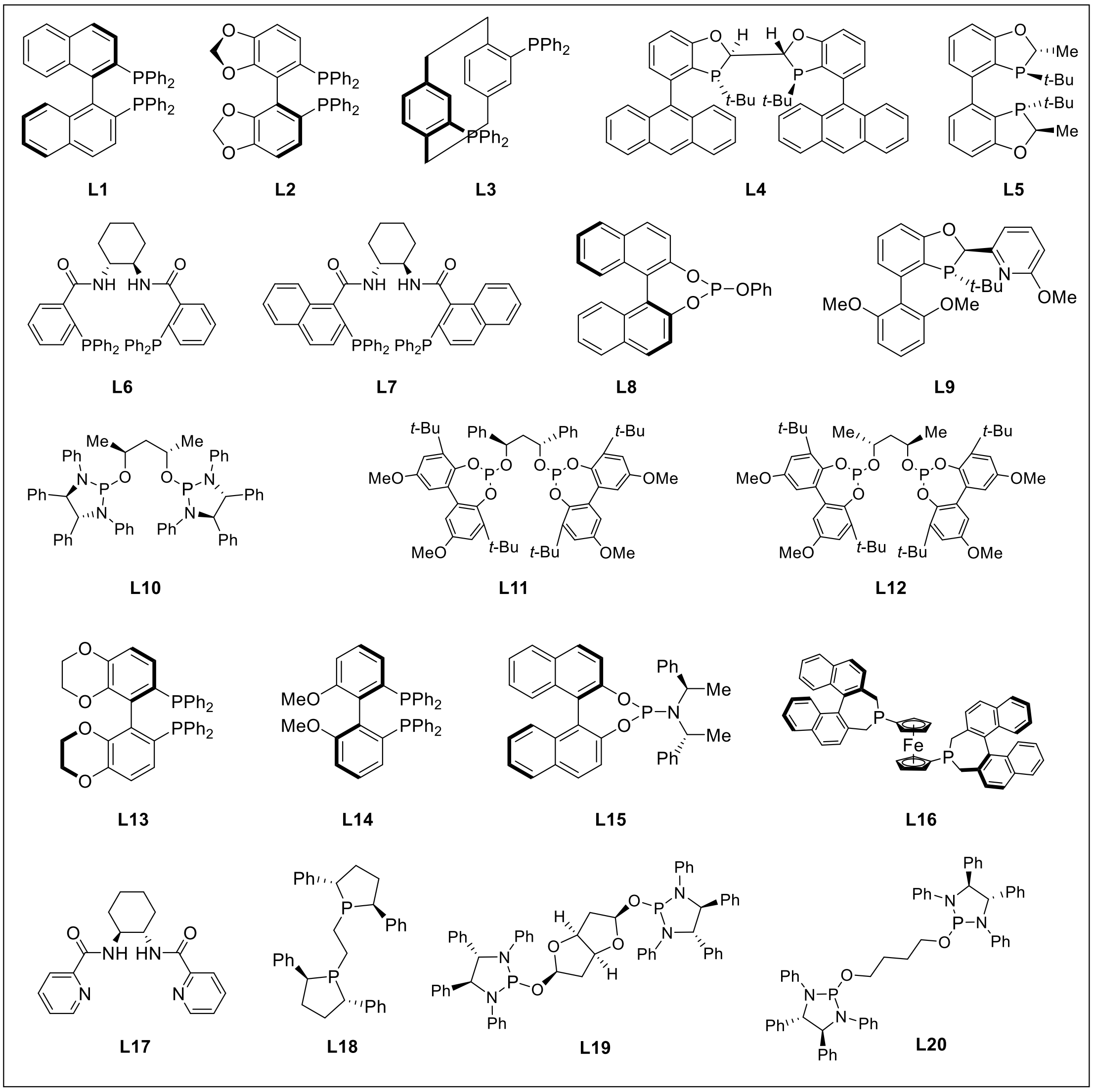

entry	ligand	3a'		4a'	
		yield (%)[b]	ee (%)[c]	yield (%)[b]	ee (%)[c]
1	**L1**	0	-	0	-
2	**L2**	0	-	0	-
3	**L3**	0	-	0	-
4	**L4**	0	-	0	-
5	**L5**	67	0	0	-

6	**L6**	0	-	trace	-
7	**L7**	0	-	24	45
8	**L8**	0	-	0	-
9	**L9**	0	-	0	
10	**L10**	75	-55	0	-
11	**L11**	71	-69	0	-
12	**L12**	70	73	0	-
13	**L13**	0	-	0	-
14	**L14**	0	-	0	-
15	**L15**	0	-	0	-
16	**L16**	0	-	0	-
17[d]	**L17**	0	-	0	-
18	**L18**	0	-	0	-
19	**L19**	0	-	0	-
20	**L20**	0	-	0	-

[a]Reaction conditions: **1a** (0.1 mmol), **2a'** (0.13 mmol), $[Pd(allyl)Cl]_2$ (2.5 mol %), ligand (monophosphines 11.0 mol %, diphosphines 5.5 mol %), K_2CO_3 (3.0 equiv) and THF (0.8 mL). [b]Yield of isolated product. [c]Determined by chiral HPLC analysis. [d]**L17** (5.5 mol %) was used. Ts = 4-Methylbenzenesulfonyl.

Table S2: Base screening[a]

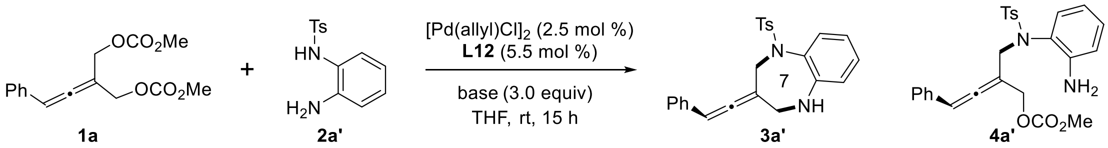

entry	base	3a'		4a'	
		yield (%)[b]	ee (%)[c]	yield (%)[b]	ee (%)[c]
1	Li_2CO_3	47	84	0	-
2	Na_2CO_3	54	87	0	-
3	K_2CO_3	70	73	0	-
4	K_3PO_4	47	73	0	-

[a]Reaction conditions: **1a** (0.1 mmol), **2a'** (0.13 mmol), $[Pd(allyl)Cl]_2$ (2.5 mol %), **L12** (5.5 mol %), base (3.0 equiv) and THF (0.8 mL). [b]Yield of isolated product. [c]Determined by chiral HPLC analysis.

Table S3: Protective group and base screening[a]

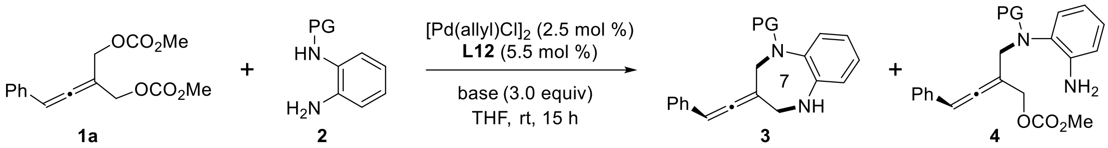

entry	PG	base	3		4	
			yield (%)[b]	ee (%)[c]	yield (%)[b]	ee (%)[c]
1	Ns	Na_2CO_3	72	86	0	-
2	Ns	K_2CO_3	93	85	0	-
3	Mts	Li_2CO_3	82	90	0	-
4	Mts	Na_2CO_3	81	95	0	-
5	Mts	K_2CO_3	91	85	0	-

[a]Reaction conditions: **1a** (0.1 mmol), **2** (0.13 mmol), $[Pd(allyl)Cl]_2$ (2.5 mol %), **L12** (5.5 mol %), base (3.0 equiv) and THF (0.8 mL). [b]Yield of isolated product. [c]Determined by chiral HPLC analysis. Ns = 4-Nitrobenzenesulfonyl, Mts = 2,4,6-Trimethylbenzenesulfonyl. PG = Protective Group.

Table S4: Solvents screening[a]

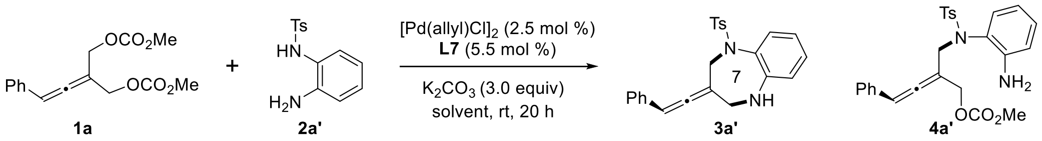

entry	solvent	3a'		4a'	
		yield (%)[b]	ee (%)[c]	yield (%)[b]	ee (%)[c]
1	MeCN	0	-	trace	-
2	PhMe	0	-	42	66
3[d]	PhMe	0	-	61	60
4	EtOAc	0	-	trace	-
5	MeOH	0	-	0	-
6	*n*-hexane	0	-	0	-
7	DCM	0	-	79	40
8	DCE	0	-	74	37

[a]Reaction conditions: **1a** (0.1 mmol), **2a'** (0.13 mmol), $[Pd(allyl)Cl]_2$ (2.5 mol %), **L7** (5.5 mol %), K_2CO_3 (3.0 equiv) and solvent (1.0 mL). [b]Yield of isolated product. [c]Determined by chiral HPLC analysis.[d]50 ^oC instead of rt.

Table S5: Screening of temperature and solvents[a]

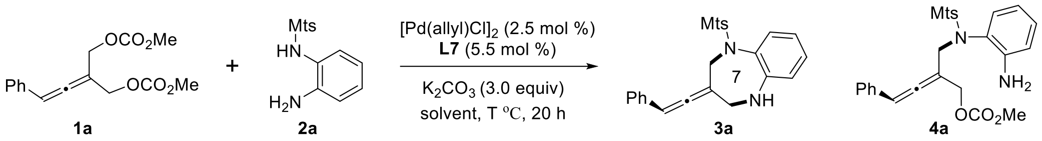

entry	T (°C)	solvent	3a		4a	
			yield (%)[b]	ee (%)[c]	yield (%)[b]	ee (%)[c]
1	50	PhMe	0	-	74	83
2	50	*o*-Xylene	0	-	71	87
3	50	*m*-Xylene	0	-	76	86
4	50	*p*-Xylene	0	-	74	86
5	50	TMB	0	-	83	88
6	46	TMB	0	-	75	90
7	40	TMB	0	-	52	93

[a]Reaction conditions: **1a** (0.1 mmol), **2a** (0.13 mmol), $[Pd(allyl)Cl]_2$ (2.5 mol %), **L7** (5.5 mol %), K_2CO_3 (3.0 equiv) and solvent (1.0 mL). [b]Yield of isolated product. [c]Determined by chiral HPLC analysis. TMB = 1, 3, 5-Trimethylbenzene. T = Temperature.

Table S6: Variable-temperature ^{1}H NMR experiments of product 4a (33 ^{o}C to -3.5 ^{o}C)

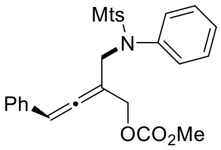

4a

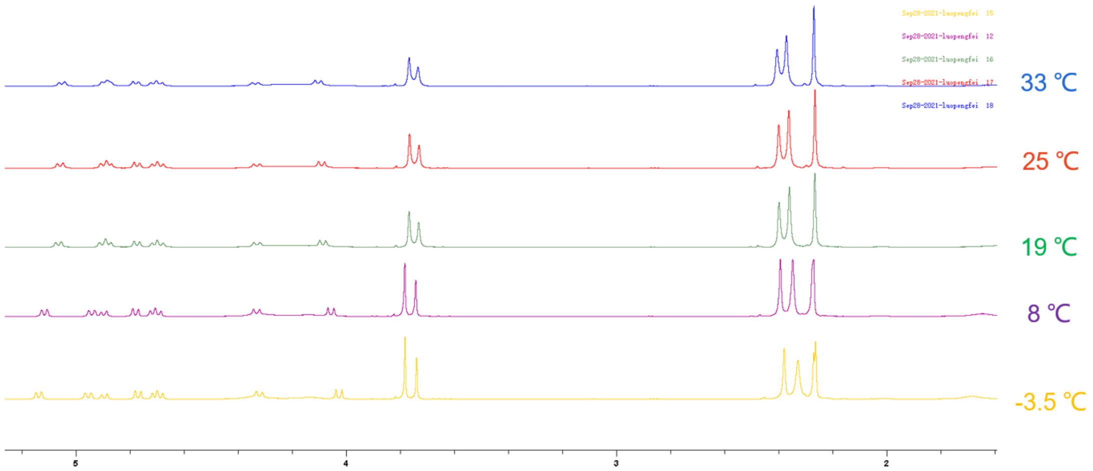

The high-temperature NMR experiments for the standard product **3a** were performed at 33 ^{o}C, which is cleaner than the corresponding spectra at -3.5 ^{o}C. For instance, the two peaks of the CH_3 group (at ~2.27 ppm) in ^{1}H NMR at -3.5 ^{o}C merged into one peak at high temperature.[3]

Optimization of reaction conditions of allenylic decarbonate 1a with 8

Optimal conditions for the use of *o*-phenylenediamine and allenylic dicarbonate[a]

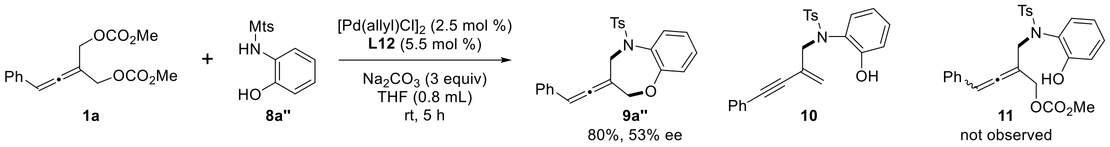

[a]Reaction conditions: **1a** (0.1 mmol), **8a''** (0.13 mmol), $[Pd(allyl)Cl]_2$ (2.5 mol %), **L12** (5.5 mol %), Na_2CO_3 (3.0 equiv) and THF (0.8 mL). isolated yields, the ee values were determined by chiral HPLC.

Table S7: Chiral ligand catalyst screening[a]

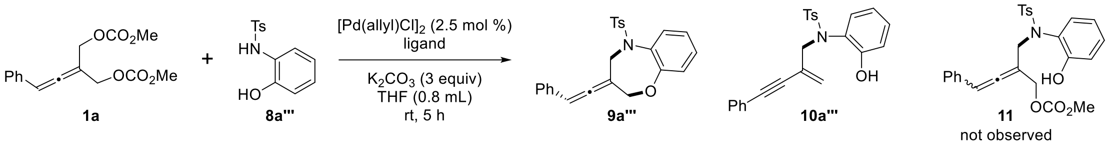

entry	ligand	**9a'''**		**10a'''**
		yield (%)[b]	ee (%)[c]	yield (%)[b]
1	**L1**	70	0	0
2	**L2**	71	0	0
3	**L3**	75	0	0
4	**L4**	<10	67	0
5	**L5**	27	0	0
6	**L6**	trace	-	0
7	**L7**	trace	-	0
8	**L8**	trace	-	0
9	**L9**	<10	-	<10
10	**L10**	82	44	0
11	**L11**	80	66	0
12	**L12**	84	-53	0
13	**L13**	71	0	0
14	**L14**	0	-	0
15	**L15**	83	0	0
16	**L16**	68	0	0
17	**L17**	0	-	0
18	**L18**	0	-	0
19	**L19**	81	3	0
20	**L20**	83	40	0

[a]Reaction conditions: **1a** (0.1 mmol), **8a'''** (0.13 mmol), $[Pd(allyl)Cl]_2$ (2.5 mol %), ligand (monophosphines 11.0 mol %, diphosphines 5.5 mol %), K_2CO_3 (3.0 equiv) and THF (0.8 mL). [b]Yield of isolated product. [c]Determined by chiral HPLC analysis.

Table S8: Base screening[a]

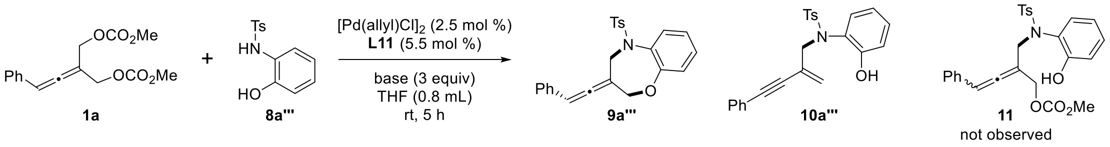

entry	base	9a'''		10a'''
		yield (%)[b]	ee (%)[c]	yield (%)[b]
1	-	0	-	0
2	Li_2CO_3	80	70	0
3	Na_2CO_3	85	71	0
4	K_2CO_3	81	66	0
5	Ru_2CO_3	84	47	0
6	Cs_2CO_3	86	53	0

[a]Reaction conditions: **1a** (0.1 mmol), **8a'''** (0.13 mmol), $[Pd(allyl)Cl]_2$ (2.5 mol %), **L11** (5.5 mol %), base (3.0 equiv) and THF (0.8 mL). [b]Yield of isolated product. [c]Determined by chiral HPLC analysis.

Table S9: Protective group screening[a]

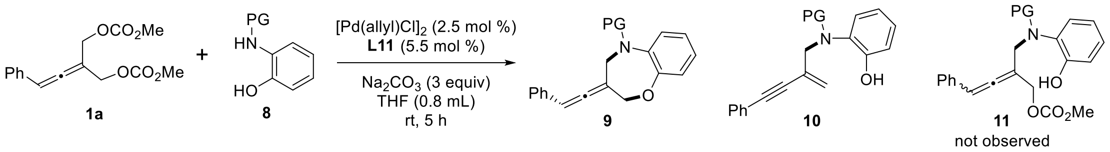

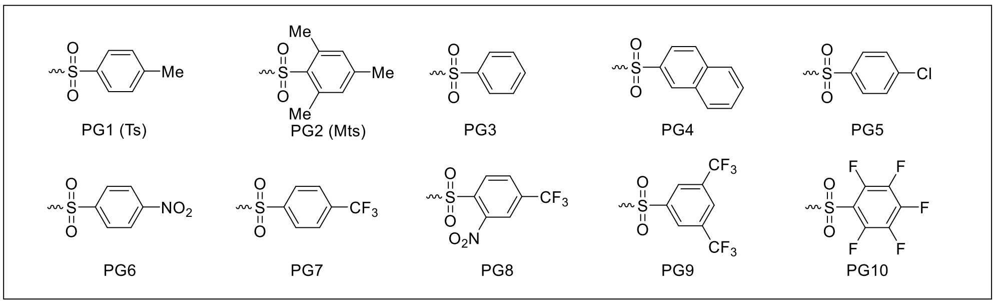

entry	PG	yield of **9** (%)[b]	ee of **9** (%)[c]	yield of **10** (%)[b]
1	PG1	85	71	0
2	PG2	82	58	0
3	PG3	82	73	0
4	PG4	87	76	0
5	PG5	86	75	0
6	PG6	91	75	0
7	PG7	87	80	0
8	PG8	82	86	0
9	PG9	94	88	0
10	PG10	84	85	0

[a]Reaction conditions: **1a** (0.1 mmol), **8** (0.13 mmol), [Pd(allyl)Cl]$_2$ (2.5 mol %), **L11** (5.5 mol %), Na_2CO_3 (3.0 equiv) and THF (0.8 mL). [b]Yield of isolated product. [c]Determined by chiral HPLC analysis.

Table S10: Screening of reactant ratio[a]

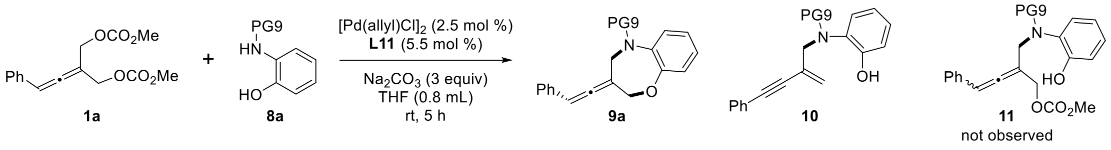

entry	**1a/8a** (mmol)	yield of **9a** (%)[b]	ee of **9a** (%)[c]	yield of **10** (%)[b]
1	0.20/0.10	92	84	0
2	0.15/0.10	92	87	0
3	0.13/0.10	94	88	0
4	0.10/0.10	85	83	0
5	0.10/0.15	86	84	0
6	0.10/0.20	88	88	0
7	0.10/0.25	87	90	0
8	0.10/0.30	88	88	0

[a]Reaction conditions: **1a**, **8a**, [Pd(allyl)Cl]$_2$ (2.5 mol %), **L11** (5.5 mol %), Na_2CO_3 (3.0 equiv) and THF (0.8 mL). [b]Yield of isolated product. [c]Determined by chiral HPLC analysis.

Table S11: Temperature screening[a]

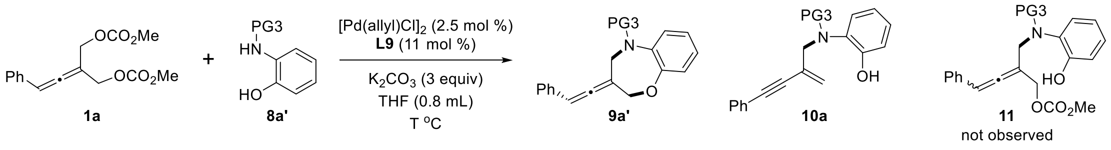

entry	T (°C)	yield of **9a'** (%)[b]	ee of **9a'** (%)	yield of **10a** (%)[b]
1	rt	10	-	38
2	60	-	-	85

[a]Reaction conditions: **1a** (0.1 mmol), **8a'''** (0.13 mmol), [Pd(allyl)Cl]$_2$ (2.5 mol %), **L9** (11 mol %), K_2CO_3 (3.0 equiv) and THF (0.8 mL). [b]Yield of isolated product.

4. General condition

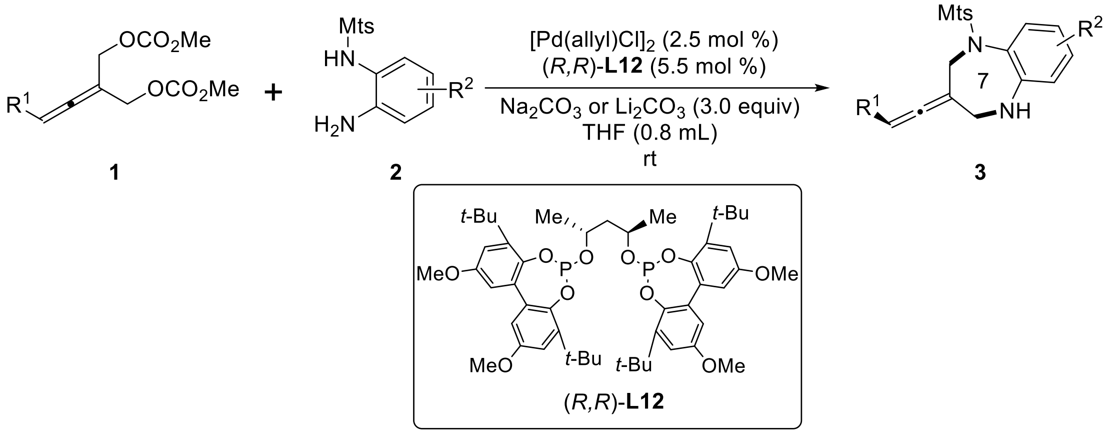

General condition A: In an Ar-filled glovebox, dissolving the [Pd(allyl)Cl]$_2$ (0.9 mg, 2.5 mol %), and **L12** (4.8 mg, 5.5 mol %) in THF (0.8 mL) was stirred for 15 min at rt. Subsequently, *o*-Phenylenediamine derivatives **2** (0.13 mmol, 1.3 equiv), **1** (0.1 mmol, 1 equiv), and Na_2CO_3 or Li_2CO_3 (3.0 equiv) were added. The reaction mixture was stirred outside the glove box and at rt for 15 hours. The solution was concentrated in vacuo and the crude product was purified by column chromatography on silica gel to afford the chiral product **3**.

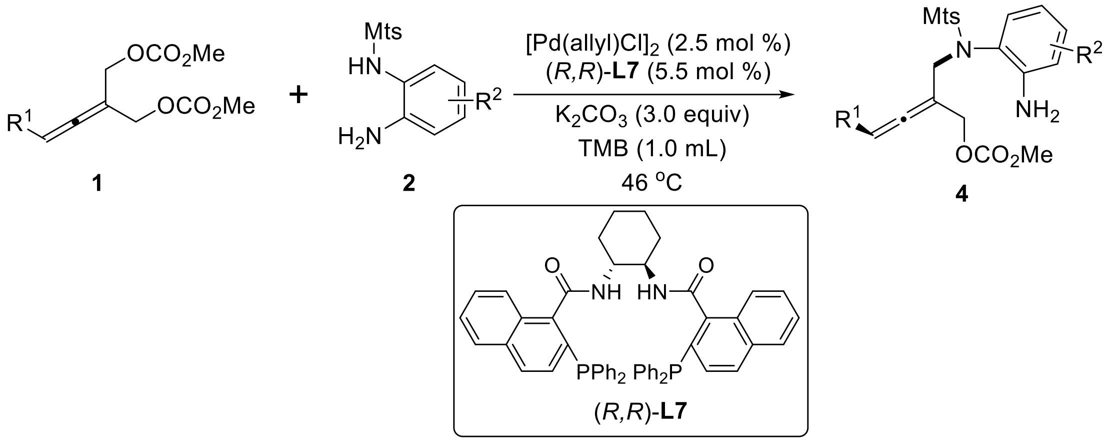

General condition B: In an Ar-filled glovebox, dissolving the [Pd(allyl)Cl]$_2$ (0.9 mg, 2.5 mol %), and **L7** (4.4 mg, 5.5 mol %) in TMB (1.0 mL) was stirred for 15 min at rt. Subsequently, *o*-Phenylenediamine derivatives **1** (0.1 mmol, 1 equiv), **2** (0.13 mmol, 1.3 equiv), and K_2CO_3 (3.0 equiv) were added. The reaction mixture was stirred outside the glove box and heated at 46 °C for 20 hours. The solution was concentrated in vacuo and the crude product was purified by column chromatography on silica gel to afford the chiral product **4**.

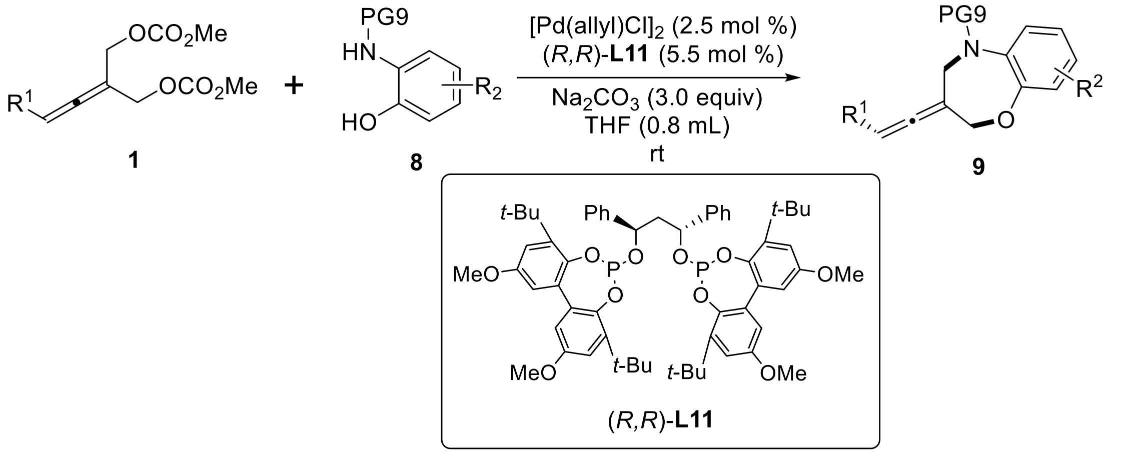

General condition C: In an Ar-filled glovebox, dissolving the [Pd(allyl)Cl]$_2$ (0.9 mg, 2.5 mol %), and **L11** (5.6 mg, 5.5 mol %) in THF (0.8 mL) was stirred for 15 min at rt. Subsequently, *o*-aminophenol derivatives **8** and **1**, and Na_2CO_3 were added. The reaction mixture was stirred outside the glove box and at rt for 5 hours. The solution was concentrated in vacuo and the crude product was purified by column chromatography on silica gel to afford the chiral product **9**.

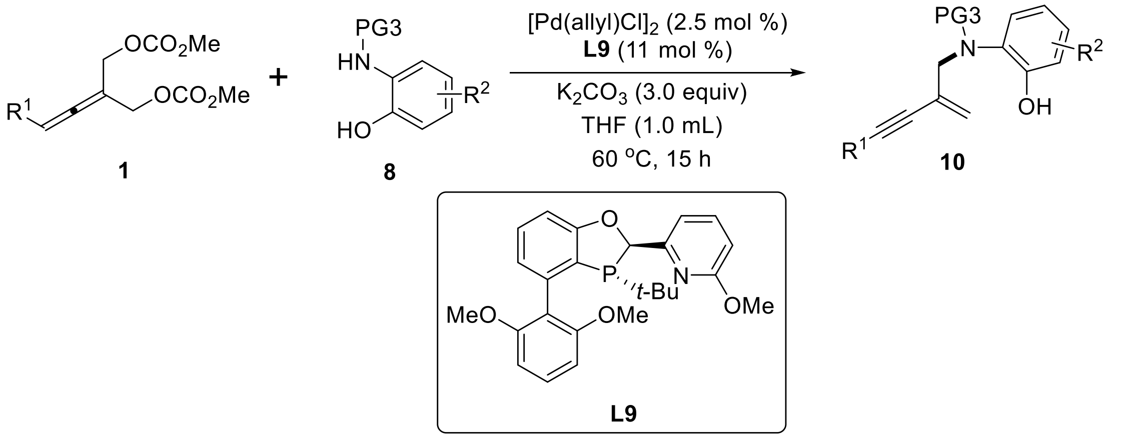

General condition D: In an Ar-filled glovebox, dissolving the [Pd(allyl)Cl]$_2$ (0.9 mg, 2.5 mol %), and **L9** (4.8 mg, 11 mol %) in THF (1.0 mL) was stirred for 15 min at rt. Subsequently, **8** (0.13 mmol, 1.3 equiv), **1** (0.1 mmol, 1.0 equiv), and K_2CO_3 (3.0 equiv) were added, at 60 ^oC for 15 h. The reaction mixture was stirred outside the glove box. The solution was concentrated in vacuo and the crude product was purified by column chromatography on silica gel to afford the product **10**.

5. Transformations of the products

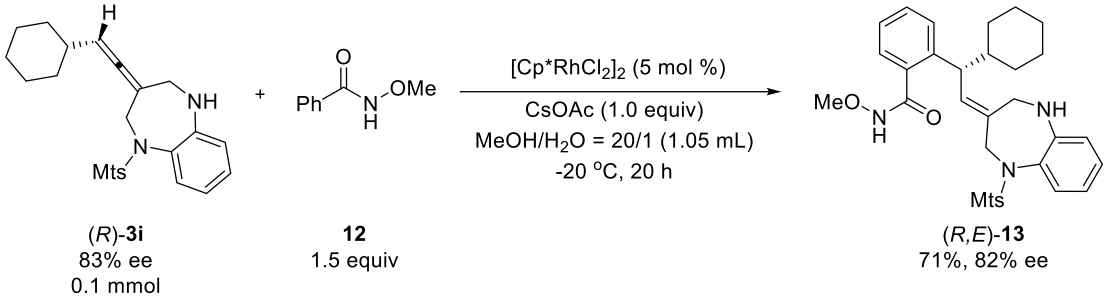

Transformations A: A dried Schlenk flask was charged with **12** (1.5 equiv), [Cp*RhCl$_2$]$_2$ (5 mol %), CsOAc (1.0 equiv) backfilled with argon for 3 times, a mixture of (*R*)-**3i** (43.6 mg, 0.1 mmol) in anhydrous MeOH/H_2O (1.05 mL) was added. The resulting mixture was stirred at -20 ^oC for 20 h. Upon completion, the reaction mixture was filtered through Celite® and the filtrate was evaporated under reduced pressure and purified by flash column chromatography (PE/EA = 3/1) to afford (*R*,*E*)-**13** (41.7 mg).

(*R*,*E*)-2-(1-cyclohexyl-2-(1-(mesitylsulfonyl)-1,2,4,5-tetrahydro-3*H*-benzo[*b*][1,4]diazepin-3-ylidene)ethyl)-*N*-methoxybenzamide (13)

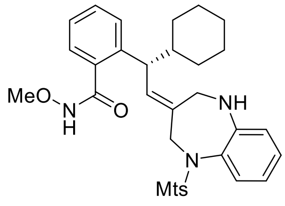

(*R*,*E*)-**13**

(Flash column chromatography eluent:petroleum ether/EtOAc = 3:1); yellow oily liquid, 41.7 mg, 71% yield, 82% ee;

^{1}H NMR (500 MHz, $CDCl_3$) δ (ppm) 8.37 (s, 1H), 7.36-7.33 (m, 1H), 7.24-7.15 (m, 3H), 6.85-6.83 (m, 3H), 6.49 (d, *J* = 7.8 Hz, 1H), 6.39 (t, *J* = 7.4 Hz, 1H), 6.30-6.28 (m, 1H), 5.57 (d, *J* = 10.0 Hz, 1H), 4.49-4.25 (m, 3H), 3.96 (d, *J* = 13.9 Hz, 1H), 3.87-3.85 (m, 4H), 3.58 (t, *J* = 10.0 Hz, 1H), 2.38 (s, 6H), 2.28 (s, 3H), 1.76-1.65 (m, 2H), 1.57 (b, 3H), 1.23-1.12 (m, 2H), 1.09-1.04 (m, 2H), 0.82-0.77 (m, 2H);

^{13}C NMR (125 MHz, $CDCl_3$) δ (ppm) 168.5, 146.9, 142.8, 142.1, 140.2, 134.6, 134.2, 132.9, 131.8, 131.7, 130.8, 130.4, 128.4, 128.1, 127.5, 125.8, 124.8, 118.2, 117.7, 64.5, 58.4, 47.0, 43.5, 43.2, 31.7, 31.1, 26.3, 26.2, 26.1, 22.8, 21.0;

HRMS (ESI) exact mass calcd. For $[C_{34}H_{42}N_3O_4S]^+$ requires *m/z* 588.2891, found *m/z* 588.2895;

$[\alpha]^{D}_{25}$ = 24.1 (*c* = 1.22, $CHCl_3$);

HPLC (Daicel Chirapak OD-3, hexane/ *i*-PrOH = 80/20, flow rate = 1.0 mL/min, T = 25 °C, 254 nm): t_R = 16.4 min (minor), t_R = 19.5 min (major).

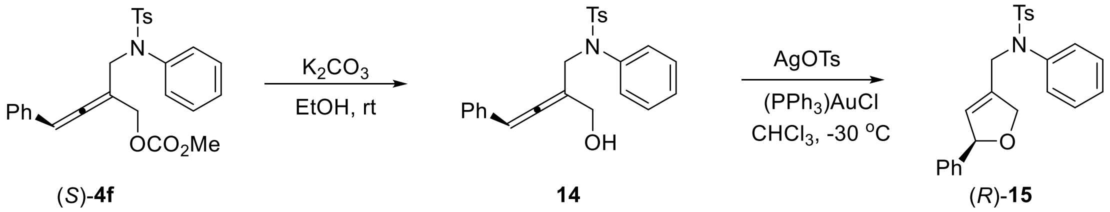

(*S*)-**4f** 0.1 mmol 90% ee — **14** — (*R*)-**15** two steps 81%, 90% ee

Transformations B: In a 10 mL screw-cap tube was placed tri-substituted allenyl acetates (*S*)-**4f** (0.1 mmol, 1.0 equiv., 46.3 mg) in MeOH (1.5 mL) under an air atmosphere. To the solution was added K_2CO_3 (0.2 mmol, 2.0 equiv., 27.6 mg) at room temperature in one portion. The solution was reacted at room temperature and monitored by TLC. After 2 h, the reaction mixture was filtered, concentrated and purified by silica gel column chromatography (PE/EA=4/1) to afford the desired product (*S*)-14 (36.5 mg, 90% yield) as a colorless oil.

To a dry Schlenk tube were added AgOTs (1.4 mg, 0.0045 mmol) in a glove box, PPh_3AuCl (2.8 mg, 0.0045 mmol), and $CHCl_3$ (1 mL) under argon atmosphere sequentially. After being stirred at room temperature for 30 min, the resulting mixture was stirred for another 10 min at -30 °C, then the **14** (36.5 mg, 0.09 mmol) and $CHCl_3$ (1 mL) were added. The resulting mixture was stirred at -30 °C for 12 h. The solution was purified by silica gel with 10:1 petroleum ether / ethyl acetate to afford compound **15** as a yellow oily liquid (32.8 mg, 90% yield).

(*R*)-4-methyl-*N*-phenyl-*N*-((5-phenyl-2,5-dihydrofuran-3-yl)methyl)benzenesulfonamide (15)

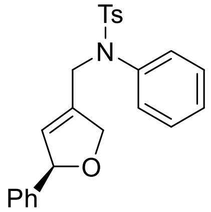

(*R*)-**15**

(Flash column chromatography eluent:petroleum ether/EtOAc = 7:1); yellow oily liquid, 32.8 mg, 81% yield, 90% ee;

^{1}H NMR (400 MHz, $CDCl_3$) δ (ppm) 7.47 (d, J = 8.3 Hz, 2H), 7.33-7.30 (m, 3H), 7.26-7.24 (m, 2H), 7.22-7.19 (m, 3H), 7.08-7.06 (m, 2H), 6.90-6.87 (m, 2H), 5.62-5.60 (m, 1H), 5.53-5.52 (m, 1H), 4.80-4.75 (m, 1H), 4.69-4.64 (m, 1H), 4.44 (d, J = 14.6 Hz, 1H), 4.30 (d, J = 14.5 Hz, 1H), 2.43 (s, 3H);

^{13}C NMR (100 MHz, $CDCl_3$) δ (ppm) 143.8, 141.2, 138.7, 135.7, 134.7, 129.5, 129.1, 129.0, 128.5, 128.4, 128.0, 127.9, 127.8, 126.6, 88.1, 75.8, 47.7, 21.6;

HRMS (ESI) exact mass calcd. For $[C_{24}H_{23}NO_3SNa]^+$ requires m/z 428.1291, found m/z 428.1290;

$[\alpha]^{D}_{25}$ = -13.5 (c = 0.61, $CHCl_3$);

HPLC (Daicel Chirapak AD-H, hexane/ i-PrOH = 75/25, flow rate = 1.0 mL/min, T = 25 °C, 254 nm): t_R = 6.2 min (minor), t_R = 7.1 min (major).

6. Procedures of control experiments

Table S12: Pd-catalyzed asymmetric [3 + 4] cycloaddition at different reaction times[a]

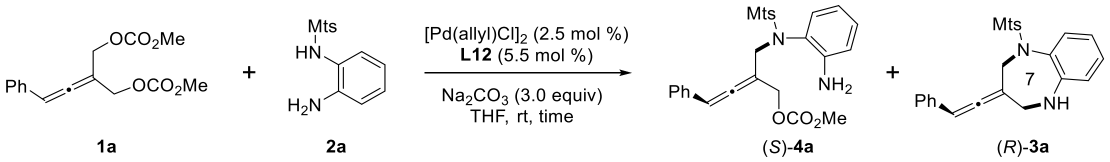

entry	time (h)	**1a** (yield) %[b]	(S)-**4a** (yield, ee) %[b, c]	**3a** (yield, ee) %[b, c]
1	0.17	43	16, 76	12, 84
2	0.5	5	24, 64	51, 93
3	2	trace	-	66, 92
4	15	-	-	81, 93

[a]Reactions were performed with **1a** (0.1 mmol), **2a** (0.13 mmol), $[Pd(allyl)Cl]_2$ (2.5 mol %), **L12** (5.5 mol %) and Na_2CO_3 (3.0 equiv) and THF (0.8 mL). [b]Yield of isolated product. [c]Determined by chiral HPLC analysis.

Table S13: Pd-catalyzed asymmetric intramolecular allenylic substitution of racemic allene 4a at different reaction times[a]

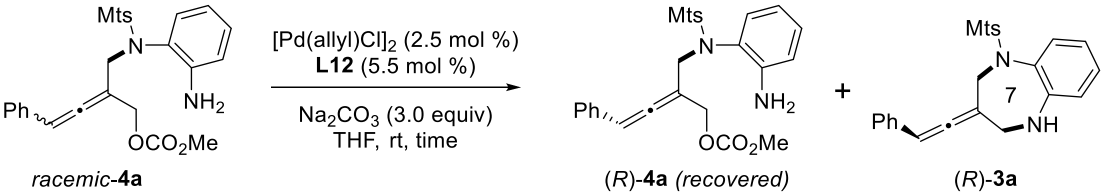

entry	time	**4a** (yield, ee) %[b, c]	**3a** (yield, ee) %[b, c]
1	1 h	32, -73	50, 86
2	6 h	22, -96	66, 90
3	13 h	8, -94	73, 88

Reactions were performed with *Racemic*-**4a** (0.1 mmol), $[Pd(allyl)Cl]_2$ (2.5 mol %), **L12** (5.5 mol %) and Na_2CO_3 (3.0 equiv) in 0.8 mL of THF at rt. [b]Yield of isolated product. [c]Determined by chiral HPLC analysis.

Table S14: Reactions with enantioenriched and racemic 4a.

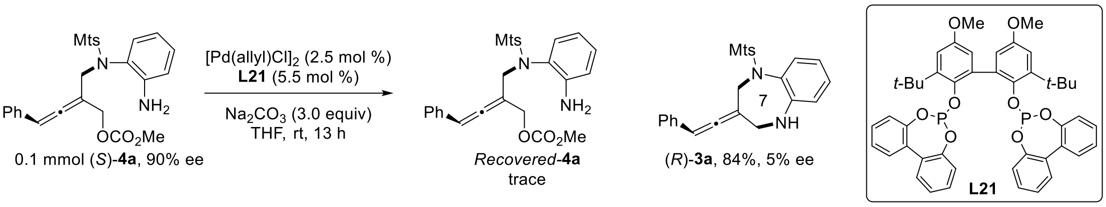

In an Argon-filled glovebox, ligand **L21** (4.3 mg, 5.5 mol %) and $[Pd(allyl)Cl]_2$ (0.9 mg, 2.5 mmol %) and THF (0.8 mL) were added to an oven-dried 10 mL screw-cap tube. After the resulting mixture was stirred at rt for 15 min, (*S*)-**4a** (0.1 mmol) and Na_2CO_3 (3.0 equiv) were added. The screw-cap tube was moved out of the glovebox and at rt for 13 hours. The reaction mixture was concentrated under reduced pressure. The residue was purified by flash column chromatography on silica gel to afford the product (*R*)-**3a** (36.1 mg, 84%, 5% ee).

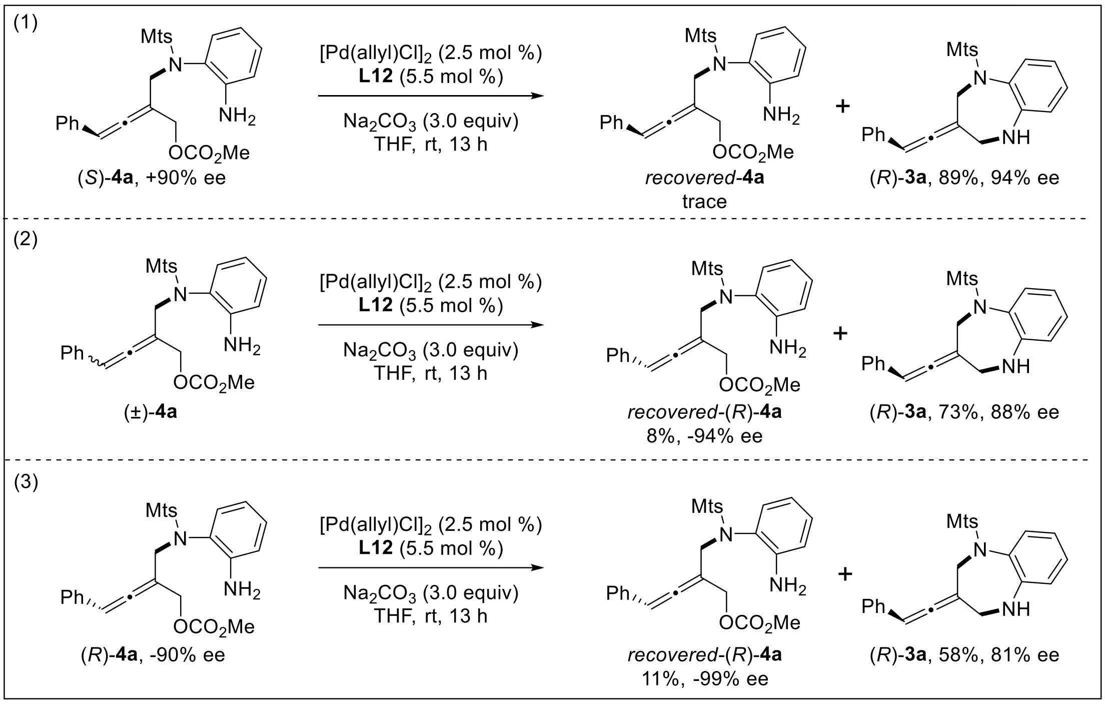

In an Argon-filled glovebox, ligand **L12** (4.8 mg, 5.5 mol %) and $[Pd(allyl)Cl]_2$ (0.9 mg, 2.5 mmol %) and THF (0.8 mL) were added to an oven-dried 10 mL screw-cap tube. After the resulting mixture was stirred at rt for 15 min, (*S*) or *racemic* or (*R*)-**4a** (0.1 mmol) and Na_2CO_3 (3.0 equiv) were added. The screw-cap tube was moved out of the glovebox and at rt for 13 hours. The reaction mixture was concentrated under reduced pressure. The residue was purified by flash column chromatography on silica gel to afford the product **3a** and recovered **4a**.

When the starting material was (*S*)-**4a**, afford the desired product (*R*)-**3a** (89% yield, 94% ee); When the starting material was *racemic*-**4a**, afford the desired product (*R*)-**3a** (73% yield, 88% ee) and recovered (*R*)-**4a** (8% yield, -94% ee); When the starting material was (*R*)-**4a**, afford the desired product (*R*)-**3a** (58% yield, 81% ee) and recovered (*R*)-**4a** (11% yield, -99% ee).

Intermediate 4a in the absence of metal ligands:

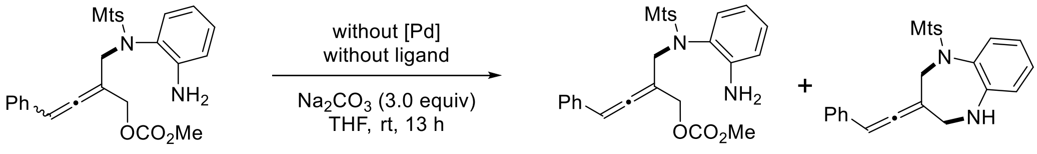

(±)-4**a**

recovered-**4a**
95%, 0% ee

3a
0

Table S15: Pd-catalyzed 10a reactions of use general condition C

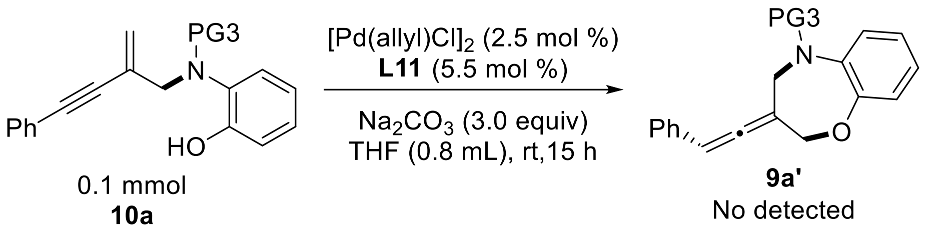

In an Argon-filled glovebox, ligand **L11** (5.6 mg, 5.5 mol %) and $[Pd(allyl)Cl]_2$ (0.9 mg, 2.5 mmol %) and THF (0.8 mL) were added to an oven-dried 10 mL screw-cap tube. After the resulting mixture was stirred at rt for 15 min, **10a** (0.1 mmol) and Na_2CO_3 (3.0 equiv) were added. The screw-cap tube was moved out of the glovebox and at rt for 15 hours. The reaction mixture was concentrated under reduced pressure. The residue was purified by flash column chromatography on silica gel to recovered **10a** (89% yield), not observed **9a'**.

Reversibility and racemization experiments of [3 + 4] cycloaddition products

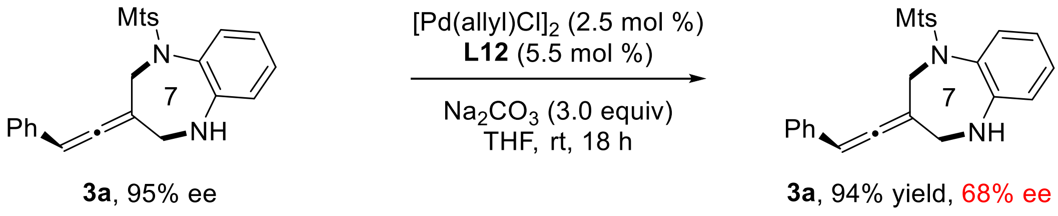

In an Argon-filled glovebox, ligand **L12** (4.8 mg, 5.5 mol %) and $[Pd(allyl)Cl]_2$ (0.9 mg, 2.5 mmol %) and THF (0.8 mL) were added to an oven-dried 10 mL screw-cap tube. After the resulting mixture was stirred at rt for 15 min, (*R*)-**3a** (0.1 mmol, 95% ee) and Na_2CO_3 (3.0 equiv) were added. The screw-cap tube was moved out of the glovebox and at rt for 18 hours. The reaction mixture was concentrated under reduced pressure. The residue was purified by flash column chromatography on silica gel to recovered (*R*)-**3a** (40.4 mg, 94%, 68% ee).

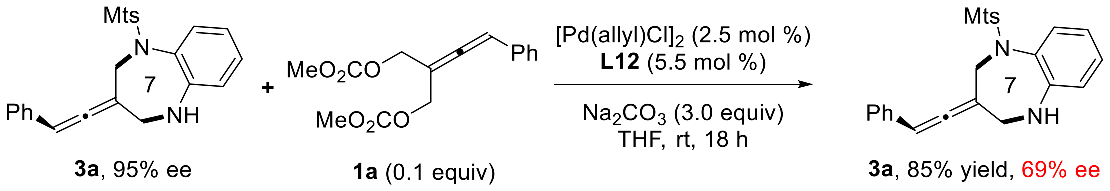

In an Argon-filled glovebox, ligand **L12** (4.8 mg, 5.5 mol %) and $[Pd(allyl)Cl]_2$ (0.9 mg, 2.5 mmol %) and THF (0.8 mL) were added to an oven-dried 10 mL screw-cap tube.

After the resulting mixture was stirred at rt for 15 min, (*R*)-**3a** (0.1 mmol, 95% ee) and **1a** (0.1 equiv) and Na_2CO_3 (3.0 equiv) were added. The screw-cap tube was moved out of the glovebox and at rt for 18 hours. The reaction mixture was concentrated under reduced pressure. The residue was purified by flash column chromatography on silica gel to recovered (*R*)-**3a** (36.6 mg, 85%, 69% ee).

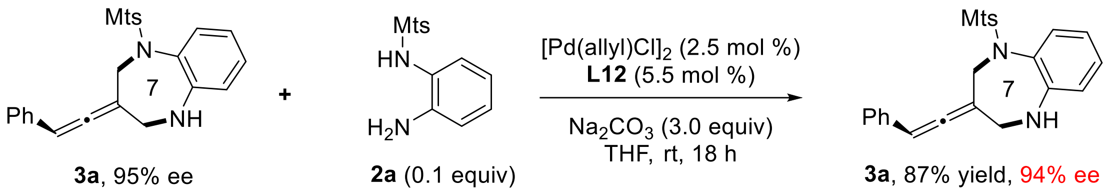

In an Argon-filled glovebox, ligand **L12** (4.8 mg, 5.5 mol %) and $[Pd(allyl)Cl]_2$ (0.9 mg, 2.5 mmol %) and THF (0.8 mL) were added to an oven-dried 10 mL screw-cap tube. After the resulting mixture was stirred at rt for 15 min, (*R*)-**3a** (0.1 mmol, 95% ee) and **2a** (0.1 equiv) and Na_2CO_3 (3.0 equiv) were added. The screw-cap tube was moved out of the glovebox and at rt for 18 hours. The reaction mixture was concentrated under reduced pressure. The residue was purified by flash column chromatography on silica gel to recovered (*R*)-**3a** (37.5 mg, 87%, 94% ee).

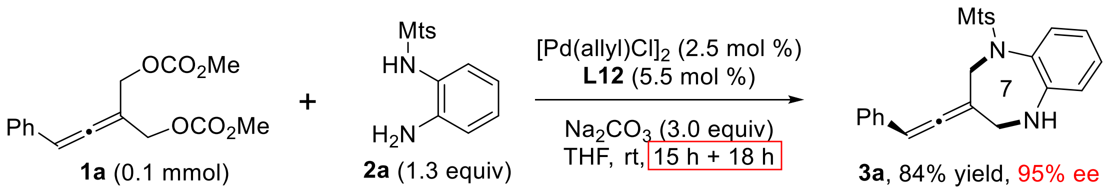

In an Argon-filled glovebox, ligand **L12** (4.8 mg, 5.5 mol %) and $[Pd(allyl)Cl]_2$ (0.9 mg, 2.5 mmol %) and THF (0.8 mL) were added to an oven-dried 10 mL screw-cap tube. After the resulting mixture was stirred at rt for 15 min, **1a** (0.1 mmol) and **2a** (1.3 equiv) and Na_2CO_3 (3.0 equiv) were added. The screw-cap tube was moved out of the glovebox and at rt for 33 hours. The reaction mixture was concentrated under reduced pressure. The residue was purified by flash column chromatography on silica gel to afford the product (*R*)-**3a** (36.2 mg, 84%, 95% ee).

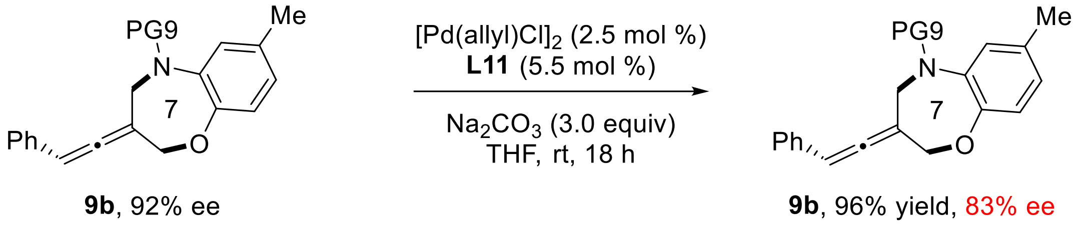

In an Argon-filled glovebox, ligand **L11** (5.6 mg, 5.5 mol %) and $[Pd(allyl)Cl]_2$ (0.9 mg, 2.5 mmol %) and THF (0.8 mL) were added to an oven-dried 10 mL screw-cap tube. After the resulting mixture was stirred at rt for 15 min, (*R*)-**9b** (0.1 mmol, 92% ee) and Na_2CO_3 (3.0 equiv) were added. The screw-cap tube was moved out of the glovebox and at rt for 18 hours. The reaction mixture was concentrated under reduced pressure. The residue was purified by flash column chromatography on silica gel to recovered (*R*)-**9b** (51.7 mg, 96%, 83% ee).

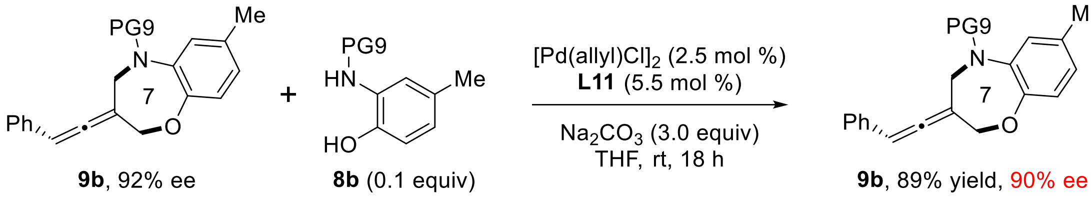

In an Argon-filled glovebox, ligand **L11** (5.6 mg, 5.5 mol %) and $[Pd(allyl)Cl]_2$ (0.9 mg, 2.5 mmol %) and THF (0.8 mL) were added to an oven-dried 10 mL screw-cap tube. After the resulting mixture was stirred at rt for 15 min, (*R*)-**9b** (0.1 mmol, 92% ee) and **8b** (0.1 equiv) and Na_2CO_3 (3.0 equiv) were added. The screw-cap tube was moved out of the glovebox and at rt for 18 hours. The reaction mixture was concentrated under reduced pressure. The residue was purified by flash column chromatography on silica gel to recovered (*R*)-**9b** (47.9 mg, 89%, 90% ee).

Table S16: 1a and 8 are reacted using condition B

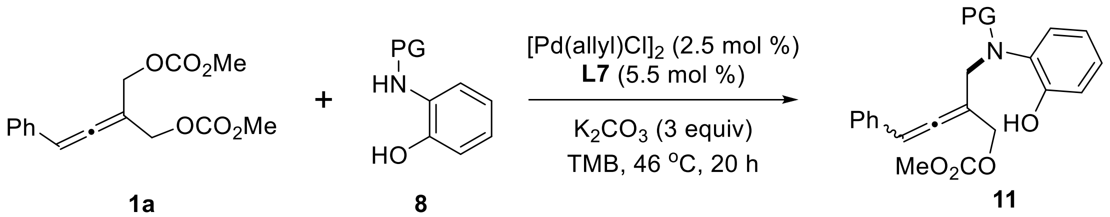

entry	PG	yield of **11** (%)[b]
1	PG9	0
2	PG2	0

In an Argon-filled glovebox, ligand **L7** (4.4 mg, 5.5 mol %) and $[Pd(allyl)Cl]_2$ (0.9 mg, 2.5 mmol %) and TMB (0.8 mL) were added to an oven-dried 10 mL screw-cap tube.

After the resulting mixture was stirred at rt for 15 min, **1a** (0.1 mmol) and **8** (1.3 equiv) and K_2CO_3 (3.0 equiv) were added. The screw-cap tube was moved out of the glovebox and at 46 °C for 20 hours. The reaction mixture is condensed under pressure reduction. The residue was purified by silica gel flash column chromatography and no **11** was detected.

Table S17: 1a and 2 are reacted using condition D

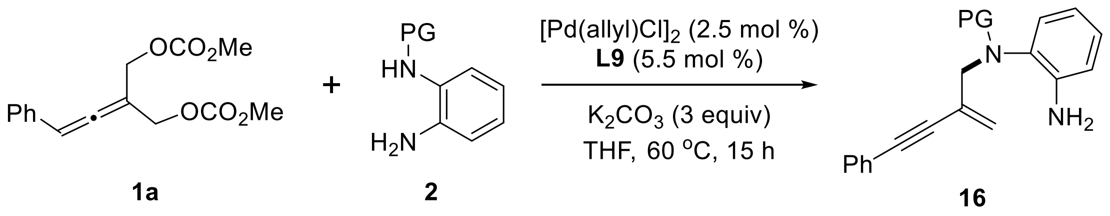

entry	PG	yield of **16** (%)[b]
1	PG3	0
2	PG2	0

In an Argon-filled glovebox, ligand **L9** (4.4 mg, 11 mol %) and $[Pd(allyl)Cl]_2$ (0.9 mg, 2.5 mmol %) and THF (0.8 mL) were added to an oven-dried 10 mL screw-cap tube. After the resulting mixture was stirred at rt for 15 min, **1a** (0.1 mmol) and **2** (1.3 equiv) and K_2CO_3 (3.0 equiv) were added. The screw-cap tube was moved out of the glovebox and at 60 °C for 15 hours. The reaction mixture is condensed under pressure reduction. The residue was purified by silica gel flash column chromatography and no **16** was detected.

7. Single crystal X-ray diffraction data

X-ray crystallographic data for (*R*)-3n

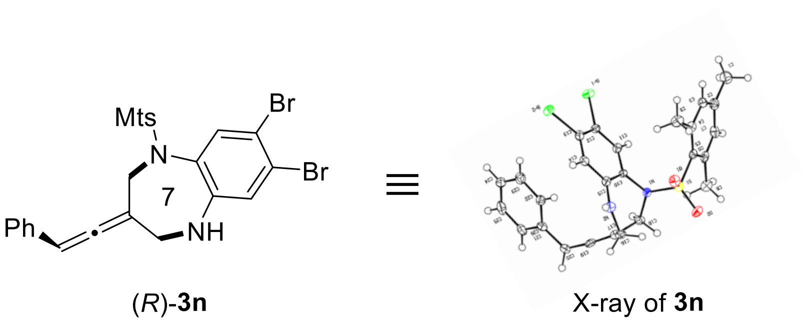

(*R*)-3n. (CCDC NO: 2251580)

Identification code	4
Empirical formula	C26 H24 Br2 N2 O2 S
Formula weight	588.35

Temperature	100.00 K
Wavelength	0.71073 A
Crystal system, space group	Orthorhombic, P2(1)2(1)2(1)
Unit cell dimensions	a = 5.8154(7) A alpha = 90 deg.
	b = 17.400(2) A beta = 90 deg.
	c = 23.776(3) A gamma = 90 deg.
Volume	2405.9(5) A^3
Z, Calculated density	4, 1.624 Mg/m^3
Absorption coefficient	3.483 mm^-1
F(000)	1184
Crystal size	0.2 x 0.18 x 0.16 mm
Theta range for data collection	2.075 to 28.277 deg.
Limiting indices	-7<=h<=7, -22<=k<=23, -31<=l<=31
Reflections collected / unique	5619 / 5619 [R(int) = 0.0477]
Completeness to theta = 25.242	98.9 %
Absorption correction	Semi-empirical from equivalents
Max. and min. transmission	0.745686 and 0.450138
Refinement method	Full-matrix least-squares on F^2
Data / restraints / parameters	5619 / 0 / 301
Goodness-of-fit on F^2	1.039
Final R indices [I>2sigma(I)]	R1 = 0.0472, wR2 = 0.1101
R indices (all data)	R1 = 0.0661, wR2 = 0.1185
Absolute structure parameter	0.044(11)
Extinction coefficient	n/a
Largest diff. peak and hole	0.877 and -0.611 e.A^-3

X-ray crystallographic data for (*S*)-4f

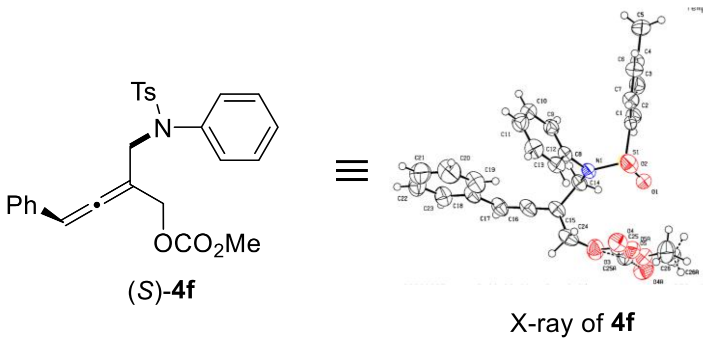

(*S*)-4f. (CCDC NO: 2251582)

Identification code	cu_20221205_Zh_YN_LPF_0m
Empirical formula	$C_{26}H_{25}NO_5S$
Formula weight	463.53
Temperature/K	193.00
Crystal system	orthorhombic
Space group	$P2_12_12_1$
a/Å	6.04550(10)
b/Å	15.2328(3)
c/Å	25.3404(5)
α/°	90
β/°	90
γ/°	90
Volume/Å^3	2333.59(8)
Z	4
ρ_{calc}g/cm^3	1.319
μ/mm^{-1}	1.546
F(000)	976.0
Crystal size/mm^3	0.15 × 0.13 × 0.11
Radiation	CuKα (λ = 1.54178)
2Θ range for data collection/°	6.77 to 136.82
Index ranges	$-7 \leq h \leq 7$, $-18 \leq k \leq 18$, $-30 \leq l \leq 30$
Reflections collected	23877
Independent reflections	4285 [R_{int} = 0.0586, R_{sigma} = 0.0437]
Data/restraints/parameters	4285/0/339
Goodness-of-fit on F^2	1.047
Final R indexes [I>=2σ (I)]	R_1 = 0.0366, wR_2 = 0.0922
Final R indexes [all data]	R_1 = 0.0406, wR_2 = 0.0954
Largest diff. peak/hole / e Å^{-3}	0.25/-0.21
Flack parameter	0.024(8)

X-ray crystallographic data for (*R*)-9a

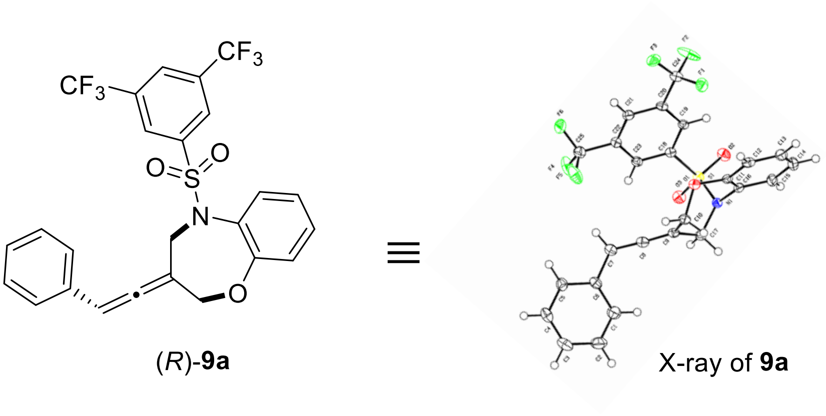

(*R*)-9a. (CCDC NO: 2251583)

Identification code	1
Empirical formula	C25 H17 F6 N O3 S
Formula weight	525.46
Temperature	100.00 K
Wavelength	0.71073 A
Crystal system, space group	Orthorhombic, P2(1)2(1)2(1)
Unit cell dimensions	a = 8.0066(3) A alpha = 90 deg.
	b = 14.4740(6) A beta = 90 deg.
	c = 19.8606(8) A gamma = 90 deg.
Volume	2301.60(16) A^3
Z, Calculated density	4, 1.516 Mg/m^3
Absorption coefficient	0.218 mm^-1
F(000)	1072
Crystal size	0.2 x 0.18 x 0.16 mm
Theta range for data collection	2.051 to 28.298 deg.
Limiting indices	-10<=h<=9, -19<=k<=19, -26<=l<=23
Reflections collected / unique	19572 / 5707 [R(int) = 0.0279]
Completeness to theta = 25.242	100.0 %
Absorption correction	Semi-empirical from equivalents
Max. and min. transmission	0.7457 and 0.6863
Refinement method	Full-matrix least-squares on F^2
Data / restraints / parameters	5707 / 0 / 325
Goodness-of-fit on F^2	1.039

Final R indices [I>2sigma(I)]	R1 = 0.0298, wR2 = 0.0700
R indices (all data)	R1 = 0.0324, wR2 = 0.0717
Absolute structure parameter	0.01(2)
Extinction coefficient	n/a
Largest diff. peak and hole	0.246 and -0.304 e.A^-3

8. References

[1] C. Manzuna Sapu, J.-E. Bäckvall, J. Deska, *Angew. Chem. Int. Ed*. 2011, **50**, 9731.

[2] S. Song, S. Ma, *Chin. J. Chem.* 2020, **38**, 1233.

[3] D. Zhang, Y.-B. Shao, W. Xie, Y. Chen, W. Liu, H. Bao, F. He, X.-S. Xue, X. Yang, *ACS Catal*. 2022, **12**, 14609.

9. Spectral data of products

Spectral data of products 1

dimethyl (2-(2-phenylvinylidene)propane-1,3-diyl) bis(carbonate)

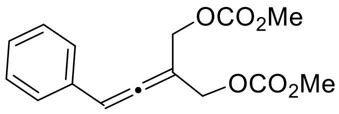

1a

Yellow liquid, 74% yield, flash column chromatography (SiO_2, 10:1 PE/EtOAc);

^{1}H NMR (400 MHz, $CDCl_3$) δ 7.33-7.22 (m, 5H), 6.43-6.42 (m, 1H), 4.84-4.75 (m, 4H), 3.75 (s, 6H);

^{13}C NMR (100 MHz, $CDCl_3$) δ 205.3, 155.4, 132.5, 128.7, 127.8, 127.3, 99.9, 98.2, 65.5, 54.9;

HRMS (ESI) *m/z*: $[M + H]^+$ Calcd for $C_{15}H_{17}O_6^+$ 293.1020; Found 293.1023.

dimethyl (2-(2-(p-tolyl)vinylidene)propane-1,3-diyl) bis(carbonate)

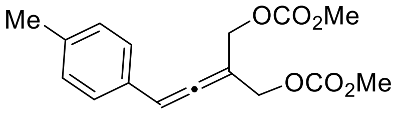

1b

Yellow liquid, 92% yield, flash column chromatography (SiO_2, 10:1 PE/EtOAc);

^{1}H NMR (400 MHz, $CDCl_3$) δ 7.10-7.02 (m, 4H), 6.32 (s, 1H), 4.75-4.66 (m, 4H), 3.67 (s, 6H), 2.24 (s, 3H);

^{13}C NMR (100 MHz, $CDCl_3$) δ 205.2, 155.4, 137.7, 129.5, 129.4, 127.3, 99.7, 98.0, 65.7, 54.9, 21.2;

HRMS (ESI) *m/z*: $[M + H]^+$ Calcd for $C_{16}H_{19}O_6^+$ 307.1176; Found 307.1175.

2-(2-([1,1'-biphenyl]-4-yl)vinylidene)propane-1,3-diyl dimethyl bis(carbonate)

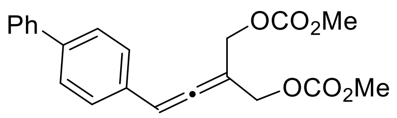

1c

White solid, 76% yield, flash column chromatography (SiO_2, 10:1 PE/EtOAc);

^{1}H NMR (400 MHz, $CDCl_3$) δ 7.60-7.54 (m, 4H), 7.45-7.42 (m, 2H), 7.36-7.34 (m, 3H), 6.48-6.47 (m, 1H), 4.86-4.78 (m, 4H), 3.78 (s, 6H);

^{13}C NMR (100 MHz, $CDCl_3$) δ 205.6, 155.5, 140.7, 140.6, 131.5, 128.8, 127.8, 127.4, 127.0, 100.0, 97.9, 65.6, 55.0;

HRMS (ESI) *m/z*: $[M + H]^+$ Calcd for $C_{21}H_{21}O_6^+$ 369.1333; Found 369.1336.

2-(2-(4-methoxyphenyl)vinylidene)propane-1,3-diyl dimethyl bis(carbonate)

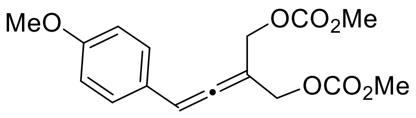

1d

Yellow liquid, 79% yield, flash column chromatography (SiO_2, 10:1 PE/EtOAc);

^{1}H NMR (600 MHz, $CDCl_3$) δ 7.22-7.19 (m, 2H), 6.86-6.84 (m, 2H), 6.40-6.39 (m, 1H), 4.82-4.75 (m, 4H), 3.79 (s, 3H), 3.76 (s, 6H);

^{13}C NMR (150 MHz, $CDCl_3$) δ 205.0, 159.4, 155.5, 128.5, 124.7, 114.2, 99.7, 97.7, 65.8, 55.3, 54.9;

HRMS (ESI) *m/z*: $[M + H]^+$ Calcd for $C_{16}H_{19}O_7^+$ 323.1125; Found 323.1127.

2-(2-(4-chlorophenyl)vinylidene)propane-1,3-diyl dimethyl bis(carbonate)

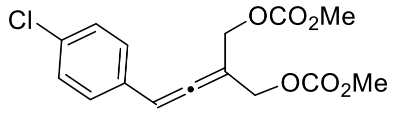

1e

Yellow liquid, 80% yield, flash column chromatography (SiO_2, 10:1 PE/EtOAc);

^{1}H NMR (600 MHz, $CDCl_3$) δ 7.28 (d, J = 8.4 Hz, 2H), 7.21 (d, J = 8.4 Hz, 2H), 6.39 (s, 1H), 4.83-4.76 (m, 4H), 3.76 (s, 6H);

^{13}C NMR (150 MHz, $CDCl_3$) δ 205.2, 155.4, 133.5, 131.1, 128.9, 128.5, 100.4, 97.4, 65.3, 55.0;

HRMS (ESI) *m/z*: $[M + H]^+$ Calcd for $C_{15}H_{16}ClO_6^+$ 327.0630; Found 327.0629.

dimethyl (2-(2-(m-tolyl)vinylidene)propane-1,3-diyl) bis(carbonate)

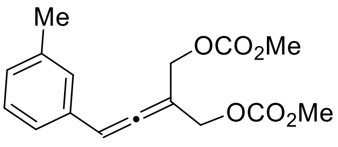

1f

Yellow liquid, 79% yield, flash column chromatography (SiO_2, 10:1 PE/EtOAc);

^{1}H NMR (500 MHz, $CDCl_3$) δ 7.22-7.18 (m, 1H), 7.09-7.04 (m, 3H), 6.40-6.39 (m, 1H), 4.84-4.76 (m, 4H), 3.77 (s, 6H), 2.33 (s, 3H);

^{13}C NMR (125 MHz, $CDCl_3$) δ 205.5, 155.5, 138.3, 132.3, 128.7, 128.6, 128.0, 124.5, 99.7, 98.1, 65.7, 55.0, 21.3;

HRMS (ESI) *m/z*: $[M + H]^+$ Calcd for $C_{16}H_{19}O_6^+$ 307.1176; Found 307.1179.

2-(2-(3-fluorophenyl)vinylidene)propane-1,3-diyl dimethyl bis(carbonate)

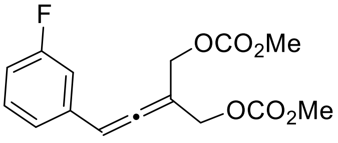

1g

Yellow liquid, 76% yield, flash column chromatography (SiO_2, 10:1 PE/EtOAc);

^{1}H NMR (600 MHz, $CDCl_3$) δ 7.28-7.26 (m, 1H), 7.06-6.92 (m, 3H), 6.41 (s, 1H), 4.80 (dd, J = 20.6, 12.1 Hz, 4H), 3.77 (s, 6H);

^{13}C NMR (150 MHz, $CDCl_3$) δ 205.4, 163.1 (d, J_{C-F} = 246.0 Hz), 155.4, 135.0 (d, J_{C-F} = 7.7 Hz), 130.1 (d, J_{C-F} = 8.2 Hz), 123.1, 114.7 (d, J_{C-F} = 21.3 Hz), 113.9 (d, J_{C-F} = 22.5 Hz), 100.5, 97.5, 65.3, 55.0;

^{19}F NMR (564 MHz, $CDCl_3$) δ -113.1;

HRMS (ESI) *m/z*: $[M + H]^+$ Calcd for $C_{15}H_{16}FO_6^+$ 311.0925; Found 311.0929.

dimethyl (2-(2-(o-tolyl)vinylidene)propane-1,3-diyl) bis(carbonate)

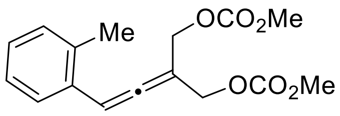

1h

Yellow liquid, 77% yield, flash column chromatography (SiO_2, 10:1 PE/EtOAc);

^{1}H NMR (500 MHz, $CDCl_3$) δ 7.32-7.30 (m, 1H), 7.16-7.14 (m, 3H), 6.63-6.62 (m, 1H), 4.84-4.77 (m, 4H), 3.77 (s, 6H), 2.35 (s, 3H);

^{13}C NMR (125 MHz, $CDCl_3$) δ 206.2, 155.5, 135.6, 130.8, 130.6, 128.0, 127.8, 126.3, 98.7, 95.5, 65.8, 55.0, 19.9;

HRMS (ESI) *m/z*: $[M + H]^+$ Calcd for $C_{16}H_{19}O_6^+$ 307.1176; Found 307.1177.

dimethyl (2-(2-(naphthalen-2-yl)vinylidene)propane-1,3-diyl) bis(carbonate)

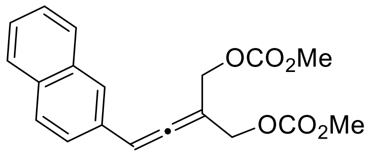

1i

White solid, 79% yield, flash column chromatography (SiO_2, 10:1 PE/EtOAc);

^{1}H NMR (400 MHz, $CDCl_3$) δ 7.78-7.76 (m, 3H), 7.67 (s, 1H), 7.48-7.42 (m, 3H), 6.61 (s, 1H), 4.84 (dd, J = 20.4, 12.3 Hz, 4H), 3.76 (s, 6H);

^{13}C NMR (100 MHz, $CDCl_3$) δ 205.8, 155.5, 133.5, 133.0, 130.0, 128.4, 127.9, 127.8, 126.6, 126.4, 126.1, 124.8, 100.1, 98.6, 65.6, 55.0;

HRMS (ESI) *m/z*: $[M + H]^+$ Calcd for $C_{19}H_{19}O_6^+$ 343.1176; Found 343.1175.

dimethyl (2-(non-1-en-1-ylidene)propane-1,3-diyl) bis(carbonate)

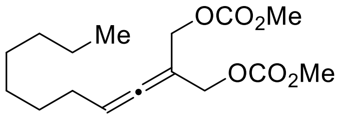

1g

Yellow liquid, 87% yield, flash column chromatography (SiO_2, 20:1 PE/EtOAc);

^{1}H NMR (500 MHz, $CDCl_3$) δ 5.41 (s, 1H), 4.68 (s, 4H), 3.79 (s, 6H), 2.06-2.03 (m, 2H), 1.41-1.28 (m, 10H), 0.89-0.87 (m, 3H);

^{13}C NMR (125 MHz, $CDCl_3$) δ 204.6, 155.5, 95.4, 94.6, 66.3, 54.8, 31.8, 29.0, 28.9, 28.8, 28.1, 22.6, 14.0;

HRMS (ESI) *m/z*: $[M + H]^+$ Calcd for $C_{16}H_{27}O_6^+$ 315.1802; Found 315.1802.

2-(2-cyclohexylvinylidene)propane-1,3-diyl dimethyl bis(carbonate)

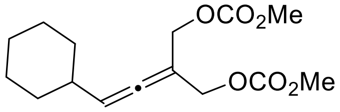

1k

Yellow liquid, 81% yield, flash column chromatography (SiO_2, 20:1 PE/EtOAc);

^{1}H NMR (400 MHz, $CDCl_3$) δ 5.42-5.40 (m, 1H), 4.68 (m, 4H), 3.78 (s, 6H), 2.04-2.02 (m, 1H), 1.76-1.62 (m, 5H), 1.29-1.04 (m, 5H);

^{13}C NMR (100 MHz, $CDCl_3$) δ 203.4, 155.4, 100.4, 96.4, 66.2, 54.6, 36.7, 32.6, 25.8, 25.7;

HRMS (ESI) *m/z*: $[M + H]^+$ Calcd for $C_{15}H_{23}O_6^+$ 299.1489; Found 299.1487.

Spectral data of products 3

(*R*)-1-(mesitylsulfonyl)-3-(2-phenylvinylidene)-2,3,4,5-tetrahydro-1*H*-benzo[*b*][1,4]diazepine (3a)

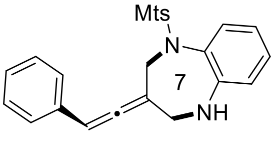

3a, 81%, 95% ee

(Flash column chromatography eluent:petroleum ether/EtOAc = 8:1); white solid, 34.8 mg, 81% yield, 95% ee;

^{1}H NMR (400 MHz, $CDCl_3$) δ (ppm) 7.23-7.21 (m, 2H), 7.18-7.14 (m, 3H), 7.07-7.03 (m, 1H), 6.93 (s, 2H), 6.67-6.65 (m, 1H), 6.51-6.47 (m, 1H), 6.44-6.42 (m, 1H), 6.20 (s, 1H), 4.70 (s, 2H), 4.21-4.08 (m, 3H), 2.46 (s, 6H), 2.32 (s, 3H);

^{13}C NMR (100 MHz, $CDCl_3$) δ (ppm) 201.5, 146.3, 142.4, 140.2, 134.6, 133.9, 131.9, 130.3, 128.9, 128.7, 127.2, 127.0, 125.2, 118.6, 118.6, 102.9, 96.5, 53.1, 47.1, 22.9, 21.0;

HRMS (ESI) exact mass calcd. For $[C_{26}H_{26}N_2O_2SNa]^+$ requires *m/z* 453.1607, found *m/z* 453.1609;

$[\alpha]^{D}_{25}$ = -23.11 (*c* = 0.53, $CHCl_3$);

HPLC (Daicel Chirapak AD-H, hexane/ *i*-PrOH = 90/10, flow rate = 1.0 mL/min, T = 25 °C, 254 nm): t_R = 25.1 min (minor), t_R = 30.4 min (major).

(*R*)-1-(mesitylsulfonyl)-3-(2-(p-tolyl)vinylidene)-2,3,4,5-tetrahydro-1*H*-benzo[*b*][1,4]diazepine (3b)

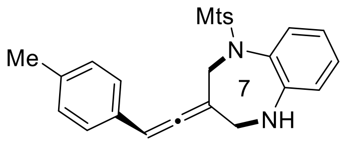

3b, 81%, 94% ee

(Flash column chromatography eluent:petroleum ether/EtOAc = 8:1); white solid, 36.0 mg, 81% yield, 94% ee;

^{1}H NMR (400 MHz, $CDCl_3$) δ (ppm) 7.06-7.03 (m, 5H), 6.93 (s, 2H), 6.67-6.65 (m, 1H), 6.51-6.43 (m, 2H), 6.16 (s, 1H), 4.68 (s, 2H), 4.17-4.06 (m, 3H), 2.46 (s, 6H), 2.31 (s, 3H), 2.29 (s, 3H);

^{13}C NMR (100 MHz, $CDCl_3$) δ (ppm) 201.3, 146.4, 142.4, 140.2, 137.1, 134.6, 131.9, 130.9, 130.3, 129.4, 128.8, 126.9, 125.2, 118.6, 118.5, 102.8, 96.3, 53.2, 47.1, 22.9, 21.2, 21.0;

HRMS (ESI) exact mass calcd. For $[C_{27}H_{28}N_2O_2SNa]^+$ requires *m/z* 467.1764, found *m/z* 467.1765;

$[\alpha]^{D}_{25}$ = -25.14 (*c* = 0.26, $CHCl_3$);

HPLC (Daicel Chirapak OD-H, hexane/ *i*-PrOH = 80/20, flow rate = 1.0 mL/min, T = 25 °C, 254 nm): t_R = 8.5 min (minor), t_R = 12.5 min (major).

(*R*)-3-(2-([1,1'-biphenyl]-4-yl)vinylidene)-1-(mesitylsulfonyl)-2,3,4,5-tetrahydro-1*H*-benzo[*b*][1,4]diazepine (3c)

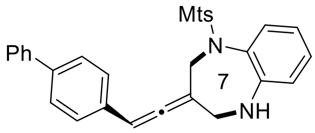

3c, 84%, 93% ee

(Flash column chromatography eluent:petroleum ether/EtOAc = 8:1); white solid, 42.6 mg, 84% yield, 93% ee;

^{1}H NMR (400 MHz, $CDCl_3$) δ (ppm) 7.56-7.54 (m, 2H), 7.47-7.45 (m, 2H), 7.42-7.38 (m, 2H), 7.33-7.29 (m, 1H), 7.23-7.21 (m, 2H), 6.07-7.02 (m, 1H), 6.93(s, 2H), 6.66-6.64 (m, 1H), 6.51-6.42 (m, 2H), 6.23 (s, 1H), 4.71 (s, 2H), 4.22-4.08 (m, 3H), 2.47 (s, 6H), 2.31 (s, 3H);

^{13}C NMR (100 MHz, $CDCl_3$) δ (ppm) 201.7, 146.4, 142.5, 140.7, 140.2, 140.1, 134.6, 133.0, 131.9, 130.3, 128.9, 128.8, 127.5, 127.4, 127.3, 126.9, 125.3, 118.7, 118.6, 103.1, 96.2, 53.2, 47.1, 23.0, 21.0;

HRMS (ESI) exact mass calcd. For $[C_{32}H_{30}N_2O_2SNa]^+$ requires *m/z* 529.1920, found *m/z* 529.1919;

$[\alpha]^{D}_{25}$ = -94.17 (*c* = 1.06, $CHCl_3$);

HPLC (Daicel Chirapak OZ-3, hexane/ *i*-PrOH = 60/40, flow rate = 1.5 mL/min, T = 25 °C, 254 nm): t_R =5.4 min (major), t_R = 11.3 min (minor).

(*R*)-3-(2-(4-chlorophenyl)vinylidene)-1-(mesitylsulfonyl)-2,3,4,5-tetrahydro-1*H*-benzo[*b*][1,4]diazepine (3d)

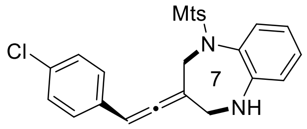

3d, 86%, 92% ee

(Flash column chromatography eluent:petroleum ether/EtOAc = 8:1); white solid, 40.0 mg, 86% yield, 92% ee;

^{1}H NMR (400 MHz, $CDCl_3$) δ (ppm) 7.19-7.17 (m, 2H), 7.09-7.02 (m, 3H), 6.93 (s, 2H), 6.67-6.65 (m, 1H), 6.51-6.47 (m, 1H), 6.40-6.38 (m, 1H), 6.15 (s, 1H), 4.67 (s, 2H), 4.22-4.06 (m, 3H), 2.46 (s, 6H), 2.31 (s, 3H);

^{13}C NMR (100 MHz, $CDCl_3$) δ (ppm) 201.5, 146.4, 142.5, 140.2, 134.5, 132.8, 132.5, 131.9, 130.2, 128.9, 128.8, 128.2, 125.4, 118.8, 103.5, 95.6, 53.0, 47.1, 22.9, 21.0;
HRMS (ESI) exact mass calcd. For $[C_{26}H_{25}ClN_2O_2SNa]^+$ requires *m/z* 487.1217, found *m/z* 487.1219;
$[\alpha]^{D}_{25}$ = -73.51 (*c* = 0.87, $CHCl_3$);
HPLC (Daicel Chirapak OZ-3, hexane/ *i*-PrOH = 70/30, flow rate = 1.0 mL/min, T = 25 °C, 254 nm): t_R = 6.7 min (major), t_R = 7.6 min (minor).

(*R*)-1-(mesitylsulfonyl)-3-(2-(m-tolyl)vinylidene)-2,3,4,5-tetrahydro-1*H*-benzo[*b*][1,4]diazepine (3e)

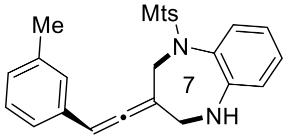

3e, 84%, 93% ee

(Flash column chromatography eluent:petroleum ether/EtOAc = 8:1); white solid, 37.4 mg, 84% yield, 93% ee;
^{1}H NMR (400 MHz, $CDCl_3$) δ (ppm) 7.13-7.09 (m, 1H), 7.06-7.02 (m, 1H), 6.98-6.93 (m, 5H), 6.66-6.63 (m, 1H), 6.50-6.43 (m, 2H), 6.16 (s, 1H), 4.75-4.64 (m, 2H), 4.16-4.08 (m, 3H), 2.46 (s, 6H), 2.31 (s, 3H), 2.24 (s, 3H);
^{13}C NMR (100 MHz, $CDCl_3$) δ (ppm) 201.7, 146.4, 142.4, 140.2, 138.3, 134.6, 133.7, 131.9, 130.4, 129.0, 128.6, 128.1, 127.5, 125.0, 124.2, 118.6, 118.5, 102.7, 96.5, 53.3, 47.0, 22.9, 21.3, 21.0;
HRMS (ESI) exact mass calcd. For $[C_{27}H_{28}N_2O_2SNa]^+$ requires *m/z* 467.1764, found *m/z* 467.1766;
$[\alpha]^{D}_{25}$ = -38.47 (*c* = 0.83, $CHCl_3$);
HPLC (Daicel Chirapak OZ-3, hexane/ *i*-PrOH = 70/30, flow rate = 1.0 mL/min, T = 25 °C, 254 nm): t_R = 10.8 min (minor), t_R = 16.6 min (major).

(*R*)-3-(2-(3-fluorophenyl)vinylidene)-1-(mesitylsulfonyl)-2,3,4,5-tetrahydro-1*H*-benzo[*b*][1,4]diazepine (3f)

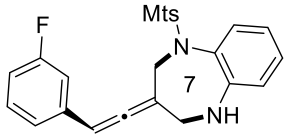

3f, 83%, 93% ee

(Flash column chromatography eluent:petroleum ether/EtOAc = 8:1); white solid, 37.3 mg, 83% yield, 93% ee;

^{1}H NMR (400 MHz, $CDCl_3$) δ (ppm) 7.20-7.18 (m, 1H), 7.07-7.03 (m, 1H), 6.97-6.94 (m, 3H), 6.87-6.84 (m, 2H), 6.68-6.66 (m, 1H), 6.52-6.48 (m, 1H), 6.43-6.40 (m, 1H), 6.17 (s, 1H), 4.69 (s, 2H), 4.21-4.09 (m, 3H), 2.46 (s, 6H), 2.32 (s, 3H);

^{13}C NMR (100 MHz, $CDCl_3$) δ (ppm) 201.5, 163.1 (d, $J_{C\text{-}F}$ = 245.6 Hz), 146.2, 142.5, 140.2, 136.5, 136.4 (d, $J_{C\text{-}F}$ = 7.8 Hz), 131.9, 130.2, 130.0 (d, $J_{C\text{-}F}$ = 8.4 Hz), 128.9, 125.3, 122.7 (d, $J_{C\text{-}F}$ = 2.7 Hz), 118.8, 118.7, 114.1 (d, $J_{C\text{-}F}$ = 21.5 Hz), 113.5 (d, $J_{C\text{-}F}$ = 22.2 Hz), 103.5, 95.9, 95.8, 52.9, 47.0, 22.9, 21.0;

^{19}F NMR (376 MHz, $CDCl_3$) δ -113.3;

HRMS (ESI) exact mass calcd. For $[C_{26}H_{25}FN_2O_2SNa]^+$ requires *m/z* 471.1513, found *m/z* 471.1514;

$[\alpha]^{D}_{25}$ = -16.37 (*c* = 0.39, $CHCl_3$);

HPLC (Daicel Chirapak OZ-3, hexane/ *i*-PrOH = 65/35, flow rate = 1.0 mL/min, T = 25 °C, 254 nm): t_R = 7.5 min (minor), t_R = 9.7 min (major).

(*R*)-1-(mesitylsulfonyl)-3-(2-(o-tolyl)vinylidene)-2,3,4,5-tetrahydro-1*H*-benzo[*b*][1,4]diazepine (3g)

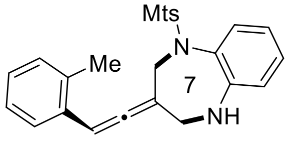

3g, 89%, 91% ee

(Flash column chromatography eluent:petroleum ether/EtOAc = 8:1); white solid, 39.6 mg, 89% yield, 91% ee;

^{1}H NMR (400 MHz, $CDCl_3$) δ (ppm) 7.16-7.13 (m, 1H), 7.10-7.01 (m, 4H), 6.93 (s, 2H), 6.64-6.62 (m, 1H), 6.50-6.43 (m, 2H), 6.38 (s, 1H), 4.76-4.63 (m, 2H), 4.18-4.13 (m, 3H), 2.46 (s, 6H), 2.31 (s, 3H), 2.28 (s, 3H);

^{13}C NMR (100 MHz, $CDCl_3$) *δ* (ppm) 202.2, 146.3, 142.4, 140.2, 135.3, 134.6, 132.0, 131.9, 130.6, 130.3, 128.9, 127.7, 127.2, 126.2, 125.1, 118.6, 118.5, 101.7, 93.9, 53.2, 47.1, 23.0, 21.0, 19.9;

HRMS (ESI) exact mass calcd. For $[C_{27}H_{28}N_2O_2SNa]^+$ requires *m/z* 467.1764, found *m/z* 467.1762;

$[\alpha]^{D}_{25}$ = -35.67 (*c* = 0.67, $CHCl_3$);

HPLC (Daicel Chirapak OD-H, hexane/ *i*-PrOH = 70/30, flow rate = 1.0 mL/min, T = 25 °C, 254 nm): t_R =11.9 min (major), t_R = 15.2 min (minor).

(*R*)-1-(mesitylsulfonyl)-3-(2-(naphthalen-2-yl)vinylidene)-2,3,4,5-tetrahydro-1*H*-benzo[*b*][1,4]diazepine (3h)

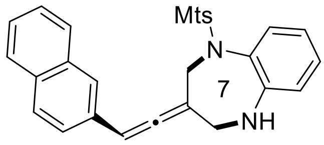

3h, 87%, 94% ee

(Flash column chromatography eluent:petroleum ether/EtOAc = 8:1); white solid, 41.8 mg, 87% yield, 94% ee;

^{1}H NMR (400 MHz, $CDCl_3$) *δ* (ppm) 7.75-7.65 (m, 3H), 7.57 (s, 1H), 7.44-7.38 (m, 2H), 7.29-7.26 (m, 1H), 7.10-7.06 (m, 1H), 6.93 (s, 2H), 6.67 (d, *J* = 8.1 Hz, 1H), 6.53-6.49 (m, 1H), 6.45-6.42 (m, 1H), 6.36 (s, 1H), 4.74 (s, 2H), 4.24-4.10 (m, 3H), 2.47 (s, 6H), 2.31 (s, 3H);

^{13}C NMR (100 MHz, $CDCl_3$) *δ* (ppm) 202.1, 146.4, 142.5, 140.2, 134.6, 133.6, 132.8, 131.9, 131.4, 130.3, 129.0, 128.3, 127.8, 127.7, 126.2, 125.9, 125.8, 125.1, 124.8, 118.7, 118.6, 103.2, 96.9, 53.2, 47.1, 23.0, 21.0;

HRMS (ESI) exact mass calcd. For $[C_{30}H_{28}N_2O_2SNa]^+$ requires *m/z* 503.1764, found *m/z* 503.1765;

$[\alpha]^{D}_{25}$ = -62.22 (*c* = 0.89, $CHCl_3$);

HPLC (Daicel Chirapak AD-H, hexane/ *i*-PrOH = 70/30, flow rate = 1.0 mL/min, T = 25 °C, 254 nm): t_R =9.6 min (minor), t_R = 11.9 min (major).

(*R*)-3-(2-cyclohexylvinylidene)-1-(mesitylsulfonyl)-2,3,4,5-tetrahydro-1*H*-benzo[*b*][1,4]diazepine (3i)

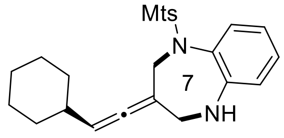

3i, 85%, 83% ee

(Flash column chromatography eluent:petroleum ether/EtOAc = 15:1); colorless oily liquid, 37.1 mg, 85% yield, 83% ee;

^{1}H NMR (500 MHz, $CDCl_3$) δ (ppm) 7.01-6.97 (m, 1H), 6.91 (s, 2H), 6.61-6.59 (m, 1H), 6.49-6.43 (m, 2H), 5.17-5.16 (m, 1H), 4.54 (s, 2H), 4.11-3.89 (m, 3H), 2.44 (s, 6H), 2.30 (s, 3H), 1.95-1.91 (m, 1H), 1.65-1.56 (m, 5H), 1.25-0.94 (m, 5H);

^{13}C NMR (125 MHz, $CDCl_3$) δ (ppm) 199.4, 146.5, 142.2, 140.2, 134.5, 131.8, 130.4, 128.7, 125.1, 118.6, 118.3, 99.5, 99.4, 53.7, 47.4, 37.2, 32.9, 32.8, 26.1, 25.9, 22.9, 21.0;

HRMS (ESI) exact mass calcd. For $[C_{26}H_{32}N_2O_2SNa]^+$ requires *m/z* 459.2077, found *m/z* 459.2079;

$[\alpha]^{D}_{25}$ = -51.36 (*c* = 1.02, $CHCl_3$);

HPLC (Daicel Chirapak OZ-3, hexane/ *i*-PrOH = 80/20, flow rate = 1.0 mL/min, T = 25 °C, 254 nm): t_R = 6.0 min (minor), t_R = 6.6 min (major).

(*R*)-1-(mesitylsulfonyl)-3-(non-1-en-1-ylidene)-2,3,4,5-tetrahydro-1*H*-benzo[*b*][1,4]diazepine (3j)

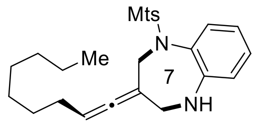

3j, 86%, 72% ee

(Flash column chromatography eluent:petroleum ether/EtOAc = 20:1); colorless oily liquid, 39.0 mg, 86% yield, 72% ee;

^{1}H NMR (400 MHz, $CDCl_3$) δ (ppm) 7.01-6.97 (m, 1H), 6.91 (s, 2H), 6.616.59 (m, 1H), 6.51-6.43 (m, 2H), 5.19-5.16 (m, 1H), 4.54 (s, 2H), 4.10-3.89 (m, 3H), 2.45 (s, 6H), 2.30 (s, 3H), 1.94 (q, *J* = 13.9, 6.8 Hz, 2H), 1.30-1.22 (m, 10H), 0.87 (t, *J* = 6.9 Hz, 3H);

^{13}C NMR (100 MHz, $CDCl_3$) δ (ppm) 200.1, 146.4, 142.2, 140.2, 134.6, 131.8, 130.4, 128.7, 125.2, 118.5, 118.3, 98.6, 93.4, 53.5, 47.4, 31.9, 29.1, 29.0, 28.9, 28.6, 22.9, 22.7, 21.0, 14.1;

HRMS (ESI) exact mass calcd. For $[C_{27}H_{36}N_2O_2SNa]^+$ requires *m/z* 475.2390, found *m/z* 475.2387;

$[\alpha]_{25}^{D}$ = -40.92 (*c* = 1.09, $CHCl_3$);

HPLC (Daicel Chirapak OZ-3, hexane/ *i*-PrOH = 85:15, flow rate = 1.0 mL/min, T = 25 °C, 254 nm): t_R = 6.3 min (minor), t_R = 7.0 min (major).

(*R*)-1-(mesitylsulfonyl)-7,8-dimethyl-3-(2-phenylvinylidene)-2,3,4,5-tetrahydro-1*H*-benzo[*b*][1,4]diazepine (3k)

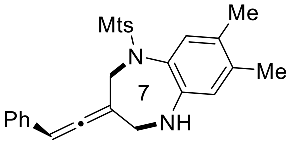

3k, 80%, 90% ee

(Flash column chromatography eluent:petroleum ether/EtOAc = 8:1); white solid, 36.7 mg, 80% yield, 90% ee;

^{1}H NMR (500 MHz, $CDCl_3$) δ (ppm) 7.25-7.21 (m, 2H), 7.17-7.15 (m, 3H), 6.94 (s, 2H), 6.48 (s, 1H), 6.22 (s, 1H), 6.16 (s, 1H), 4.63-4.54 (m, 2H), 4.16-3.95 (m, 3H), 2.47 (s, 6H), 2.32 (s, 3H), 2.13 (s, 3H), 1.90 (s, 3H);

^{13}C NMR (100 MHz, $CDCl_3$) δ (ppm) 201.5, 143.8, 142.3, 140.3, 137.2, 134.6, 134.0, 131.8, 130.8, 128.6, 127.3, 127.2, 127.1, 123.7, 120.3, 103.1, 96.1, 52.9, 47.5, 23.0, 21.0, 19.4, 18.4;

HRMS (ESI) exact mass calcd. For $[C_{28}H_{30}N_2O_2SNa]^+$ requires *m/z* 481.1920, found *m/z* 481.1920;

$[\alpha]_{25}^{D}$ = -62.99 (*c* = 1.34, $CHCl_3$);

HPLC (Daicel Chirapak OZ-3, hexane/ *i*-PrOH = 70/30, flow rate = 1.0 mL/min, T = 25 °C, 254 nm): t_R = 10.1 min (minor), t_R = 15.2 min (major).

(*R*)-7,8-difluoro-1-(mesitylsulfonyl)-3-(2-phenylvinylidene)-2,3,4,5-tetrahydro-1*H*-benzo[*b*][1,4]diazepine (3l)

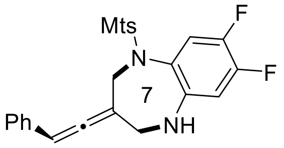

3l, 80%, 92% ee

(Flash column chromatography eluent:petroleum ether/EtOAc = 8:1); white solid, 37.4 mg, 80% yield, 92% ee;

^{1}H NMR (500 MHz, $CDCl_3$) δ (ppm) 7.27-7.24 (m, 2H), 7.20-7.15 (m, 3H), 6.97 (s, 2H), 6.45 (dd, J = 11.7, 7.6 Hz, 1H), 6.29-6.25 (m, 1H), 6.21 (s, 1H), 4.64 (s, 2H), 4.14-4.03 (m, 3H), 2.48 (s, 6H), 2.33 (s, 3H);
^{13}C NMR (100 MHz, $CDCl_3$) δ (ppm) 201.6, 150.1 (dd, $J_{C\text{-}F}$ = 248.4, 13.7 Hz), 143.8, 143.5 (d, $J_{C\text{-}F}$ = 8.4 Hz), 143.0, 140.2, 133.7, 133.6, 132.1, 128.7, 127.4, 127.0, 118.7 (dd, $J_{C\text{-}F}$ = 18.3, 2.2 Hz), 106.3 (d, $J_{C\text{-}F}$ = 20.4 Hz), 102.3, 96.8, 53.1, 46.9, 22.9, 21.0;
^{19}F NMR (376 MHz, $CDCl_3$) δ -136.6, -149.3;
HRMS (ESI) exact mass calcd. For $[C_{26}H_{24}F_2N_2O_2SNa]^+$ requires *m/z* 489.1419, found *m/z* 489.1420;
$[\alpha]^{D}_{25}$ = -29.03 (c = 0.73, $CHCl_3$);
HPLC (Daicel Chirapak OZ-3, hexane/ *i*-PrOH = 70/30, flow rate = 1.0 mL/min, T = 25 °C, 254 nm): t_R = 5.6 min (minor), t_R = 6.9 min (major).

(*R*)-7,8-dichloro-1-(mesitylsulfonyl)-3-(2-phenylvinylidene)-2,3,4,5-tetrahydro-1*H*-benzo[*b*][1,4]diazepine (3m)

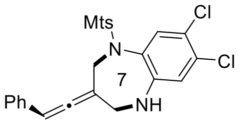

3m, 85%, 93% ee

(Flash column chromatography eluent:petroleum ether/EtOAc = 8:1); white solid, 42.4 mg, 85% yield, 93% ee;
^{1}H NMR (500 MHz, $CDCl_3$) δ (ppm) 7.27-7.14 (m, 5H), 6.99 (s, 2H), 6.74 (s, 1H), 6.47 (s, 1H), 6.22 (s, 1H), 4.65 (s, 2H), 4.24-4.06 (m, 3H), 2.49 (s, 6H), 2.34 (s, 3H);
^{13}C NMR (150 MHz, $CDCl_3$) δ (ppm) 201.6, 145.7, 143.1, 140.2, 133.7, 133.5, 132.2, 132.1, 131.5, 128.7, 128.6, 127.5, 127.0, 126.4, 124.4, 120.3, 118.9, 102.0, 97.0, 53.0, 46.6, 23.0, 21.0;
HRMS (ESI) exact mass calcd. For $[C_{26}H_{24}Cl_2N_2O_2SNa]^+$ requires *m/z* 521.0828, found *m/z* 521.0829;
$[\alpha]^{D}_{25}$ = -34.21 (c = 0.67, $CHCl_3$);
HPLC (Daicel Chirapak OZ-3, hexane/ *i*-PrOH = 70:30, flow rate = 1.0 mL/min, T = 25 °C, 254 nm): t_R = 4.7 min (minor), t_R = 5.9 min (major).

(*R*)-7,8-dibromo-1-(mesitylsulfonyl)-3-(2-phenylvinylidene)-2,3,4,5-tetrahydro-1*H*-benzo[*b*][1,4]diazepine (3n)

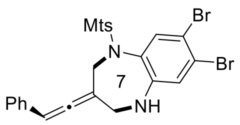

3n, 85%, 93% ee

(Flash column chromatography eluent:petroleum ether/EtOAc = 8:1); white solid, 50.0 mg, 85% yield, 93% ee;

^{1}H NMR (600 MHz, $CDCl_3$) δ (ppm) 7.25-7.24 (m, 2H), 7.20-7.17 (m, 1H), 7.14-7.13 (m, 2H), 6.98 (s, 2H), 6.90 (s, 1H), 6.56 (s, 1H), 6.22 (s, 1H), 4.64 (s, 2H), 4.26 (s, 1H), 4.17-4.05 (m, 2H), 2.48 (s, 6H), 2.33 (s, 3H);

^{13}C NMR (150 MHz, $CDCl_3$) δ (ppm) 201.6, 146.3, 143.1, 140.3, 134.6, 133.6, 133.5, 132.1, 128.7, 127.5, 127.0, 125.2, 124.3, 122.1, 110.9, 102.0, 97.0, 53.0, 46.6, 23.0, 21.0;

HRMS (ESI) exact mass calcd. For $[C_{26}H_{24}Br_2N_2O_2SNa]^+$ requires *m/z* 608.9817, found *m/z* 608.9815;

$[\alpha]_{25}^{D}$ = -84.10 (*c* = 1.33, $CHCl_3$);

HPLC (Daicel Chirapak OZ-3, hexane/ *i*-PrOH = 70/30, flow rate = 1.0 mL/min, T = 25 °C, 254 nm): t_R = 5.0 min (minor), t_R = 6.6 min (major). Absolute configuration is confirmed by X-ray.

Melting range:176.4-177.3 ^{o}C.

(*R*)-1-(mesitylsulfonyl)-3-(2-phenylvinylidene)-2,3,4,5-tetrahydro-1*H*-naphtho[2,3-*b*][1,4]diazepine (3o)

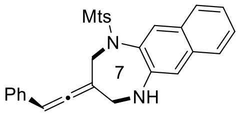

3o, 71%, 93% ee

(Flash column chromatography eluent:petroleum ether/EtOAc = 8:1); white solid, 34.1 mg, 71% yield, 93% ee;

^{1}H NMR (500 MHz, $CDCl_3$) δ (ppm) 7.56 (d, *J* = 8.3 Hz, 1H), 7.37-7.34 (m, 2H), 7.19-7.16 (m, 1H), 7.09-7.01 (m, 7H), 6.95 (s, 2H), 6.17 (s, 1H), 4.70-4.61 (m, 2H), 4.30 (s, 1H), 4.15-4.05 (m, 2H), 2.47 (s, 6H), 2.33 (s, 3H);

^{13}C NMR (150 MHz, $CDCl_3$) δ (ppm) 202.0, 144.5, 142.6, 140.3, 134.3, 133.9, 133.7, 132.0, 129.2, 128.6, 128.6, 128.5, 128.1, 127.6, 127.2, 127.0, 126.9, 125.5, 123.2, 113.8, 102.5, 96.3, 53.0, 47.9, 23.0, 21.0;

HRMS (ESI) exact mass calcd. For $[C_{30}H_{28}N_2O_2SNa]^+$ requires *m/z* 503.1764, found *m/z* 503.1763;

$[\alpha]^D_{25}$ = -48.37 (*c* = 0.89, $CHCl_3$);

HPLC (Daicel Chirapak OZ-3, hexane/ *i*-PrOH = 70/30, flow rate = 1.0 mL/min, T = 25 °C, 254 nm): t_R = 7.5 min (minor), t_R = 9.7 min (major).

Spectral data of products 4

(*S*)-2-(((*N*-(2-aminophenyl)-2,4,6-trimethylphenyl)sulfonamido)methyl)-4-phenyl buta-2,3-dien-1-yl methyl carbonate (4a)

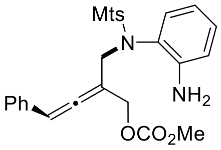

4a, 75%, 90% ee

(Flash column chromatography eluent:petroleum ether/EtOAc = 8:1); yellow oily liquid, 30.3 mg, 75% yield, 90% ee;

^{1}H NMR (400 MHz, $CDCl_3$) δ (ppm) 7.18-7.07 (m, 4H), 6.91-6.87 (m, 3H), 6.79-6.57 (m, 3H), 6.50-6.39 (m, 1H), 6.07 (m, 1H), 5.11-4.68 (m, 3H), 4.35-4.06 (m, 3H), 3.77-3.73 (m, 3H), 2.40-2.36 (m, 6H), 2.27 (s, 3H);

^{13}C NMR (100 MHz, $CDCl_3$) δ (ppm) 206.2, 205.9, 155.5, 155.4, 146.9, 146.5, 142.6, 142.6, 140.3, 132.8, 132.6, 132.5, 132.3, 132.1, 131.2, 129.9, 129.6, 129.5, 128.5, 128.4, 127.4, 127.3, 127.2, 123.4, 123.1, 118.8, 118.5, 117.3, 117.2, 99.8, 99.7, 97.0, 65.9, 65.7, 55.0, 51.5, 49.4, 23.5, 23.4, 21.0;

HRMS (ESI) exact mass calcd. For $[C_{28}H_{30}N_2O_5SNa]^+$ requires *m/z* 529.1768, found *m/z* 529.1767;

$[\alpha]^D_{25}$ = -127.8 (*c* = 1.31, $CHCl_3$);

HPLC (Daicel Chirapak OD-H, hexane/ *i*-PrOH = 70/30, flow rate = 1.0 mL/min, T = 25 °C, 254 nm): t_R = 7.2 min (major), t_R = 8.9 min (minor).

(*S*)-2-(((*N*-(2-amino-4,5-dimethylphenyl)-2,4,6-trimethylphenyl)sulfonamido)methyl)-4-phenylbuta-2,3-dien-1-yl methyl carbonate (4b)

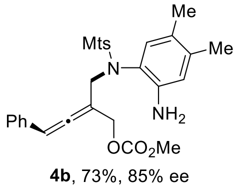

4b, 73%, 85% ee

(Flash column chromatography eluent:petroleum ether/EtOAc = 8:1); yellow oily liquid, 39.1 mg, 73% yield, 85% ee;

^{1}H NMR (600 MHz, $CDCl_3$) δ (ppm) 7.14-7.05 (m, 3H), 6.88-6.86 (m, 3H), 6.71-6.70 (m, 1H), 6.57-6.51 (m, 1H), 6.44-6.19 (m, 1H), 6.12-6.06 (m, 1H), 5.05-4.68 (m, 3H), 4.58-4.33 (m, 1H), 4.05-4.03 (m, 2H), 3.76-3.71 (m, 3H), 2.43-2.36 (m, 6H), 2.26 (s, 3H), 2.15-2.13 (m, 3H), 2.01-1.67 (m, 3H);

^{13}C NMR (150 MHz, $CDCl_3$) δ (ppm) 206.0, 205.9, 155.5, 155.4, 144.3, 143.7, 142.5, 142.4, 140.3, 138.0, 137.8, 133.0, 132.8, 132.5, 131.9, 130.3, 128.4, 128.2, 127.3, 127.2, 126.9, 126.6, 121.0, 118.8, 118.6, 100.0, 97.1, 65.9, 65.7, 54.9, 51.0, 49.0, 23.5, 23.4, 20.9, 19.6, 18.5, 18.2;

HRMS (ESI) exact mass calcd. For $[C_{30}H_{34}N_2O_5SNa]^+$ requires *m/z* 557.2081, found *m/z* 557.2083;

$[\alpha]^{D}_{25}$ = -37.20 (*c* = 0.97, $CHCl_3$);

HPLC (Daicel Chirapak OD-H, hexane/ *i*-PrOH = 70/30, flow rate = 1.0 mL/min, T = 25 °C, 254 nm): t_R = 9.5 min (major), t_R = 13.5 min (minor).

(*S*)-2-(((*N*-(2-amino-4,5-difluorophenyl)-2,4,6-trimethylphenyl)sulfonamido)methyl)-4-phenylbuta-2,3-dien-1-yl methyl carbonate (4c)

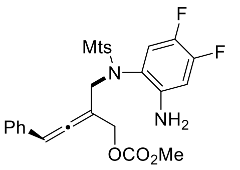

4c, 73%, 90% ee

(Flash column chromatography eluent:petroleum ether/EtOAc = 8:1); yellow oily liquid, 39.6 mg, 73% yield, 90% ee;

^{1}H NMR (500 MHz, $CDCl_3$) δ (ppm) 7.26-7.16 (m, 3H), 6.97-6.90 (m, 3H), 6.80 (s, 1H), 6.62-6.53 (m, 1H), 6.42-6.31 (m, 1H), 6.16-6.10 (m, 1H), 5.01-4.60 (m, 3H), 4.28-3.98 (m, 3H), 3.77-3.73 (m, 3H),2.43-2.40 (m, 6H), 2.28 (s, 3H);

^{13}C NMR (125 MHz, $CDCl_3$) δ (ppm) 206.1, 206.0, 155.5, 151.8, 151.7, 149.8, 149.7, 144.2, 143.7, 143.1, 140.2, 132.4, 132.2, 131.8, 131.7, 128.5, 127.7, 127.1, 127.0, 119.6, 119.5, 118.4, 104.8, 104.6, 99.4, 97.4, 65.8, 65.6, 54.9, 51.3, 49.5, 23.4, 23.4, 20.9;

^{19}F NMR (470 MHz, $CDCl_3$) δ -135.0, -135.1, -135.2, -149.2, -149.3, -149.5, -149.6;

HRMS (ESI) exact mass calcd. For $[C_{28}H_{28}F_2N_2O_5SNa]^+$ requires *m/z* 565.1579, found *m/z* 565.1577;

$[\alpha]_{25}^{D}$ = -57.68 (c = 0.78, $CHCl_3$);

HPLC (Daicel Chirapak OD-H, hexane/ *i*-PrOH = 80/20, flow rate = 1.0 mL/min, T = 25 °C, 254 nm): t_R = 12.3 min (major), t_R = 16.0 min (minor).

(*S*)-2-(((*N*-(2-aminophenyl)-2,4,6-trimethylphenyl)sulfonamido)methyl)-4-(p-tolyl)buta-2,3-dien-1-yl methyl carbonate (4d)

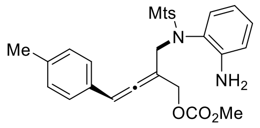

4d, 79%, 90% ee

(Flash column chromatography eluent:petroleum ether/EtOAc = 8:1); yellow oily liquid, 41.1 mg, 79% yield, 90% ee;

^{1}H NMR (500 MHz, $CDCl_3$) δ (ppm) 7.08 (t, J = 7.6 Hz, 1H), 6.98 (d, J = 7.6 Hz, 1H), 6.91 (d, J = 7.7 Hz, 1H), 6.86 (s, 2H), 6.81-6.58 (m, 4H), 6.51-6.42 (m, 1H), 6.09-6.00 (m, 1H), 5.06-4.66 (m, 3H), 4.33-4.06 (m, 3H), 3.76-3.72 (m, 3H), 2.40-2.36 (m, 6H), 2.28-2.26 (m, 6H);

^{13}C NMR (125 MHz, $CDCl_3$) δ (ppm) 206.1, 205.8, 155.5, 155.4, 146.9, 146.5, 142.5, 140.3, 137.3, 137.1, 132.6, 132.5, 132.0, 131.2, 130.1, 129.8, 129.6, 129.5, 129.5, 129.2, 129.1, 127.1, 123.5, 123.2, 118.7, 118.5, 117.3, 117.1, 99.6, 99.5, 96.9, 66.0, 65.8, 54.9, 51.5, 49.5, 23.5, 23.3, 21.2, 20.9;

HRMS (ESI) exact mass calcd. For $[C_{29}H_{32}N_2O_5SNa]^+$ requires *m/z* 543.1924, found *m/z* 543.1924;

$[\alpha]_{25}^{D}$ = -64.84 (c = 0.86, $CHCl_3$);

HPLC (Daicel Chirapak AS-H, hexane/ *i*-PrOH = 80/20, flow rate = 1.0 mL/min, T = 25 °C, 254 nm): t_R = 10.6 min (minor), t_R = 16.1 min (major).

(*S*)-2-(((*N*-(2-aminophenyl)-2,4,6-trimethylphenyl)sulfonamido)methyl)-4-cyclohexylbuta-2,3-dien-1-yl methyl carbonate (4e)

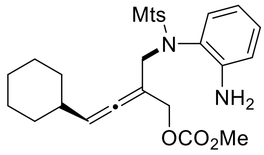

4e, 73%, 88% ee

(Flash column chromatography eluent:petroleum ether/EtOAc = 8:1); yellow oily liquid, 37.4 mg, 73% yield, 88% ee;

^{1}H NMR (600 MHz, $CDCl_3$) δ (ppm) 7.03-7.01 (m, 1H), 6.87 (s, 2H), 6.68-6.49 (m, 3H), 5.05-4.48 (m, 4H), 4.23-3.90 (m, 3H), 3.79-3.77 (m, 3H), 2.41-2.35 (m, 6H), 2.27 (s, 3H), 1.67-0.60 (m, 11H);

^{13}C NMR (150 MHz, $CDCl_3$) δ (ppm) 204.3, 155.6, 147.1, 146.6, 142.4, 140.2, 132.9, 132.7, 131.9, 131.6, 130.1, 129.3, 123.6, 123.2, 118.2, 116.9, 99.4, 96.0, 66.6, 54.7, 52.1, 49.8, 36.9, 32.7, 32.5, 32.3, 25.8, 23.4, 23.3, 20.9;

HRMS (ESI) exact mass calcd. For $[C_{28}H_{36}N_2O_5SNa]^+$ requires *m/z* 535.2237, found *m/z* 535.2237;

$[\alpha]^{D}_{25}$ = -29.44 (*c* = 0.84, $CHCl_3$);

HPLC (Daicel Chirapak AD-H, hexane/ *i*-PrOH = 95/5, flow rate = 1.0 mL/min, T = 25 °C, 254 nm): t_R = 36.7 min (minor), t_R = 47.7 min (major).

(*S*)-methyl(2-(((4-methyl-N-phenylphenyl)sulfonamido)methyl)-4-phenylbuta-2,3-dien-1-yl) carbonate (4f)

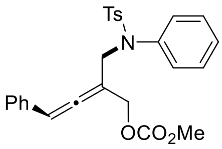

4f, 75%, 90% ee

(Flash column chromatography eluent:petroleum ether/EtOAc = 8:1); white solid, 34.8 mg, 75% yield, 90% ee;

^{1}H NMR (500 MHz, $CDCl_3$) δ (ppm) 7.46 (d, *J* = 8.3 Hz, 2H), 7.30-7.21 (m, 5H), 7.13-7.05 (m, 5H), 6.76-6.74 (m, 2H), 6.12-6.11 (m, 1H), 4.79-4.78 (m, 2H), 4.51-4.48 (m, 1H), 4.21-4.18 (m, 1H), 3.73 (s, 3H), 2.40 (s, 3H);

^{13}C NMR (125 MHz, $CDCl_3$) δ (ppm) 205.4, 155.4, 143.6, 138.6, 134.9, 132.5, 129.5, 129.1, 128.8, 128.4, 128.0, 127.8, 127.4, 127.3, 127.1, 99.6, 97.6, 65.4, 54.9, 50.8, 21.5;

HRMS (ESI) exact mass calcd. For $[C_{26}H_{25}NO_5SNa]^+$ requires *m/z* 486.1346, found *m/z* 486.1345;

$[\alpha]^{D}_{25}$ = -19.25 (*c* = 0.86, $CHCl_3$);

HPLC (Daicel Chirapak OD-H, hexane/ *i*-PrOH = 75/25, flow rate = 1.0 mL/min, T = 25 °C, 254 nm): t_R = 9.1 min (major), t_R = 10.6 min (minor). Absolute configuration is confirmed by X-ray.

Melting range:79.6-80.3 ^{o}C.

Spectral data of products 9

(*R*)-5-((3,5-bis(trifluoromethyl)phenyl)sulfonyl)-3-(2-phenylvinylidene)-2,3,4,5-tetrahydrobenzo[*b*][1,4]oxazepine (9a)

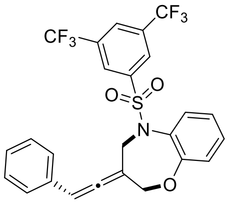

9a, 87%, 90% ee

(Flash column chromatography eluent:petroleum ether/EtOAc = 12:1); white solid, 45.7 mg, 87% yield, 90% ee;

^{1}H NMR (400 MHz, $CDCl_3$) δ (ppm) 8.04 (s, 1H), 7.97 (s, 2H), 7.62-7.59 (m, 1H), 7.39-7.35 (m, 1H), 7.28-7.20 (m, 4H), 7.01-6.95 (m, 3H), 6.31 (s, 1H), 4.84 (d, J = 14.4 Hz, 1H), 4.35 (d, J = 12.3 Hz, 1H), 4.22-4.14 (m, 2H);

^{13}C NMR (100 MHz, $CDCl_3$) δ (ppm) 204.5, 155.7, 141.7, 132.4 (d, J_{C-F} = 3.0 Hz), 132.0, 131.1, 130.8, 128.8, 128.1 (d, J_{C-F} = 3.1 Hz), 127.9, 127.1, 126.1 (t, J_{C-F} = 3.4 Hz), 125.3, 123.9 (d, J_{C-F} = 273.5 Hz), 122.7, 98.8, 96.3, 72.6, 52.2;

^{19}F NMR (376 MHz, $CDCl_3$) δ -62.9;

HRMS (ESI) exact mass calcd. For $[C_{25}H_{17}F_6NO_3SNa]^+$ requires *m/z* 548.0726, found *m/z* 548.0725;

$[\alpha]^{D}_{25}$ = 231.88 (c = 1.53, $CHCl_3$);

HPLC (Daicel Chirapak ID, hexane/ *i*-PrOH = 90/10, flow rate = 1.0 mL/min, T = 25 °C, 254 nm): t_R = 4.5 min (minor), t_R = 5.0 min (major). Absolute configuration is confirmed by X-ray.

Melting range:144.5-144.7 ^{o}C.

(*R*)-5-((3,5-bis(trifluoromethyl)phenyl)sulfonyl)-7-methyl-3-(2-phenylvinylidene)-2,3,4,5-tetrahydrobenzo[*b*][1,4]oxazepine (9b)

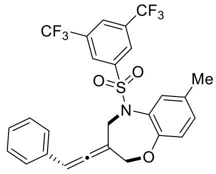

9b, 80%, 92% ee

(Flash column chromatography eluent:petroleum ether/EtOAc = 12:1); white solid, 43.1 mg, 80% yield, 92% ee;

^{1}H NMR (400 MHz, $CDCl_3$) δ (ppm) 8.03 (s, 1H), 7.99 (s, 2H), 7.40-7.39 (m, 1H), 7.26-7.14 (m, 4H), 7.01-6.98 (m, 2H), 6.84 (d, J = 8.2 Hz, 1H), 6.29 (s, 1H), 4.81 (d, J = 14.4 Hz, 1H), 4.31 (d, J = 12.3 Hz, 1H), 4.19-4.09 (m, 2H), 2.41 (s, 3H);

^{13}C NMR (100 MHz, $CDCl_3$) δ (ppm) 204.5, 153.4, 141.9, 135.2, 132.6 (q, $J_{C\text{-}F}$ = 34.4 Hz), 132.5, 132.2, 131.3, 130.8, 128.7, 128.1 (d, $J_{C\text{-}F}$ = 3.1 Hz), 127.8, 127.1, 126.0 (t, $J_{C\text{-}F}$ = 3.7 Hz), 124.0 (d, $J_{C\text{-}F}$ = 273.2 Hz), 122.3, 98.9, 96.1, 72.7, 52.2, 20.8;

^{19}F NMR (376 MHz, $CDCl_3$) δ -62.9;

HRMS (ESI) exact mass calcd. For $[C_{26}H_{19}F_6NO_3SNa]^+$ requires *m/z* 562.0882, found *m/z* 562.0880;

$[\alpha]_{25}^{D}$ = 130.92 (c = 1.01, $CHCl_3$);

HPLC (Daicel Chirapak ID, hexane/ *i*-PrOH = 90/10, flow rate = 1.0 mL/min, T = 25 °C, 254 nm): t_R = 12.0 min (minor), t_R = 12.8 min (major).

(*R*)-5-((3,5-bis(trifluoromethyl)phenyl)sulfonyl)-7-methoxy-3-(2-phenylvinylidene)-2,3,4,5-tetrahydrobenzo[*b*][1,4]oxazepine (9c)

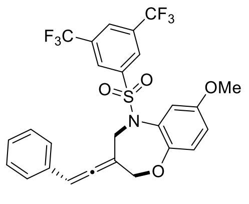

9c, 84%, 91% ee

(Flash column chromatography eluent:petroleum ether/EtOAc = 12:1); white solid, 46.6 mg, 84% yield, 91% ee;

^{1}H NMR (400 MHz, $CDCl_3$) δ (ppm) 8.04 (s, 1H), 8.00 (s, 2H), 7.27-7.21 (m, 3H), 7.13 (d, J = 2.8 Hz, 1H), 7.01-6.99 (m, 2H), 6.92-6.86 (m, 2H), 6.29 (s, 1H), 4.80 (d, J = 14.2 Hz, 1H), 4.29 (d, J = 12.3 Hz, 1H), 4.20-4.16 (m, 1H), 4.10-4.07 (m, 1H), 3.86 (s, 3H);

^{13}C NMR (100 MHz, $CDCl_3$) δ (ppm) 204.5, 156.6, 149.3, 141.6, 132.6 (q, $J_{C\text{-}F}$ = 34.2 Hz), 132.4, 131.8, 128.8, 128.1 (d, $J_{C\text{-}F}$ = 3.1 Hz), 127.9, 127.1, 126.1 (t, $J_{C\text{-}F}$ = 3.6 Hz), 124.0 (q, $J_{C\text{-}F}$ = 273.1 Hz), 123.1, 116.7, 116.2, 98.9, 96.1, 72.8, 55.9, 52.3;

^{19}F NMR (376 MHz, $CDCl_3$) δ -62.9;

HRMS (ESI) exact mass calcd. For $[C_{26}H_{19}F_6NO_4SNa]^+$ requires *m/z* 578.0831, found *m/z* 578.0831;

$[\alpha]_{25}^{D}$ = 142.48 (c = 1.03, $CHCl_3$);

HPLC (Daicel Chirapak ID, hexane/ *i*-PrOH = 90/10, flow rate = 1.0 mL/min, T = 25 °C, 254 nm): t_R = 4.8 min (minor), t_R = 5.1 min (major).

(*R*)-5-((3,5-bis(trifluoromethyl)phenyl)sulfonyl)-7-chloro-3-(2-phenylvinylidene)-2,3,4,5-tetrahydrobenzo[*b*][1,4]oxazepine (9d)

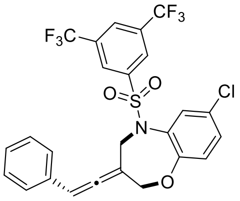

9d, 95%, 82% ee

(Flash column chromatography eluent:petroleum ether/EtOAc = 12:1); white solid, 53.2 mg, 95% yield, 82% ee;

^{1}H NMR (500 MHz, $CDCl_3$) δ (ppm) 8.06 (s, 1H), 8.02 (s, 2H), 7.61 (d, J = 2.5 Hz, 1H), 7.35-7.33 (m, 1H), 7.27-7.20 (m, 3H), 6.98-6.97 (m, 2H), 6.91 (d, J = 8.6 Hz, 1H), 6.31 (s, 1H), 4.78 (d, J = 14.3 Hz, 1H), 4.34 (d, J = 12.3 Hz, 1H), 4.22-4.14 (m, 2H);

^{13}C NMR (125 MHz, $CDCl_3$) δ (ppm) 204.9, 154.1, 141.6, 132.8 (d, $J_{C\text{-}F}$ = 34.1 Hz), 132.1 (d, $J_{C\text{-}F}$ = 2.5 Hz), 131.8, 130.7, 130.0, 128.8, 128.0, 127.1, 126.3 (t, $J_{C\text{-}F}$ = 3.2 Hz), 125.8 (q, $J_{C\text{-}F}$ = 272.6 Hz), 123.7, 98.4, 96.6, 72.8, 52.2;

^{19}F NMR (470 MHz, $CDCl_3$) δ -62.9;

HRMS (ESI) exact mass calcd. For $[C_{25}H_{16}ClF_6NO_3SNa]^+$ requires *m/z* 582.0336, found *m/z* 582.0334;

$[\alpha]_{25}^{D}$ = 98.21 (c = 1.03, $CHCl_3$);

HPLC (Daicel Chirapak ID, hexane/ *i*-PrOH = 95/5, flow rate = 1.0 mL/min, T = 25 °C, 254 nm): t_R = 5.3 min (minor), t_R = 6.2 min (major).

(*R*)-5-((3,5-bis(trifluoromethyl)phenyl)sulfonyl)-8-methyl-3-(2-phenylvinylidene)-2,3,4,5-tetrahydrobenzo[*b*][1,4]oxazepine (9e)

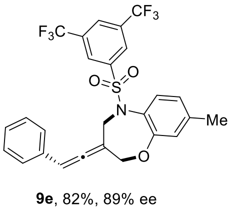

9e, 82%, 89% ee

(Flash column chromatography eluent:petroleum ether/EtOAc = 12:1); white solid, 44.2 mg, 82% yield, 89% ee;

^{1}H NMR (600 MHz, $CDCl_3$) δ (ppm) 8.03 (s, 1H), 7.98 (s, 2H), 7.46 (d, J = 8.0 Hz, 1H), 7.25-7.21 (m, 3H), 7.07-7.01 (m, 3H), 6.76 (s, 1H), 6.30 (s, 1H), 4.83 (d, J = 14.3 Hz, 1H), 4.32 (d, J = 12.3 Hz, 1H), 4.18-4.12 (m, 2H), 2.36 (s, 3H);

^{13}**C NMR** (100 MHz, $CDCl_3$) δ (ppm) 204.4, 155.5, 141.8 (d, $J_{C\text{-}F}$ = 29.8 Hz), 132.5, 132.3 (q, $J_{C\text{-}F}$ = 33.9 Hz), 131.5, 128.8, 128.2, 128.1, 127.8, 127.1, 126.0 (d, $J_{C\text{-}F}$ = 7.7 Hz), 124.0 (d, $J_{C\text{-}F}$ = 274.7 Hz), 123.0, 99.0, 96.1, 72.6, 52.2, 21.1;

^{19}**F NMR** (376 MHz, $CDCl_3$) δ -62.9;

HRMS (ESI) exact mass calcd. For $[C_{26}H_{19}F_6NO_3SNa]^+$ requires *m/z* 562.0882, found *m/z* 562.0884;

$[\alpha]^{D}_{25}$ = 95.17 (*c* = 1.23, $CHCl_3$);

HPLC (Daicel Chirapak ID, hexane/ *i*-PrOH = 95/5, flow rate = 0.7 mL/min, T = 25 °C, 254 nm): t_R = 7.0 min (minor), t_R = 7.5 min (major).

(*R*)-5-((3,5-bis(trifluoromethyl)phenyl)sulfonyl)-7-methyl-3-(2-(p-tolyl)vinylidene)-2,3,4,5-tetrahydrobenzo[*b*][1,4]oxazepine (9f)

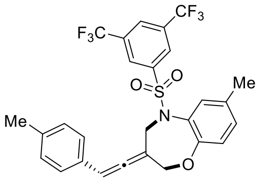

9f, 81%, 92% ee

(Flash column chromatography eluent:petroleum ether/EtOAc = 12:1); white solid, 44.9 mg, 81% yield, 92% ee;

^{1}**H NMR** (400 MHz, $CDCl_3$) δ (ppm) 8.03 (s, 1H), 7.99 (s, 2H), 7.40-7.39 (m, 1H), 7.17-7.14 (m, 1H), 7.04 (d, *J* = 7.9 Hz, 2H), 6.89 (d, *J* = 8.1 Hz, 2H), 6.83 (d, *J* = 8.2 Hz, 1H), 6.27 (s, 1H), 4.80 (d, *J* = 14.3 Hz, 1H), 4.29 (d, *J* = 12.2 Hz, 1H), 4.17-4.07 (m, 2H), 2.41 (s, 3H), 2.31 (s, 3H);

^{13}**C NMR** (100 MHz, $CDCl_3$) δ (ppm) 204.4, 153.4, 141.8, 137.8, 135.2, 132.6 (q, $J_{C\text{-}F}$ = 34.6 Hz), 132.2, 131.3, 130.8, 129.5, 129.4, 128.2 (d, $J_{C\text{-}F}$ = 3.2 Hz), 127.0, 126.0 (d, $J_{C\text{-}F}$ = 3.6 Hz), 124.0 (d, $J_{C\text{-}F}$ = 273.5 Hz), 122.3, 98.8, 95.9, 72.8, 52.3, 21.2, 20.8;

^{19}**F NMR** (470 MHz, $CDCl_3$) δ -62.9;

HRMS (ESI) exact mass calcd. For $[C_{27}H_{21}F_6NO_3SNa]^+$ requires *m/z* 576.1039, found *m/z* 576.1041;

$[\alpha]^{D}_{25}$ = 138.43 (*c* = 1.65, $CHCl_3$);

HPLC (Daicel Chirapak ID, hexane/ *i*-PrOH = 95/5, flow rate = 1.0 mL/min, T = 25 °C, 254 nm): t_R = 4.6 min (minor), t_R = 4.9 min (major).

(*R*)-3-(2-([1,1'-biphenyl]-4-yl)vinylidene)-5-((3,5-bis(trifluoromethyl)phenyl)sulfonyl)-7-methyl-2,3,4,5-tetrahydrobenzo[*b*][1,4]oxazepine (9g)

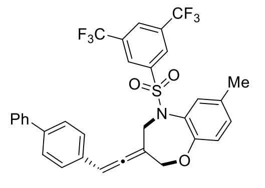

9g, 87%, 90% ee

(Flash column chromatography eluent:petroleum ether/EtOAc = 12:1); white solid, 53.6 mg, 87% yield, 90% ee;

^{1}H NMR (600 MHz, $CDCl_3$) δ (ppm) 8.04 (s, 1H), 8.00 (s, 2H), 7.55 (d, *J* = 7.7 Hz, 2H), 7.48-7.33 (m, 6H), 7.16 (d, *J* = 8.1 Hz, 1H), 7.07 (d, *J* = 7.4 Hz, 2H), 6.85 (d, *J* = 8.0 Hz, 1H), 6.34 (s, 1H), 4.83 (d, *J* = 14.3 Hz, 1H), 4.32 (d, *J* = 12.2 Hz, 1H), 4.19-4.11 (m, 2H), 2.42 (s, 3H);

^{13}C NMR (100 MHz, $CDCl_3$) δ (ppm) 204.7, 153.4, 141.9, 140.8 (d, J_{C-F} = 26.7 Hz), 135.3, 132.3 (q, J_{C-F} = 34.1 Hz), 132.2, 131.5, 131.4, 130.8, 128.9, 128.2, 127.5, 127.4, 127.0, 126.0 (q, J_{C-F} = 3.3 Hz), 124.0 (d, J_{C-F} = 273.6 Hz), 122.3, 99.1, 95.8, 72.7, 52.3, 20.8;

^{19}F NMR (376 MHz, $CDCl_3$) δ -62.9;

HRMS (ESI) exact mass calcd. For $[C_{32}H_{23}F_6NO_3SNa]^+$ requires *m/z* 638.1195, found *m/z* 638.1192;

$[\alpha]^{D}_{25}$ = 160.44 (*c* = 1.69, $CHCl_3$);

HPLC (Daicel Chirapak ID, hexane/ *i*-PrOH = 90/10, flow rate = 1.0 mL/min, T = 25 °C, 254 nm): t_R = 19.1 min (minor), t_R = 22.3 min (major).

(*R*)-5-((3,5-bis(trifluoromethyl)phenyl)sulfonyl)-3-(2-(4-methoxyphenyl)vinylidene)-7-methyl-2,3,4,5-tetrahydrobenzo[*b*][1,4]oxazepine (9h)

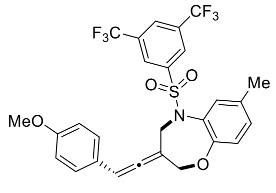

9h, 83%, 90% ee

(Flash column chromatography eluent:petroleum ether/EtOAc = 12:1); white solid, 47.3 mg, 83% yield, 90% ee;

^{1}H NMR (600 MHz, $CDCl_3$) δ (ppm) 8.03 (s, 1H), 7.99 (s, 2H), 7.39 (d, *J* = 1.6 Hz, 1H), 7.16-7.14 (m, 1H), 6.91 (d, *J* = 8.8 Hz, 2H), 6.83 (d, *J* = 8.2 Hz, 1H), 6.78-6.76 (m, 2H),

6.25 (s, 1H), 4.78 (d, J = 14.2 Hz, 1H), 4.29 (d, J = 12.2 Hz, 1H), 4.17-4.08 (m, 2H), 3.78 (s, 3H), 2.41 (s, 3H);

^{13}C NMR (150 MHz, $CDCl_3$) δ (ppm) 204.2, 159.4, 153.4, 141.9, 135.1, 132.5 (q, $J_{C\text{-}F}$ = 34.1 Hz), 132.1, 131.3, 130.8, 128.3, 128.1 (d, $J_{C\text{-}F}$ = 3.3 Hz), 125.9 (q, $J_{C\text{-}F}$ = 3.3 Hz), 125.3 (q, $J_{C\text{-}F}$ = 273.4 Hz), 124.6, 122.3, 114.3, 98.8, 95.6, 72.9, 55.3, 52.4, 20.7;

^{19}F NMR (564 MHz, $CDCl_3$) δ -62.9;

HRMS (ESI) exact mass calcd. For $[C_{27}H_{21}F_6NO_4SNa]^+$ requires m/z 592.0988, found m/z 592.0990;

$[\alpha]^{D}_{25}$ = 187.22 (c = 1.67, $CHCl_3$);

HPLC (Daicel Chirapak IB-3, hexane/ i-PrOH = 90/10, flow rate = 1.0 mL/min, T = 25 °C, 254 nm): t_R = 11.7 min (minor), t_R = 12.8 min (major).

(*R*)-5-((3,5-bis(trifluoromethyl)phenyl)sulfonyl)-3-(2-(3-fluorophenyl)vinylidene)-7-methyl-2,3,4,5-tetrahydrobenzo[*b*][1,4]oxazepine (9i)

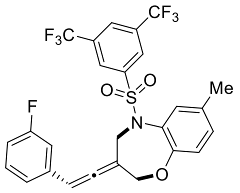

9i, 78%, 84% ee

(Flash column chromatography eluent:petroleum ether/EtOAc = 12:1); white solid, 43.4 mg, 78% yield, 84% ee;

^{1}H NMR (600 MHz, $CDCl_3$) δ (ppm) 8.04 (s, 1H), 7.99 (s, 2H), 7.40 (s, 1H), 7.22-7.16 (m, 2H), 6.91-6.81 (m, 3H), 6.60 (d, J = 9.7 Hz, 1H), 6.26 (s, 1H), 4.80 (d, J = 14.2 Hz, 1H), 4.32 (d, J = 12.2 Hz, 1H), 4.19-4.10 (m, 2H), 2.41 (s, 3H);

^{13}C NMR (100 MHz, $CDCl_3$) δ (ppm) 204.6, 164.3 (d, $J_{C\text{-}F}$ = 246.1 Hz), 153.2, 141.8, 135.5, 134.9 (d, $J_{C\text{-}F}$ = 7.7 Hz), 132.3 (q, $J_{C\text{-}F}$ = 34.4 Hz), 132.1, 131.5, 130.6, 130.2 (d, $J_{C\text{-}F}$ = 8.4 Hz), 128.1, 126.0 (t, $J_{C\text{-}F}$ = 2.7 Hz), 123.9 (q, $J_{C\text{-}F}$ = 274.1 Hz), 123.0, 122.2, 114.9 (d, $J_{C\text{-}F}$ = 21.5 Hz), 113.6 (d, $J_{C\text{-}F}$ = 22.3 Hz), 99.4, 95.4, 72.4, 52.0, 20.7;

^{19}F NMR (376 MHz, $CDCl_3$) -62.9, -112.9;

HRMS (ESI) exact mass calcd. For $[C_{26}H_{18}F_7NO_3SNa]^+$ requires m/z 580.0788, found m/z 580.0787;

$[\alpha]^{D}_{25}$ = 92.36 (c = 1.27, $CHCl_3$);

HPLC (Daicel Chirapak ID, hexane/ i-PrOH = 95/5, flow rate = 1.0 mL/min, T = 25 °C, 254 nm): t_R = 4.8 min (minor), t_R = 5.0 min (major).

(*R*)-5-((3,5-bis(trifluoromethyl)phenyl)sulfonyl)-7-methyl-3-(2-(m-tolyl)vinylidene)-2,3,4,5-tetrahydrobenzo[*b*][1,4]oxazepine (9j)

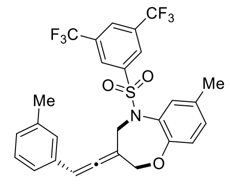

9j, 86%, 90% ee

(Flash column chromatography eluent:petroleum ether/EtOAc = 12:1); white solid, 47.6 mg, 86% yield, 90% ee;

^{1}H NMR (500 MHz, $CDCl_3$) δ (ppm) 8.03 (s, 1H), 7.99 (s, 2H), 7.40-7.39 (m, 1H), 7.16-7.11 (m, 2H), 7.02 (d, J = 7.5 Hz, 1H), 6.84-6.80 (m, 3H), 6.26 (s, 1H), 4.82 (d, J = 14.3 Hz, 1H), 4.31 (d, J = 12.3 Hz, 1H), 4.18-4.09 (m, 2H), 2.41 (s, 3H), 2.27 (s, 3H);

^{13}C NMR (125 MHz, $CDCl_3$) δ (ppm) 204.5, 153.4, 141.8, 138.4, 135.2, 132.3, 132.2 (q, $J_{C\text{-}F}$ = 34.6 Hz), 132.1, 131.4, 130.7, 128.7, 128.6, 128.1 (d, $J_{C\text{-}F}$ = 3.1 Hz), 127.7, 126.0 (t, $J_{C\text{-}F}$ = 3.6 Hz), 124.3, 123.7 (q, $J_{C\text{-}F}$ = 273.2 Hz), 122.2, 98.7, 96.2, 72.6, 52.3, 21.2, 20.8;

^{19}F NMR (470 MHz, $CDCl_3$) -62.9;

HRMS (ESI) exact mass calcd. For $(C_{27}H_{21}F_6NO_3SNa)^+$ requires *m/z* 576.1039, found *m/z* 576.1040;

$[\alpha]^{D}_{25}$ = 113.44 (c = 1.43, $CHCl_3$);

HPLC (Daicel Chirapak ID, hexane/ *i*-PrOH = 95/5, flow rate = 1.0 mL/min, T = 25 °C, 254 nm): t_R = 6.3 min (minor), t_R = 6.9 min (major).

(*R*)-5-((3,5-bis(trifluoromethyl)phenyl)sulfonyl)-7-methyl-3-(2-(o-tolyl)vinylidene)-2,3,4,5-tetrahydrobenzo[*b*][1,4]oxazepine (9k)

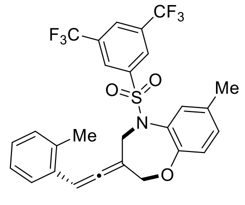

9k, 82%, 87% ee

(Flash column chromatography eluent:petroleum ether/EtOAc = 12:1); white solid, 45.4 mg, 82% yield, 87% ee;

^{1}H NMR (600 MHz, $CDCl_3$) δ (ppm) 8.03 (s, 1H), 7.99 (s, 2H), 7.39 (s, 1H), 7.15-7.11 (m, 3H), 7.05-7.03 (m, 1H), 6.91 (d, J = 7.6 Hz, 1H), 6.83 (d, J = 8.0 Hz, 1H), 6.51 (s,

1H), 4.86 (d, J = 14.5 Hz, 1H), 4.31 (d, J = 12.3 Hz, 1H), 4.14-4.06 (m, 2H), 2.40 (s, 3H), 2.31 (s, 3H);

^{13}C NMR (100 MHz, $CDCl_3$) δ (ppm) 204.9, 153.6, 141.9, 135.6, 135.2, 132.2, 131.9, 131.3, 130.8, 130.7 (d, $J_{C\text{-}F}$ = 2.3 Hz), 128.2, 127.8, 127.5, 126.2, 126.0 (t, $J_{C\text{-}F}$ = 3.5 Hz), 124.0 (d, $J_{C\text{-}F}$ = 274.5 Hz), 122.2, 97.9, 93.4, 72.8, 52.4, 20.8, 19.7;

^{19}F NMR (376 MHz, $CDCl_3$) δ -62.9;

HRMS (ESI) exact mass calcd. For $[C_{27}H_{21}F_6NO_3SNa]^+$ requires *m/z* 576.1039, found *m/z* 576.1040;

$[\alpha]^{D}_{25}$ = 131.29 (c = 1.68, $CHCl_3$);

HPLC (Daicel Chirapak ID, hexane/ *i*-PrOH = 90/10, flow rate = 1.0 mL/min, T = 25 °C, 254 nm): t_R = 15.1 min (minor), t_R = 16.0 min (major).

(*R*)-5-((3,5-bis(trifluoromethyl)phenyl)sulfonyl)-7-methyl-3-(2-(naphthalen-2-yl)vinylidene)-2,3,4,5-tetrahydrobenzo[*b*][1,4]oxazepine (9l)

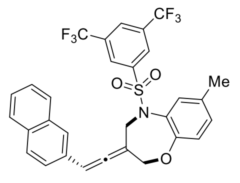

9l, 95%, 91% ee

(Flash column chromatography eluent:petroleum ether/EtOAc = 12:1); white solid, 56.1 mg, 95% yield, 91% ee;

^{1}H NMR (400 MHz, $CDCl_3$) δ (ppm) 8.05 (s, 1H), 8.02 (s, 2H), 7.77-7.72 (m, 2H), 7.65 (d, J = 8.6 Hz, 1H), 7.53 (s, 1H), 7.46-7.41 (m, 3H), 7.19-7.17 (m, 1H), 7.01-6.99 (m, 1H), 6.86 (d, J = 8.1 Hz, 1H), 6.47 (s, 1H), 4.86 (d, J = 14.4 Hz, 1H), 4.35 (d, J = 12.3 Hz, 1H), 4.23-4.14 (m, 2H), 2.43 (s, 3H);

^{13}C NMR (100 MHz, $CDCl_3$) δ (ppm) 205.0, 153.4, 141.9, 135.3, 133.5, 132.9, 132.3 (q, $J_{C\text{-}F}$ = 34.1 Hz), 132.2, 131.4, 130.8, 129.9, 128.4, 128.2 (d, $J_{C\text{-}F}$ = 3.2 Hz), 127.8, 127.7, 126.5, 126.2, 126.0 (t, $J_{C\text{-}F}$ = 3.6 Hz), 124.4, 124.0 (d, $J_{C\text{-}F}$ = 273.5 Hz), 122.3, 99.2, 96.5, 72.7, 52.3, 20.8;

^{19}F NMR (376 MHz, $CDCl_3$) δ -62.9;

HRMS (ESI) exact mass calcd. For $[C_{30}H_{21}F_6NO_3SNa]^+$ requires *m/z* 612.1039, found *m/z* 612.1042;

$[\alpha]^{D}_{25}$ = 169.74 (c = 1.49, $CHCl_3$);

HPLC (Daicel Chirapak ID, hexane/ *i*-PrOH = 90/10, flow rate = 1.0 mL/min, T = 25 °C, 254 nm): t_R = 14.1 min (minor), t_R = 15.8 min (major).

Spectral data of products 10

***N*-(2-hydroxyphenyl)-*N*-(2-methylene-4-phenylbut-3-yn-1-yl)benzenesulfonamide (10a)**

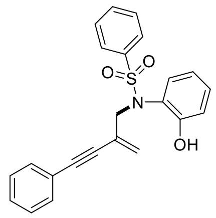

10a, 85%

(Flash column chromatography eluent:petroleum ether/EtOAc = 10:1); yellow oily liquid, 33.1 mg, 85% yield;

^{1}H NMR (400 MHz, CD_3OD) δ (ppm) 7.71(d, J = 7.8 Hz, 2H), 7.60-7.56 (m, 1H), 7.48-7.44 (m, 4H), 7.36 (s, 3H), 7.21-7.12 (m, 2H), 6.76-6.73 (m, 2H), 5.41 (d, J = 11.5 Hz, 2H), 4.66 (s, 1H), 4.46 (s, 2H);

^{13}C NMR (100 MHz, CD_3OD) δ (ppm) 154.8, 139.9, 132.7, 132.4, 131.3, 129.5, 128.4, 128.2, 128.1, 127.9, 127.3, 124.3, 122.9, 122.8, 118.8, 116.0, 90.4, 87.5, 54.3;

HRMS (ESI) exact mass calcd. For $[C_{23}H_{19}NO_3SNa]^+$ requires *m/z* 412.0978, found *m/z* 412.0976.

***N*-(2-hydroxyphenyl)-*N*-(4-(4-methoxyphenyl)-2-methylenebut-3-yn-1-yl)benzene sulfonamide (10b)**

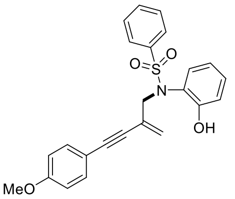

10b, 72%

(Flash column chromatography eluent:petroleum ether/EtOAc = 10:1); yellow oily liquid, 30.2 mg, 72% yield;

^{1}H NMR (500 MHz, $CDCl_3$) δ (ppm) 7.70-7.68 (m, 2H), 7.61 (t, J = 7.5 Hz, 1H), 7.48-7.43 (m, 4H), 7.20-7.17 (m, 1H), 7.03-7.01 (m, 1H), 6.88-6.85 (m, 2H), 6.74 (s, 1H), 6.71-6.67 (m, 1H), 6.43-6.41 (m, 1H), 5.41 (s, 1H), 5.24 (d, J = 0.8 Hz, 1H), 5.06-3.96 (m, 2H), 4.50 (b, 2H), 3.82 (s, 3H);

^{13}C NMR (125 MHz, $CDCl_3$) δ (ppm) 160.0, 155.2, 137.2, 133.4, 133.3, 130.3, 129.0, 128.1, 127.9, 126.4, 125.3, 124.1, 120.4, 117.5, 114.4, 114.1, 91.9, 86.0, 57.4, 55.3;
HRMS (ESI) exact mass calcd. For $[C_{24}H_{22}NO_4S]^+$ requires *m/z* 420.1264, found *m/z* 420.1265.

2,4,6-trimethyl-*N*-(2-methylene-4-phenylbut-3-yn-1-yl)-*N*-phenylbenzenesulfonamide (10c)

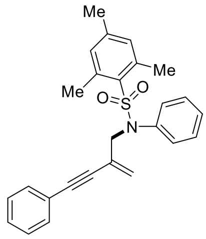

10c, 61%

(Flash column chromatography eluent:petroleum ether/EtOAc = 10:1); yellow oily liquid, 25.3 mg, 61% yield;
^{1}H NMR (400 MHz, $CDCl_3$) δ (ppm) 7.42-7.39 (m, 2H), 7.33-7.31 (m, 3H), 7.26-7.20 (m, 5H), 6.83 (s, 2H), 5.43 (s, 1H), 5.32 (s, 1H), 4.46 (s, 2H), 2.47 (s, 6H), 2.25 (s, 3H);
^{13}C NMR (100 MHz, $CDCl_3$) δ (ppm) 142.6, 140.6, 138.6, 132.6, 131.8, 131.7, 129.8, 129.0, 128.4, 128.3, 128.1, 126.9, 125.0, 122.9, 90.8, 87.9, 55.7, 23.1, 21.0;
HRMS (ESI) exact mass calcd. For $[C_{26}H_{25}NO_2S]^+$ requires *m/z* 438.1498, found *m/z* 438.1499.

***N*-(2-(tert-butyl)phenyl)-2,4,6-trimethyl-*N*-(2-methyleneundec-3-yn-1-yl)benzene sulfonamide (10d)**

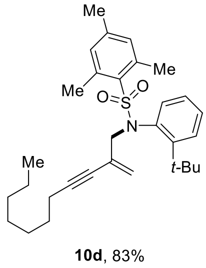

10d, 83%

(Flash column chromatography eluent:petroleum ether/EtOAc = 10:1); yellow oily liquid, 37.7 mg, 83% yield;
^{1}H NMR (400 MHz, $CDCl_3$) δ (ppm) 7.54-7.52 (m, 1H), 7.23-7.21 (m, 1H), 6.91-6.89 (m, 3H), 6.72-6.70 (m, 1H), 5.30 (s, 1H), 5.13 (s, 1H), 4.44 (d, *J* = 13.3 Hz, 1H), 4.29 (d,

J = 13.3 Hz, 1H), 2.28 (s, 3H), 2.25 (s, 6H), 2.09 (t, *J* = 7.0 Hz, 2H), 1.48-1.47 (m, 9H), 1.39-1.25 (m, 10H), 0.90-0.87 (m, 3H);

^{13}C NMR (100 MHz, $CDCl_3$) *δ* (ppm) 150.8, 141.8, 140.2, 135.4, 133.9, 132.3, 132.2, 132.1, 130.9, 128.1, 126.7, 126.4, 125.5, 125.3, 92.7, 80.2, 58.3, 37.1, 32.9, 31.7, 28.9, 28.8, 28.4, 24.2, 22.6, 20.9, 19.3, 14.1;

HRMS (ESI) exact mass calcd. For $[C_{31}H_{44}NO_2S]^+$ requires *m/z* 494.3087, found *m/z* 494.3089.

10. NMR spectra

NMR spectra of 1

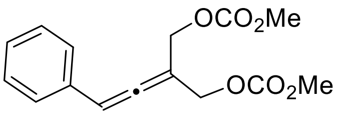

1a

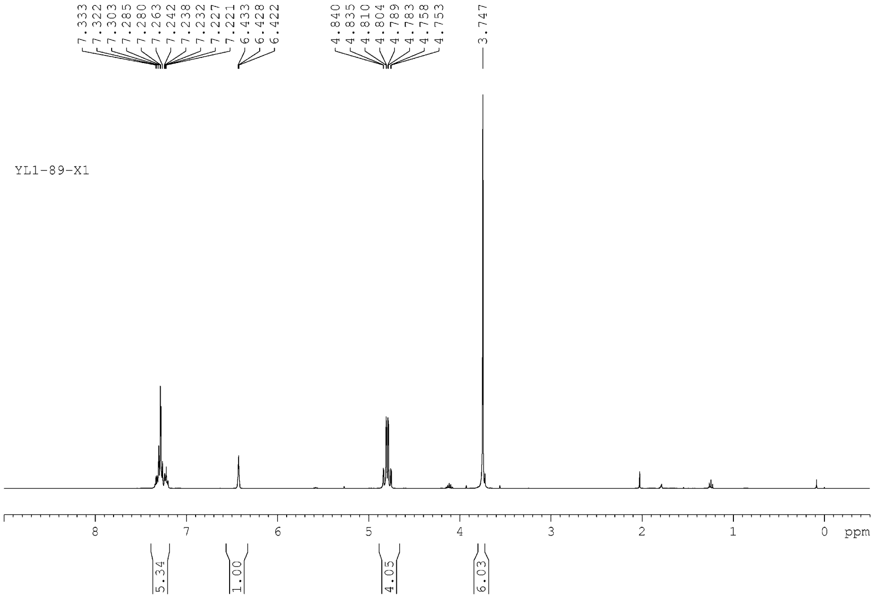

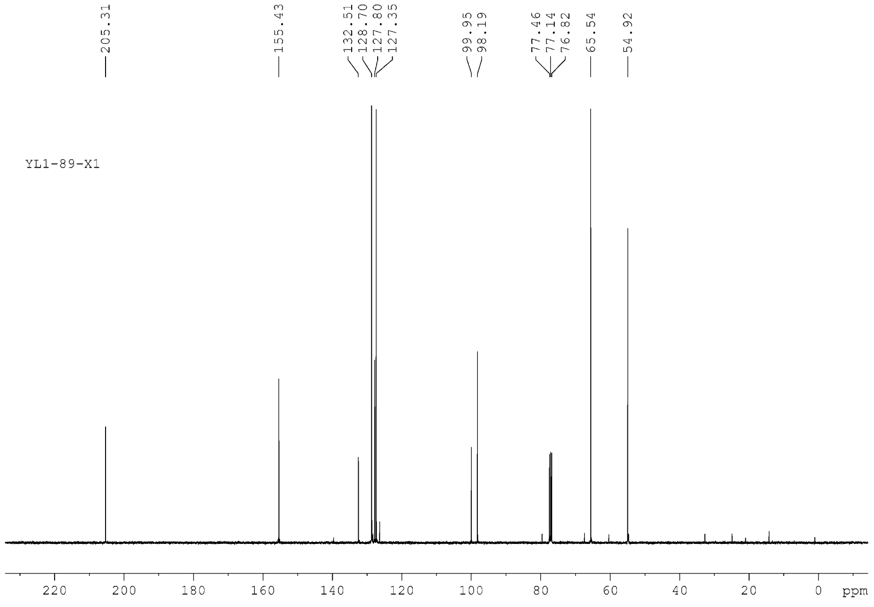

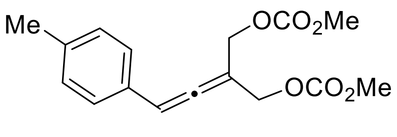
Me
OCO2Me
OCO2Me

1b

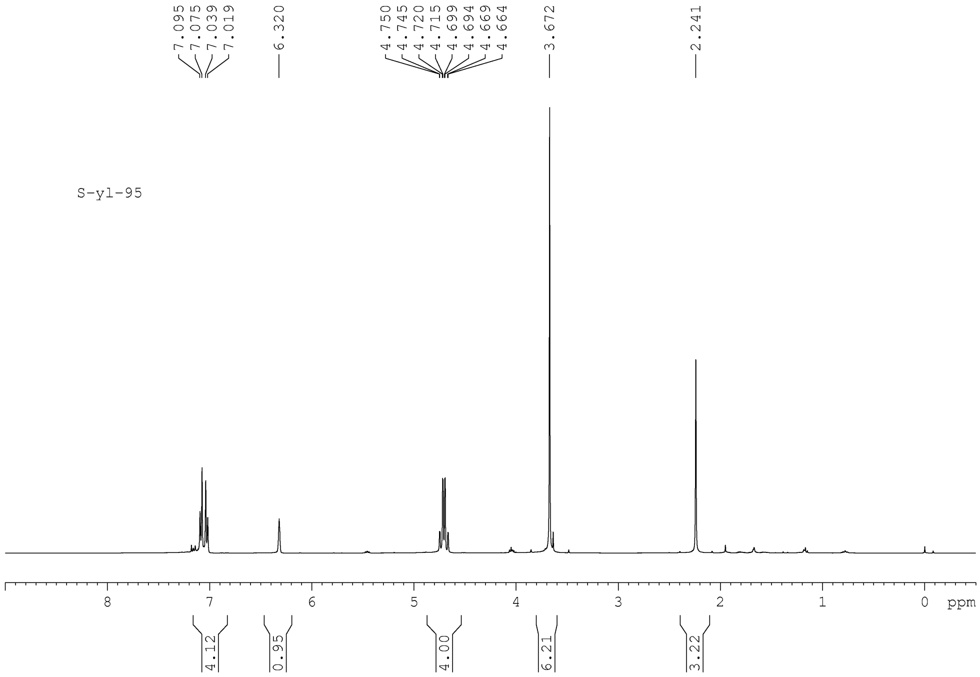
7.095
7.075
7.039
7.019
6.320
4.750
4.745
4.720
4.715
4.699
4.694
4.669
4.664
3.672
2.241
S-yl-95
8
7
6
5
4
3
2
1
0 ppm
4.12
0.95
4.00
6.21
3.22

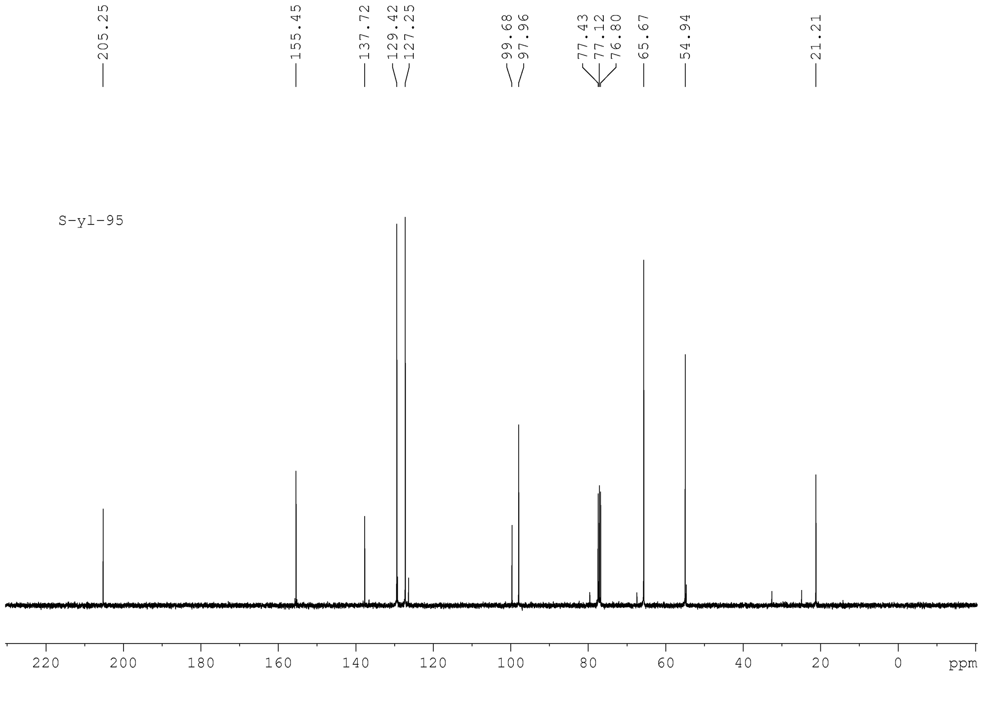
205.25
155.45
137.72
129.42
127.25
99.68
97.96
77.43
77.12
76.80
65.67
54.94
21.21
S-yl-95
220
200
180
160
140
120
100
80
60
40
20
0
ppm

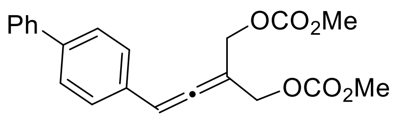
Ph
OCO2Me
OCO2Me

1c

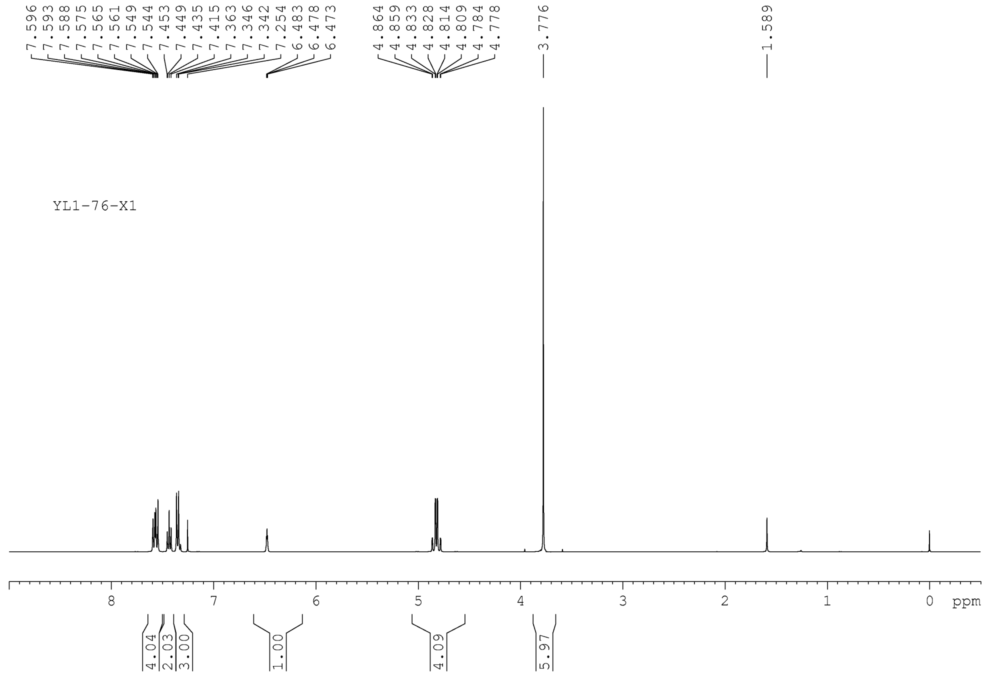
7.596 7.593 7.588 7.575 7.565 7.561 7.549 7.544 7.453 7.449 7.435 7.415 7.363 7.346 7.342 7.254 6.483 6.478 6.473
4.864 4.859 4.833 4.828 4.814 4.809 4.784 4.778
3.776
1.589
YL1-76-X1
8 7 6 5 4 3 2 1 0 ppm
4.04 2.03 3.00 1.00 4.09 5.97

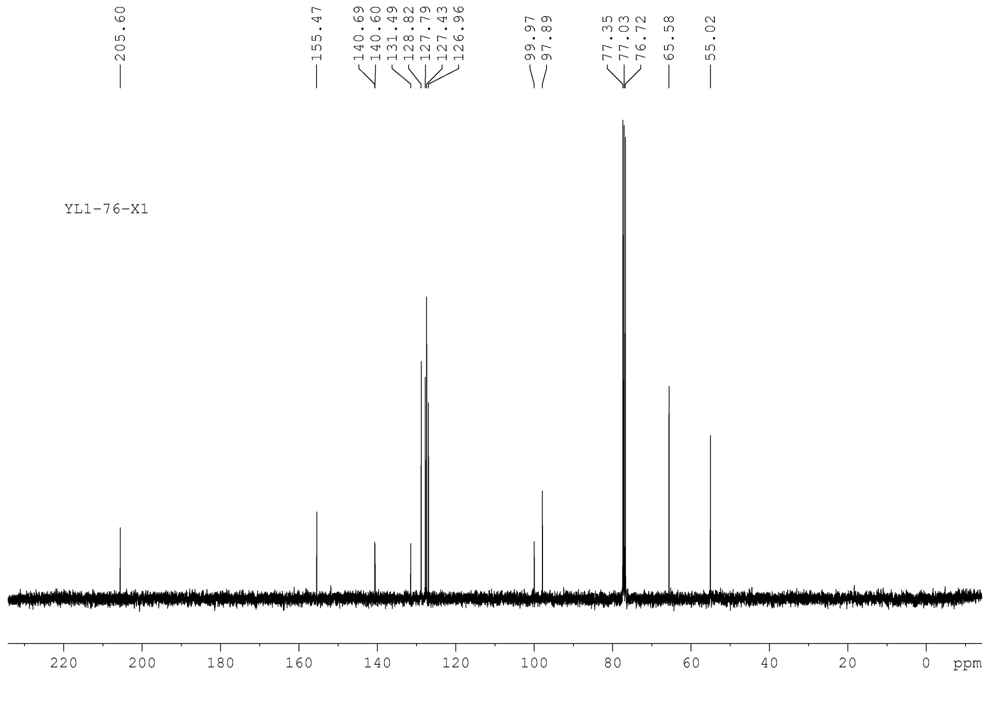
205.60
155.47
140.69 140.60 131.49 128.82 127.79 127.43 126.96
99.97 97.89
77.35 77.03 76.72
65.58
55.02
YL1-76-X1
220 200 180 160 140 120 100 80 60 40 20 0 ppm

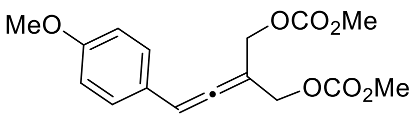
MeO
OCO2Me
OCO2Me

1d

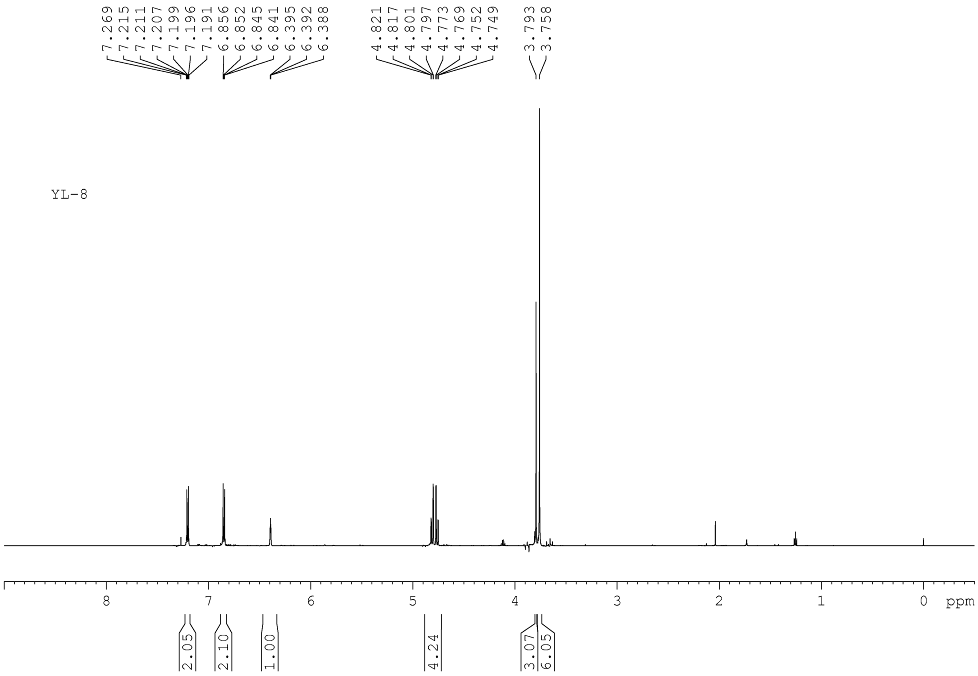
7.269
7.215
7.211
7.207
7.199
7.196
7.191
6.856
6.852
6.845
6.841
6.395
6.392
6.388
4.821
4.817
4.801
4.797
4.773
4.769
4.752
4.749
3.793
3.758
YL-8
8
7
6
5
4
3
2
1
0
ppm
2.05
2.10
1.00
4.24
3.07
6.05

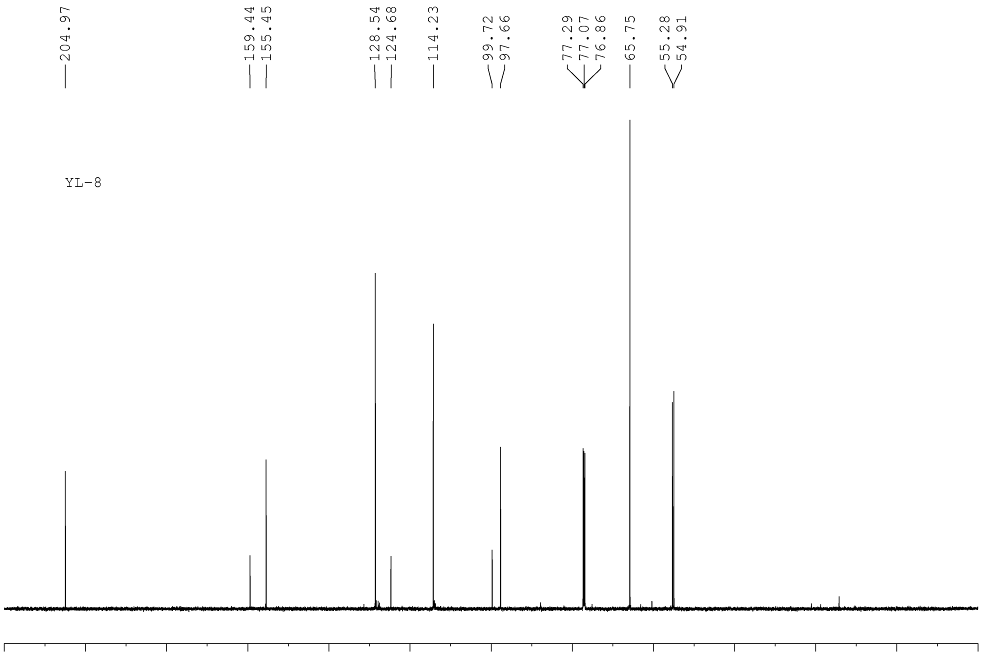
204.97
159.44
155.45
128.54
124.68
114.23
99.72
97.66
77.29
77.07
76.86
65.75
55.28
54.91
YL-8
200
180
160
140
120
100
80
60
40
20
0
ppm

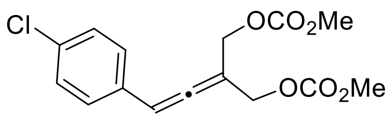
Cl
OCO2Me
OCO2Me

1e

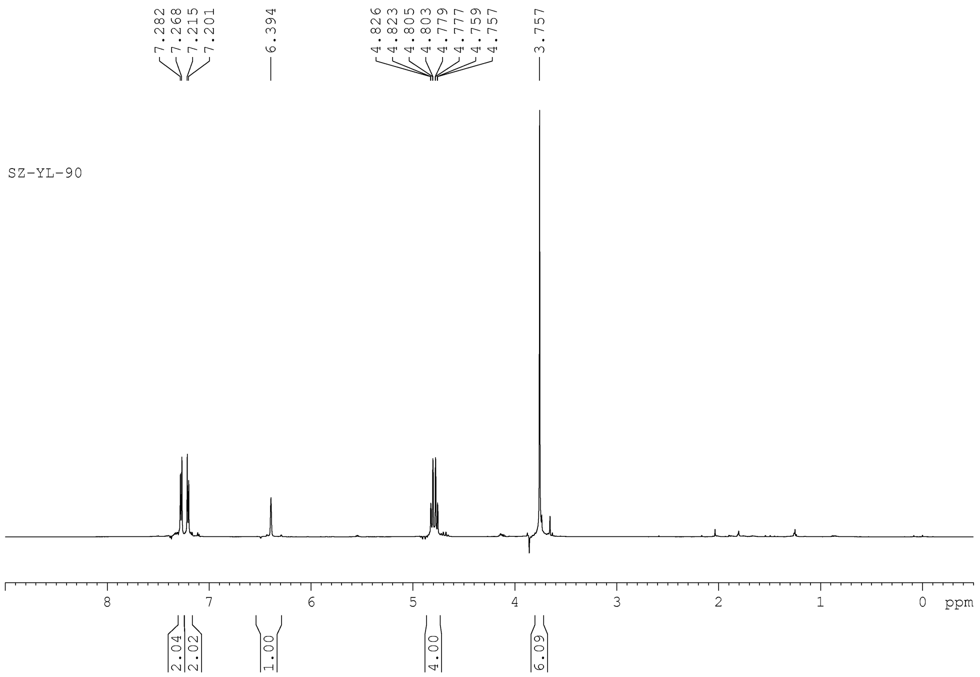
7.282
7.268
7.215
7.201
6.394
4.826
4.823
4.805
4.803
4.779
4.777
4.759
4.757
3.757
SZ-YL-90
8 7 6 5 4 3 2 1 0 ppm
2.04
2.02
1.00
4.00
6.09

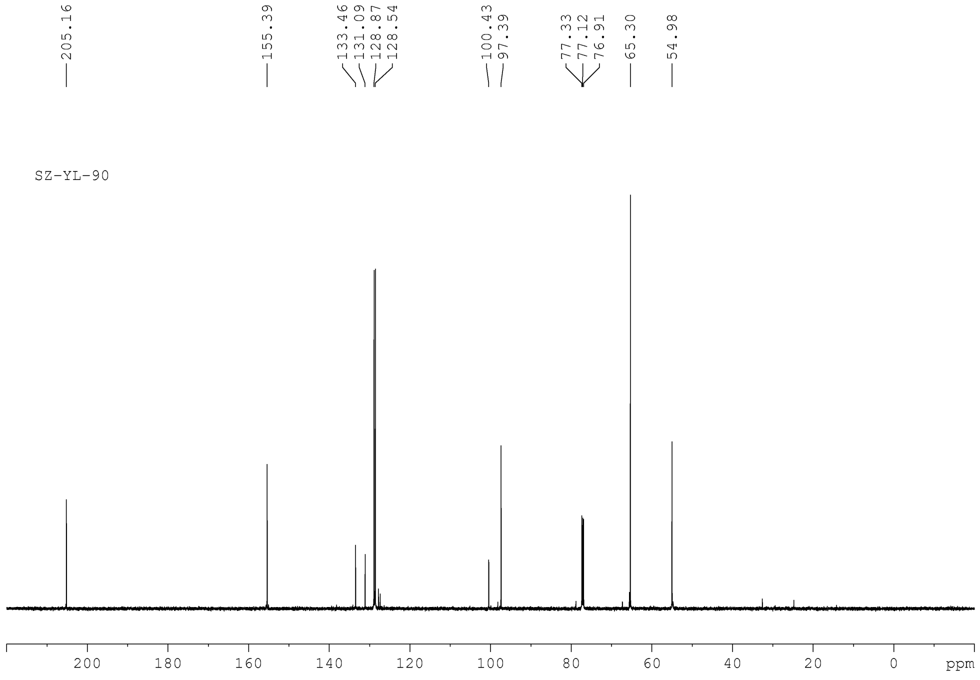
205.16
155.39
133.46
131.09
128.87
128.54
100.43
97.39
77.33
77.12
76.91
65.30
54.98
SZ-YL-90
200 180 160 140 120 100 80 60 40 20 0 ppm

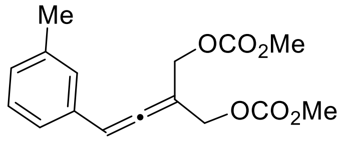
Me
OCO2Me
OCO2Me

1f

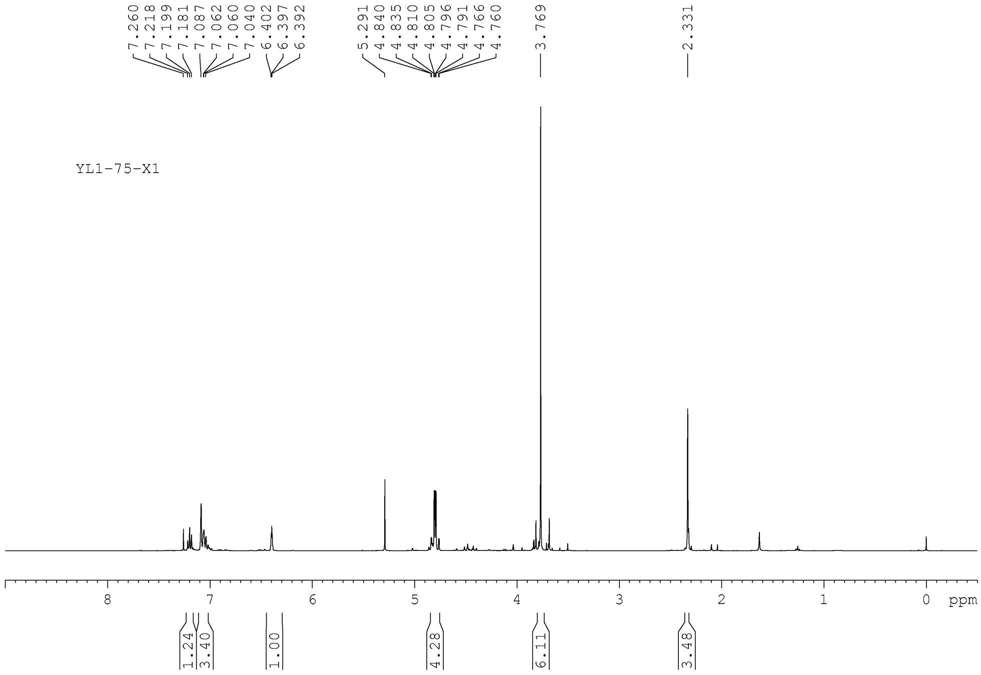
7.260
7.218
7.199
7.181
7.087
7.062
7.060
7.040
6.402
6.397
6.392
5.291
4.840
4.835
4.810
4.805
4.796
4.791
4.766
4.760
3.769
2.331
YL1-75-X1
8
7
6
5
4
3
2
1
0 ppm
1.24
3.40
1.00
4.28
6.11
3.48

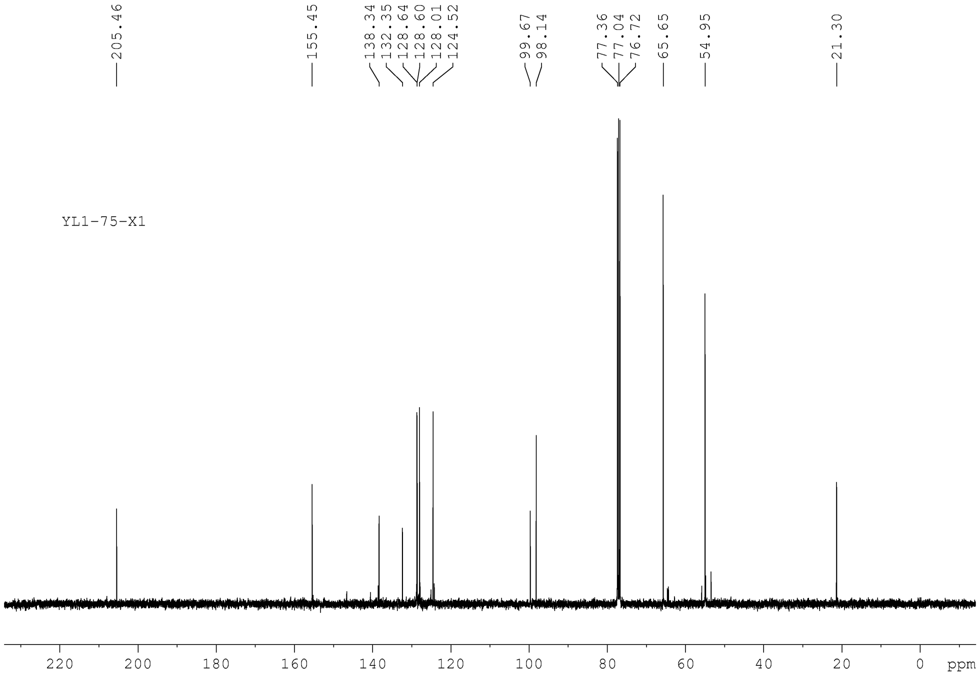
205.46
155.45
138.34
132.35
128.64
128.60
128.01
124.52
99.67
98.14
77.36
77.04
76.72
65.65
54.95
21.30
YL1-75-X1
220
200
180
160
140
120
100
80
60
40
20
0 ppm

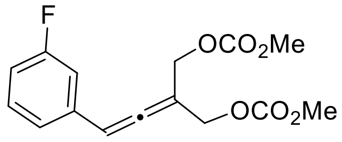
F
OCO2Me
OCO2Me

1g

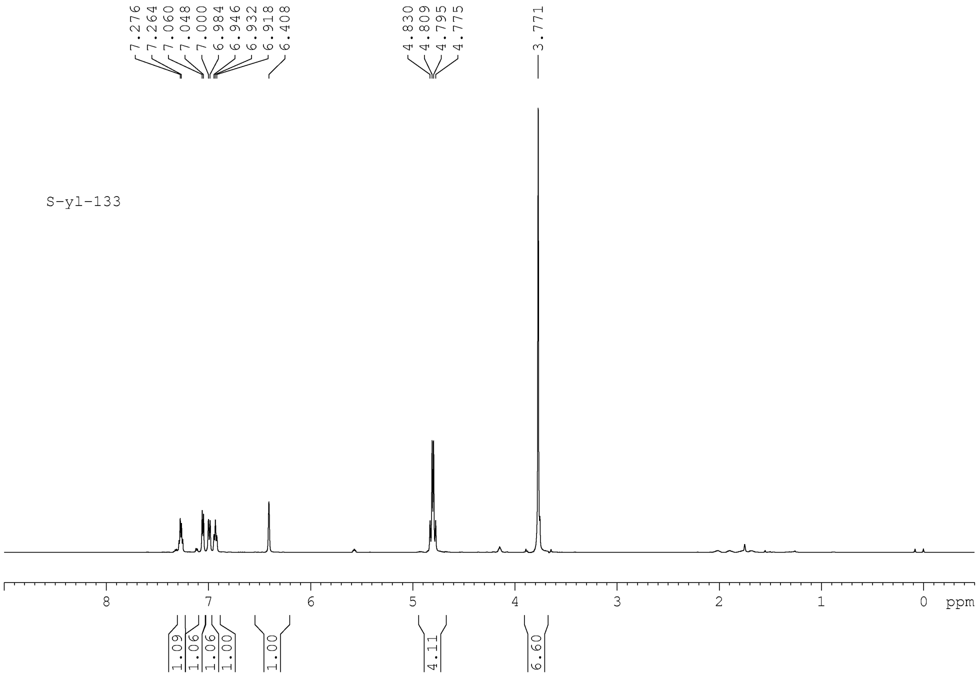
7.276
7.264
7.060
7.048
7.000
6.984
6.946
6.932
6.918
6.408
4.830
4.809
4.795
4.775
3.771
S-yl-133
8
7
6
5
4
3
2
1
0
ppm
1.09
1.06
1.06
1.00
1.00
4.11
6.60

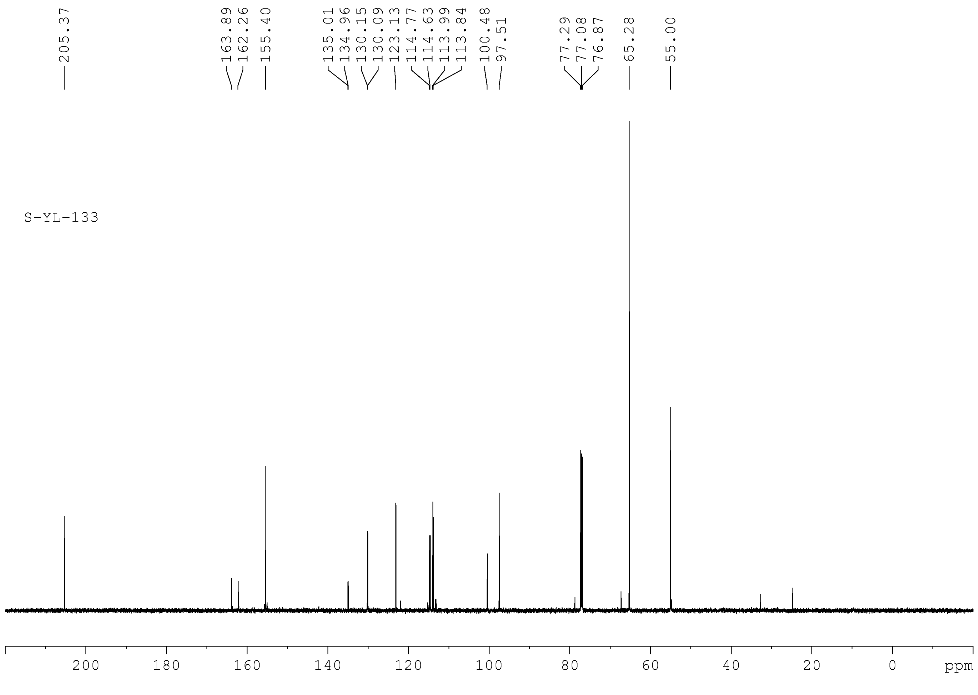
205.37
163.89
162.26
155.40
135.01
134.96
130.15
130.09
123.13
114.77
114.63
113.99
113.84
100.48
97.51
77.29
77.08
76.87
65.28
55.00
S-YL-133
200
180
160
140
120
100
80
60
40
20
0
ppm

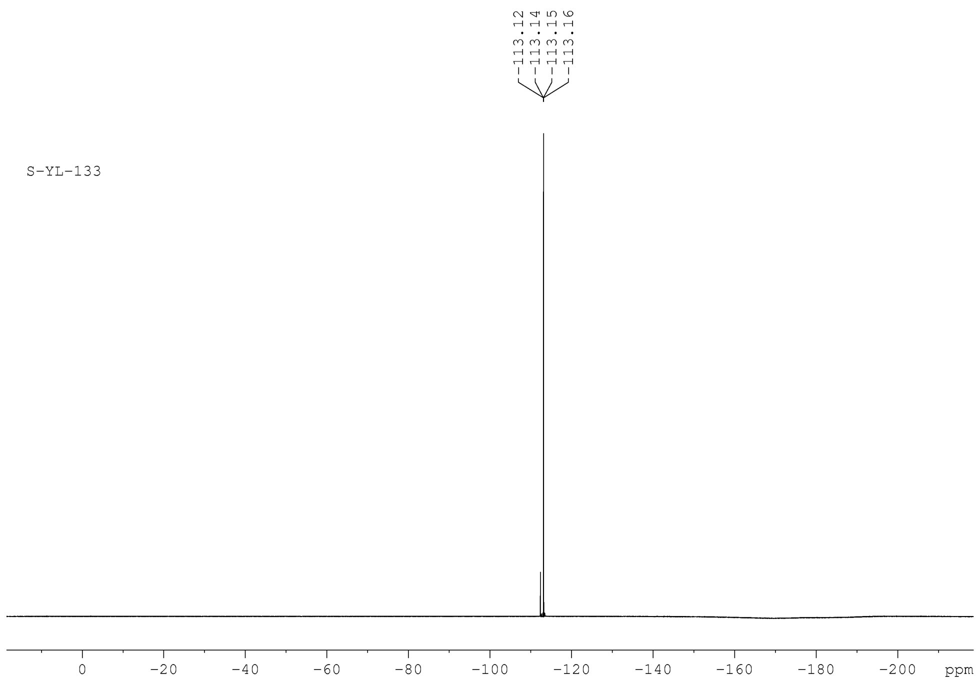
-113.12
-113.14
-113.15
-113.16
S-YL-133
0
-20
-40
-60
-80
-100
-120
-140
-160
-180
-200
ppm

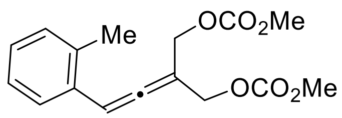

1h

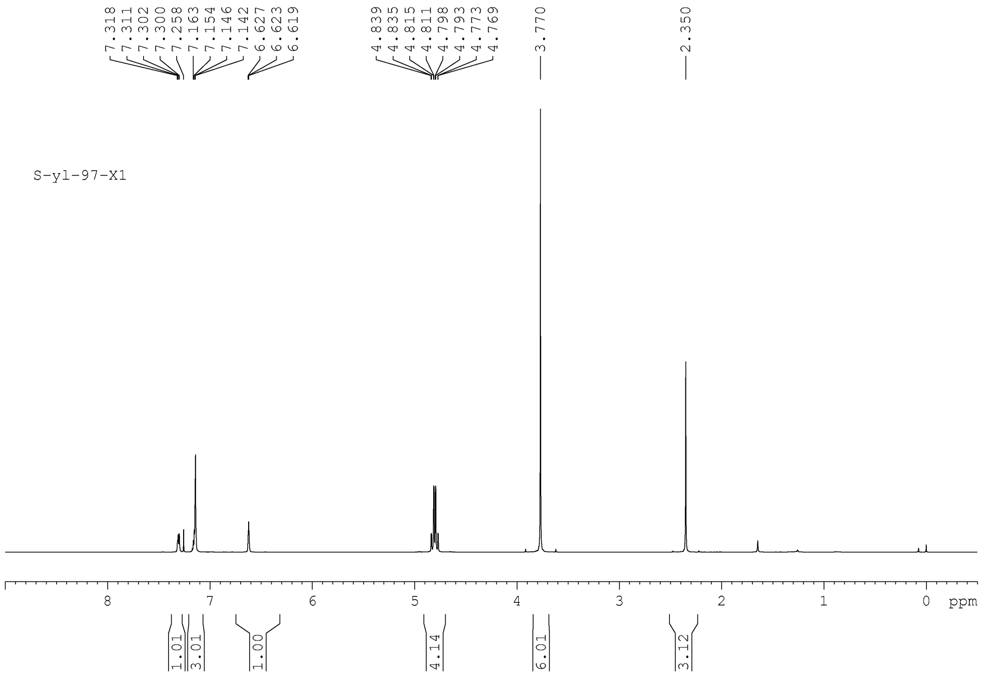

7.318
7.311
7.302
7.300
7.258
7.163
7.154
7.146
7.142
6.627
6.623
6.619
4.839
4.835
4.815
4.811
4.798
4.793
4.773
4.769
3.770
2.350
S-yl-97-X1
1.01
3.01
1.00
4.14
6.01
3.12
ppm

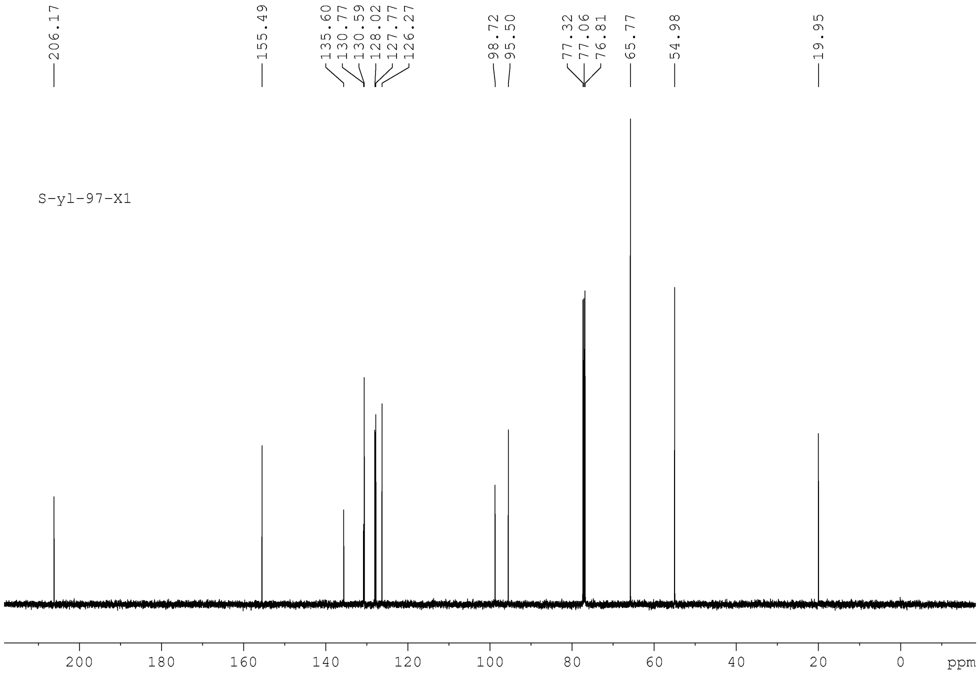

206.17
155.49
135.60
130.77
130.59
128.02
127.77
126.27
98.72
95.50
77.32
77.06
76.81
65.77
54.98
19.95
S-yl-97-X1
ppm

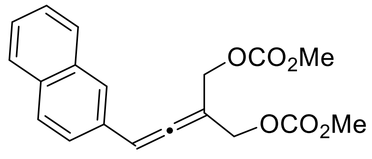

1i

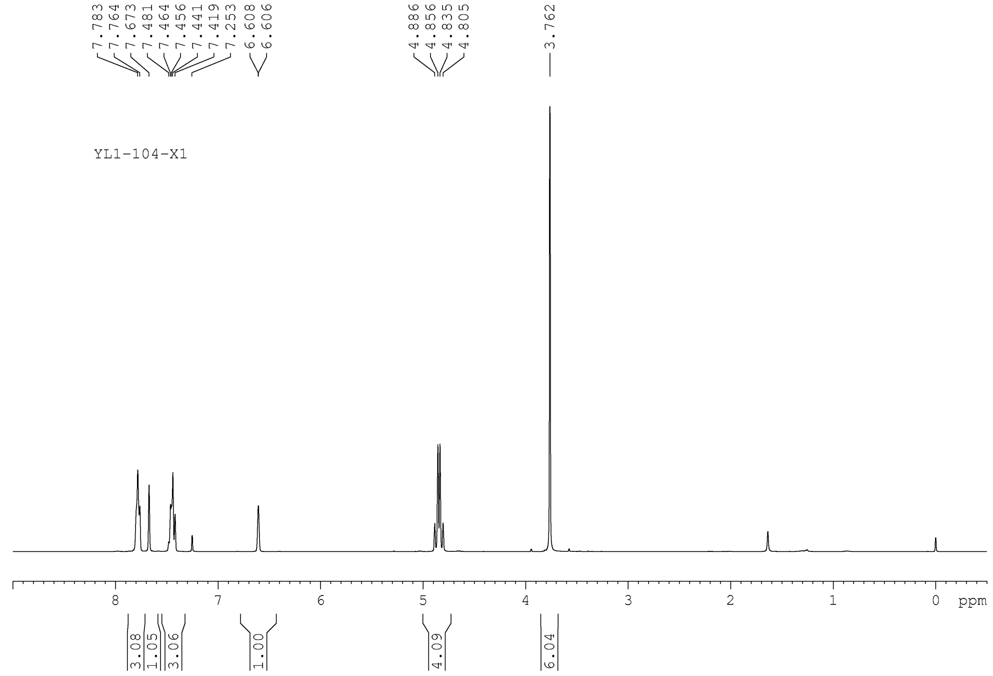
7.783
7.764
7.673
7.481
7.464
7.456
7.441
7.419
7.253
6.608
6.606
4.886
4.856
4.835
4.805
3.762
YL1-104-X1
8
7
6
5
4
3
2
1
0 ppm
3.08
1.05
3.06
1.00
4.09
6.04

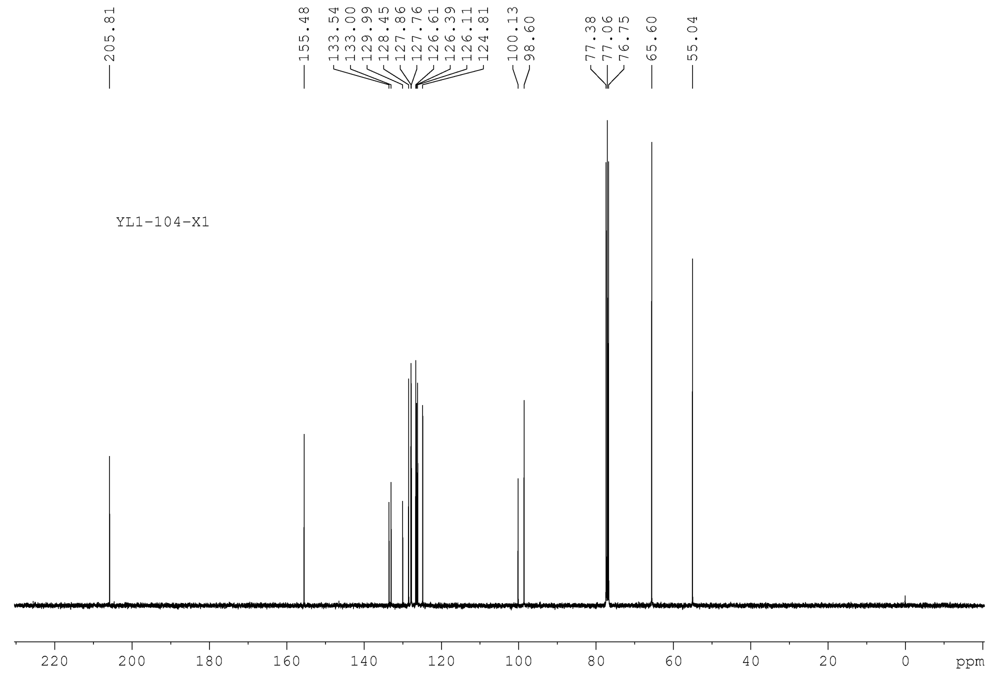
205.81
155.48
133.54
133.00
129.99
128.45
127.86
127.76
126.61
126.39
126.11
124.81
100.13
98.60
77.38
77.06
76.75
65.60
55.04
YL1-104-X1
220
200
180
160
140
120
100
80
60
40
20
0
ppm

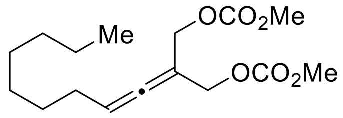

Me
OCO2Me
OCO2Me

1j

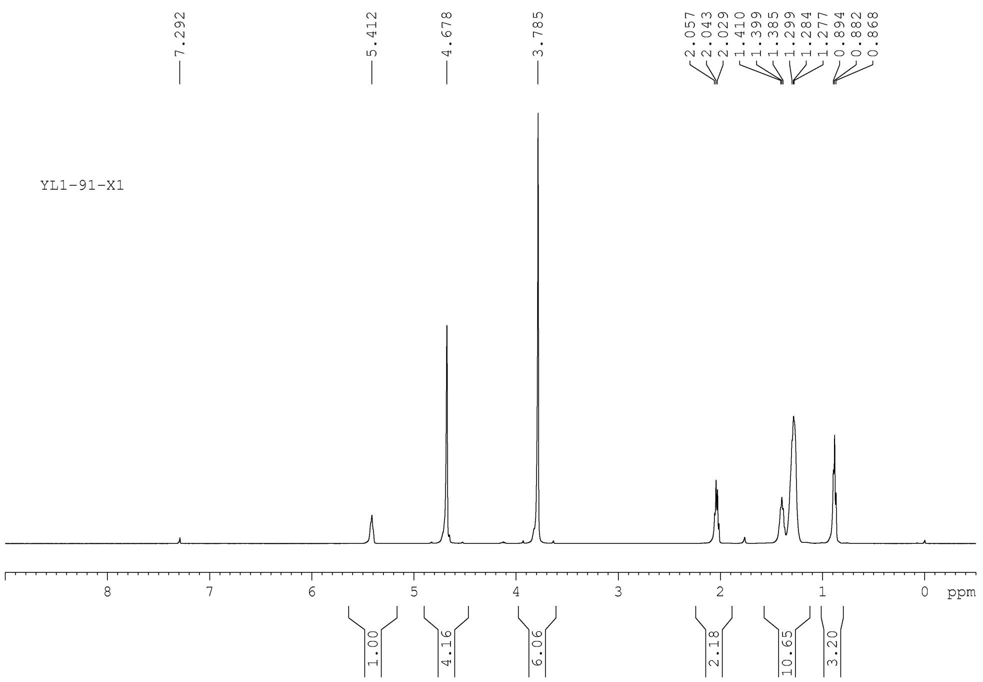

7.292
5.412
4.678
3.785
2.057
2.043
2.029
1.410
1.399
1.385
1.299
1.284
1.277
0.894
0.882
0.868
YL1-91-X1
8
7
6
5
4
3
2
1
0
ppm
1.00
4.16
6.06
2.18
10.65
3.20

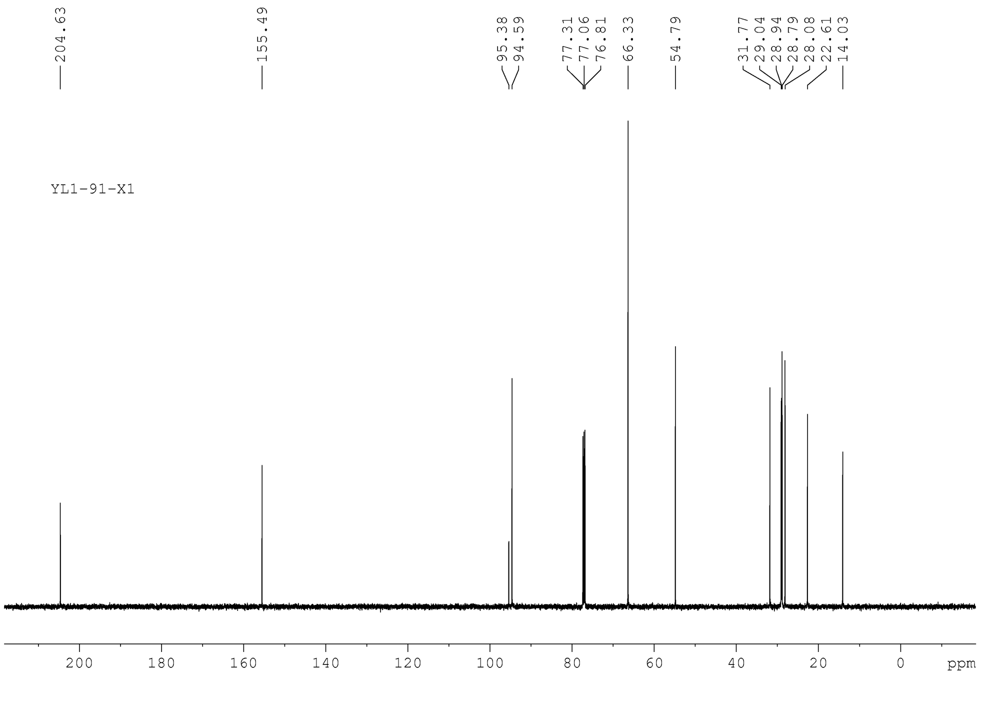

204.63
155.49
95.38
94.59
77.31
77.06
76.81
66.33
54.79
31.77
29.04
28.94
28.79
28.08
22.61
14.03
YL1-91-X1
200
180
160
140
120
100
80
60
40
20
0
ppm

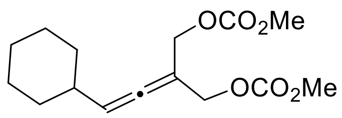
OCO2Me
OCO2Me

1k

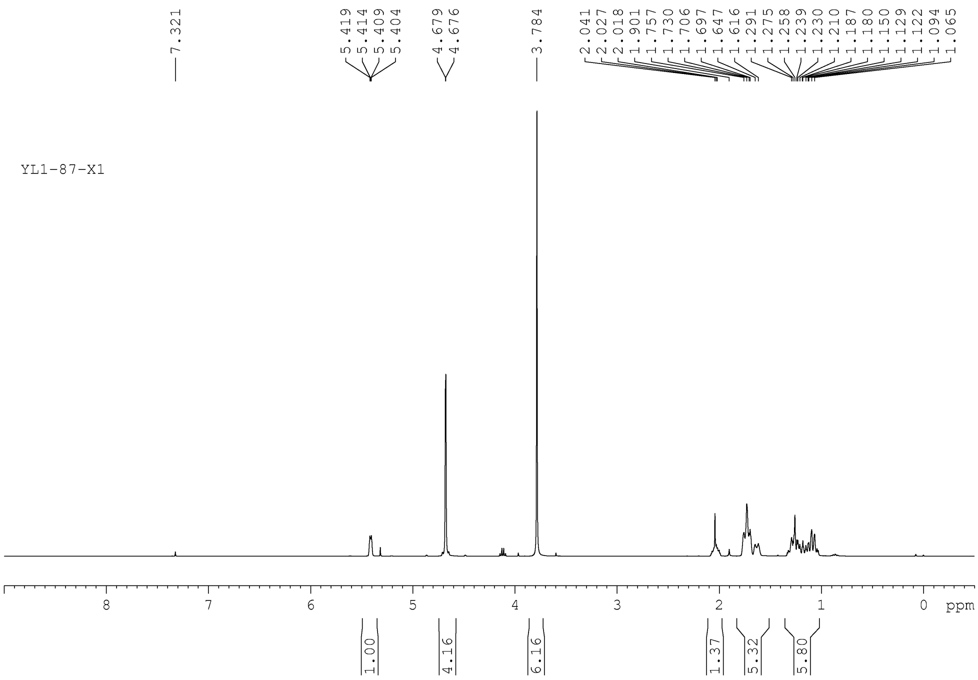
7.321
5.419
5.414
5.409
5.404
4.679
4.676
3.784
2.041
2.027
2.018
1.901
1.757
1.730
1.706
1.697
1.647
1.616
1.291
1.275
1.258
1.239
1.230
1.210
1.187
1.180
1.150
1.129
1.122
1.094
1.065
YL1-87-X1
8
7
6
5
4
3
2
1
0 ppm
1.00
4.16
6.16
1.37
5.32
5.80

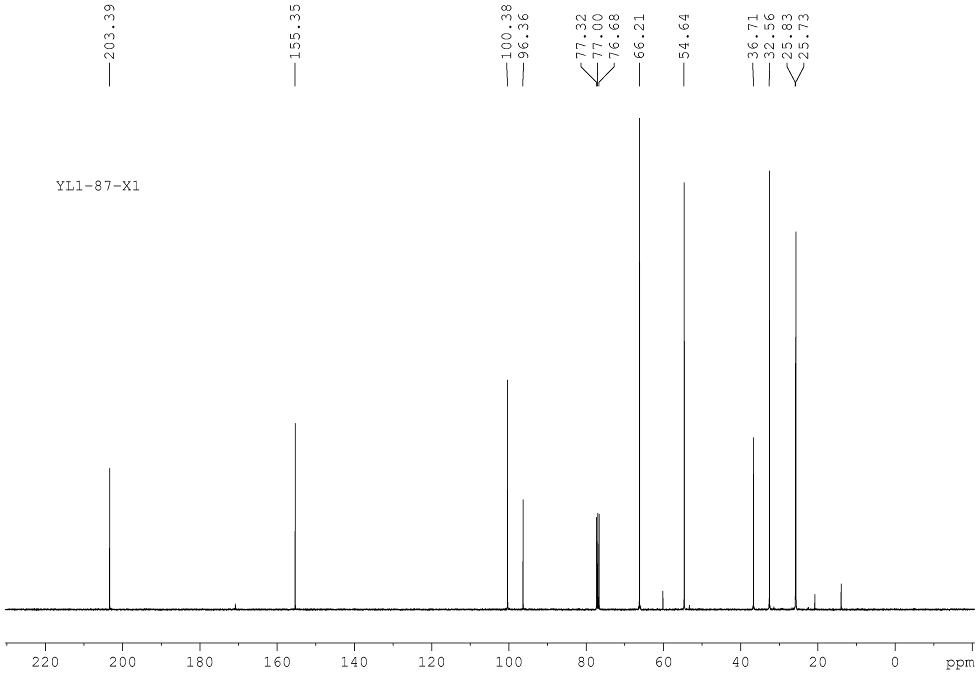
203.39
155.35
100.38
96.36
77.32
77.00
76.68
66.21
54.64
36.71
32.56
25.83
25.73
YL1-87-X1
220
200
180
160
140
120
100
80
60
40
20
0
ppm

NMR spectra of 3

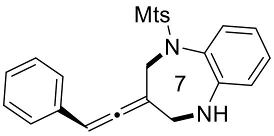

3a

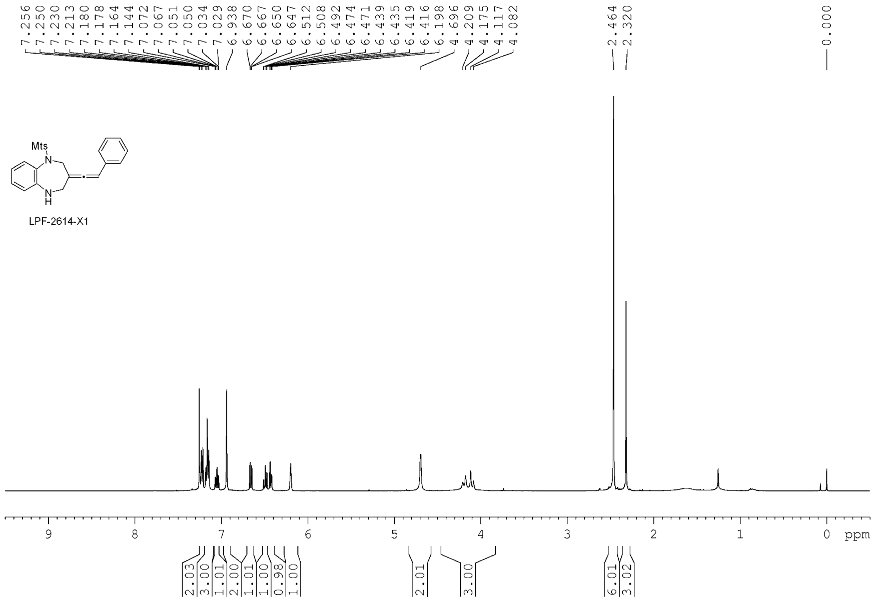

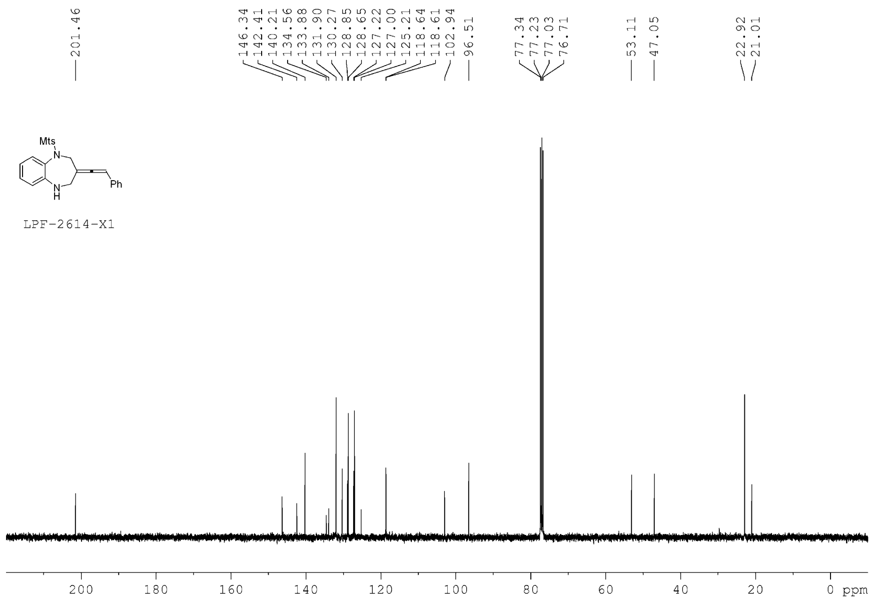

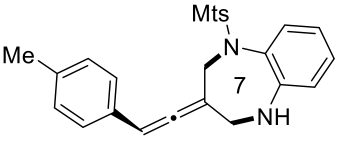
Mts
N
7
NH
Me

3b

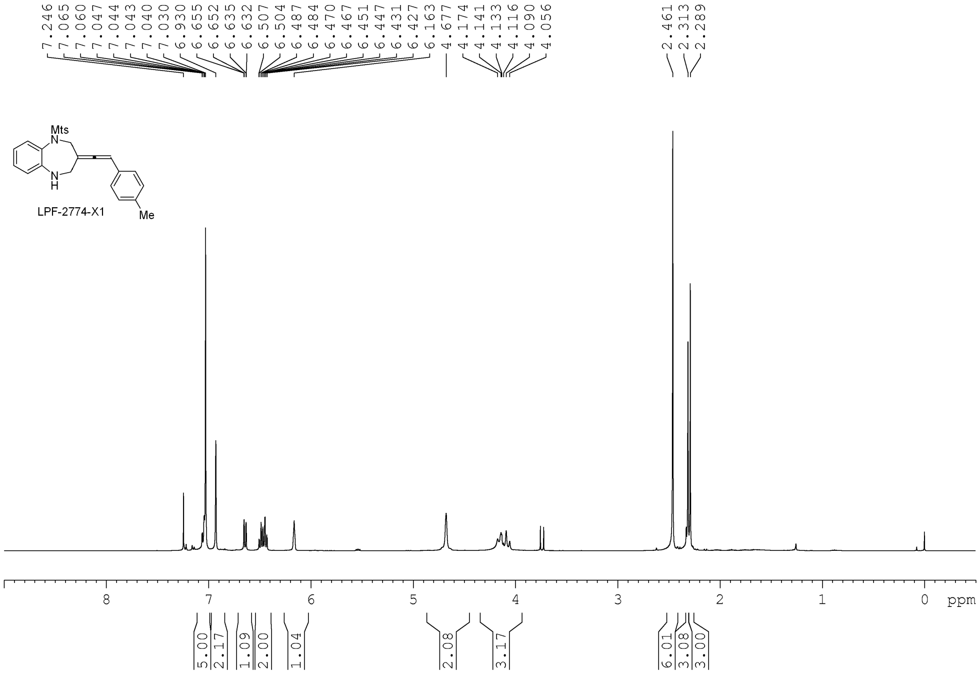
7.246
7.065
7.060
7.047
7.044
7.043
7.040
7.030
6.930
6.655
6.652
6.635
6.632
6.507
6.504
6.487
6.484
6.470
6.467
6.451
6.447
6.431
6.427
6.163
4.677
4.174
4.141
4.133
4.116
4.090
4.056
2.461
2.313
2.289
LPF-2774-X1
8 7 6 5 4 3 2 1 0 ppm
5.00
2.17
1.09
2.00
1.04
2.08
3.17
6.01
3.08
3.00

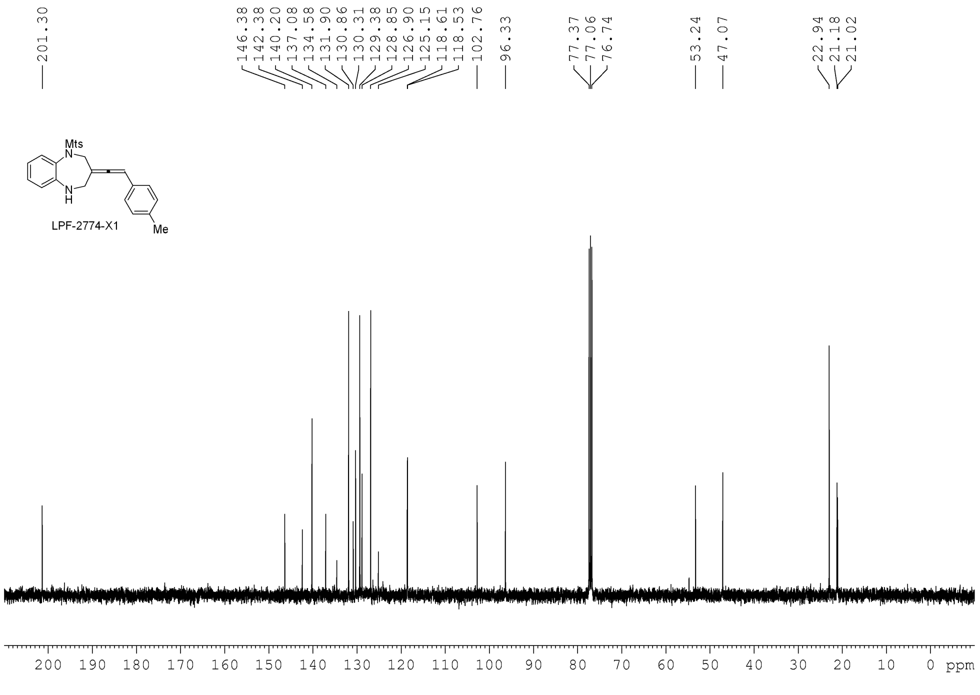
201.30
146.38
142.38
140.20
137.08
134.58
131.90
130.86
130.31
129.38
128.85
126.90
125.15
118.61
118.53
102.76
96.33
77.37
77.06
76.74
53.24
47.07
22.94
21.18
21.02
LPF-2774-X1
200 190 180 170 160 150 140 130 120 110 100 90 80 70 60 50 40 30 20 10 0 ppm

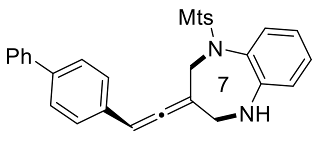
Mts
N
7
NH
Ph

3c

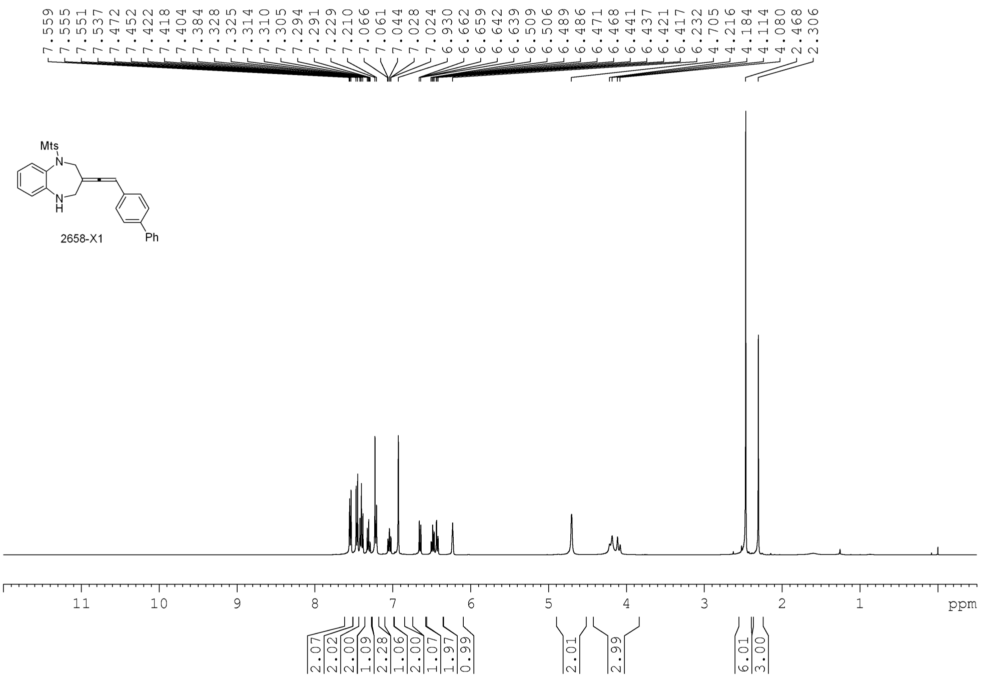
7.559
7.555
7.551
7.537
7.472
7.452
7.422
7.418
7.404
7.384
7.328
7.325
7.314
7.310
7.305
7.294
7.291
7.229
7.210
7.066
7.061
7.044
7.028
7.024
6.930
6.662
6.659
6.642
6.639
6.509
6.506
6.489
6.486
6.471
6.468
6.441
6.437
6.421
6.417
6.232
4.705
4.216
4.184
4.114
4.080
2.468
2.306
2658-X1
Mts
N
H
Ph
11 10 9 8 7 6 5 4 3 2 1 ppm
2.07
2.02
2.00
1.09
2.28
1.06
2.00
1.07
1.97
0.99
2.01
2.99
6.01
3.00

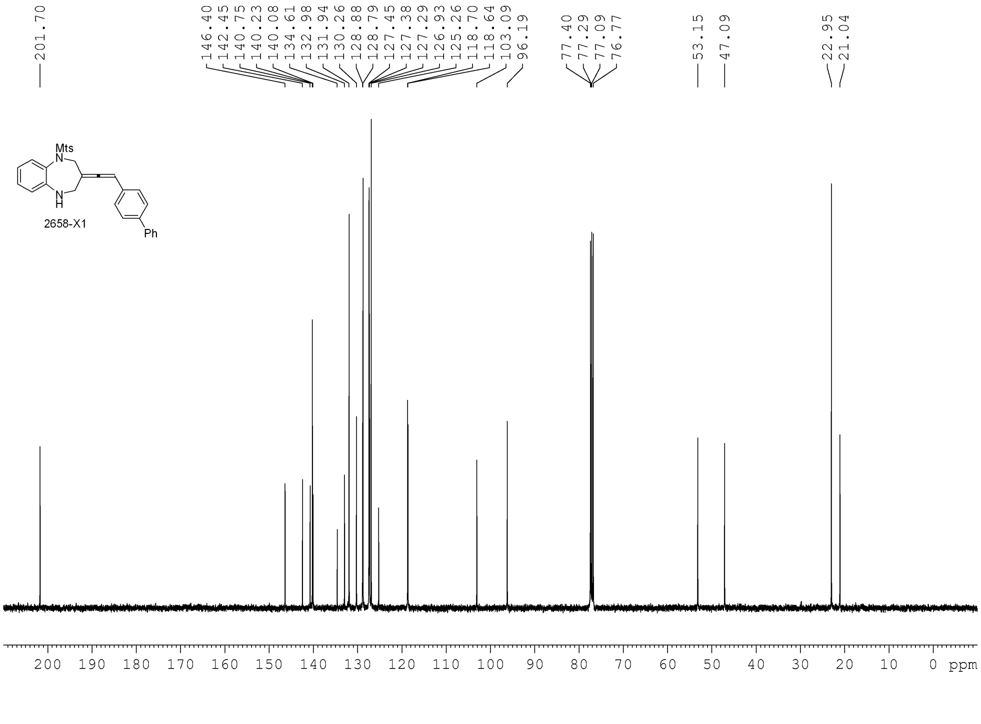
201.70
146.40
142.45
140.75
140.23
140.08
134.61
132.98
131.94
130.26
128.88
128.79
127.45
127.38
127.29
126.93
125.26
118.70
118.64
103.09
96.19
77.40
77.29
77.09
76.77
53.15
47.09
22.95
21.04
2658-X1
Mts
N
H
Ph
200 190 180 170 160 150 140 130 120 110 100 90 80 70 60 50 40 30 20 10 0 ppm

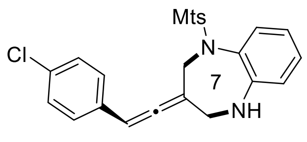
Mts
N
Cl
7
NH

3d

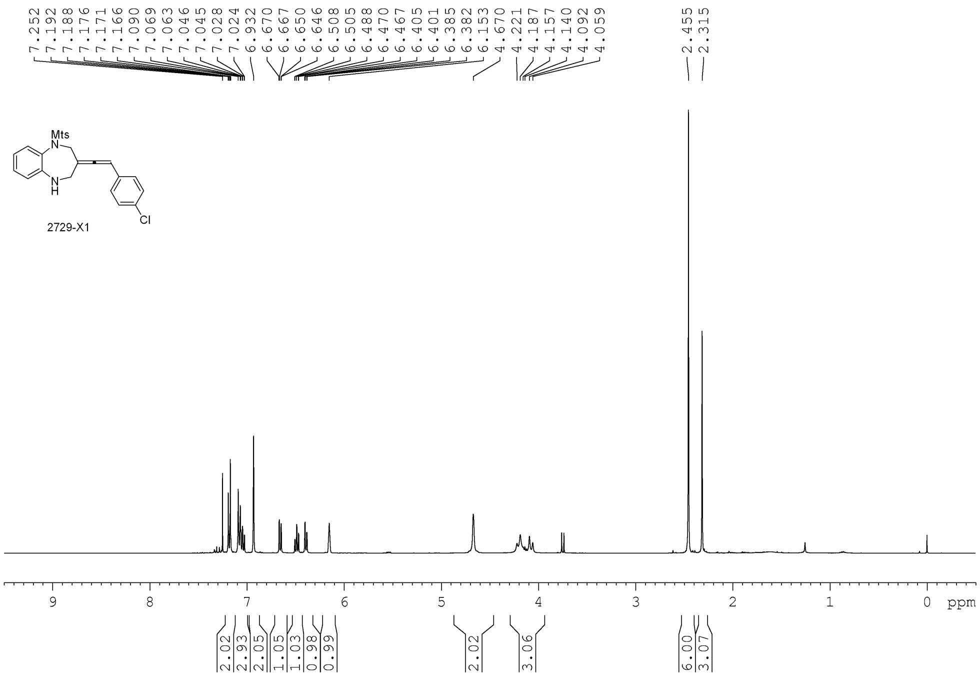
7.252
7.192
7.188
7.176
7.171
7.166
7.090
7.069
7.063
7.046
7.045
7.028
7.024
6.932
6.670
6.667
6.650
6.646
6.508
6.505
6.488
6.470
6.467
6.405
6.401
6.385
6.382
6.153
4.670
4.221
4.187
4.157
4.140
4.092
4.059
2.455
2.315
Mts
N
N
H
Cl
2729-X1
9 8 7 6 5 4 3 2 1 0 ppm
2.02
2.93
2.05
1.05
1.03
0.98
0.99
2.02
3.06
6.00
3.07

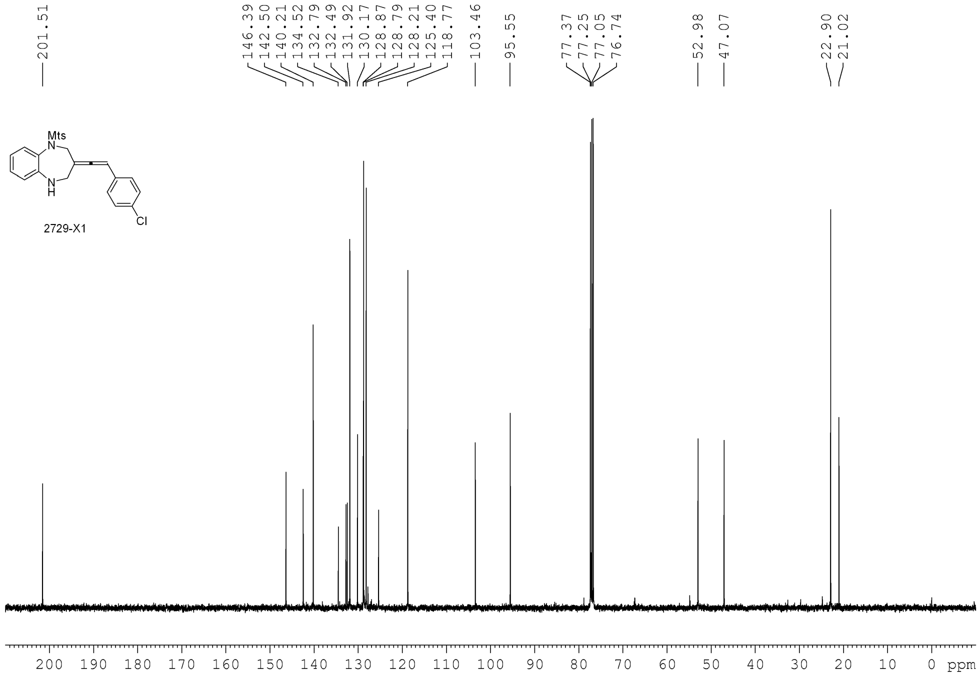
201.51
146.39
142.50
140.21
134.52
132.79
132.49
131.92
130.17
128.87
128.79
128.21
125.40
118.77
103.46
95.55
77.37
77.25
77.05
76.74
52.98
47.07
22.90
21.02
Mts
N
N
H
Cl
2729-X1
200 190 180 170 160 150 140 130 120 110 100 90 80 70 60 50 40 30 20 10 0 ppm

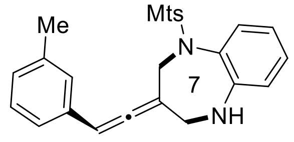
Me
Mts
N
7
NH

3e

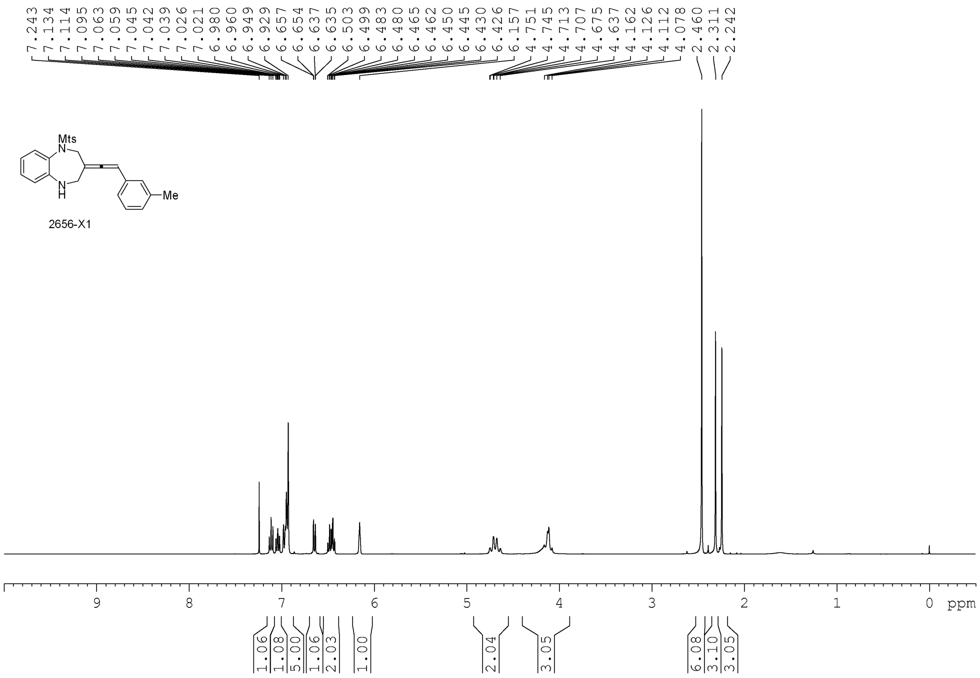
7.243
7.134
7.114
7.095
7.063
7.059
7.045
7.042
7.039
7.026
7.021
6.980
6.960
6.949
6.929
6.657
6.654
6.637
6.635
6.503
6.499
6.483
6.480
6.465
6.462
6.450
6.445
6.430
6.426
6.157
4.751
4.745
4.713
4.707
4.675
4.637
4.162
4.126
4.112
4.078
2.460
2.311
2.242
Mts
N
N
H
Me
2656-X1
9
8
7
6
5
4
3
2
1
0 ppm
1.06
1.08
5.00
1.06
2.03
1.00
2.04
3.05
6.08
3.10
3.05

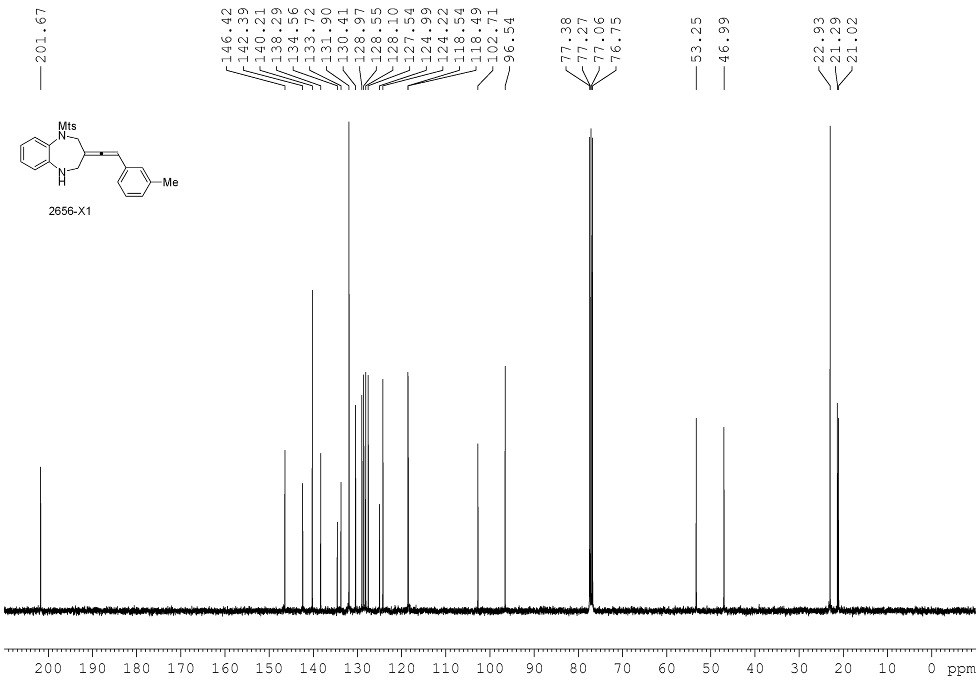
201.67
146.42
142.39
140.21
138.29
134.56
133.72
131.90
130.41
128.97
128.55
128.10
127.54
124.99
124.22
118.54
118.49
102.71
96.54
77.38
77.27
77.06
76.75
53.25
46.99
22.93
21.29
21.02
Mts
N
N
H
Me
2656-X1
200
190
180
170
160
150
140
130
120
110
100
90
80
70
60
50
40
30
20
10
0 ppm

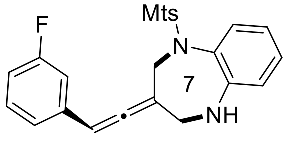
F
Mts
N
7
NH

3f

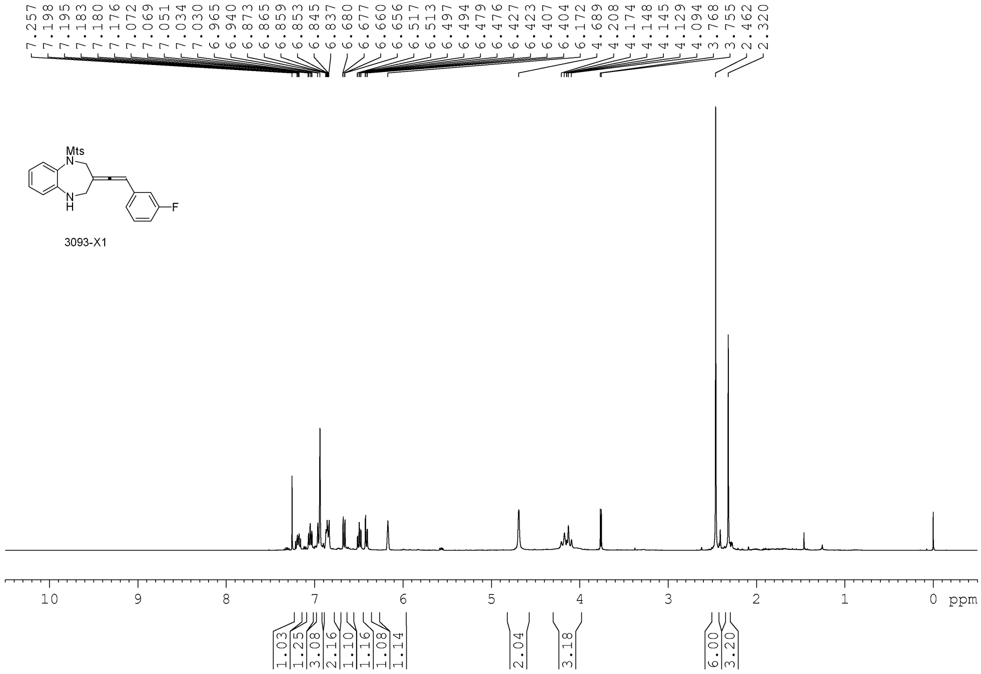
7.257 7.198 7.195 7.183 7.180 7.176 7.072 7.069 7.051 7.034 7.030 6.965 6.940 6.873 6.865 6.859 6.853 6.845 6.837 6.680 6.677 6.660 6.656 6.517 6.513 6.497 6.494 6.479 6.476 6.427 6.423 6.407 6.404 6.172 4.689 4.208 4.174 4.148 4.145 4.129 4.094 3.768 3.755 2.462 2.320
Mts
N
H
F
3093-X1
10 9 8 7 6 5 4 3 2 1 0 ppm
1.03 1.25 3.08 2.16 1.10 1.16 1.08 1.14 2.04 3.18 6.00 3.20

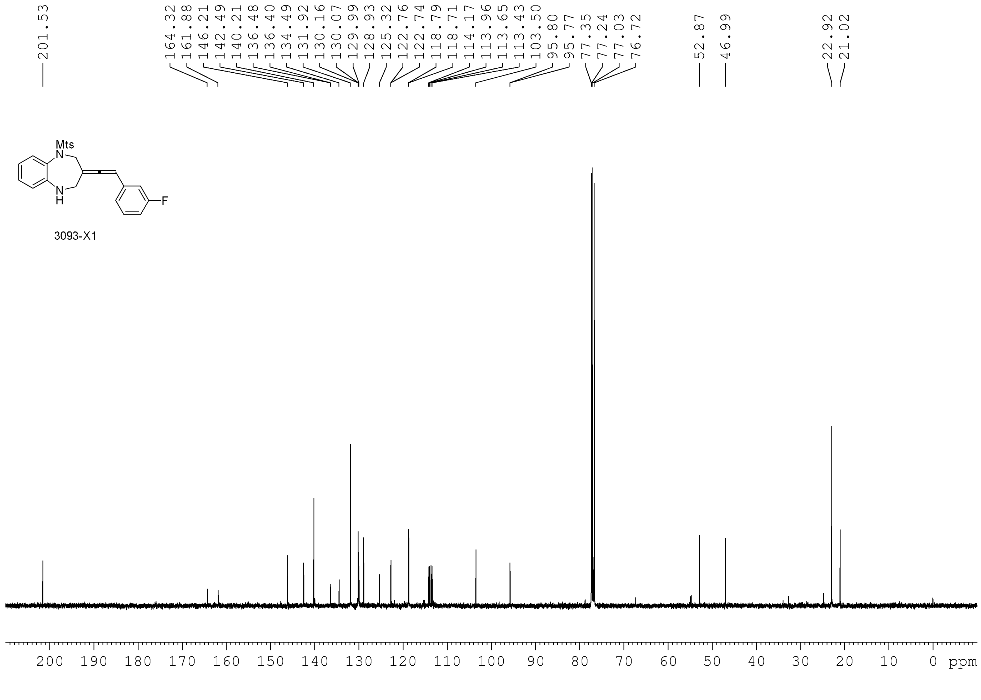
201.53
164.32 161.88 146.21 142.49 140.21 136.48 136.40 134.49 131.92 130.16 130.07 129.99 128.93 125.32 122.76 122.74 118.79 118.71 114.17 113.96 113.65 113.43 103.50 95.80 95.77 77.35 77.24 77.03 76.72
52.87 46.99
22.92 21.02
Mts
N
H
F
3093-X1
200 190 180 170 160 150 140 130 120 110 100 90 80 70 60 50 40 30 20 10 0 ppm

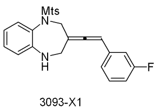

3093-X1

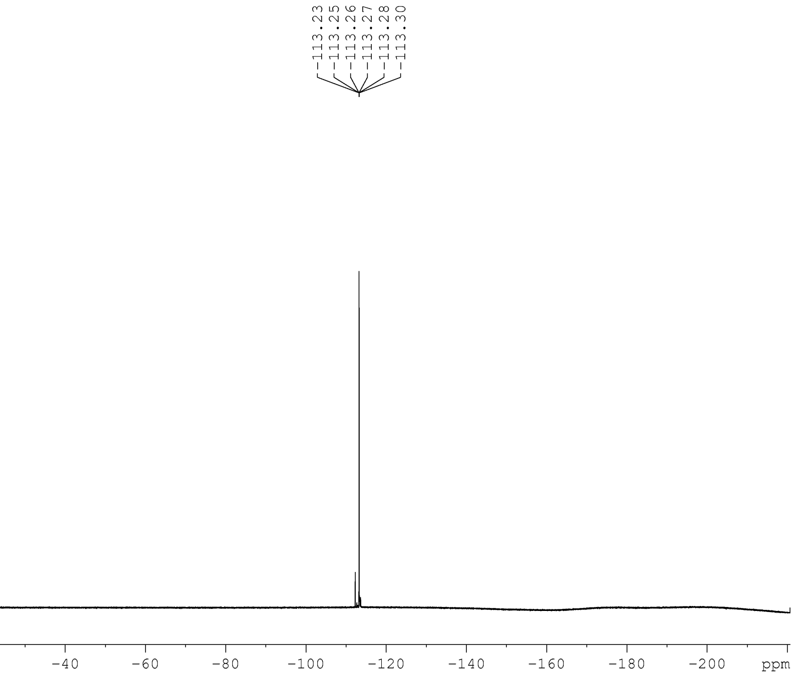
-113.23
-113.25
-113.26
-113.27
-113.28
-113.30
0
-20
-40
-60
-80
-100
-120
-140
-160
-180
-200
ppm

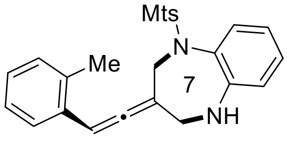

Mts
Me
N
7
NH

3g

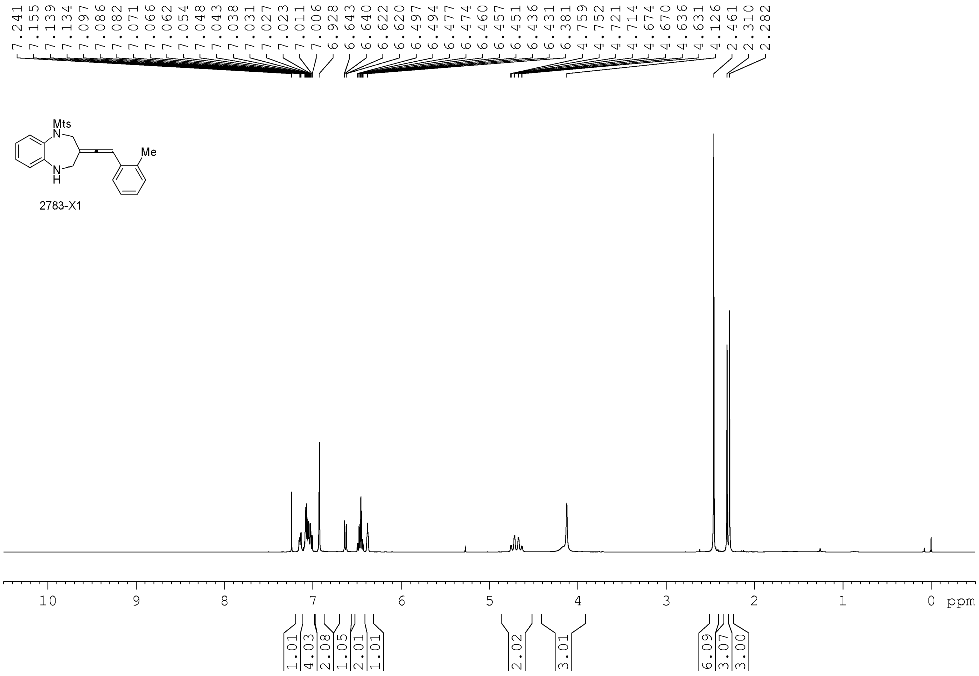

7.241
7.155
7.139
7.134
7.097
7.086
7.082
7.071
7.066
7.062
7.054
7.048
7.043
7.038
7.031
7.027
7.023
7.011
7.006
6.928
6.643
6.640
6.622
6.620
6.497
6.494
6.477
6.474
6.460
6.457
6.451
6.436
6.431
6.381
4.759
4.752
4.721
4.714
4.674
4.670
4.636
4.631
4.126
2.461
2.310
2.282
Mts
N
H
Me
2783-X1
10
9
8
7
6
5
4
3
2
1
0 ppm
1.01
4.03
2.08
1.05
2.01
1.01
2.02
3.01
6.09
3.07
3.00

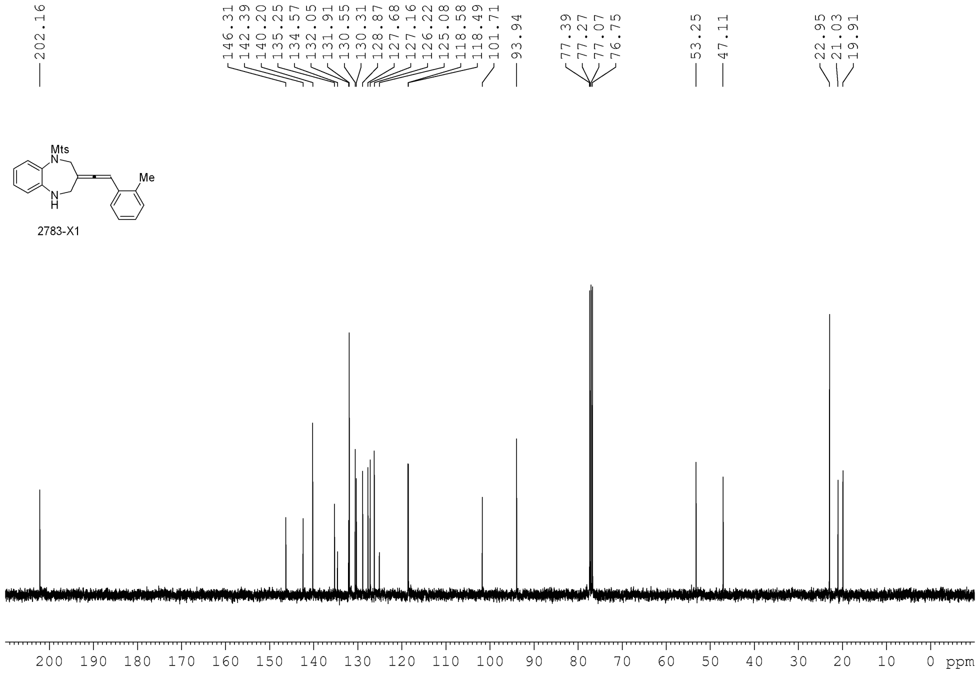

202.16
146.31
142.39
140.20
135.25
134.57
132.05
131.91
130.55
130.31
128.87
127.68
127.16
126.22
125.08
118.58
118.49
101.71
93.94
77.39
77.27
77.07
76.75
53.25
47.11
22.95
21.03
19.91
Mts
N
H
Me
2783-X1
200
190
180
170
160
150
140
130
120
110
100
90
80
70
60
50
40
30
20
10
0 ppm

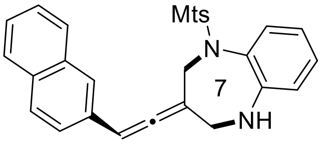

Mts
N
7
NH

3h

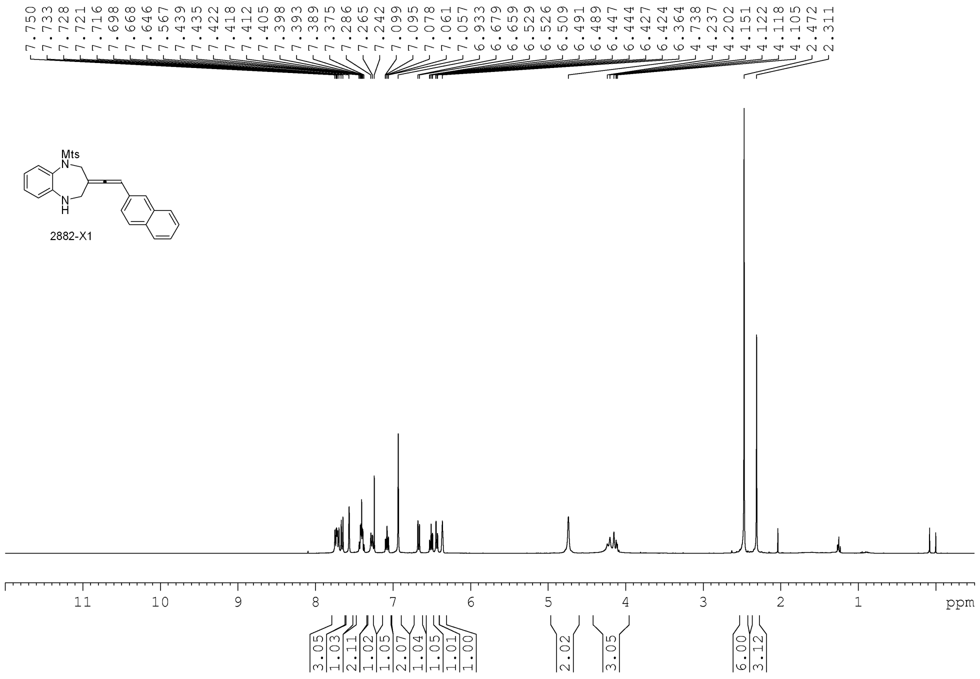

7.750
7.733
7.728
7.721
7.716
7.698
7.646
7.567
7.439
7.435
7.422
7.418
7.412
7.405
7.398
7.393
7.389
7.375
7.286
7.265
7.242
7.099
7.095
7.078
7.061
7.057
6.933
6.679
6.659
6.529
6.526
6.509
6.491
6.489
6.447
6.444
6.427
6.424
6.364
4.738
4.237
4.202
4.151
4.122
4.118
4.105
2.472
2.311
Mts
N
H
2882-X1
11 10 9 8 7 6 5 4 3 2 1 ppm
3.05
1.03
2.11
1.02
1.05
2.07
1.04
1.05
1.01
1.00
2.02
3.05
6.00
3.12

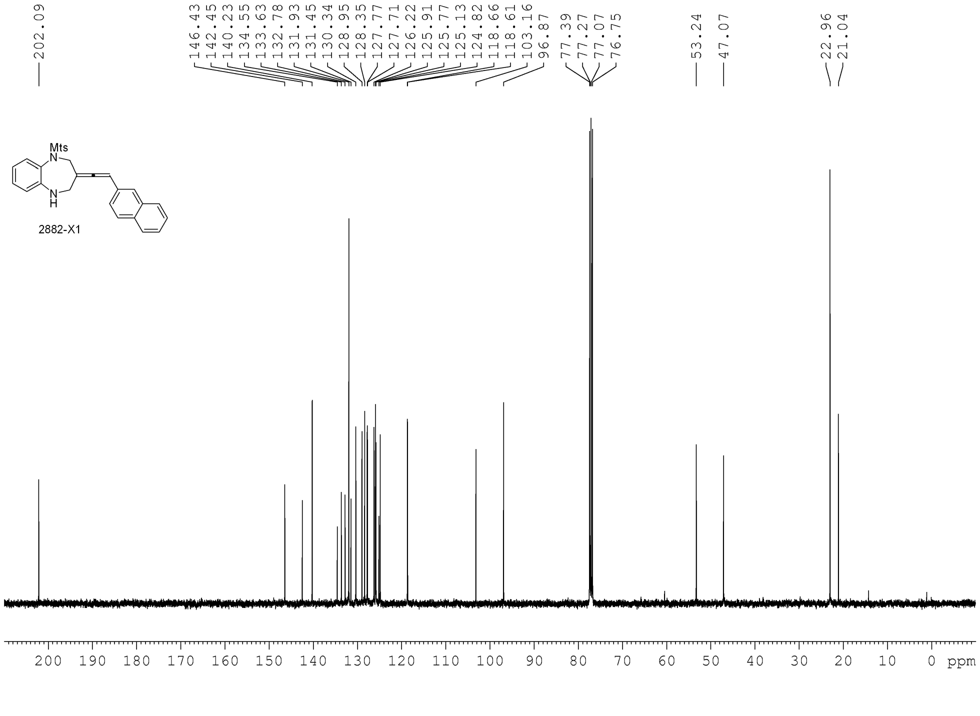

202.09
146.43
142.45
140.23
134.55
133.63
132.78
131.93
131.45
130.34
128.95
128.35
127.77
127.71
126.22
125.91
125.77
125.13
124.82
118.66
118.61
103.16
96.87
77.39
77.27
77.07
76.75
53.24
47.07
22.96
21.04
Mts
N
H
2882-X1
200 190 180 170 160 150 140 130 120 110 100 90 80 70 60 50 40 30 20 10 0 ppm

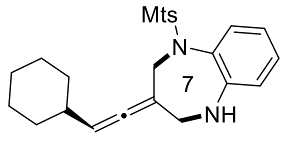
Mts
N
7
NH

3i

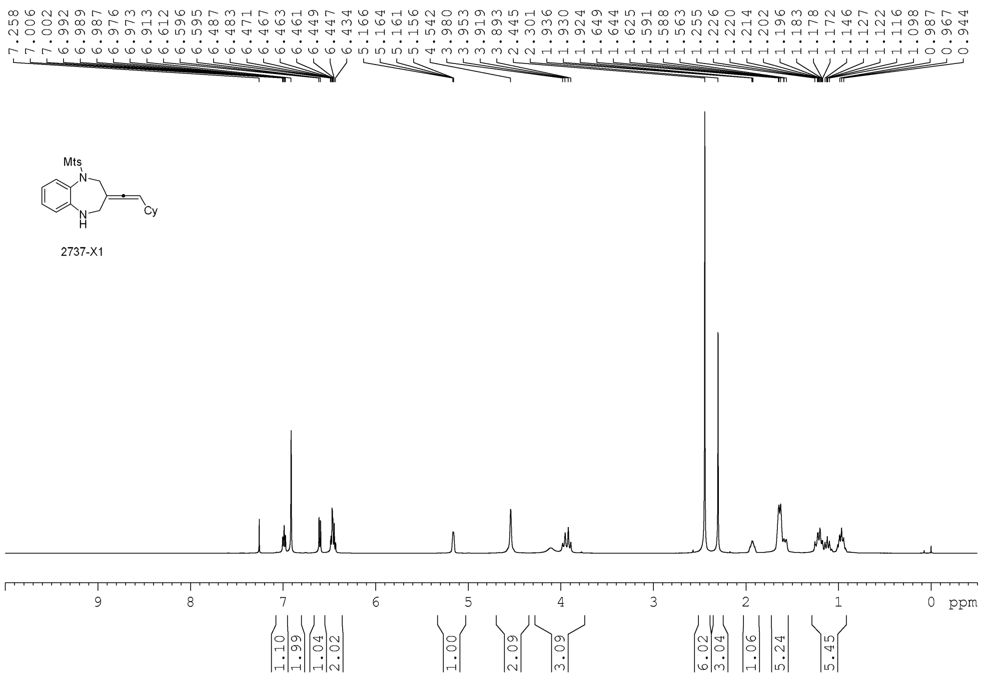
7.258
7.006
6.992
6.989
6.987
6.976
6.973
6.913
6.612
6.596
6.595
6.487
6.483
6.471
6.467
6.463
6.461
6.449
6.447
6.434
5.166
5.164
5.161
5.156
4.542
3.980
3.953
3.919
3.893
2.445
2.301
1.936
1.930
1.924
1.649
1.644
1.625
1.591
1.588
1.563
1.255
1.226
1.220
1.214
1.202
1.196
1.183
1.178
1.172
1.146
1.127
1.122
1.116
1.098
0.987
0.967
0.944
Mts
N
N
H
Cy
2737-X1
9
8
7
6
5
4
3
2
1
0 ppm
1.10
1.99
1.04
2.02
1.00
2.09
3.09
6.02
3.04
1.06
5.24
5.45

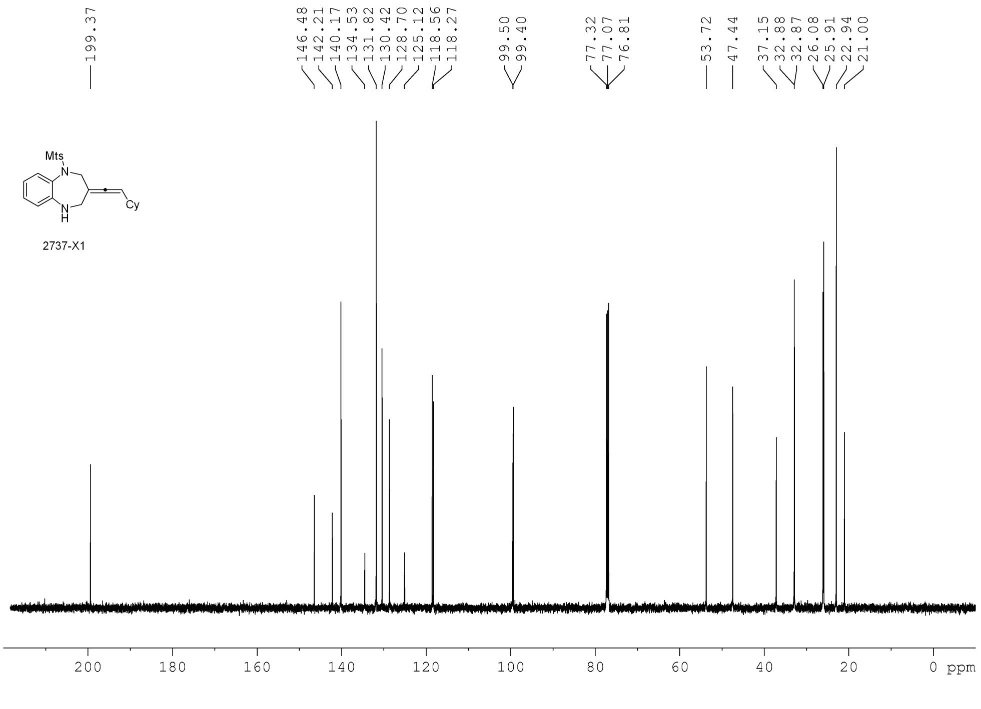
199.37
146.48
142.21
140.17
134.53
131.82
130.42
128.70
125.12
118.56
118.27
99.50
99.40
77.32
77.07
76.81
53.72
47.44
37.15
32.88
32.87
26.08
25.91
22.94
21.00
Mts
N
N
H
Cy
2737-X1
200
180
160
140
120
100
80
60
40
20
0 ppm

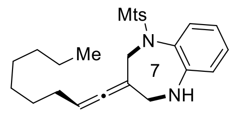
Mts
N
Me
7
NH

3j

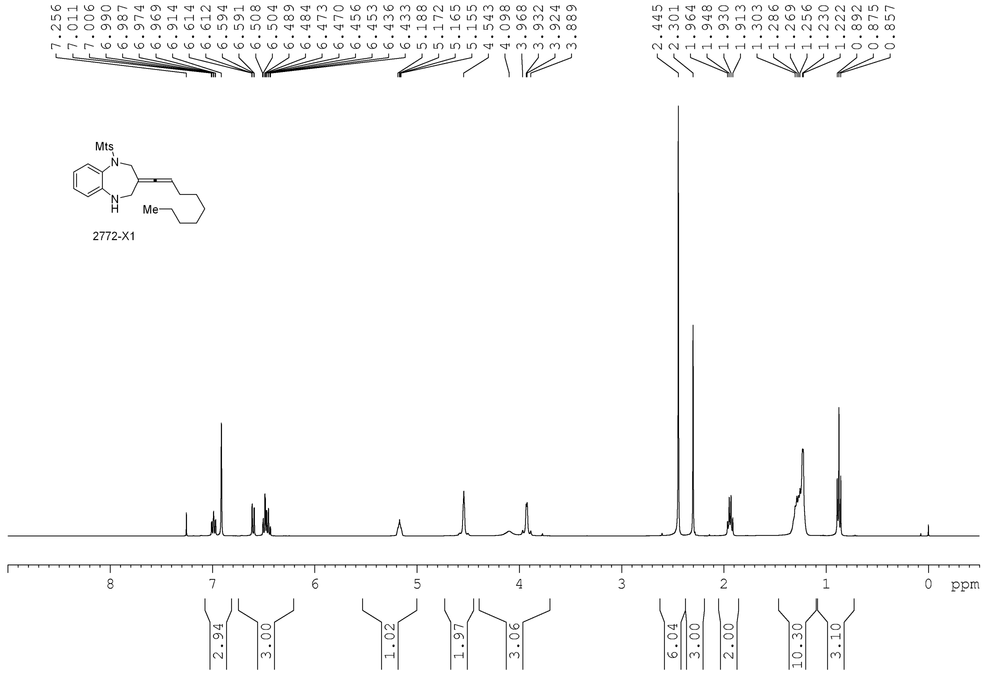
7.256
7.011
7.006
6.990
6.987
6.974
6.969
6.914
6.614
6.612
6.594
6.591
6.508
6.504
6.489
6.484
6.473
6.470
6.456
6.453
6.436
6.433
5.188
5.172
5.165
5.155
4.543
4.098
3.968
3.932
3.924
3.889
2.445
2.301
1.964
1.948
1.930
1.913
1.303
1.286
1.269
1.256
1.230
1.222
0.892
0.875
0.857
Mts
N
H
Me
2772-X1
8
7
6
5
4
3
2
1
0 ppm
2.94
3.00
1.02
1.97
3.06
6.04
3.00
2.00
10.30
3.10

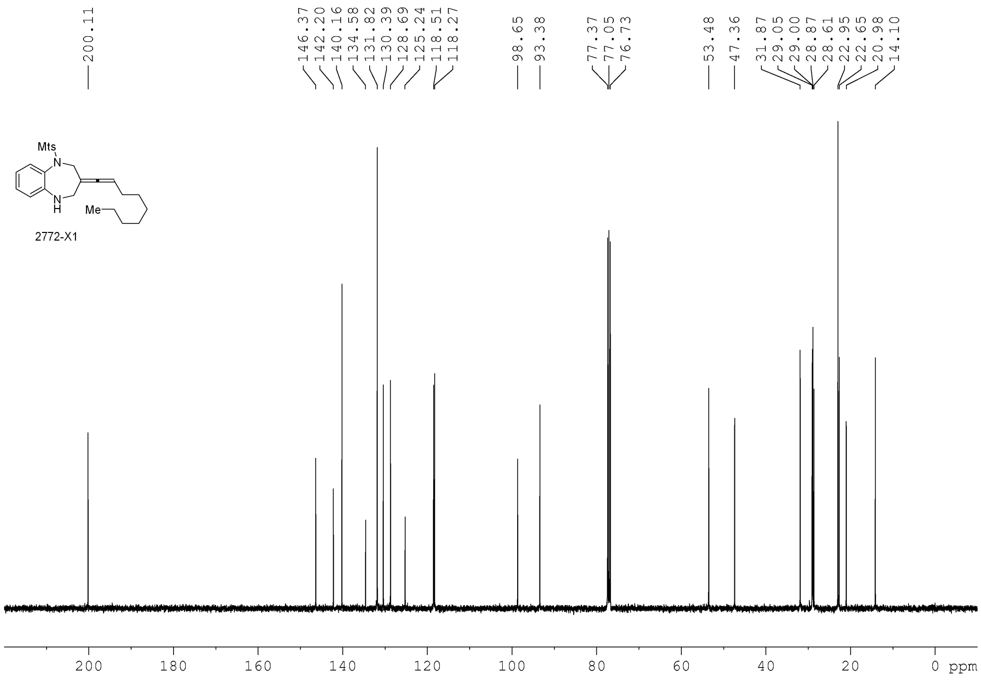
200.11
146.37
142.20
140.16
134.58
131.82
130.39
128.69
125.24
118.51
118.27
98.65
93.38
77.37
77.05
76.73
53.48
47.36
31.87
29.05
29.00
28.87
28.61
22.95
22.65
20.98
14.10
Mts
N
H
Me
2772-X1
200
180
160
140
120
100
80
60
40
20
0 ppm

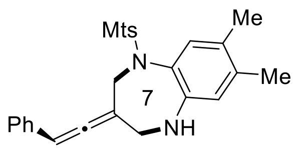
Mts
N
Me
Me
7
Ph
NH

3k

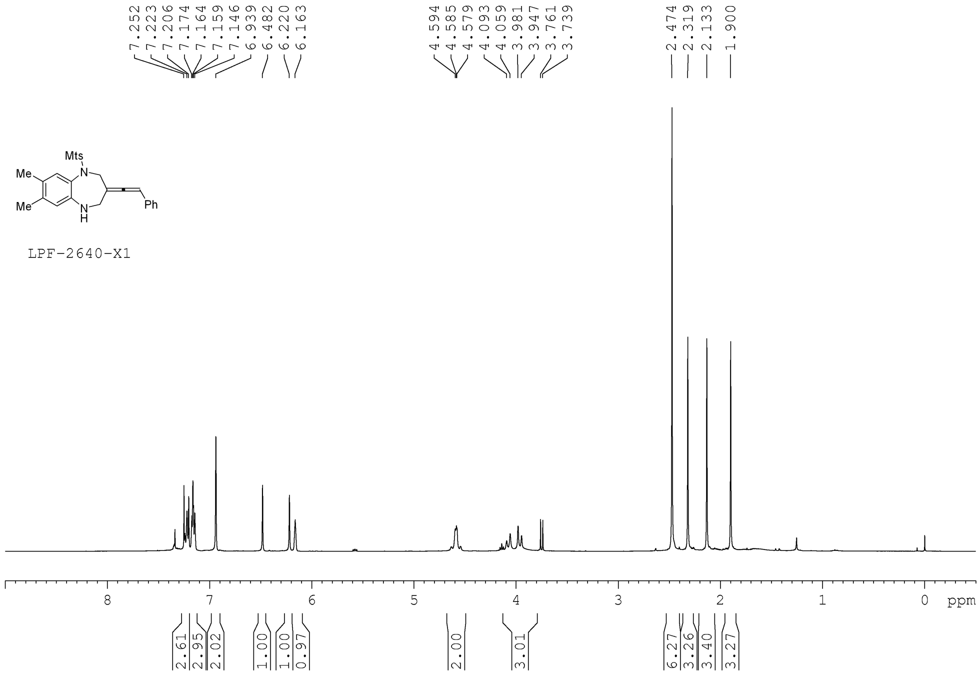
7.252
7.223
7.206
7.174
7.164
7.159
7.146
6.939
6.482
6.220
6.163
4.594
4.585
4.579
4.093
4.059
3.981
3.947
3.761
3.739
2.474
2.319
2.133
1.900
Mts
Me
Me
N
H
Ph
LPF-2640-X1
8
7
6
5
4
3
2
1
0 ppm
2.61
2.95
2.02
1.00
1.00
0.97
2.00
3.01
6.27
3.26
3.40
3.27

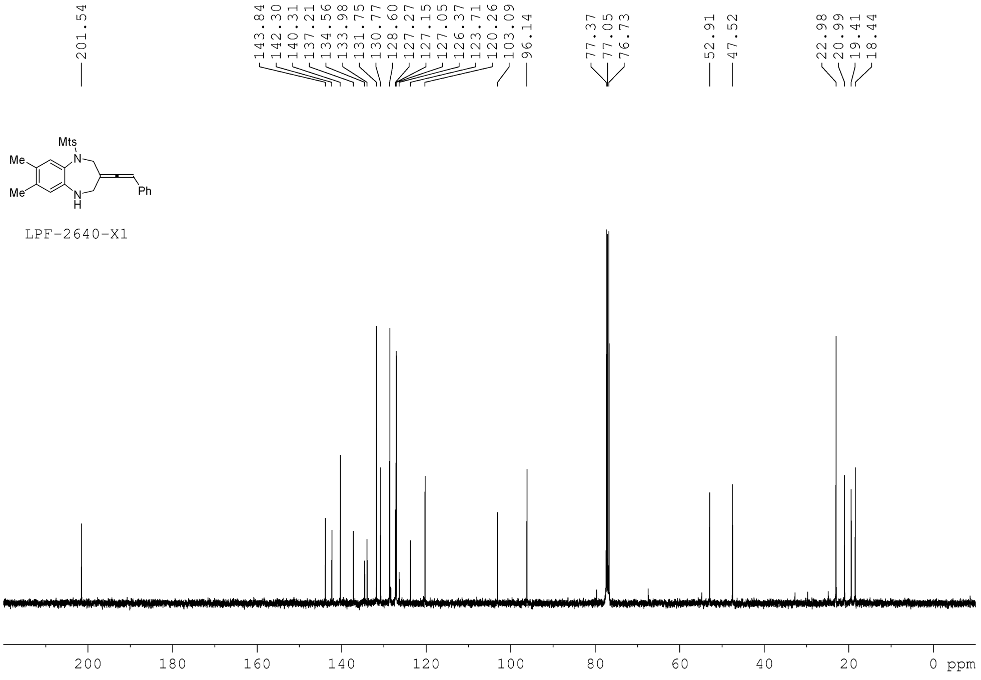
201.54
143.84
142.30
140.31
137.21
134.56
133.98
131.75
130.77
128.60
127.27
127.15
127.05
126.37
123.71
120.26
103.09
96.14
77.37
77.05
76.73
52.91
47.52
22.98
20.99
19.41
18.44
Mts
Me
Me
N
H
Ph
LPF-2640-X1
200
180
160
140
120
100
80
60
40
20
0 ppm

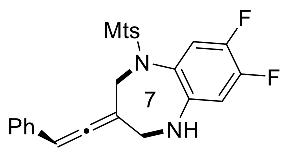

3l

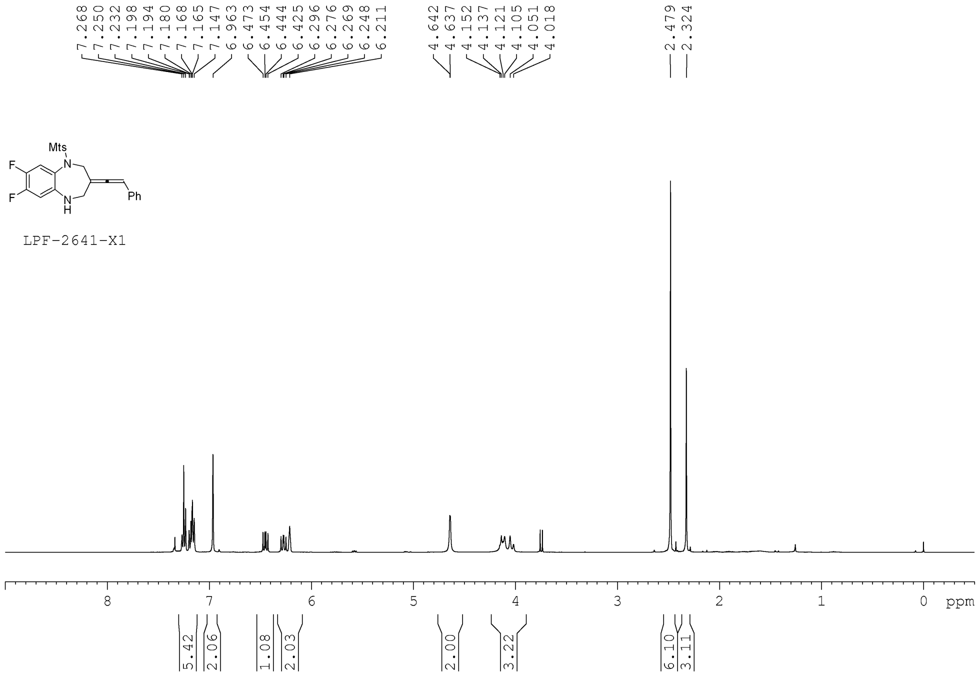

7.268
7.250
7.232
7.198
7.194
7.180
7.168
7.165
7.147
6.963
6.473
6.454
6.444
6.425
6.296
6.276
6.269
6.248
6.211
4.642
4.637
4.152
4.137
4.121
4.105
4.051
4.018
2.479
2.324
LPF-2641-X1
5.42
2.06
1.08
2.03
2.00
3.22
6.10
3.11
ppm

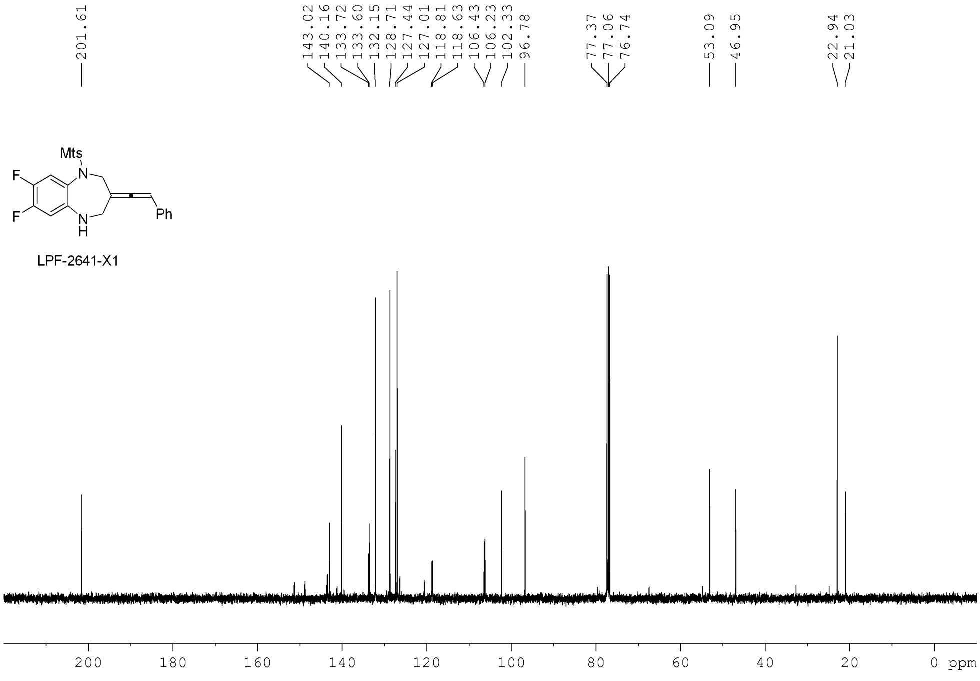

201.61
143.02
140.16
133.72
133.60
132.15
128.71
127.44
127.01
118.81
118.63
106.43
106.23
102.33
96.78
77.37
77.06
76.74
53.09
46.95
22.94
21.03
LPF-2641-X1
ppm

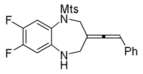

Mts
F
F
N
H
Ph

2641-X1

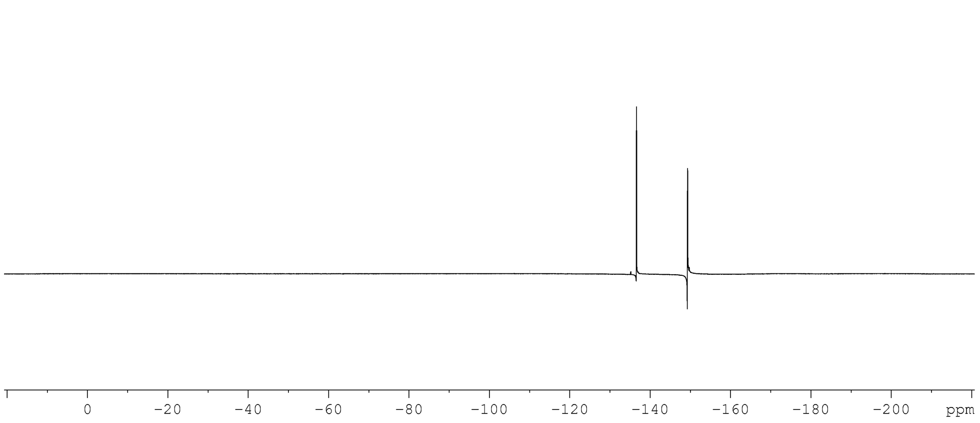

-136.57
-136.59
-136.60
-136.62
-136.63
-136.65
-136.66
-136.68
-149.29
-149.31
-149.33
-149.35
-149.36
-149.38
0
-20
-40
-60
-80
-100
-120
-140
-160
-180
-200
ppm

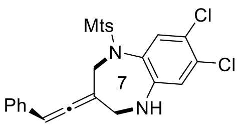

Mts
Cl
Cl
7
NH
Ph

3m

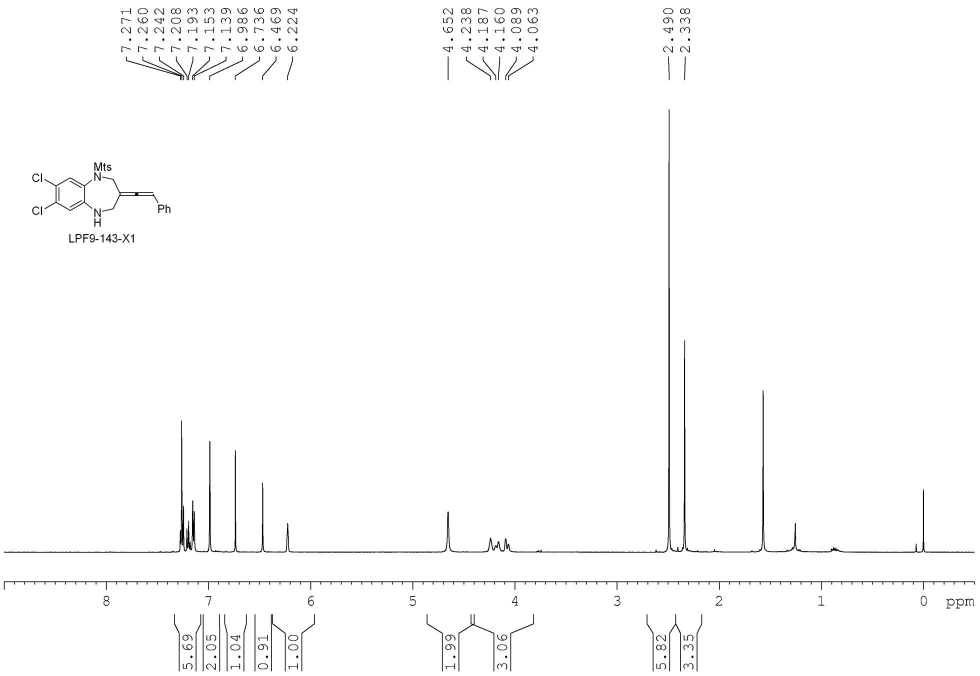

7.271
7.260
7.242
7.208
7.193
7.153
7.139
6.986
6.736
6.469
6.224
4.652
4.238
4.187
4.160
4.089
4.063
2.490
2.338
LPF9-143-X1
5.69
2.05
1.04
0.91
1.00
1.99
3.06
5.82
3.35
ppm

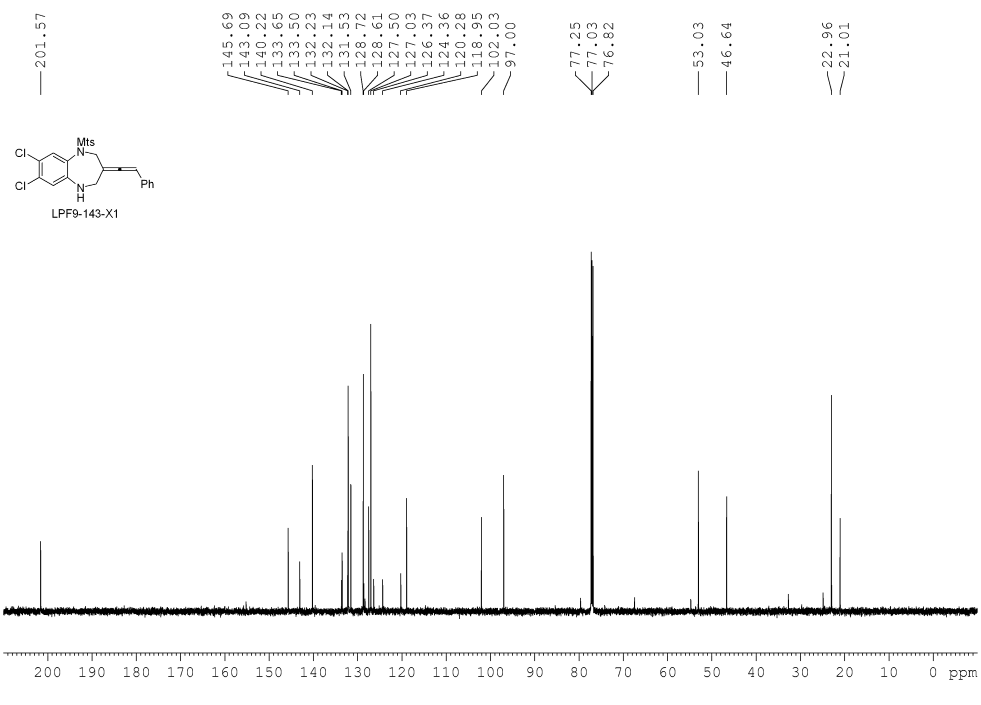

201.57
145.69
143.09
140.22
133.65
133.50
132.23
132.14
131.53
128.72
128.61
127.50
127.03
126.37
124.36
120.28
118.95
102.03
97.00
77.25
77.03
76.82
53.03
46.64
22.96
21.01
LPF9-143-X1
ppm

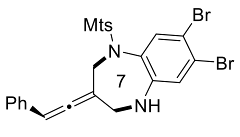
Mts
Br
Br
7
Ph
NH
N
3n

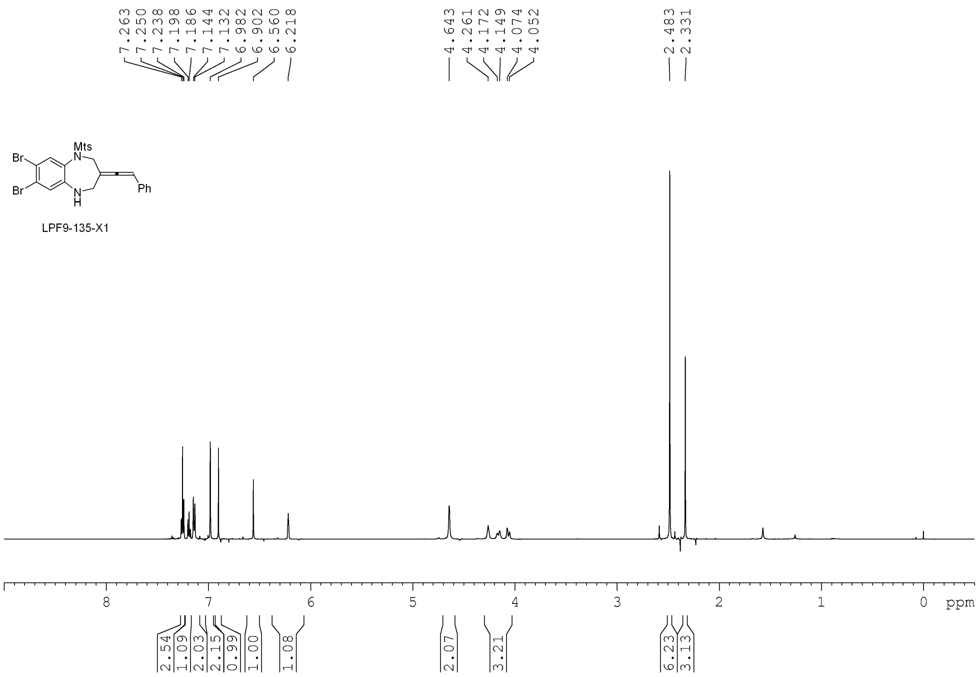
7.263
7.250
7.238
7.198
7.186
7.144
7.132
6.982
6.902
6.560
6.218
4.643
4.261
4.172
4.149
4.074
4.052
2.483
2.331
LPF9-135-X1
2.54
1.09
2.03
2.15
0.99
1.00
1.08
2.07
3.21
6.23
3.13
8
7
6
5
4
3
2
1
0 ppm

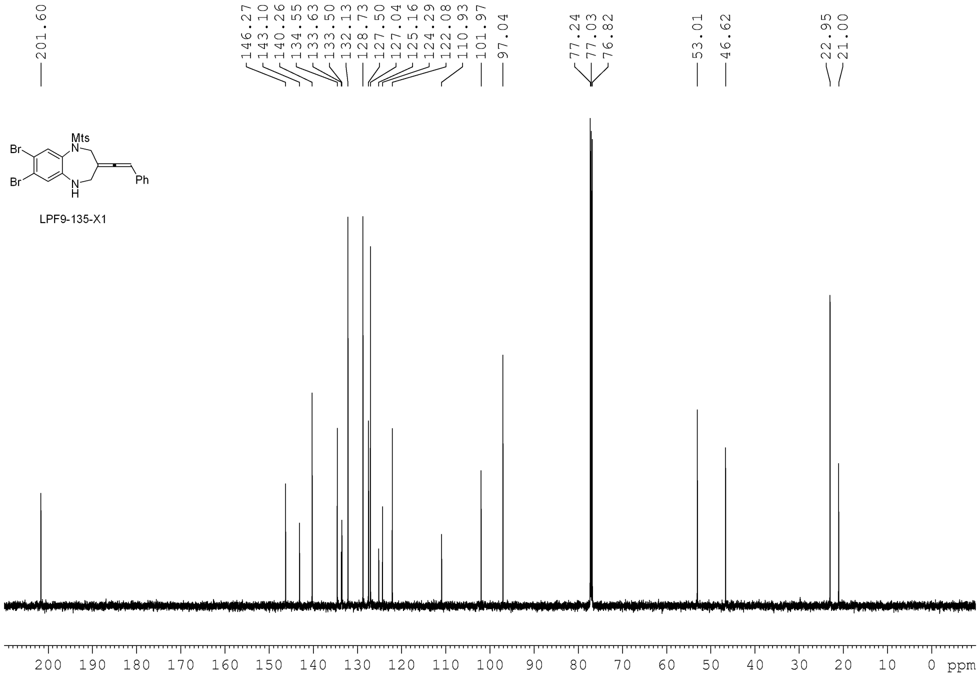
201.60
146.27
143.10
140.26
134.55
133.63
133.50
132.13
128.73
127.50
127.04
125.16
124.29
122.08
110.93
101.97
97.04
77.24
77.03
76.82
53.01
46.62
22.95
21.00
LPF9-135-X1
200
190
180
170
160
150
140
130
120
110
100
90
80
70
60
50
40
30
20
10
0 ppm

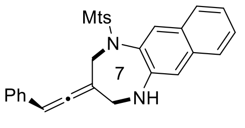

Mts
N
7
NH
Ph

3o

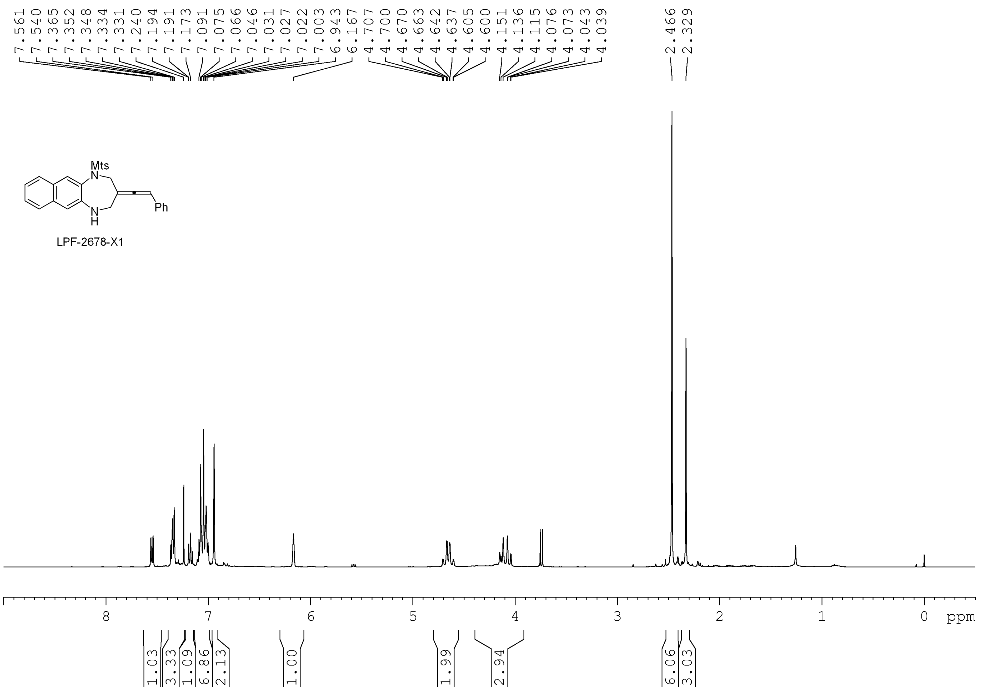

7.561
7.540
7.365
7.352
7.348
7.334
7.331
7.240
7.194
7.191
7.173
7.091
7.075
7.066
7.046
7.031
7.027
7.022
7.003
6.943
6.167
4.707
4.700
4.670
4.663
4.642
4.637
4.605
4.600
4.151
4.136
4.115
4.076
4.073
4.043
4.039
2.466
2.329
Mts
N
H
Ph
LPF-2678-X1
8
7
6
5
4
3
2
1
0 ppm
1.03
3.33
1.09
6.86
2.13
1.00
1.99
2.94
6.06
3.03

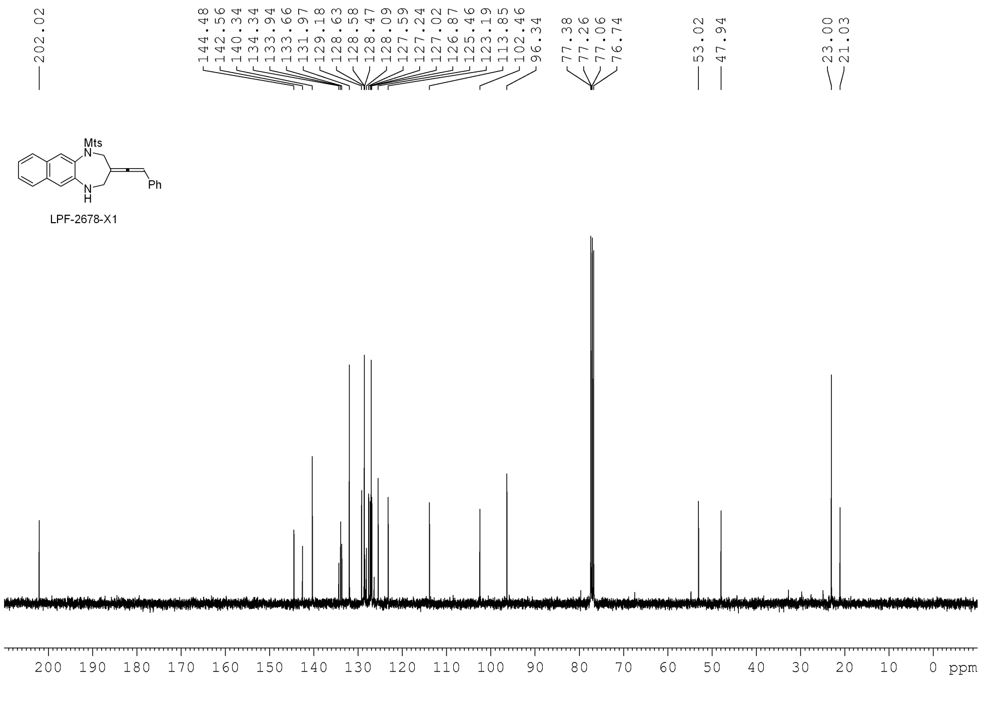

202.02
144.48
142.56
140.34
134.34
133.94
133.66
131.97
129.18
128.63
128.58
128.47
128.09
127.59
127.24
127.02
126.87
125.46
123.19
113.85
102.46
96.34
77.38
77.26
77.06
76.74
53.02
47.94
23.00
21.03
Mts
N
H
Ph
LPF-2678-X1
200
190
180
170
160
150
140
130
120
110
100
90
80
70
60
50
40
30
20
10
0 ppm

NMR spectra of 4

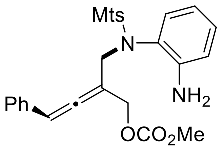

4a

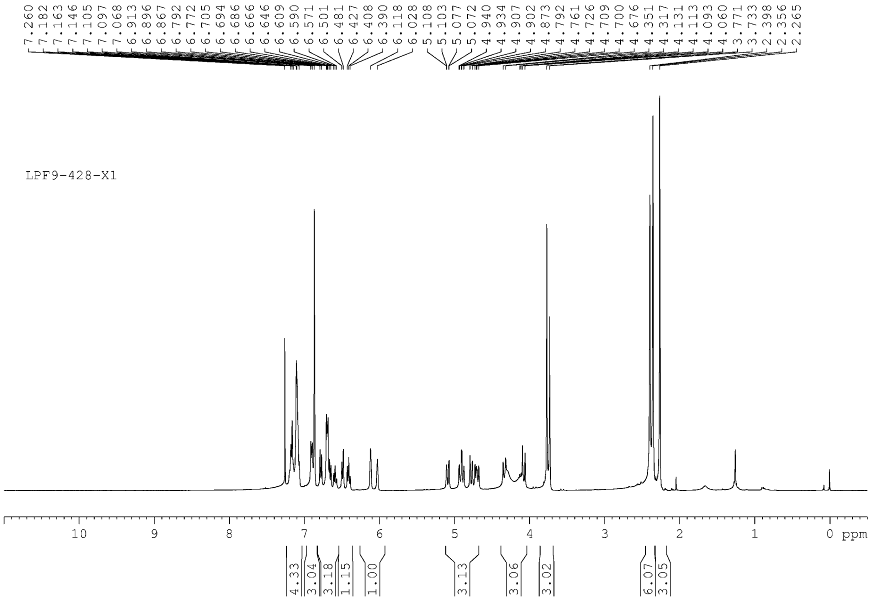

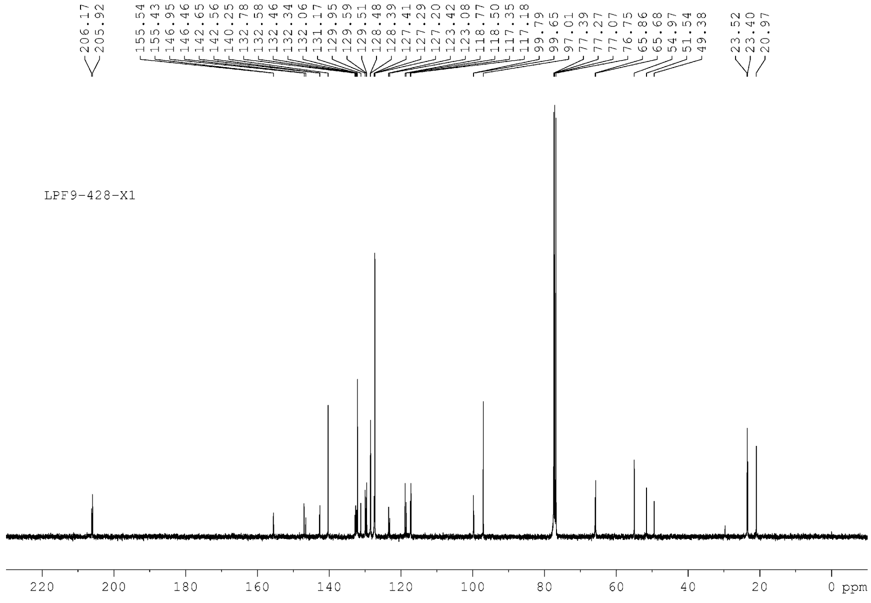

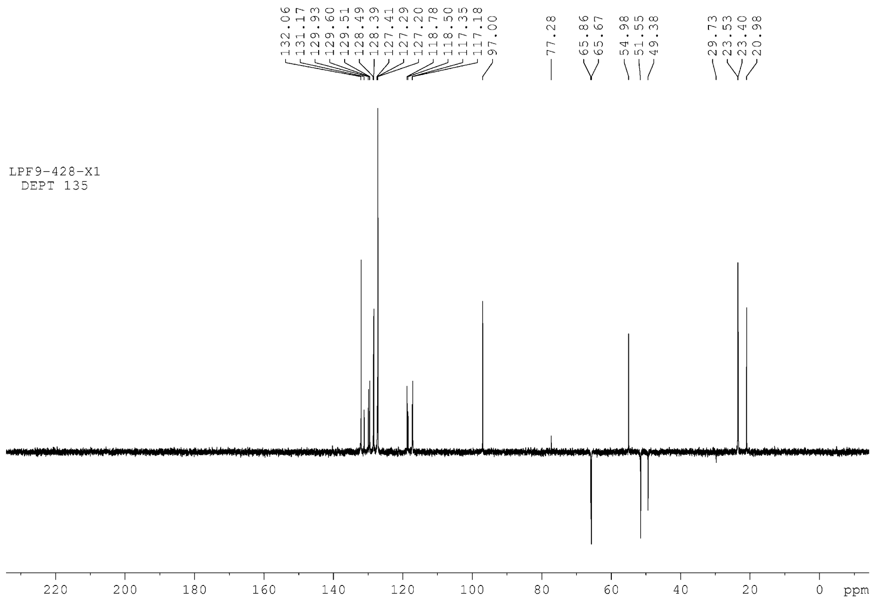

132.06
131.17
129.93
129.60
129.51
128.49
128.39
127.41
127.29
127.20
118.78
118.50
117.35
117.18
97.00
77.28
65.86
65.67
54.98
51.55
49.38
29.73
23.53
23.40
20.98
LPF9-428-X1
DEPT 135
220
200
180
160
140
120
100
80
60
40
20
0
ppm

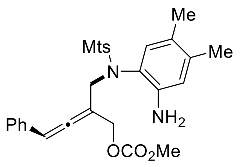
Me
Me
Mts
N
NH2
Ph
OCO2Me

4b

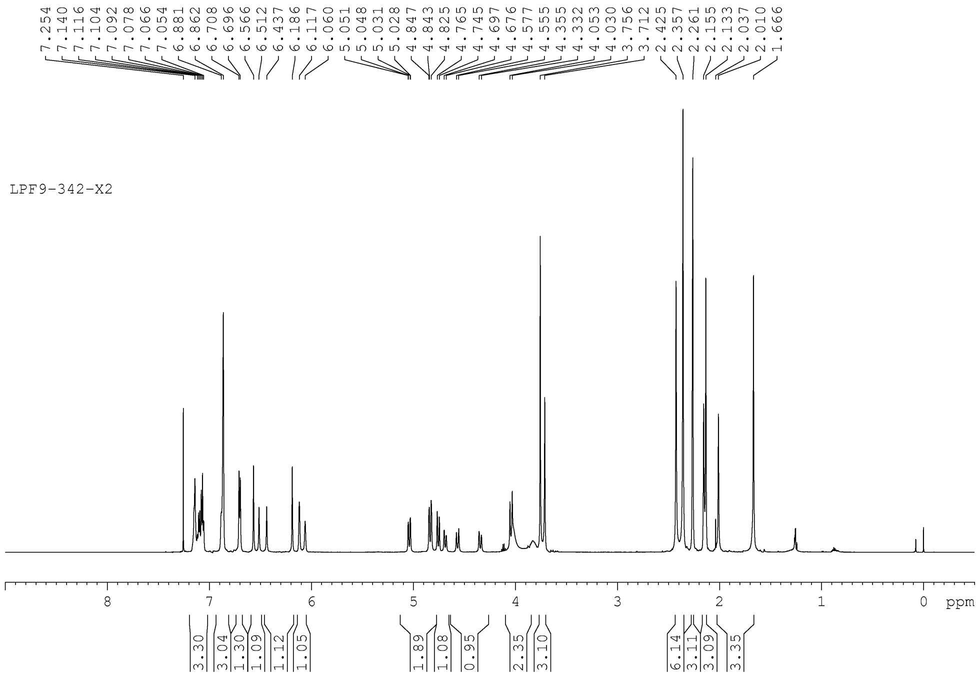
7.254
7.140
7.116
7.104
7.092
7.078
7.066
7.054
6.881
6.862
6.708
6.696
6.566
6.512
6.437
6.186
6.117
6.060
5.051
5.048
5.031
5.028
4.847
4.843
4.825
4.765
4.745
4.697
4.676
4.577
4.555
4.355
4.332
4.053
4.030
3.756
3.712
2.425
2.357
2.261
2.155
2.133
2.037
2.010
1.666
LPF9-342-X2
8
7
6
5
4
3
2
1
0
ppm
3.30
3.04
1.30
1.09
1.12
1.05
1.89
1.08
0.95
2.35
3.10
6.14
3.11
3.09
3.35

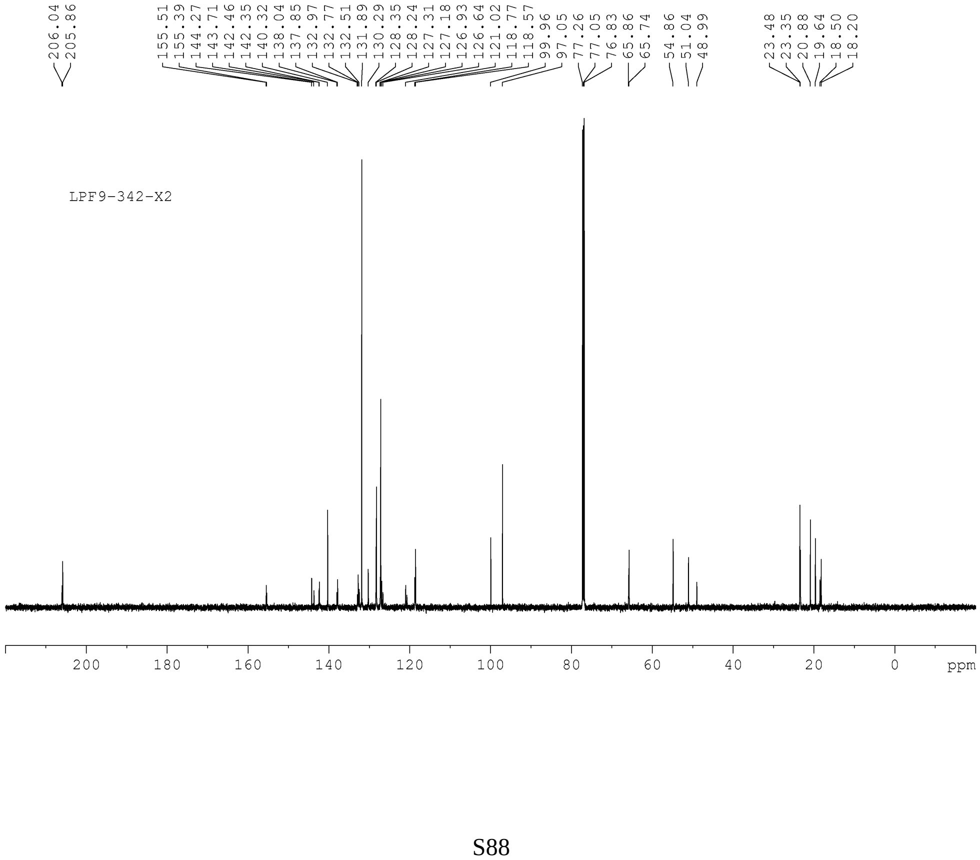
206.04
205.86
155.51
155.39
144.27
143.71
142.46
142.35
140.32
138.04
137.85
132.97
132.77
132.51
131.89
130.29
128.35
128.24
127.31
127.18
126.93
126.64
121.02
118.77
118.57
99.96
97.05
77.26
77.05
76.83
65.86
65.74
54.86
51.04
48.99
23.48
23.35
20.88
19.64
18.50
18.20
LPF9-342-X2
200
180
160
140
120
100
80
60
40
20
0
ppm

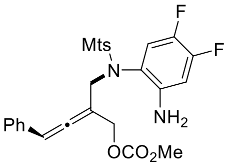
F
Mts
N
F
Ph
NH2
OCO2Me

4c

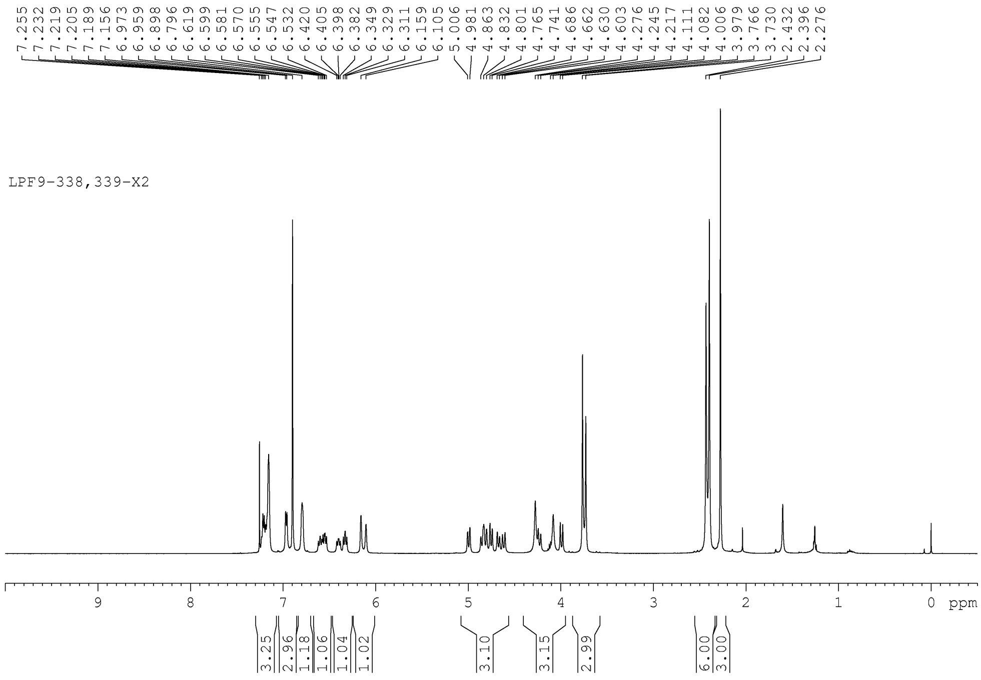
7.255
7.232
7.219
7.205
7.189
7.156
6.973
6.959
6.898
6.796
6.619
6.599
6.581
6.570
6.555
6.547
6.532
6.420
6.405
6.398
6.382
6.349
6.329
6.311
6.159
6.105
5.006
4.981
4.863
4.832
4.801
4.765
4.741
4.686
4.662
4.630
4.603
4.276
4.245
4.217
4.111
4.082
4.006
3.979
3.766
3.730
2.432
2.396
2.276
LPF9-338,339-X2
9
8
7
6
5
4
3
2
1
0 ppm
3.25
2.96
1.18
1.06
1.04
1.02
3.10
3.15
2.99
6.00
3.00

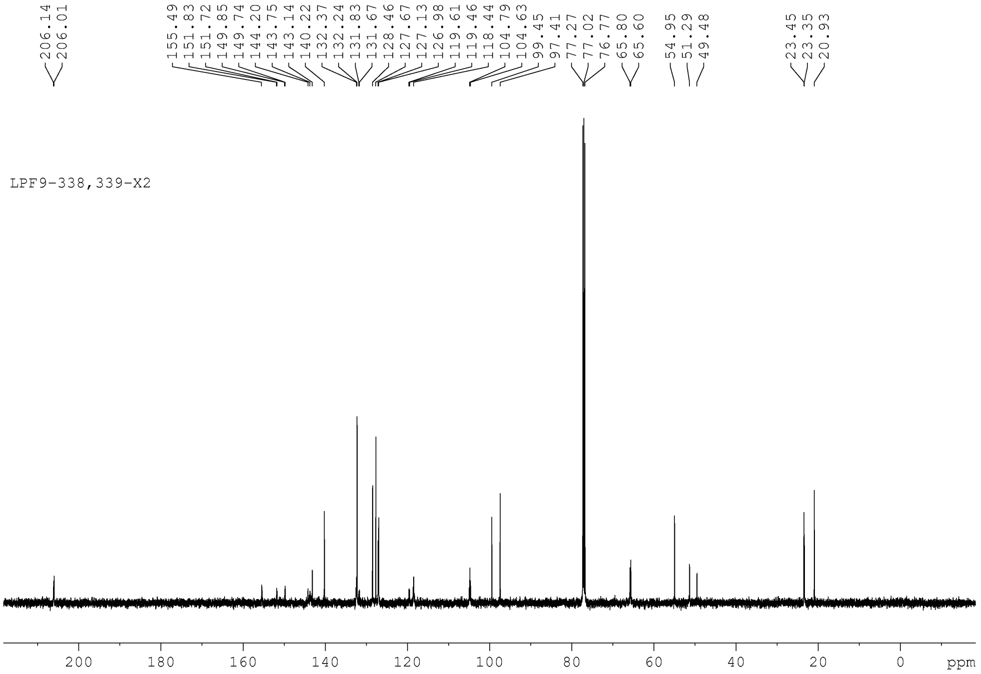
206.14
206.01
155.49
151.83
151.72
149.85
149.74
144.20
143.75
143.14
140.22
132.37
132.24
131.83
131.67
128.46
127.67
127.13
126.98
119.61
119.46
118.44
104.79
104.63
99.45
97.41
77.27
77.02
76.77
65.80
65.60
54.95
51.29
49.48
23.45
23.35
20.93
LPF9-338,339-X2
200
180
160
140
120
100
80
60
40
20
0
ppm

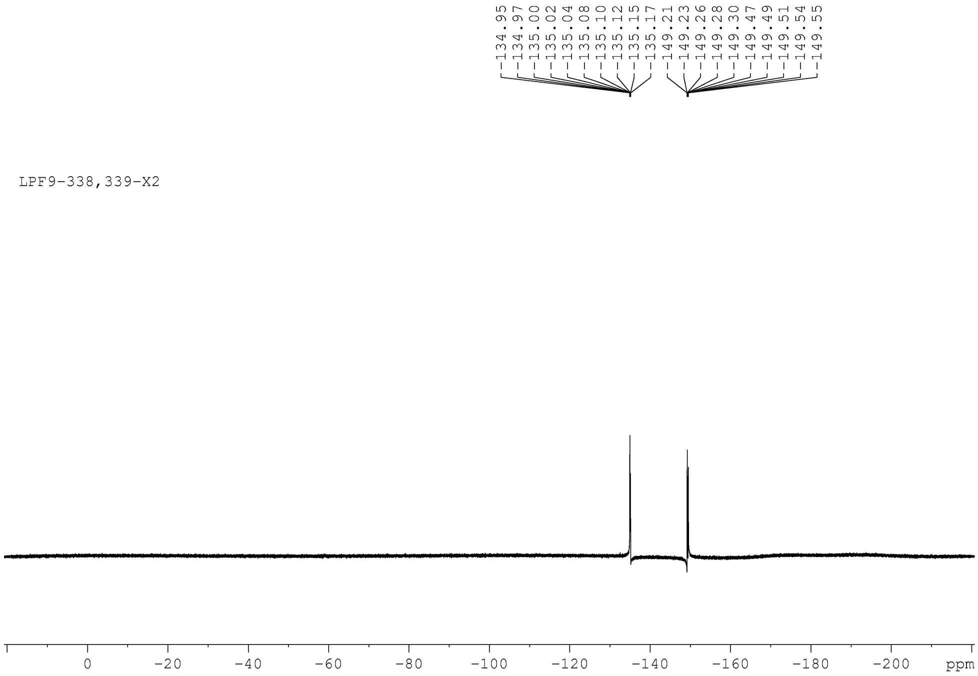
-134.95
-134.97
-135.00
-135.02
-135.04
-135.08
-135.10
-135.12
-135.15
-135.17
-149.21
-149.23
-149.26
-149.28
-149.30
-149.47
-149.49
-149.51
-149.54
-149.55
LPF9-338,339-X2
0
-20
-40
-60
-80
-100
-120
-140
-160
-180
-200
ppm

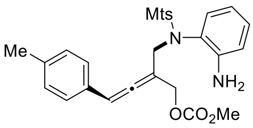
Mts
N
NH2
Me
OCO2Me

4d

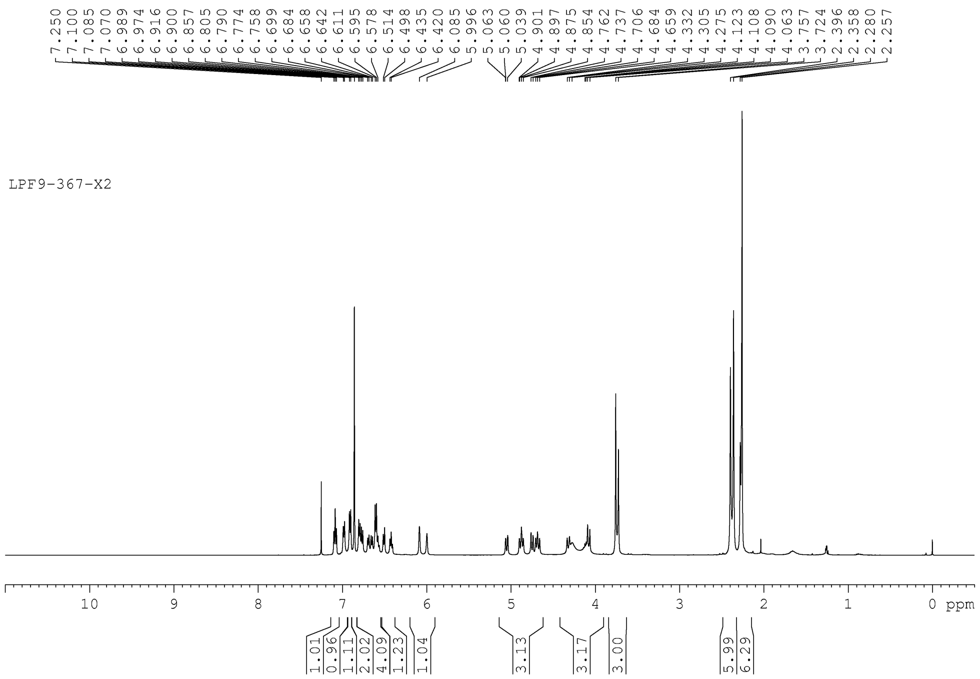
7.250
7.100
7.085
7.070
6.989
6.974
6.916
6.900
6.857
6.805
6.790
6.774
6.758
6.699
6.684
6.658
6.642
6.611
6.595
6.578
6.514
6.498
6.435
6.420
6.085
5.996
5.063
5.060
5.039
4.901
4.897
4.875
4.854
4.762
4.737
4.706
4.684
4.659
4.332
4.305
4.275
4.123
4.108
4.090
4.063
3.757
3.724
2.396
2.358
2.280
2.257
LPF9-367-X2
10
9
8
7
6
5
4
3
2
1
0 ppm
1.01
0.96
1.11
2.02
4.09
1.23
1.04
3.13
3.17
3.00
5.99
6.29

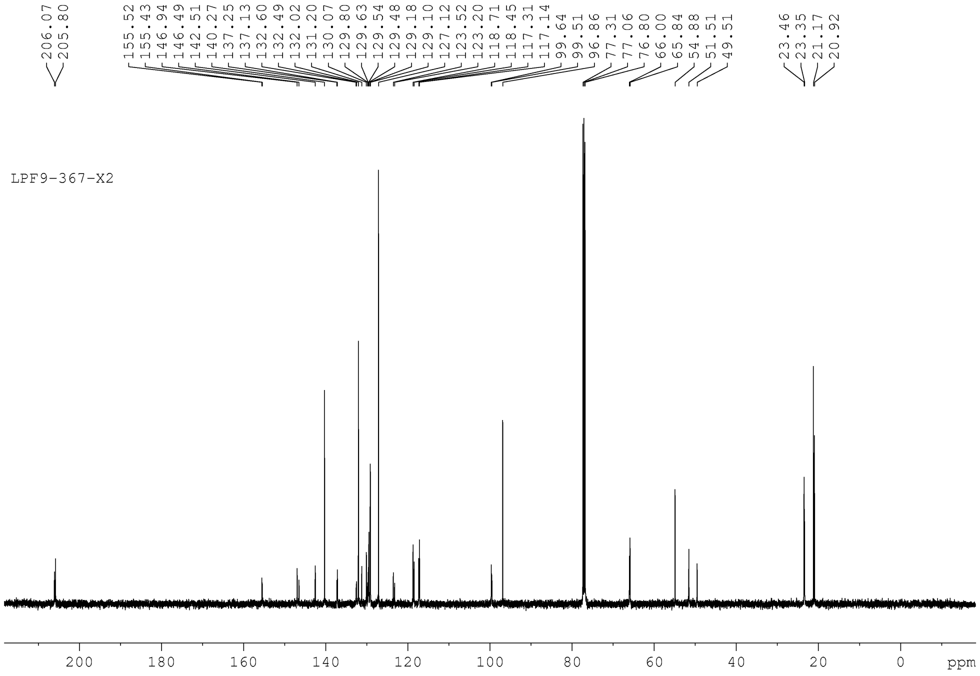
206.07
205.80
155.52
155.43
146.94
146.49
142.51
140.27
137.25
137.13
132.60
132.49
132.02
131.20
130.07
129.80
129.63
129.54
129.48
129.18
129.10
127.12
123.52
123.20
118.71
118.45
117.31
117.14
99.64
99.51
96.86
77.31
77.06
76.80
66.00
65.84
54.88
51.51
49.51
23.46
23.35
21.17
20.92
LPF9-367-X2
200
180
160
140
120
100
80
60
40
20
0
ppm

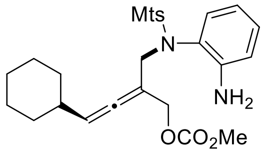

4e

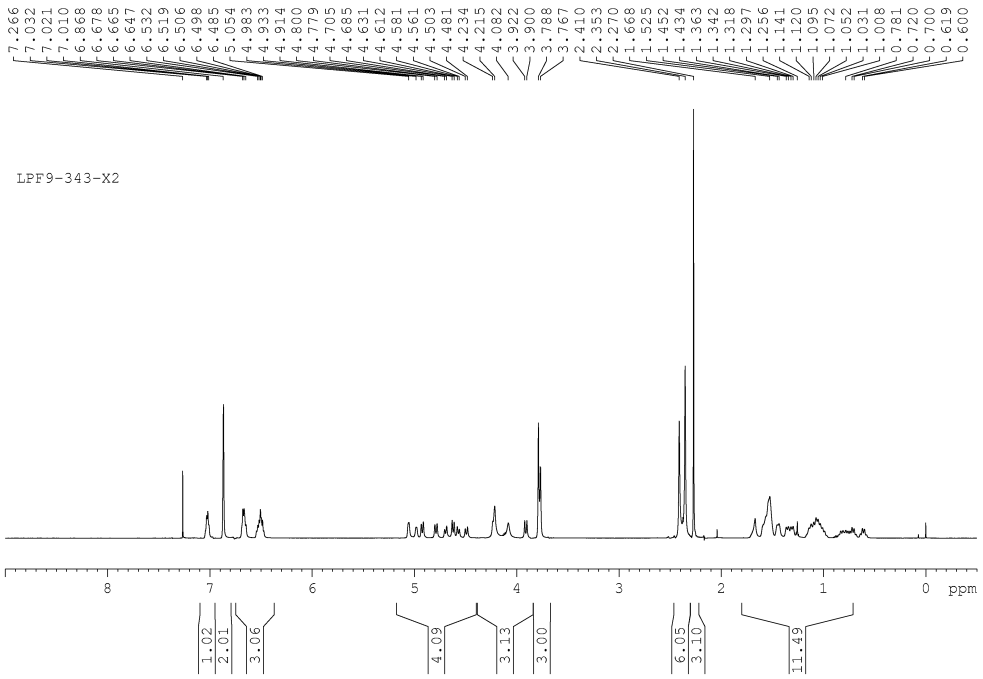

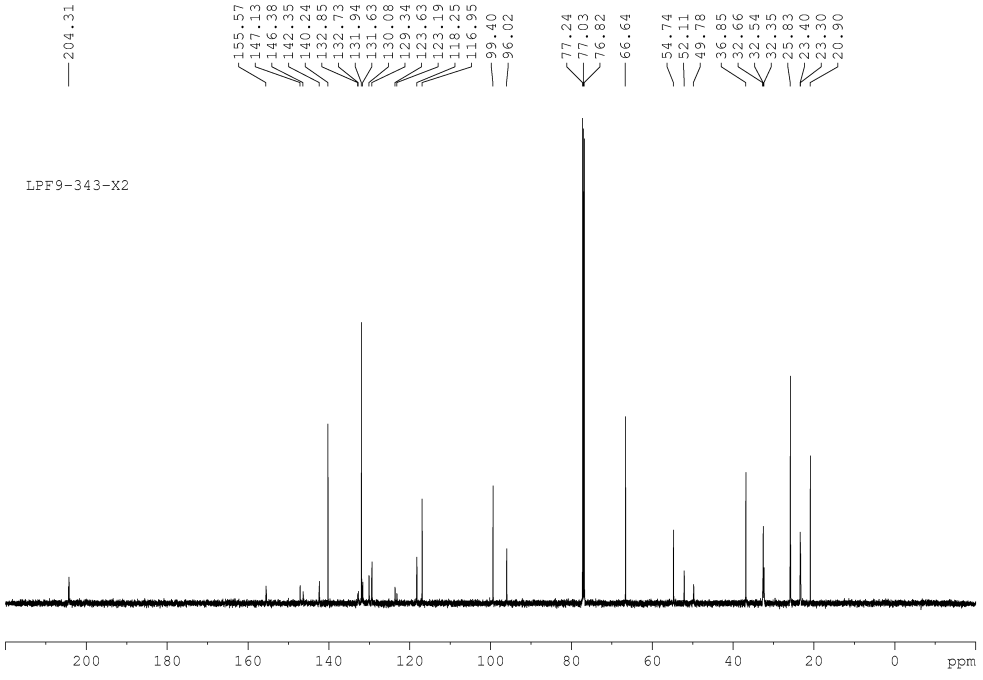

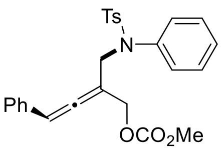

4f

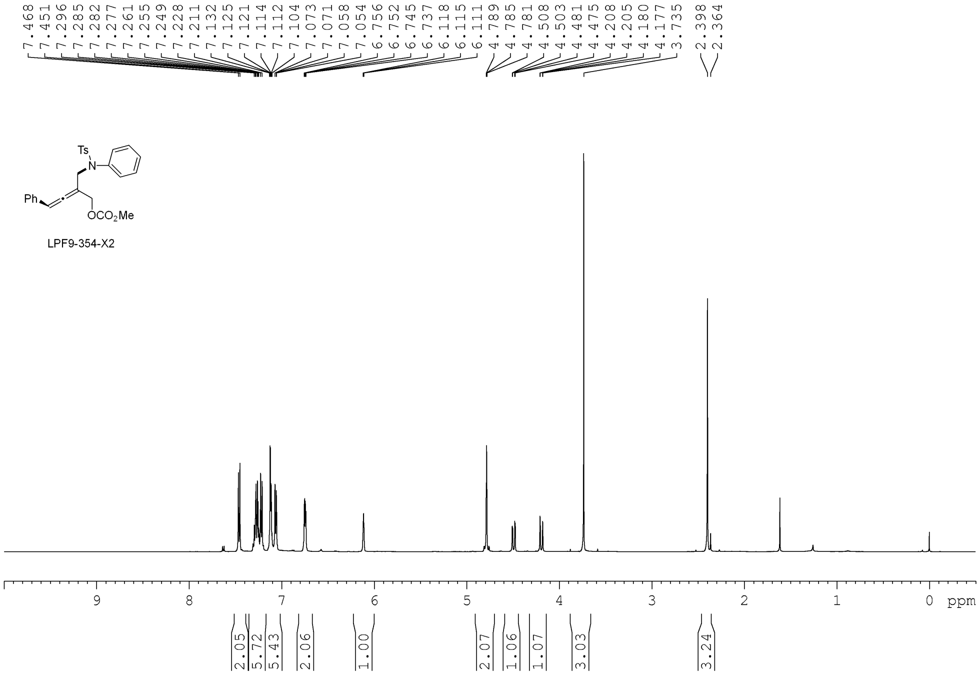
7.468
7.451
7.296
7.285
7.282
7.277
7.261
7.255
7.249
7.228
7.211
7.132
7.125
7.121
7.114
7.112
7.104
7.073
7.071
7.058
7.054
6.756
6.752
6.745
6.737
6.118
6.115
6.111
4.789
4.785
4.781
4.508
4.503
4.481
4.475
4.208
4.205
4.180
4.177
3.735
2.398
2.364
LPF9-354-X2
9
8
7
6
5
4
3
2
1
0 ppm
2.05
5.72
5.43
2.06
1.00
2.07
1.06
1.07
3.03
3.24

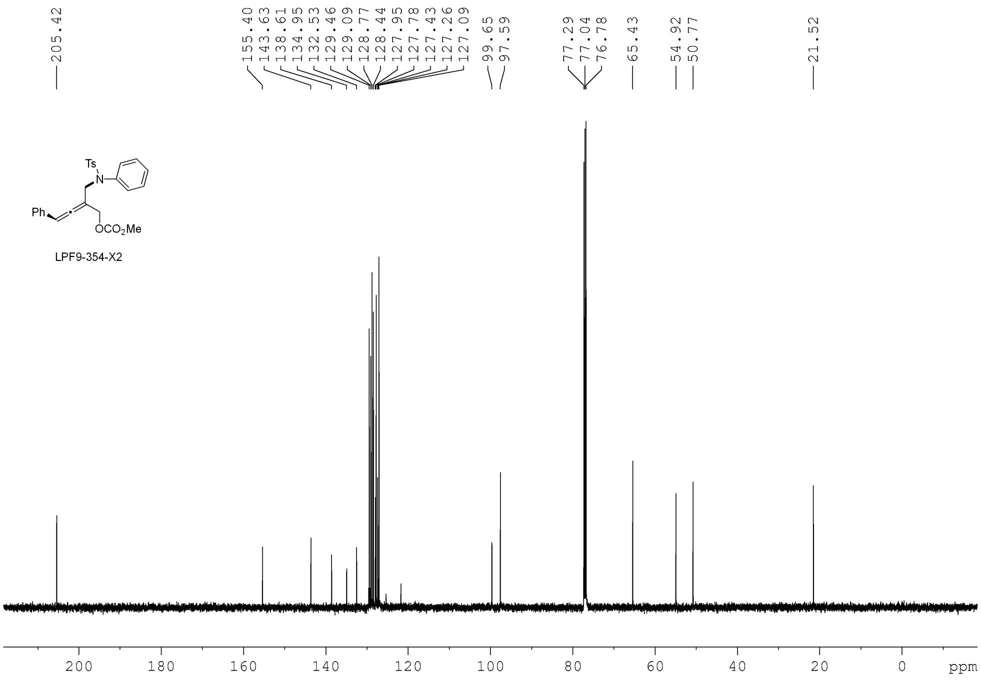
205.42
155.40
143.63
138.61
134.95
132.53
129.46
129.09
128.77
128.44
127.95
127.78
127.43
127.26
127.09
99.65
97.59
77.29
77.04
76.78
65.43
54.92
50.77
21.52
LPF9-354-X2
200
180
160
140
120
100
80
60
40
20
0
ppm

NMR spectra of 9

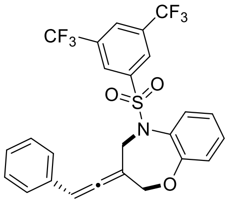

9a

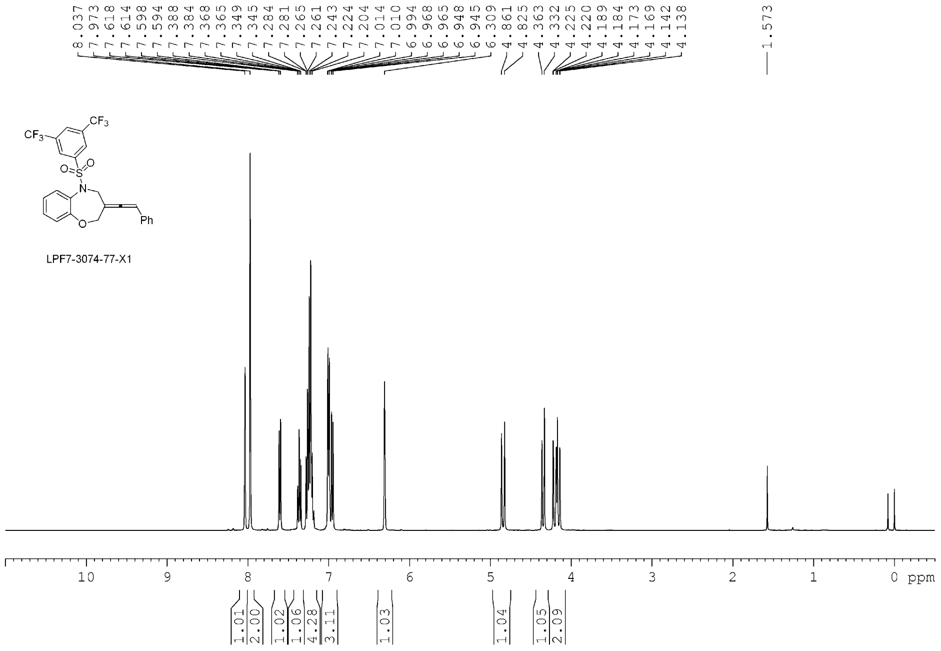

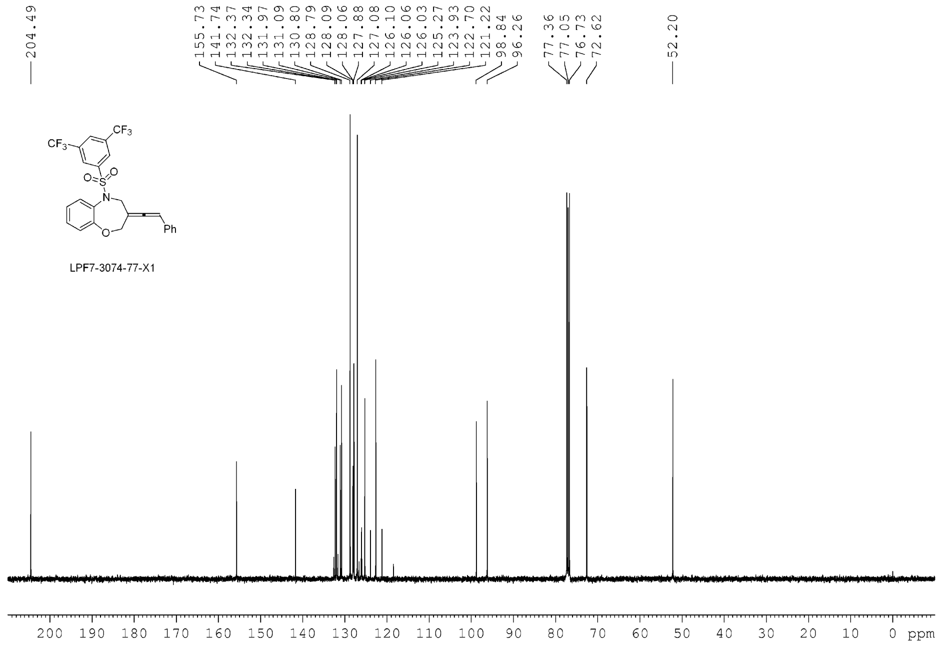

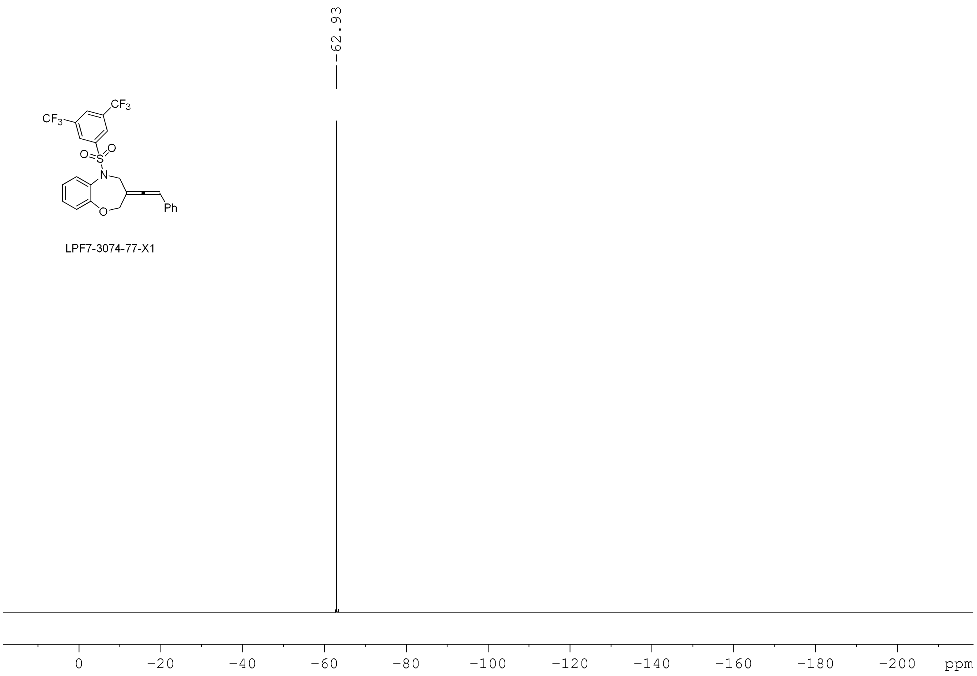

-62.93
CF3
CF3
O
S
O
N
O
Ph
LPF7-3074-77-X1
0
-20
-40
-60
-80
-100
-120
-140
-160
-180
-200
ppm

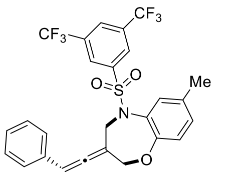

CF3
CF3
O=S=O
N
Me
O

9b

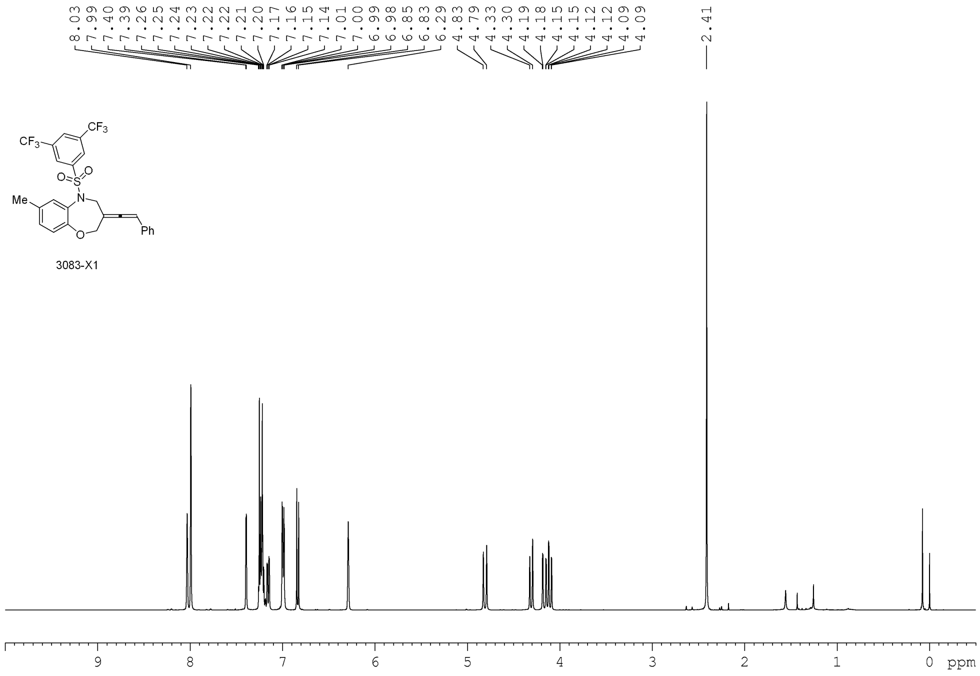

8.03
7.99
7.40
7.39
7.26
7.25
7.24
7.23
7.22
7.22
7.21
7.20
7.17
7.16
7.15
7.14
7.01
7.00
6.99
6.98
6.85
6.83
6.29
4.83
4.79
4.33
4.30
4.19
4.18
4.15
4.15
4.12
4.12
4.09
4.09
2.41
3083-X1
9 8 7 6 5 4 3 2 1 0 ppm

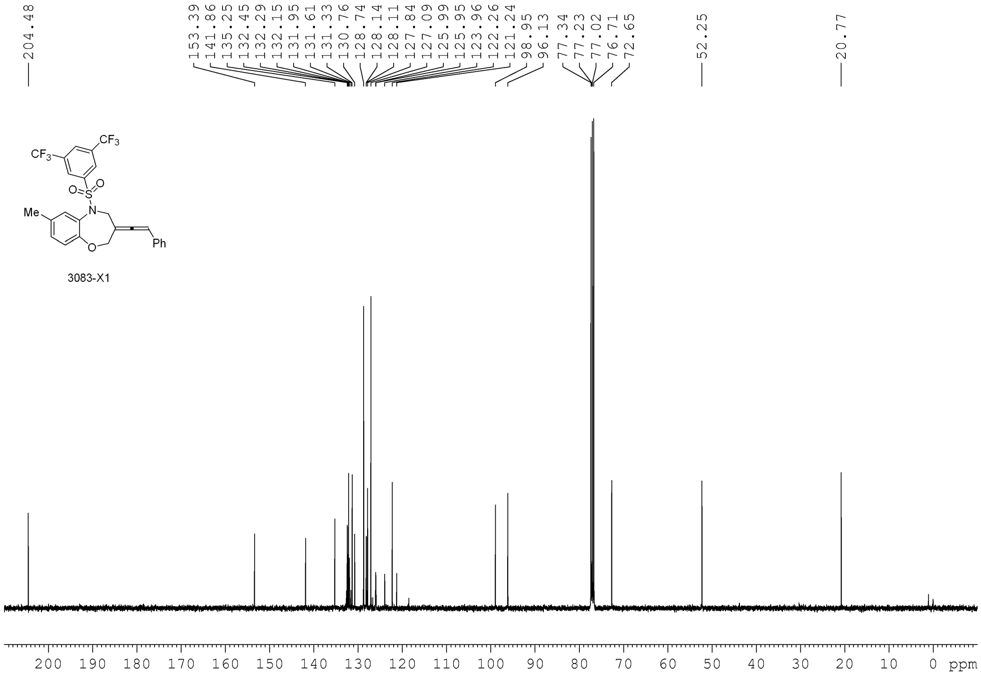

204.48
153.39
141.86
135.25
132.45
132.29
132.15
131.95
131.61
131.33
130.76
128.74
128.14
128.11
127.84
127.09
125.99
125.95
123.96
122.26
121.24
98.95
96.13
77.34
77.23
77.02
76.71
72.65
52.25
20.77
3083-X1
200 190 180 170 160 150 140 130 120 110 100 90 80 70 60 50 40 30 20 10 0 ppm

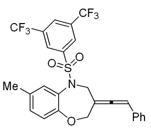

3083-X1

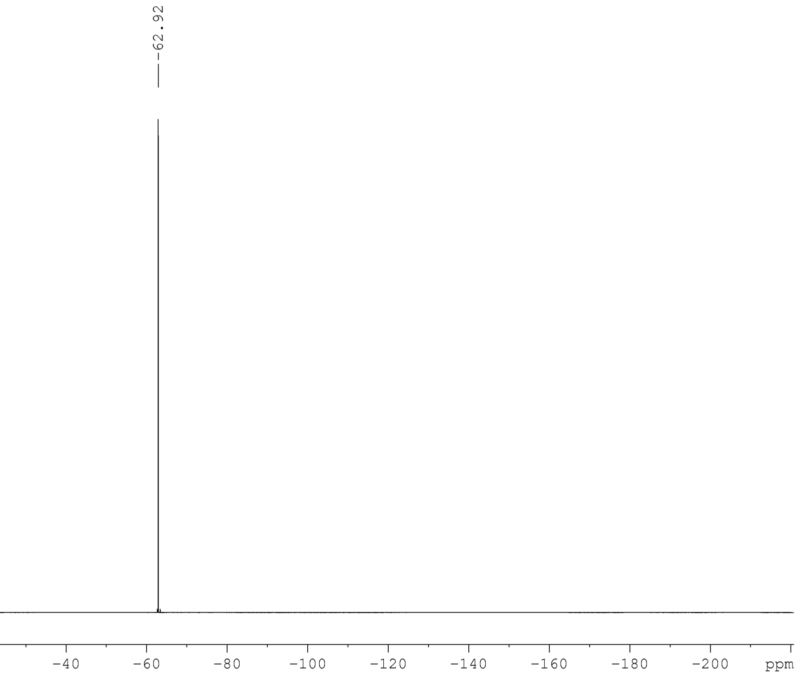

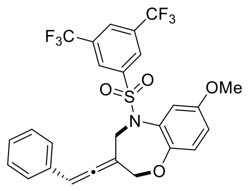
CF3
F3C
O
S
O
N
OMe
O

9c

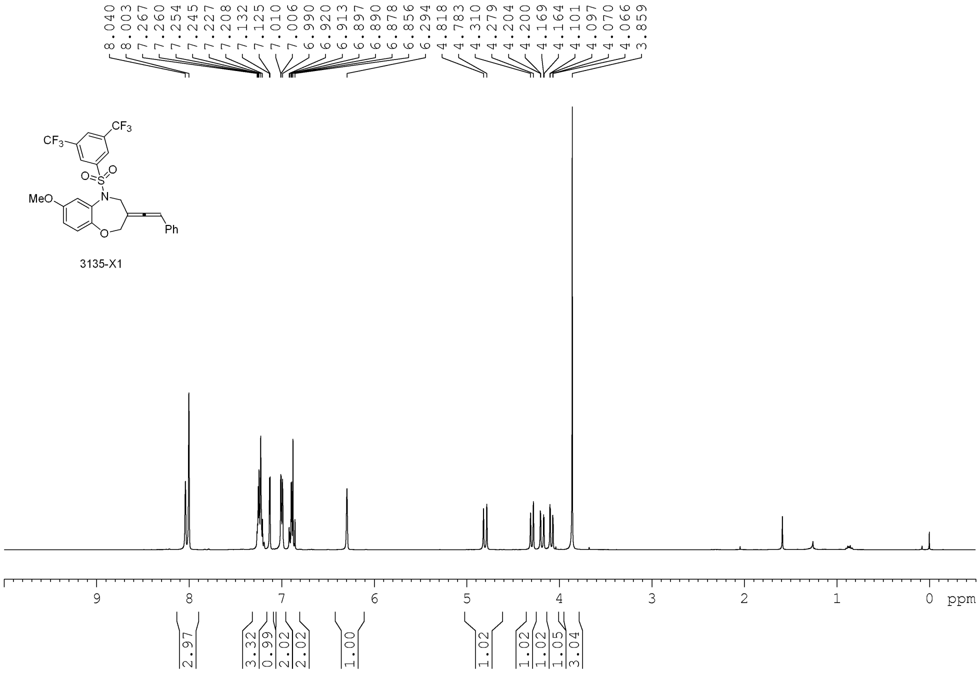
8.040
8.003
7.267
7.260
7.254
7.245
7.227
7.208
7.132
7.125
7.010
7.006
6.990
6.920
6.913
6.897
6.890
6.878
6.856
6.294
4.818
4.783
4.310
4.279
4.204
4.200
4.169
4.164
4.101
4.097
4.070
4.066
3.859
CF3
CF3
O
S
O
N
MeO
O
Ph
3135-X1
9
8
7
6
5
4
3
2
1
0 ppm
2.97
3.32
0.99
2.02
2.02
1.00
1.02
1.02
1.02
1.05
3.04

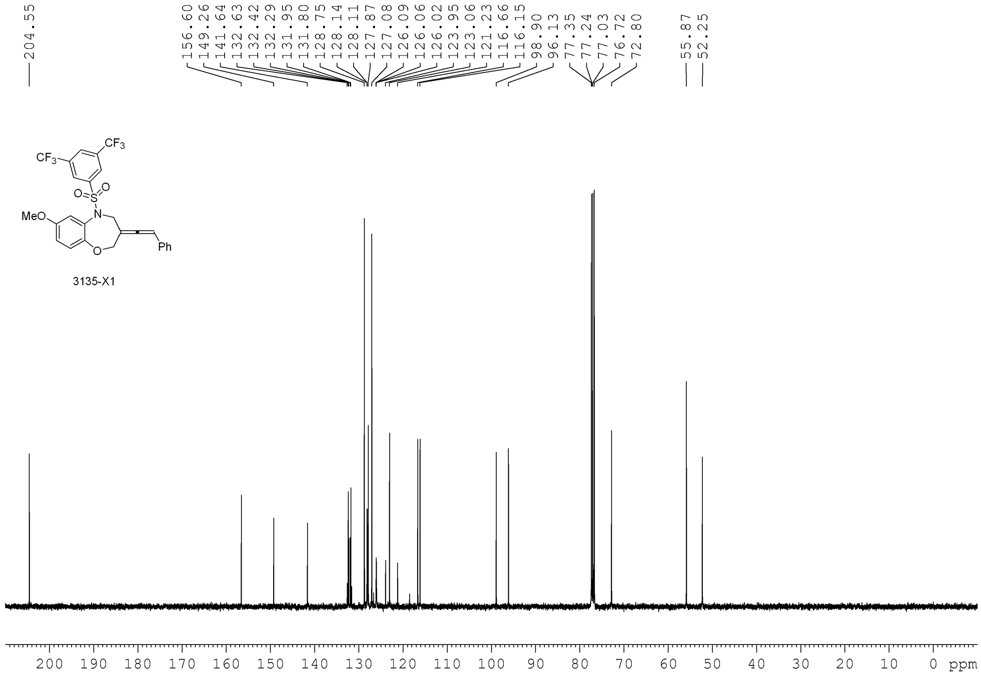
204.55
156.60
149.26
141.64
132.63
132.42
132.29
131.95
131.80
128.75
128.14
128.11
127.87
127.08
126.09
126.06
126.02
123.95
123.06
121.23
116.66
116.15
98.90
96.13
77.35
77.24
77.03
76.72
72.80
55.87
52.25
CF3
CF3
O
S
O
N
MeO
O
Ph
3135-X1
200
190
180
170
160
150
140
130
120
110
100
90
80
70
60
50
40
30
20
10
0 ppm

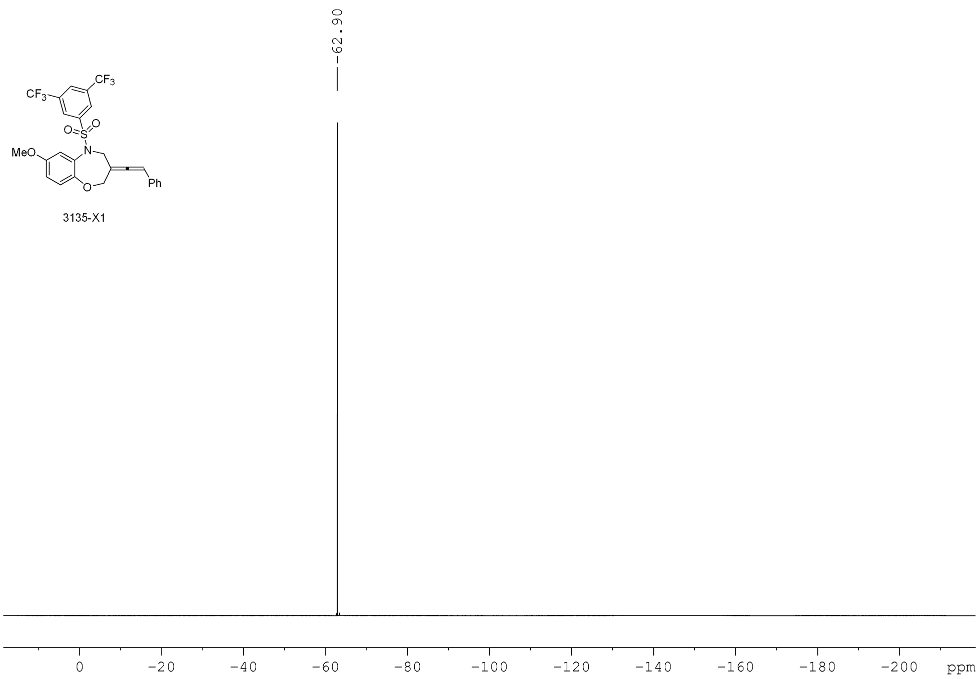
-62.90
3135-X1
0
-20
-40
-60
-80
-100
-120
-140
-160
-180
-200
ppm

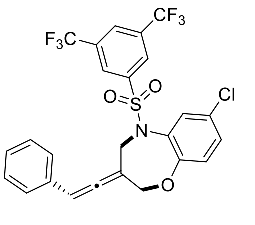
CF3
F3C
O=S=O
N
Cl
O

9d

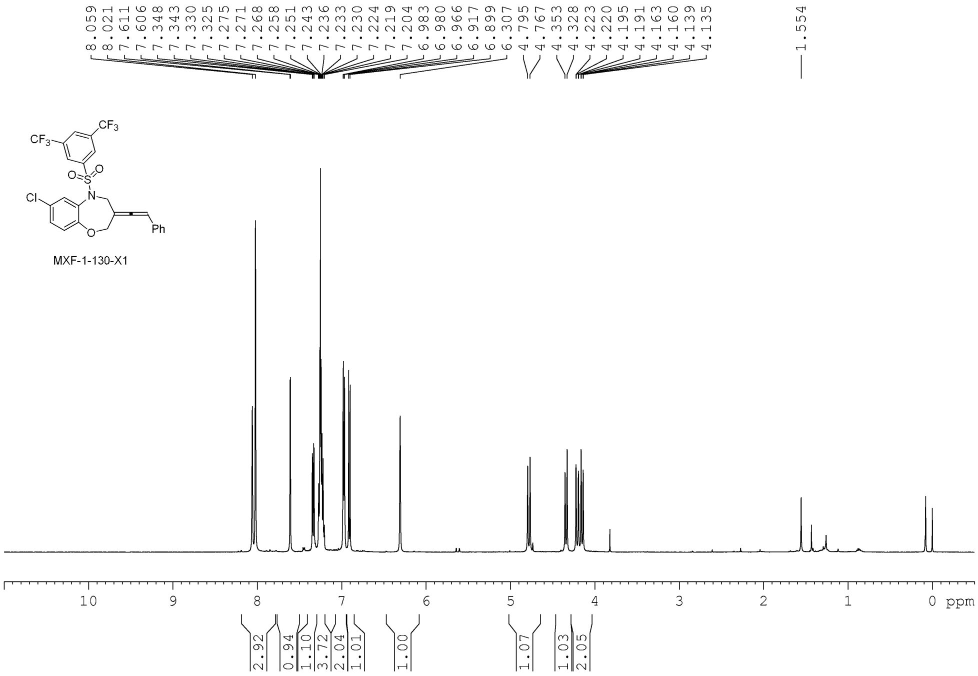
8.059
8.021
7.611
7.606
7.348
7.343
7.330
7.325
7.275
7.271
7.268
7.258
7.251
7.243
7.236
7.233
7.230
7.224
7.219
7.204
6.983
6.980
6.966
6.917
6.899
6.307
4.795
4.767
4.353
4.328
4.223
4.220
4.195
4.191
4.163
4.160
4.139
4.135
1.554
MXF-1-130-X1
10 9 8 7 6 5 4 3 2 1 0 ppm
2.92
0.94
1.10
3.72
2.04
1.01
1.00
1.07
1.03
2.05

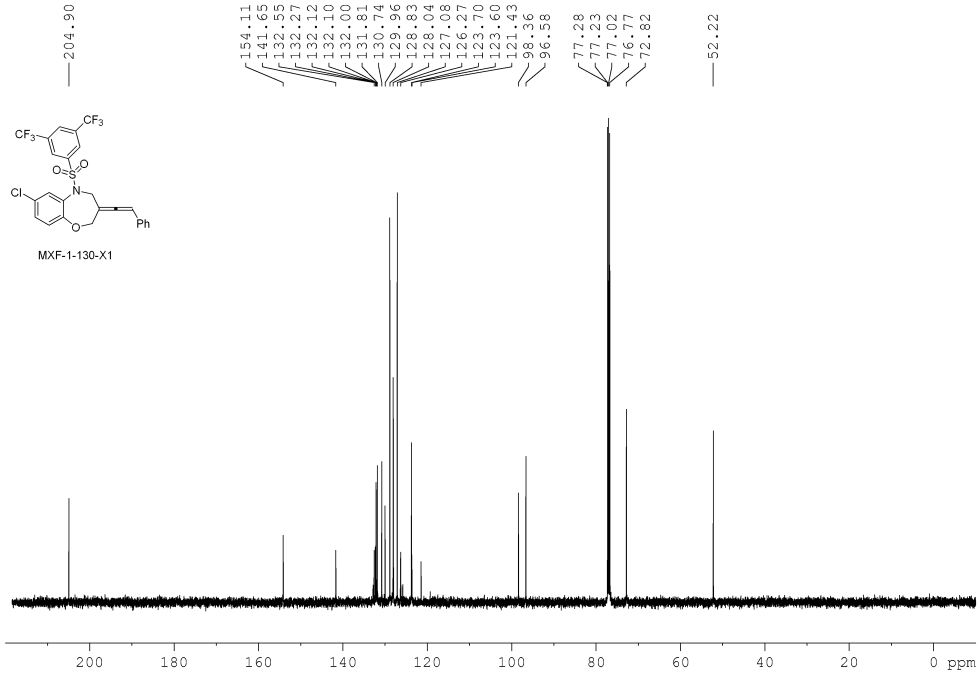
204.90
154.11
141.65
132.55
132.27
132.12
132.10
132.00
131.81
130.74
129.96
128.83
128.04
127.08
126.27
123.70
123.60
121.43
98.36
96.58
77.28
77.23
77.02
76.77
72.82
52.22
MXF-1-130-X1
200 180 160 140 120 100 80 60 40 20 0 ppm

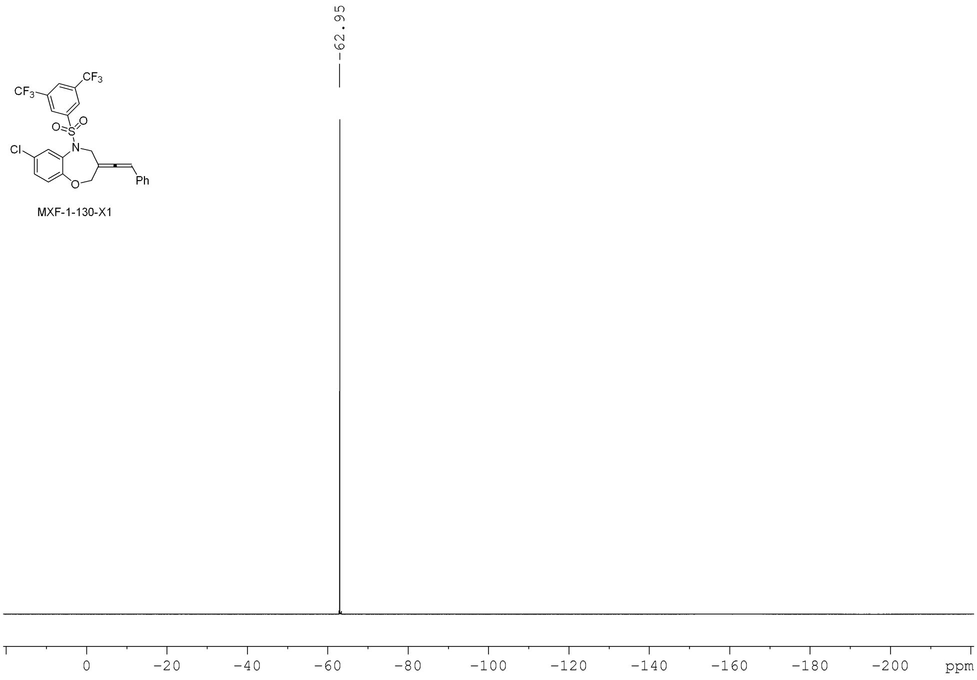

-62.95
CF3
CF3
O
S
O
N
Cl
O
Ph
MXF-1-130-X1
0
-20
-40
-60
-80
-100
-120
-140
-160
-180
-200
ppm

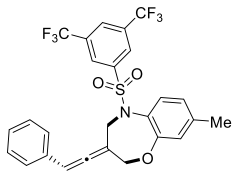
CF3
F3C
O
S
O
N
Me
O

9e

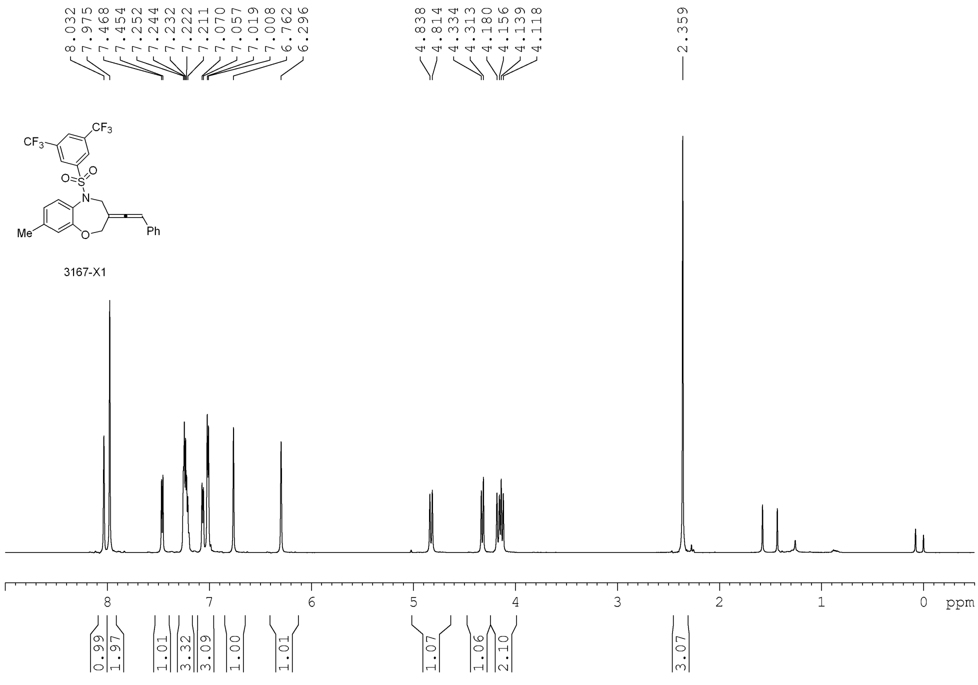
8.032
7.975
7.468
7.454
7.252
7.244
7.232
7.222
7.211
7.070
7.057
7.019
7.008
6.762
6.296
4.838
4.814
4.334
4.313
4.180
4.156
4.139
4.118
2.359
3167-X1
0.99
1.97
1.01
3.32
3.09
1.00
1.01
1.07
1.06
2.10
3.07
ppm

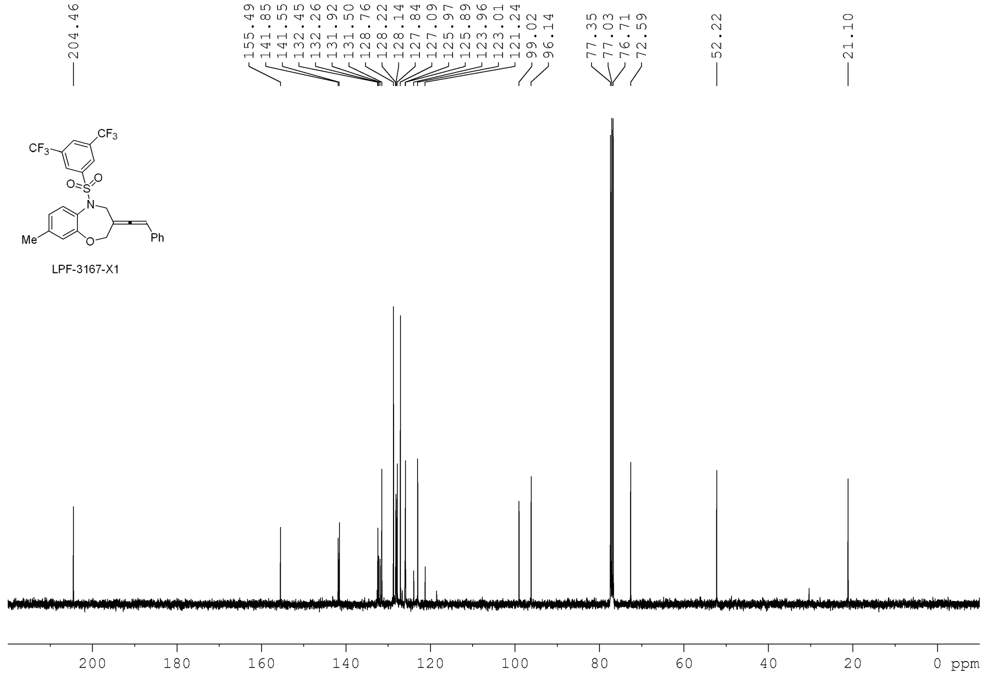
204.46
155.49
141.85
141.55
132.45
132.26
131.92
131.50
128.76
128.22
128.14
127.84
127.09
125.97
125.89
123.96
123.01
121.24
99.02
96.14
77.35
77.03
76.71
72.59
52.22
21.10
LPF-3167-X1
ppm

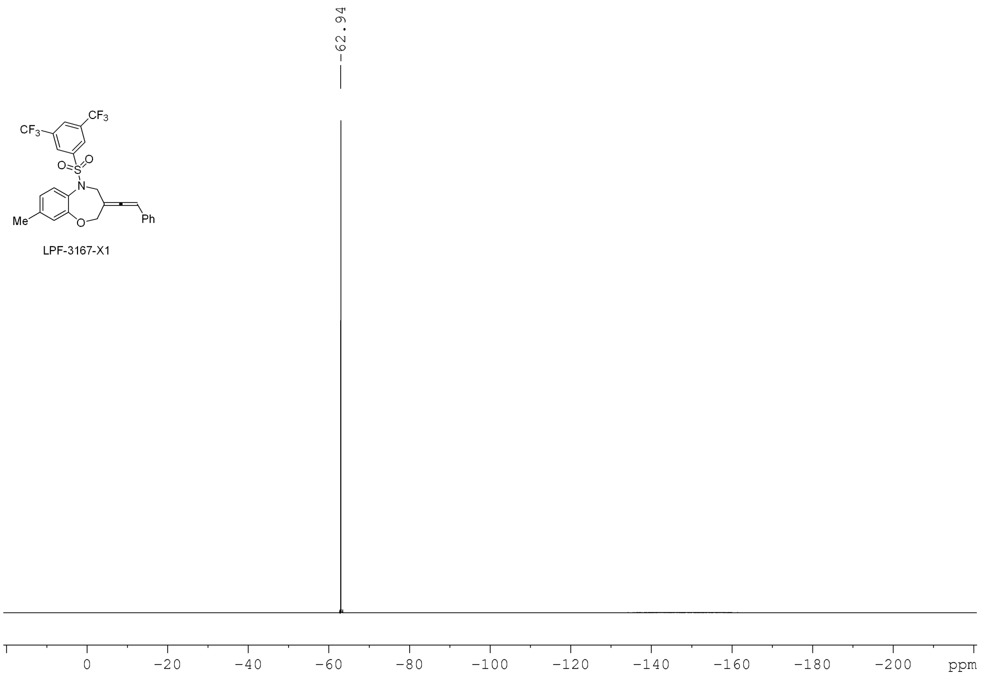

-62.94
CF3
CF3
O=S=O
N
O
Me
Ph
LPF-3167-X1
0
-20
-40
-60
-80
-100
-120
-140
-160
-180
-200
ppm

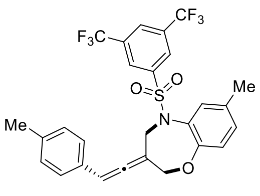

9f

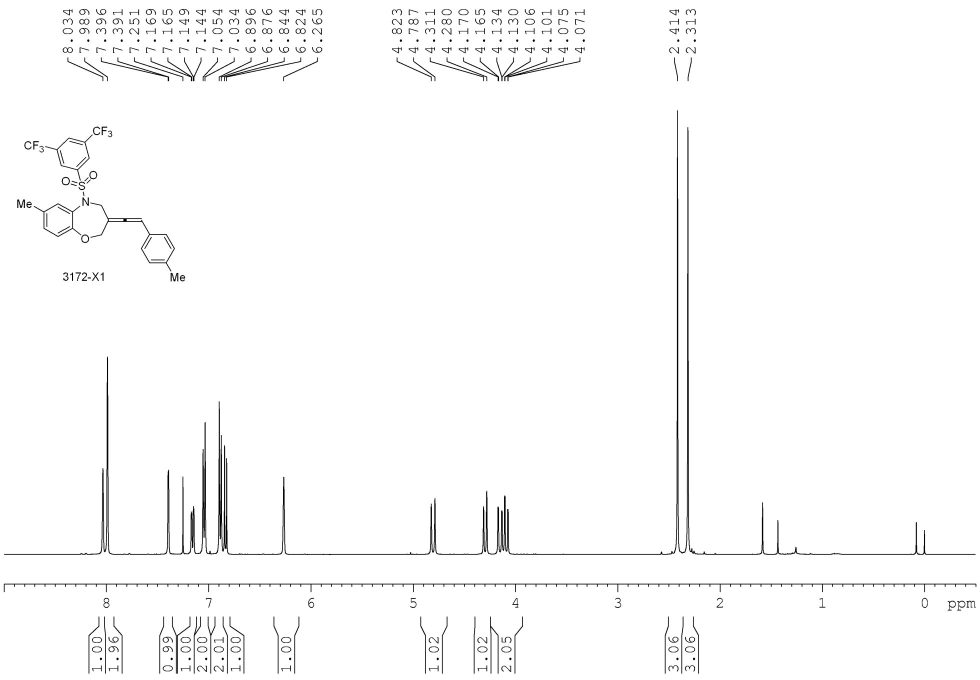
8.034
7.989
7.396
7.391
7.251
7.169
7.165
7.149
7.144
7.054
7.034
6.896
6.876
6.844
6.824
6.265
4.823
4.787
4.311
4.280
4.170
4.165
4.134
4.130
4.106
4.101
4.075
4.071
2.414
2.313
3172-X1
ppm
1.00
1.96
0.99
1.00
2.00
2.01
1.00
1.00
1.02
1.02
2.05
3.06
3.06

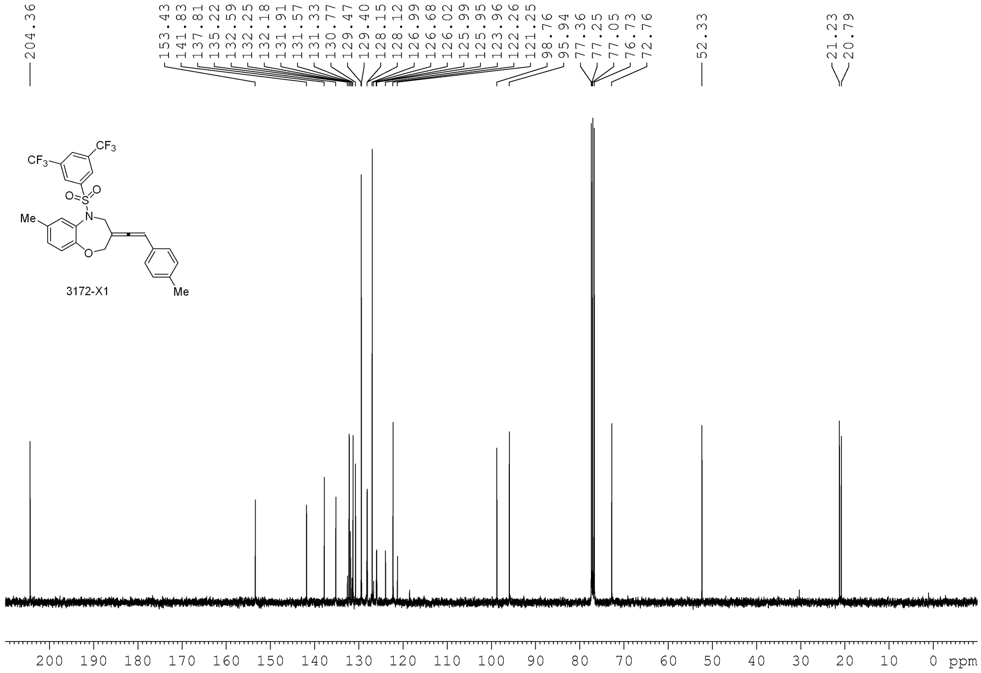
204.36
153.43
141.83
137.81
135.22
132.59
132.25
132.18
131.91
131.57
131.33
130.77
129.47
129.40
128.15
128.12
126.99
126.68
126.02
125.99
125.95
123.96
122.26
121.25
98.76
95.94
77.36
77.25
77.05
76.73
72.76
52.33
21.23
20.79
3172-X1
ppm

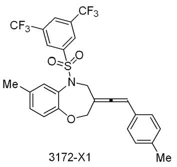

3172-X1

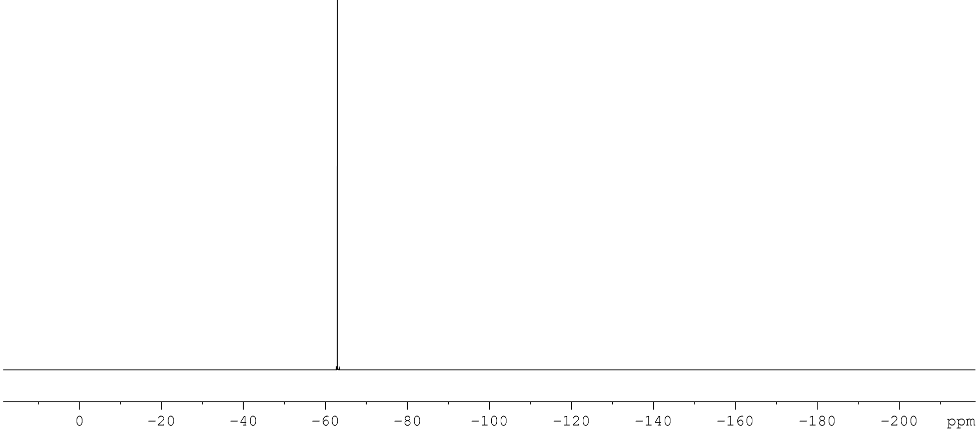

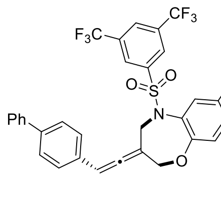
F3C
CF3
O=S=O
N
Me
Ph
O

9g

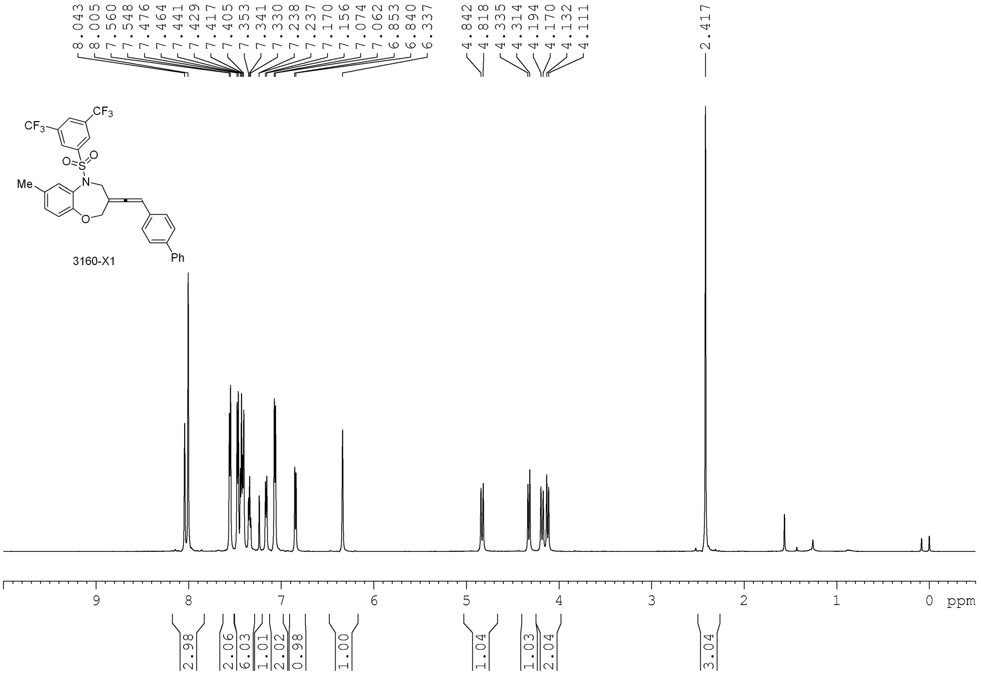
8.043 8.005 7.560 7.548 7.476 7.464 7.441 7.429 7.417 7.405 7.353 7.341 7.330 7.238 7.237 7.170 7.156 7.074 7.062 6.853 6.840 6.337
4.842 4.818 4.335 4.314 4.194 4.170 4.132 4.111
2.417
3160-X1
9 8 7 6 5 4 3 2 1 0 ppm
2.98 2.06 6.03 1.01 2.02 0.98 1.00 1.04 1.03 2.04 3.04

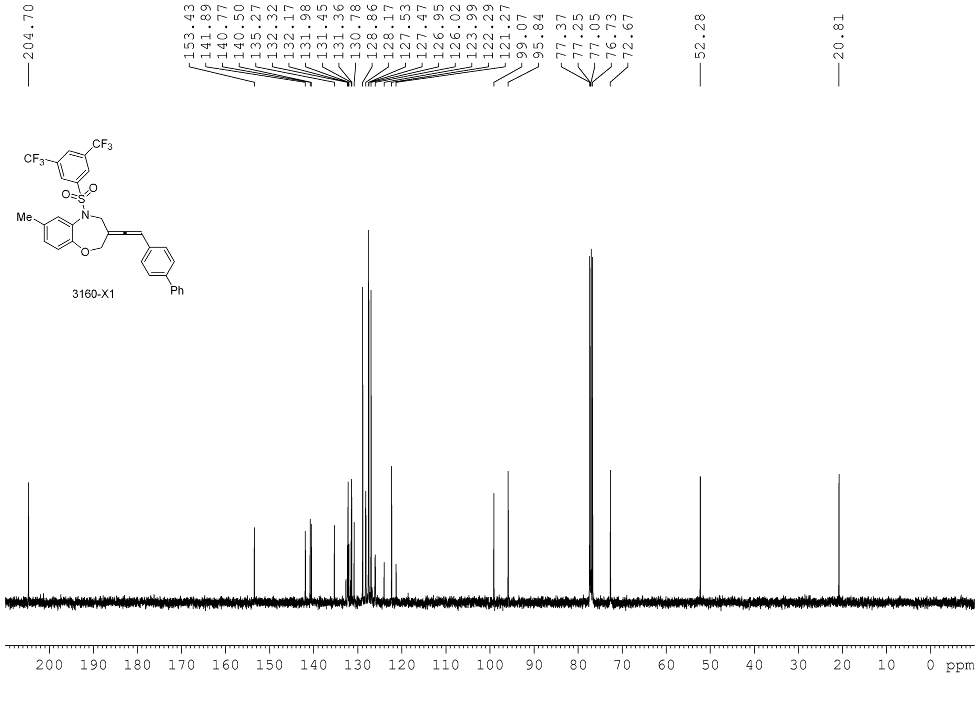
204.70
153.43 141.89 140.77 140.50 135.27 132.32 132.17 131.98 131.45 131.36 130.78 128.86 128.17 127.53 127.47 126.95 126.02 123.99 122.29 121.27 99.07 95.84
77.37 77.25 77.05 76.73 72.67
52.28
20.81
3160-X1
200 190 180 170 160 150 140 130 120 110 100 90 80 70 60 50 40 30 20 10 0 ppm

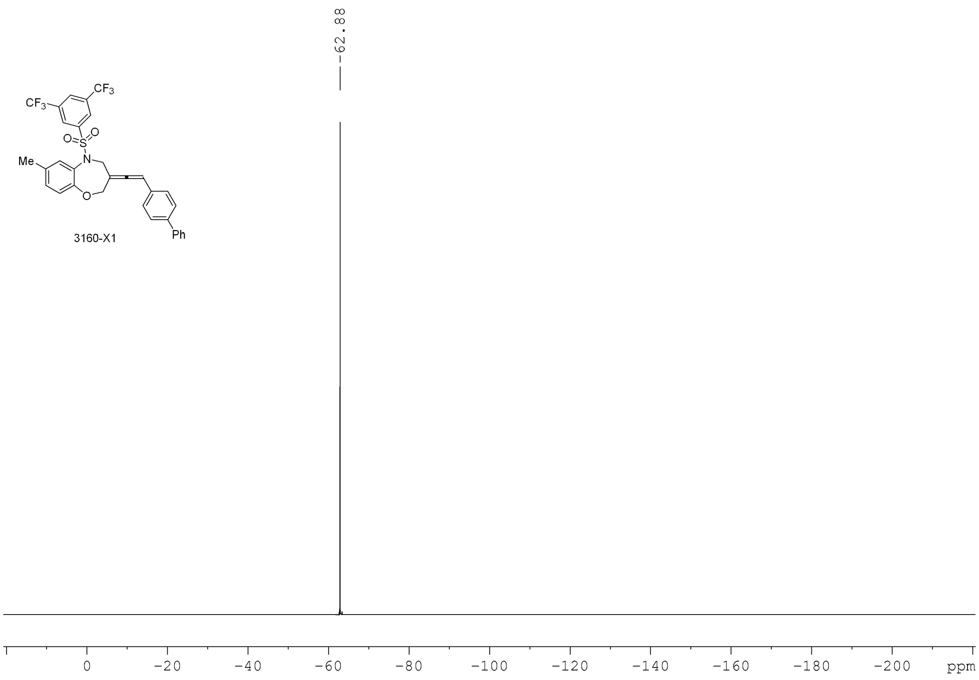
3160-X1
-62.88
0
-20
-40
-60
-80
-100
-120
-140
-160
-180
-200
ppm

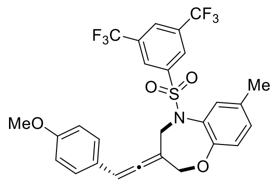

CF3
F3C
O
S
O
Me
N
MeO
O

9h

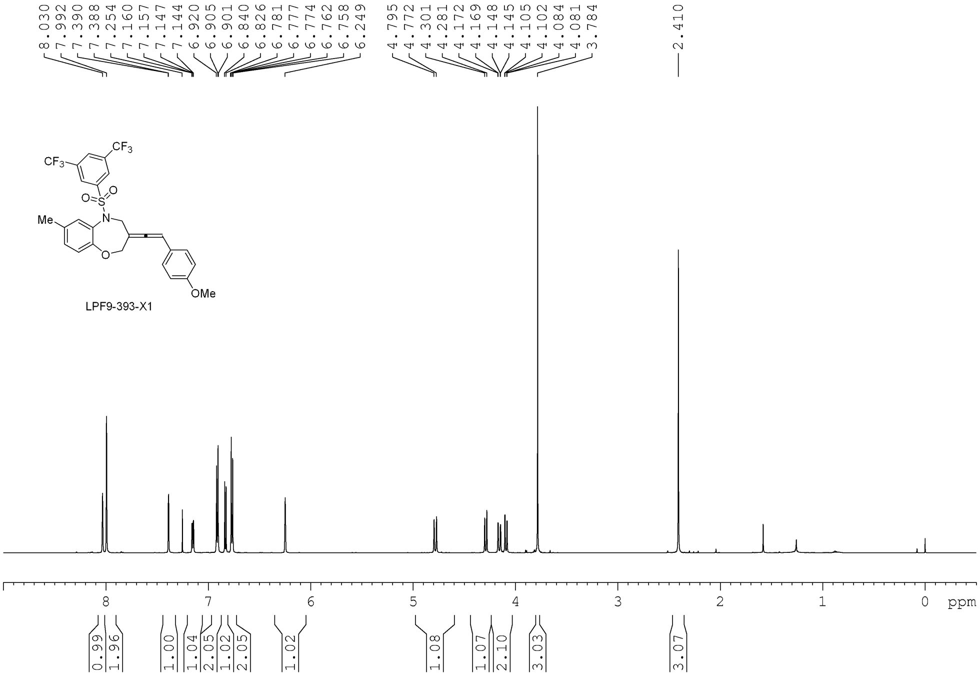

8.030
7.992
7.390
7.388
7.254
7.160
7.157
7.147
7.144
6.920
6.905
6.901
6.840
6.826
6.781
6.777
6.774
6.762
6.758
6.249
4.795
4.772
4.301
4.281
4.172
4.169
4.148
4.145
4.105
4.102
4.084
4.081
3.784
2.410
LPF9-393-X1
0.99
1.96
1.00
1.04
2.05
1.02
2.05
1.02
1.08
1.07
2.10
3.03
3.07
8
7
6
5
4
3
2
1
0
ppm

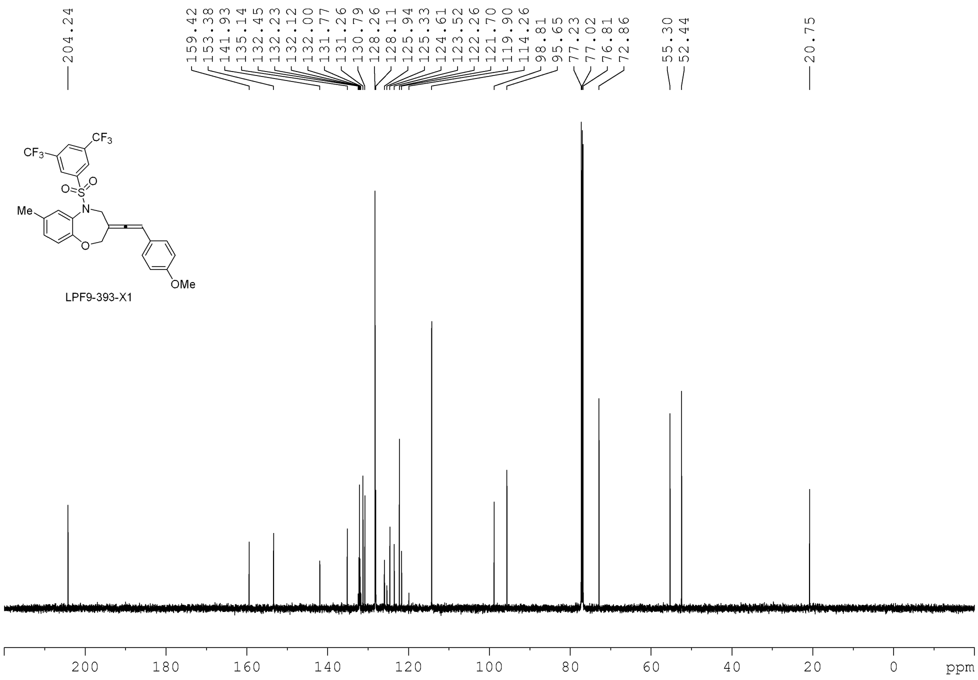

204.24
159.42
153.38
141.93
135.14
132.45
132.23
132.12
132.00
131.77
131.26
130.79
128.26
128.11
125.94
125.33
124.61
123.52
122.26
121.70
119.90
114.26
98.81
95.65
77.23
77.02
76.81
72.86
55.30
52.44
20.75
LPF9-393-X1
200
180
160
140
120
100
80
60
40
20
0
ppm

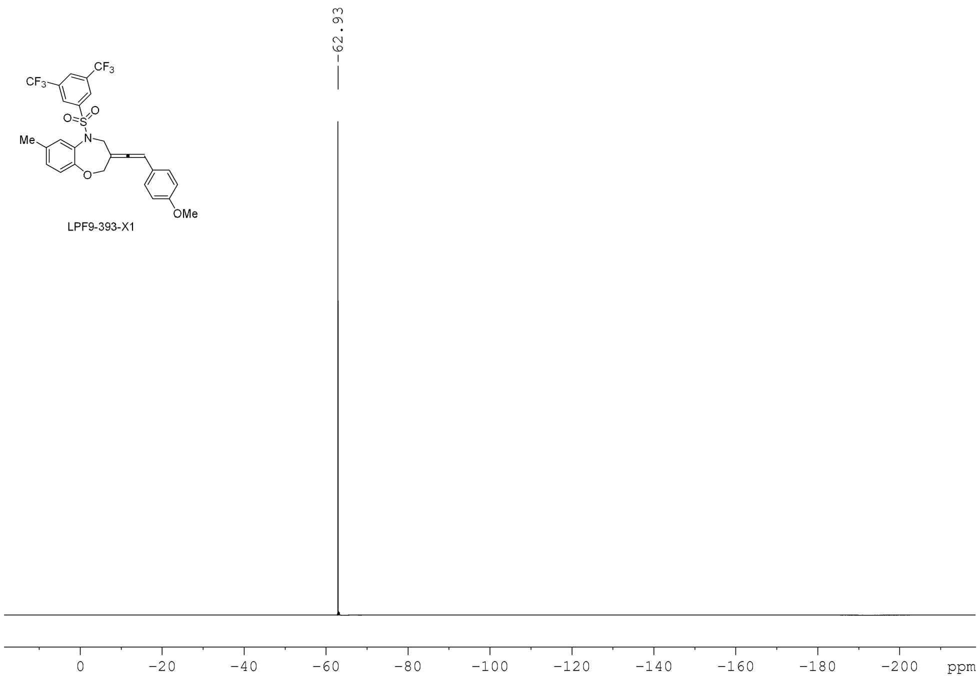
-62.93
CF_3
CF_3
O=S=O
N
Me
O
OMe
LPF9-393-X1
0 -20 -40 -60 -80 -100 -120 -140 -160 -180 -200 ppm

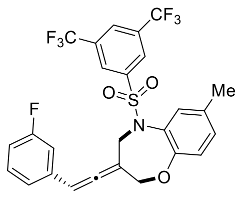

9i

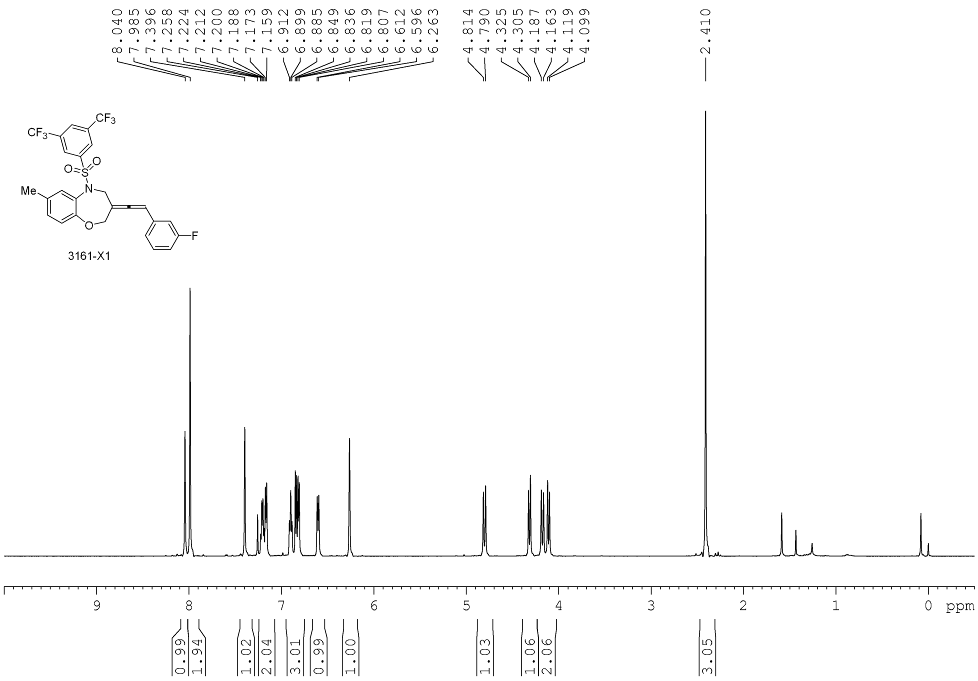

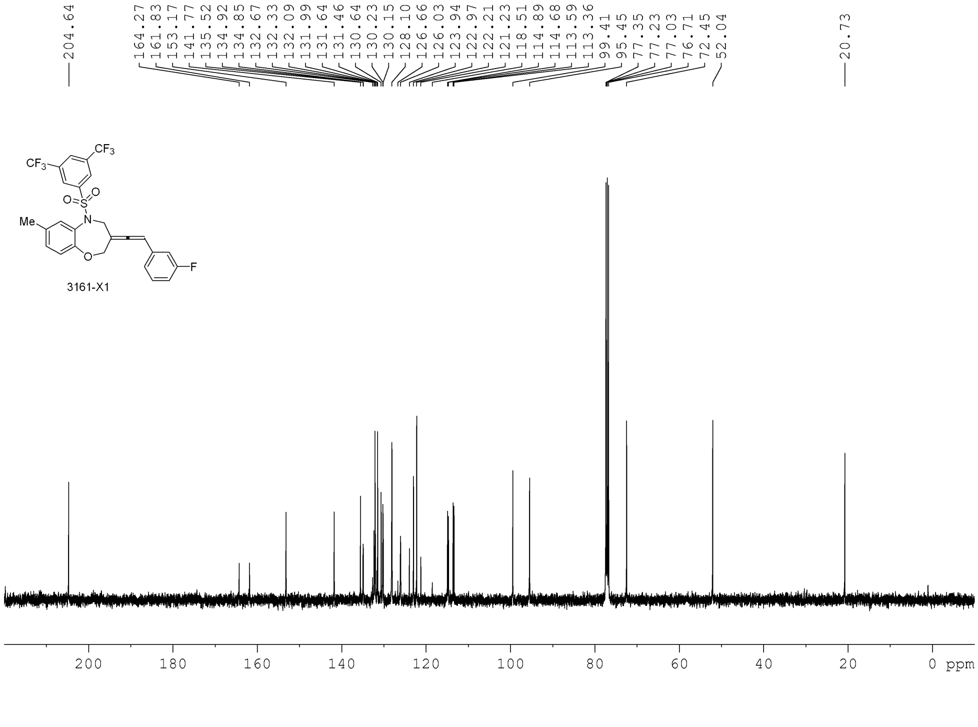

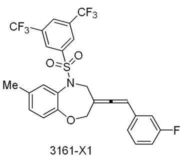
CF3
CF3
O
S
O
N
Me
O
F
3161-X1

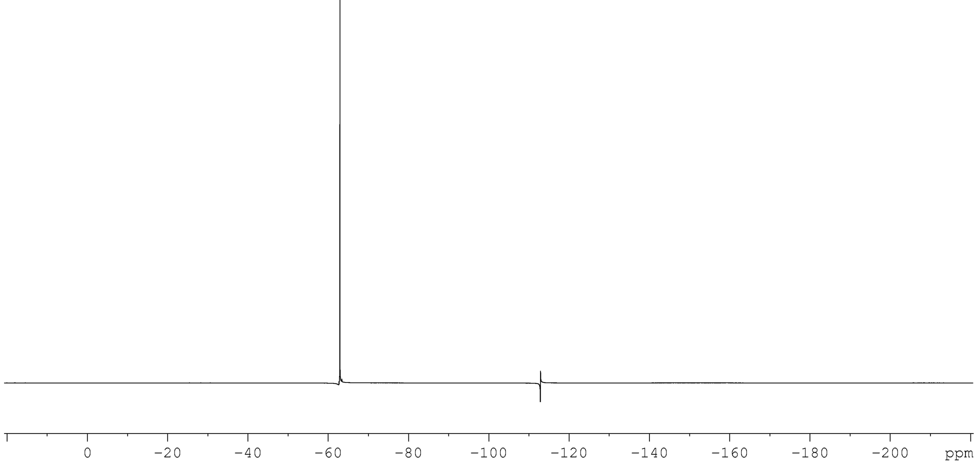
-62.94
-112.93
-112.95
0
-20
-40
-60
-80
-100
-120
-140
-160
-180
-200
ppm

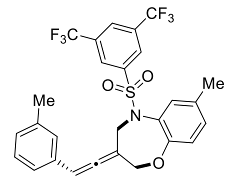

CF3
F3C
O=S=O
N
Me
Me
O

9j

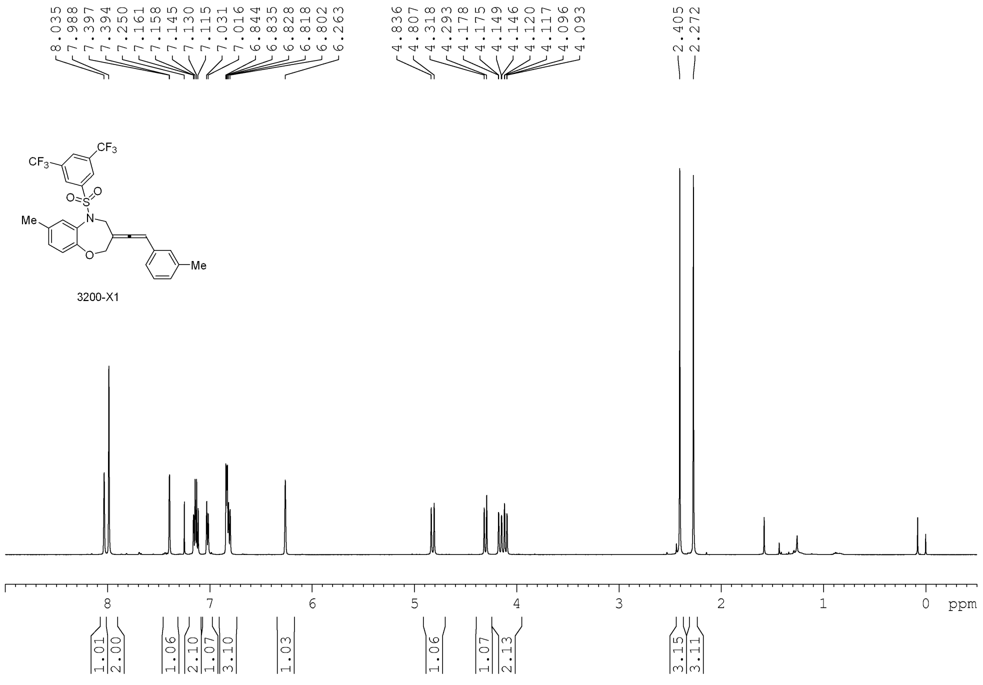

8.035
7.988
7.397
7.394
7.250
7.161
7.158
7.145
7.130
7.115
7.031
7.016
6.844
6.835
6.828
6.818
6.802
6.263
4.836
4.807
4.318
4.293
4.178
4.175
4.149
4.146
4.120
4.117
4.096
4.093
2.405
2.272
3200-X1
8 7 6 5 4 3 2 1 0 ppm
1.01
2.00
1.06
2.10
1.07
3.10
1.03
1.06
1.07
2.13
3.15
3.11

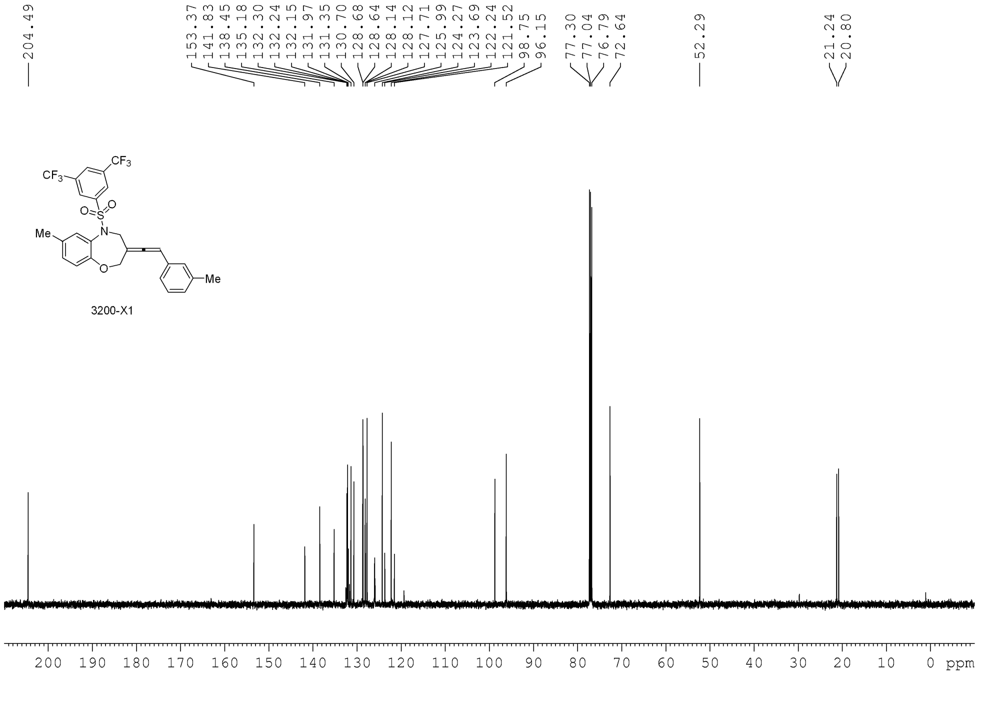

204.49
153.37
141.83
138.45
135.18
132.30
132.24
132.15
131.97
131.35
130.70
128.68
128.64
128.14
128.12
127.71
125.99
124.27
123.69
122.24
121.52
98.75
96.15
77.30
77.04
76.79
72.64
52.29
21.24
20.80
3200-X1
200 190 180 170 160 150 140 130 120 110 100 90 80 70 60 50 40 30 20 10 0 ppm

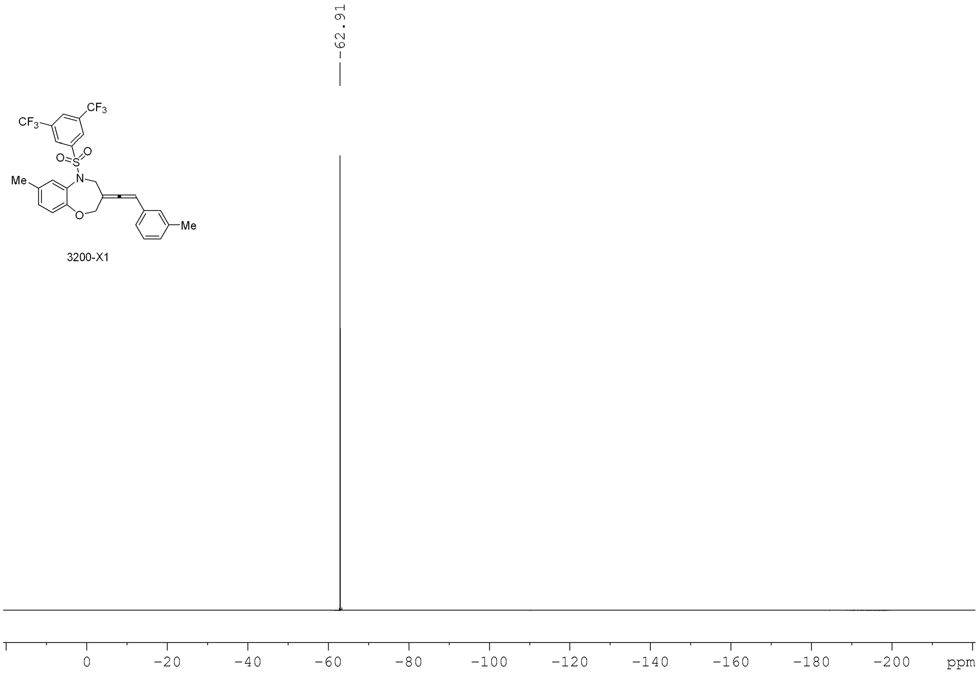
-62.91
CF3
CF3
O
S
O
N
Me
O
Me
3200-X1
0
-20
-40
-60
-80
-100
-120
-140
-160
-180
-200
ppm

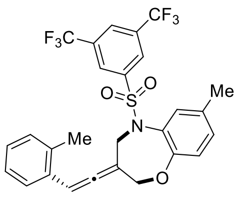

9k

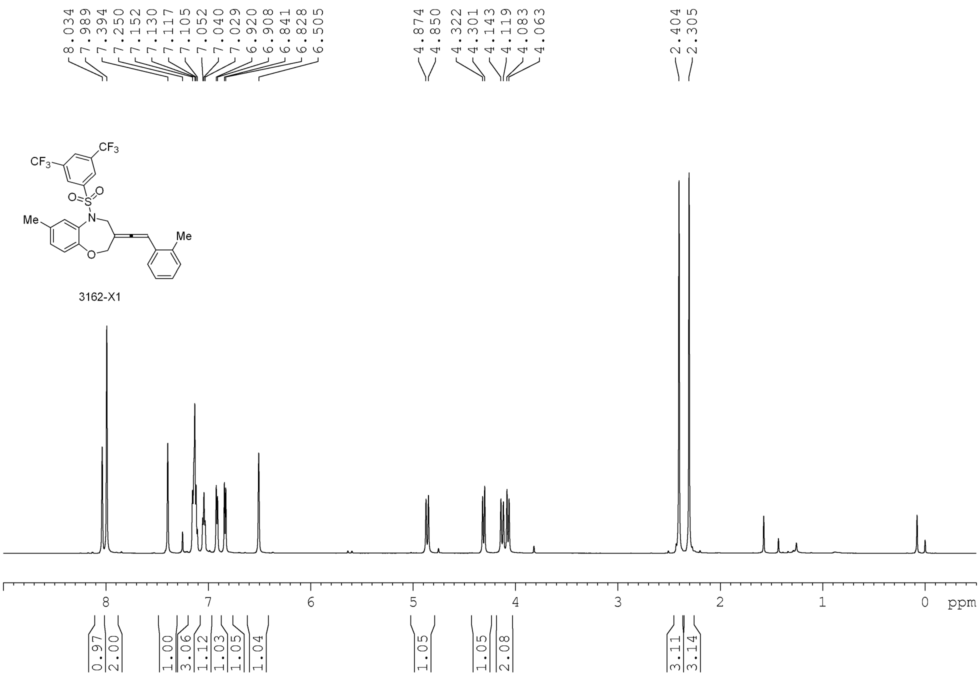

8.034
7.989
7.394
7.250
7.152
7.130
7.117
7.105
7.052
7.040
7.029
6.920
6.908
6.841
6.828
6.505
4.874
4.850
4.322
4.301
4.143
4.119
4.083
4.063
2.404
2.305
3162-X1
0.97
2.00
1.00
3.06
1.12
1.03
1.05
1.04
1.05
1.05
2.08
3.11
3.14
ppm

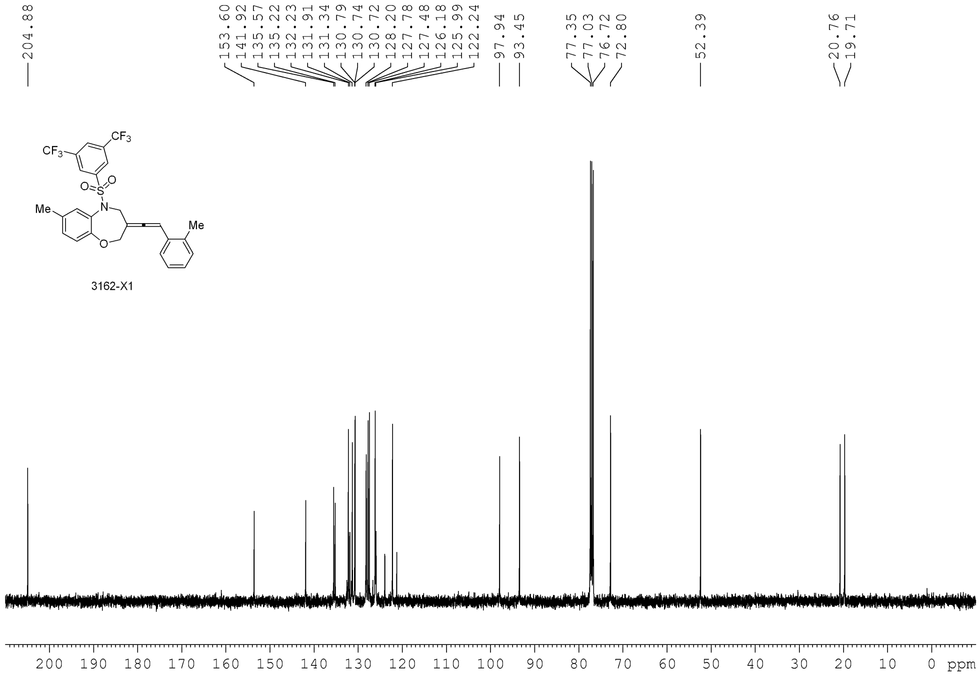

204.88
153.60
141.92
135.57
135.22
132.23
131.91
131.34
130.79
130.74
130.72
128.20
127.78
127.48
126.18
125.99
122.24
97.94
93.45
77.35
77.03
76.72
72.80
52.39
20.76
19.71
3162-X1
ppm

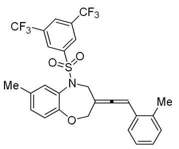

3162-X1

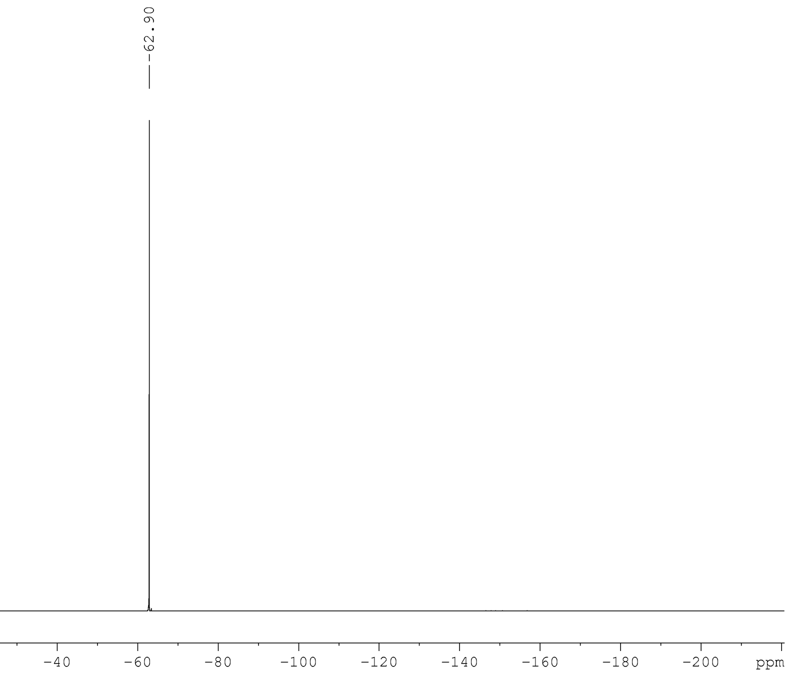

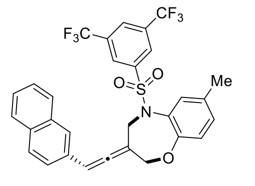
CF3
F3C
O S O
N
Me
O

9l

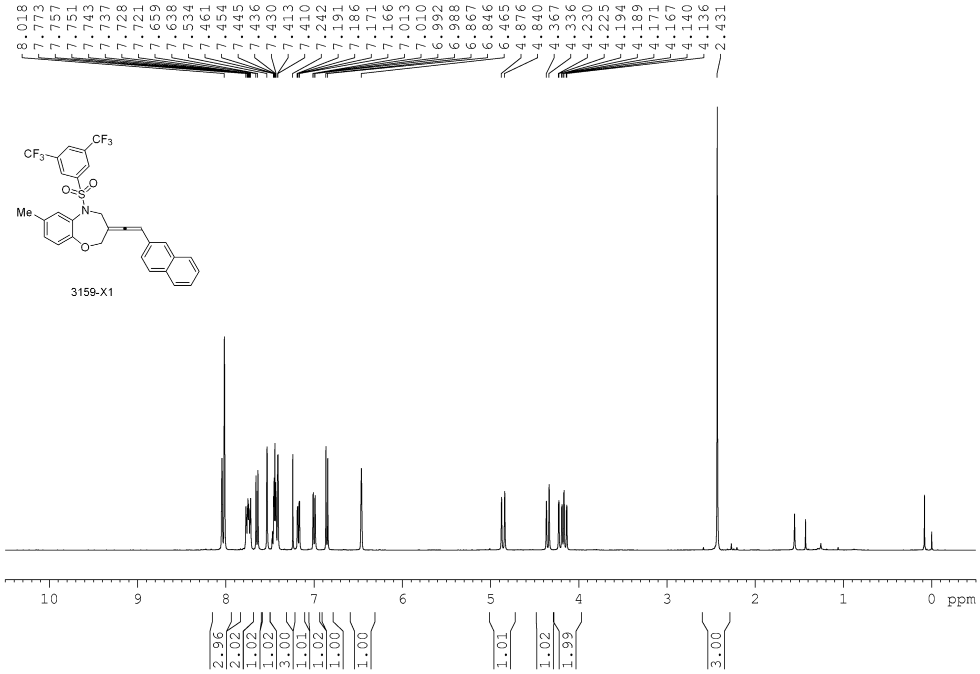
8.018
7.773
7.757
7.751
7.743
7.737
7.728
7.721
7.659
7.638
7.534
7.461
7.454
7.445
7.436
7.430
7.413
7.410
7.242
7.191
7.186
7.171
7.166
7.013
7.010
6.992
6.988
6.867
6.846
6.465
4.876
4.840
4.367
4.336
4.230
4.225
4.194
4.189
4.171
4.167
4.140
4.136
2.431
3159-X1
10 9 8 7 6 5 4 3 2 1 0 ppm
2.96
2.02
1.02
1.02
1.02
3.00
1.01
1.02
1.00
1.00
1.01
1.02
1.99
3.00

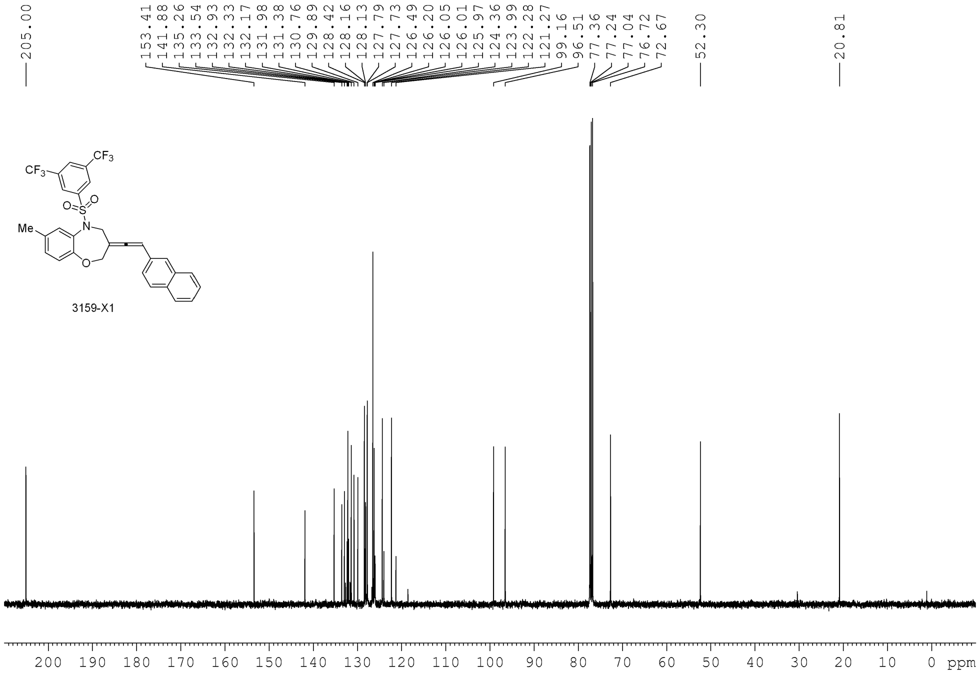
205.00
153.41
141.88
135.26
133.54
132.93
132.33
132.17
131.98
131.38
130.76
129.89
128.42
128.16
128.13
127.79
127.73
126.49
126.20
126.05
126.01
125.97
124.36
123.99
122.28
121.27
99.16
96.51
77.36
77.24
77.04
76.72
72.67
52.30
20.81
3159-X1
200 190 180 170 160 150 140 130 120 110 100 90 80 70 60 50 40 30 20 10 0 ppm

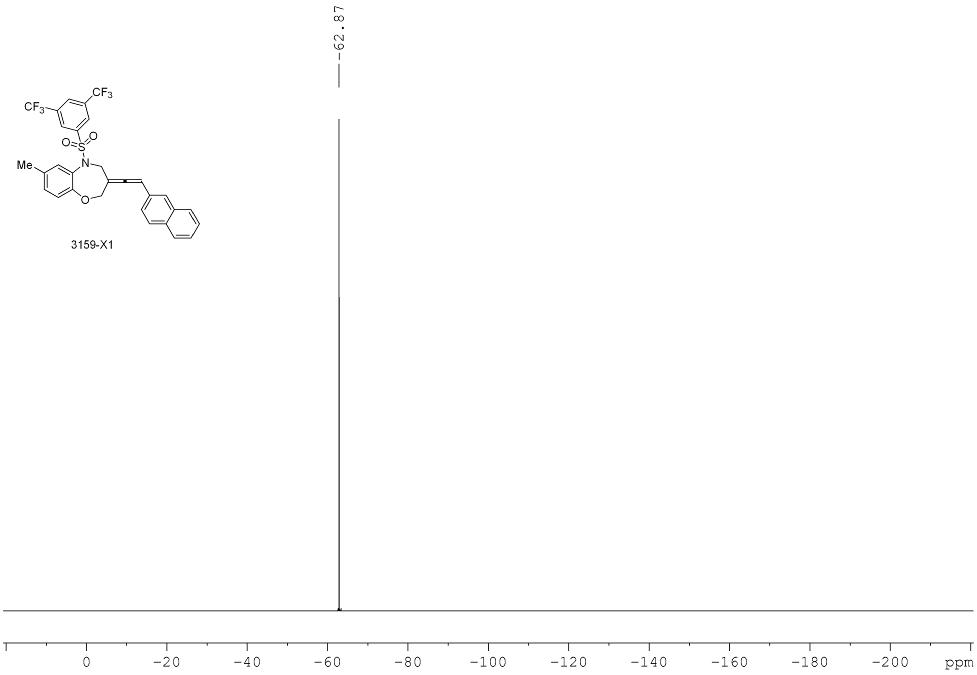
CF3
CF3
O
S
O
N
Me
O
3159-X1
-62.87
0
-20
-40
-60
-80
-100
-120
-140
-160
-180
-200
ppm

NMR spectra of 10

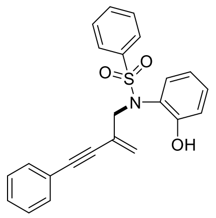

10a

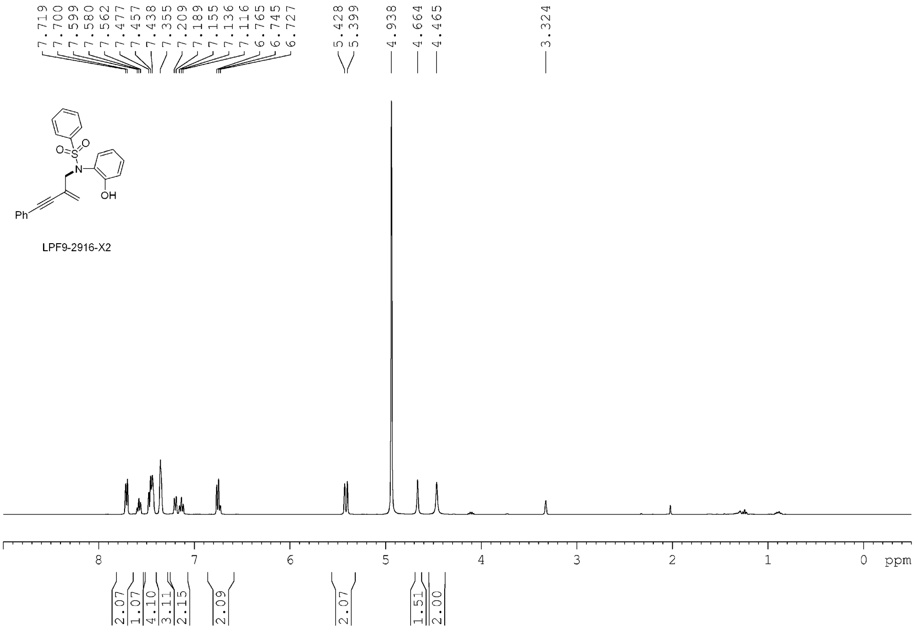

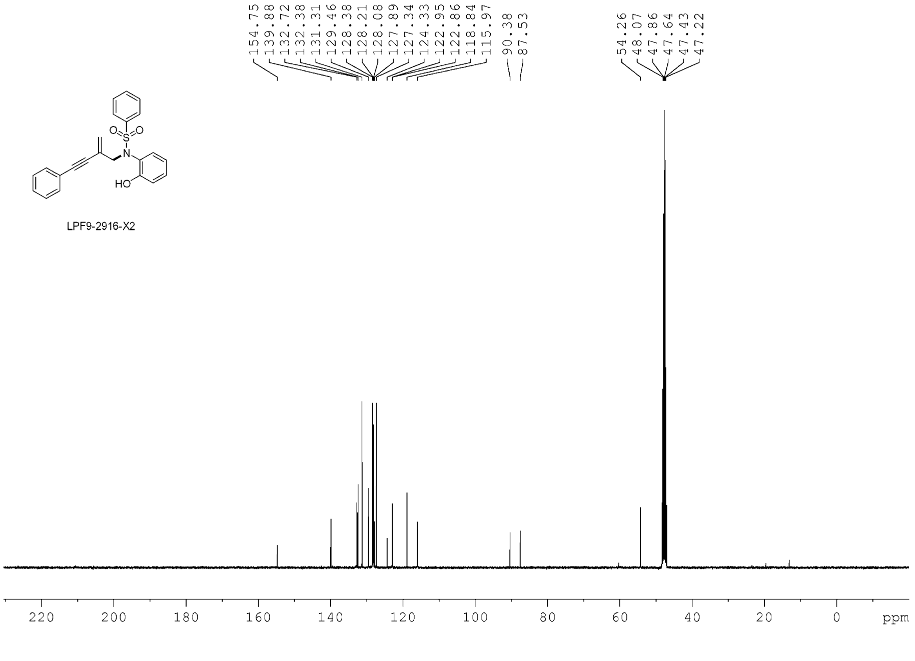

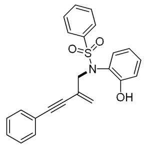

10a

DEPT 135

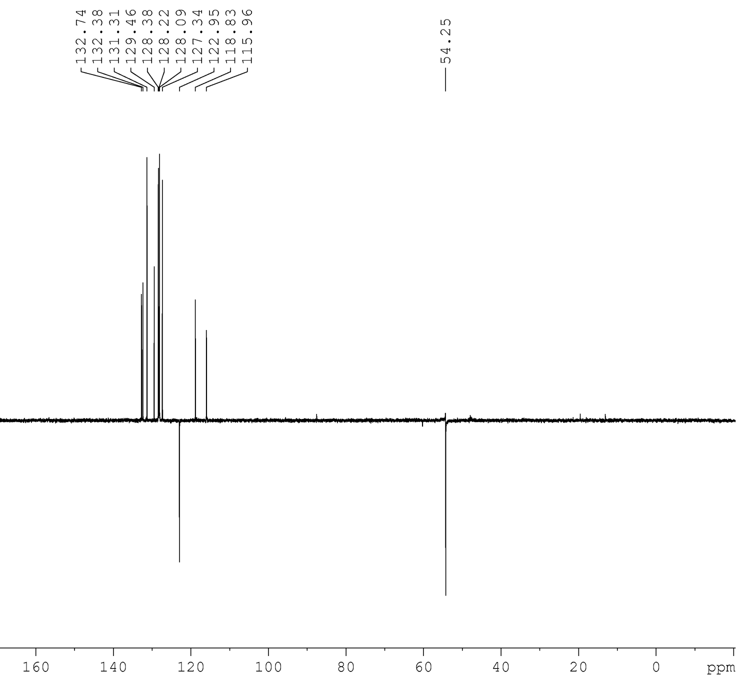
132.74
132.38
131.31
129.46
128.38
128.22
128.09
127.34
122.95
118.83
115.96
54.25
220 200 180 160 140 120 100 80 60 40 20 0 ppm

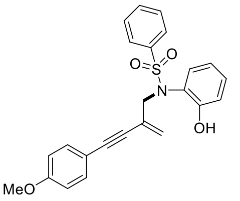

O
S
O
N
OH
MeO

10b

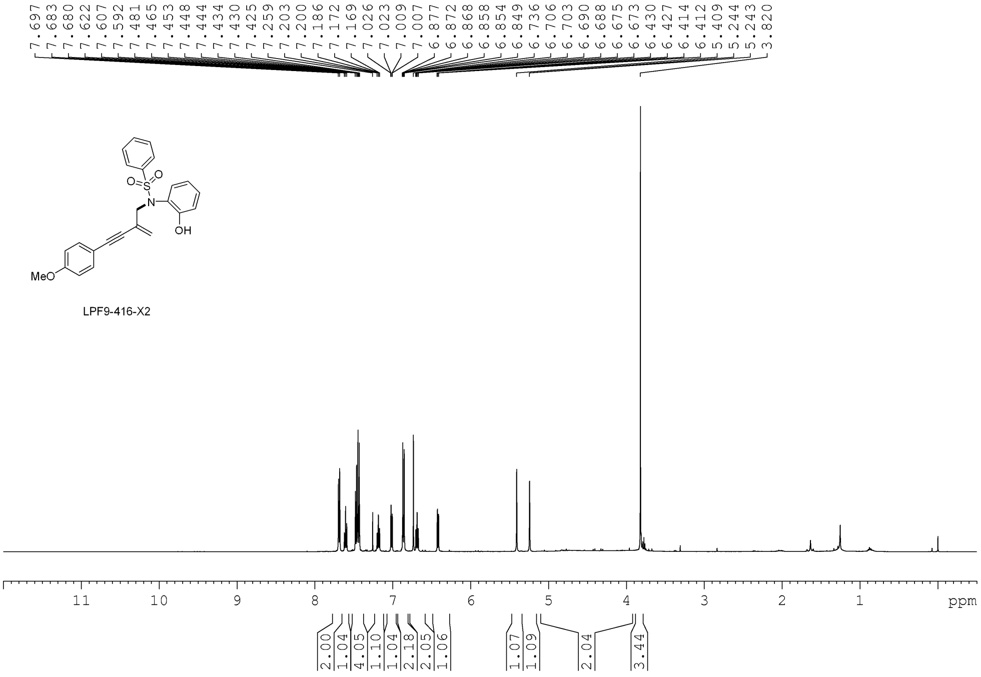

7.697
7.683
7.680
7.622
7.607
7.592
7.481
7.465
7.453
7.448
7.444
7.434
7.430
7.425
7.259
7.203
7.200
7.186
7.172
7.169
7.026
7.023
7.009
7.007
6.877
6.872
6.868
6.858
6.854
6.849
6.736
6.706
6.703
6.690
6.688
6.675
6.673
6.430
6.427
6.414
6.412
5.409
5.244
5.243
3.820
LPF9-416-X2
11
10
9
8
7
6
5
4
3
2
1
ppm
2.00
1.04
4.05
1.10
1.04
2.18
2.05
1.06
1.07
1.09
2.04
3.44

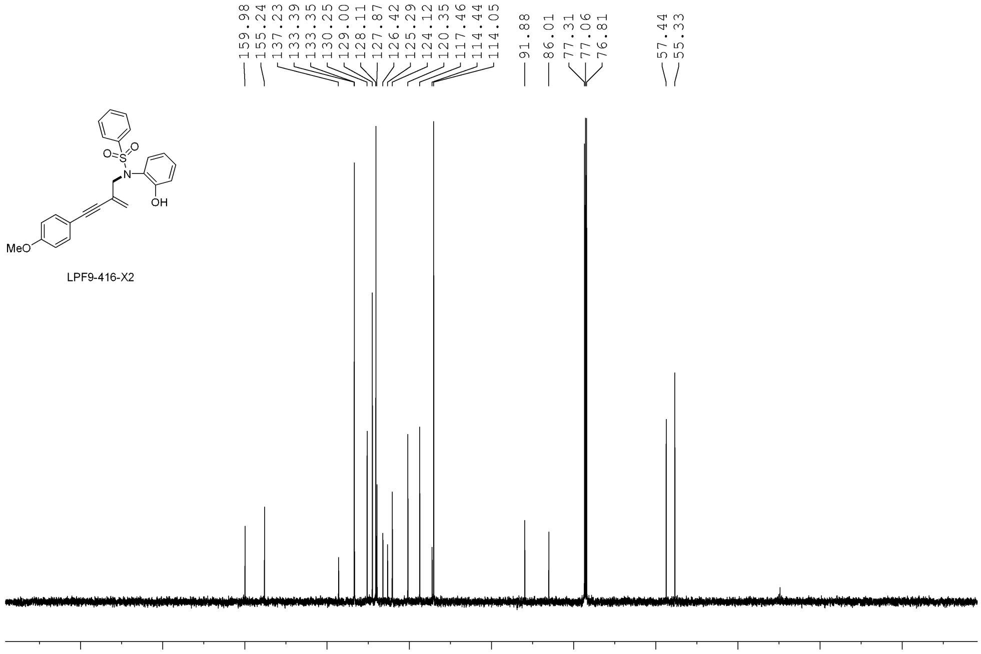

159.98
155.24
137.23
133.39
133.35
130.25
129.00
128.11
127.87
126.42
125.29
124.12
120.35
117.46
114.44
114.05
91.88
86.01
77.31
77.06
76.81
57.44
55.33
LPF9-416-X2
200
180
160
140
120
100
80
60
40
20
0
ppm

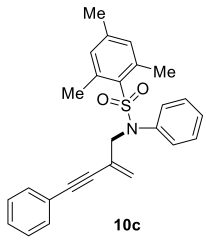
Me
Me
Me
O
S
O
N

10c

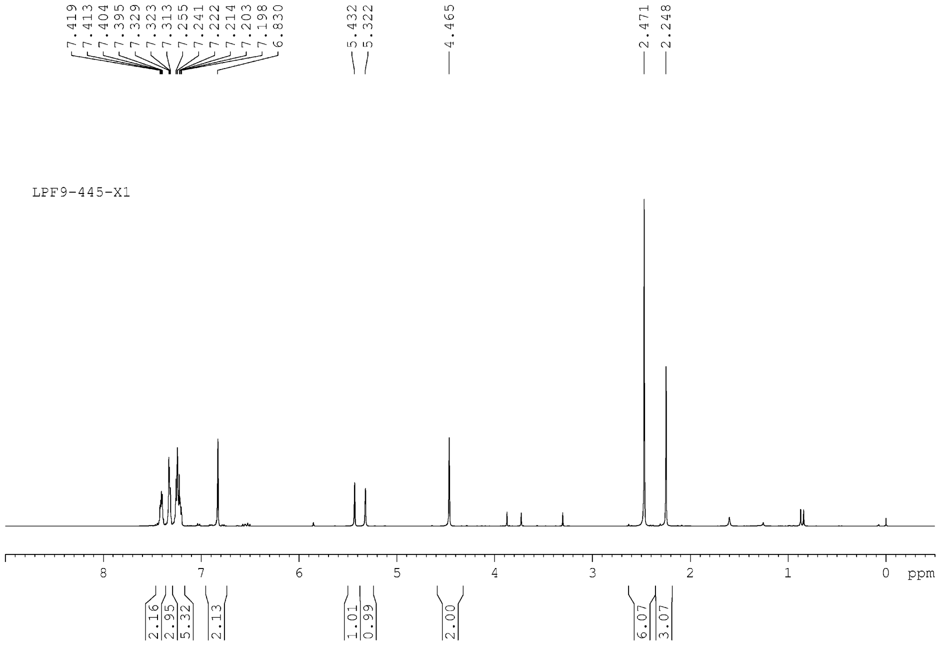
7.419
7.413
7.404
7.395
7.329
7.323
7.313
7.255
7.241
7.222
7.214
7.203
7.198
6.830
5.432
5.322
4.465
2.471
2.248
LPF9-445-X1
8
7
6
5
4
3
2
1
0 ppm
2.16
2.95
5.32
2.13
1.01
0.99
2.00
6.07
3.07

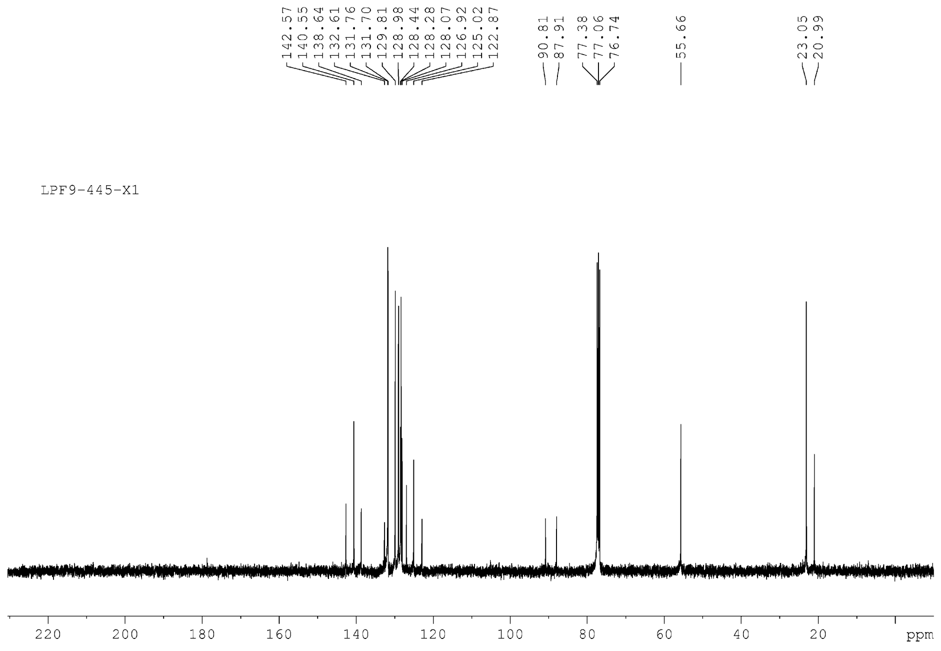
142.57
140.55
138.64
132.61
131.76
131.70
129.81
128.98
128.44
128.28
128.07
126.92
125.02
122.87
90.81
87.91
77.38
77.06
76.74
55.66
23.05
20.99
LPF9-445-X1
220
200
180
160
140
120
100
80
60
40
20
ppm

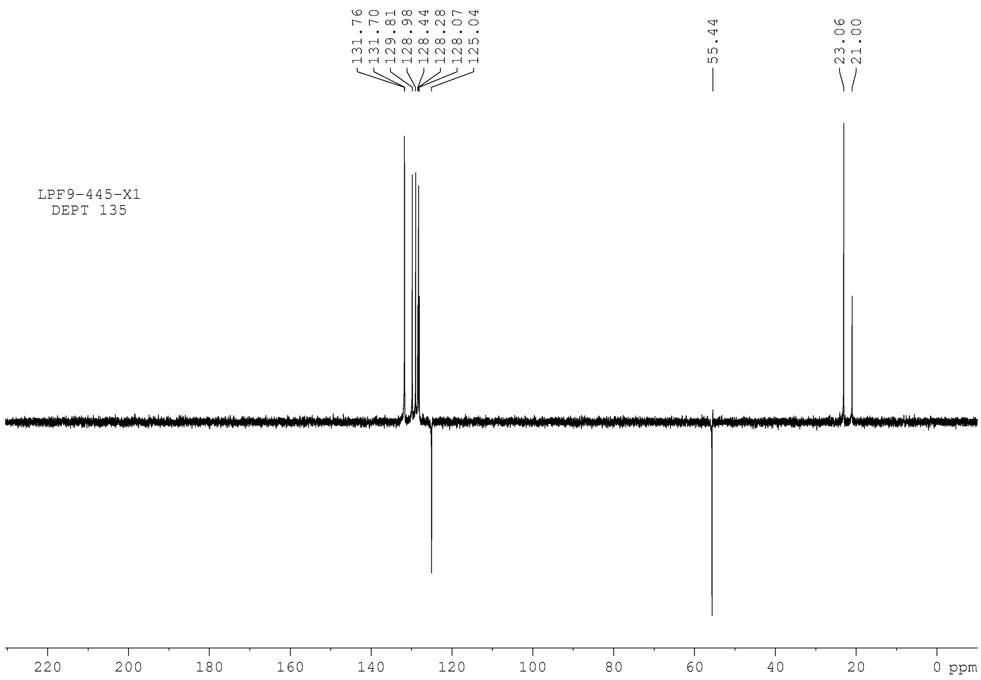
131.76
131.70
129.81
128.98
128.44
128.28
128.07
125.04
55.44
23.06
21.00
LPF9-445-X1
DEPT 135
220
200
180
160
140
120
100
80
60
40
20
0 ppm

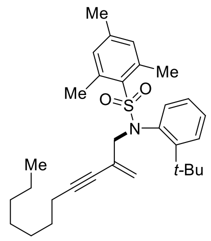
Me
Me
Me
O=S=O
N
Me
t-Bu

10d, 83%

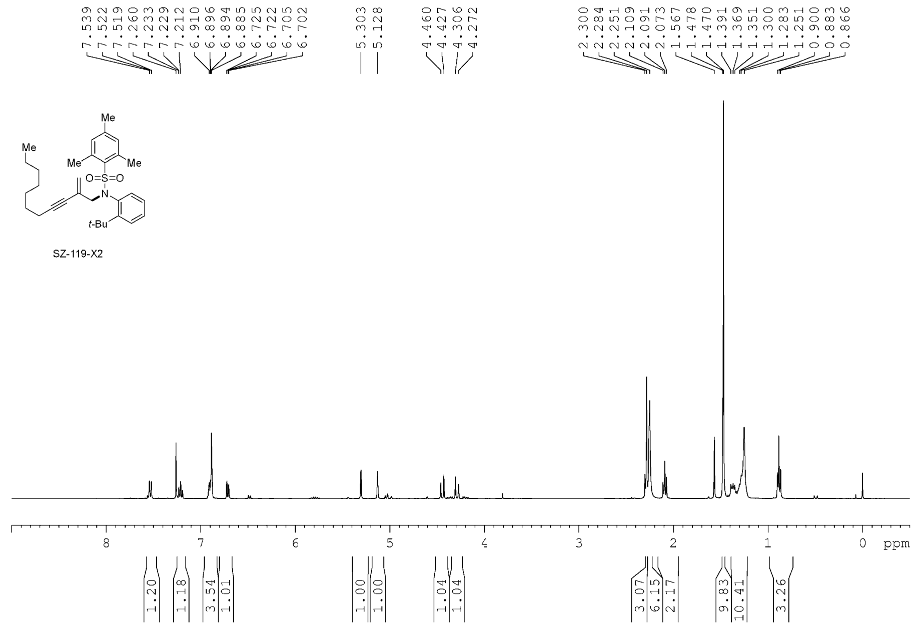
7.539
7.522
7.519
7.260
7.233
7.229
7.212
6.910
6.896
6.894
6.885
6.725
6.722
6.705
6.702
5.303
5.128
4.460
4.427
4.306
4.272
2.300
2.284
2.251
2.109
2.091
2.073
1.567
1.478
1.470
1.391
1.369
1.351
1.300
1.283
1.251
0.900
0.883
0.866
SZ-119-X2
8
7
6
5
4
3
2
1
0 ppm
1.20
1.18
3.54
1.01
1.00
1.00
1.04
1.04
3.07
6.15
2.17
9.83
10.41
3.26

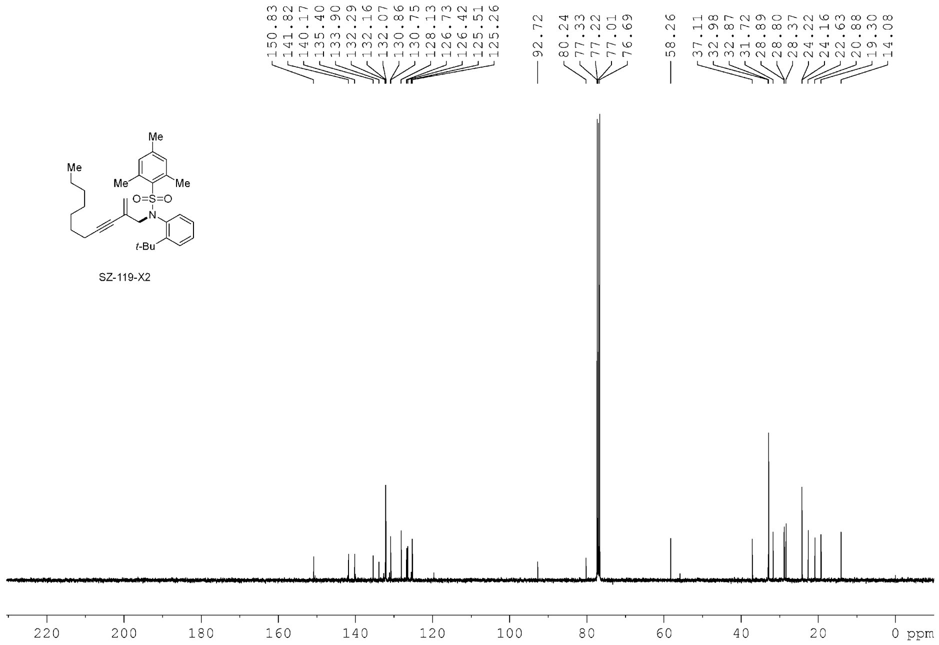
150.83
141.82
140.17
135.40
133.90
132.29
132.16
132.07
130.86
130.75
128.13
126.73
126.42
125.51
125.26
92.72
80.24
77.33
77.22
77.01
76.69
58.26
37.11
32.98
32.87
31.72
28.89
28.80
28.37
24.22
24.16
22.63
20.88
19.30
14.08
SZ-119-X2
220
200
180
160
140
120
100
80
60
40
20
0 ppm

NMR spectra of 13

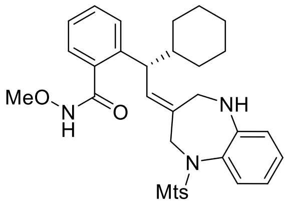

(*R*,*E*)-**13**

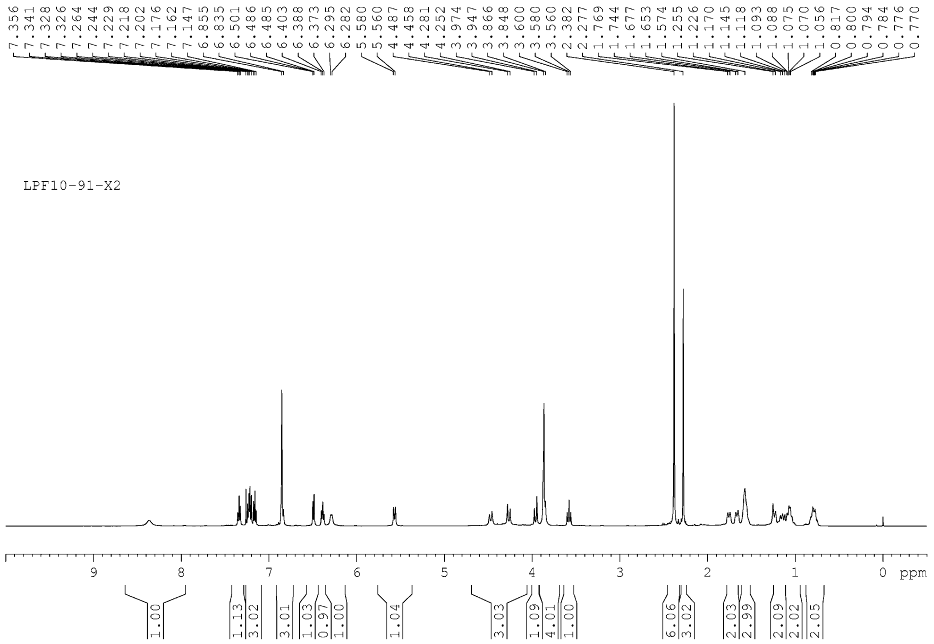

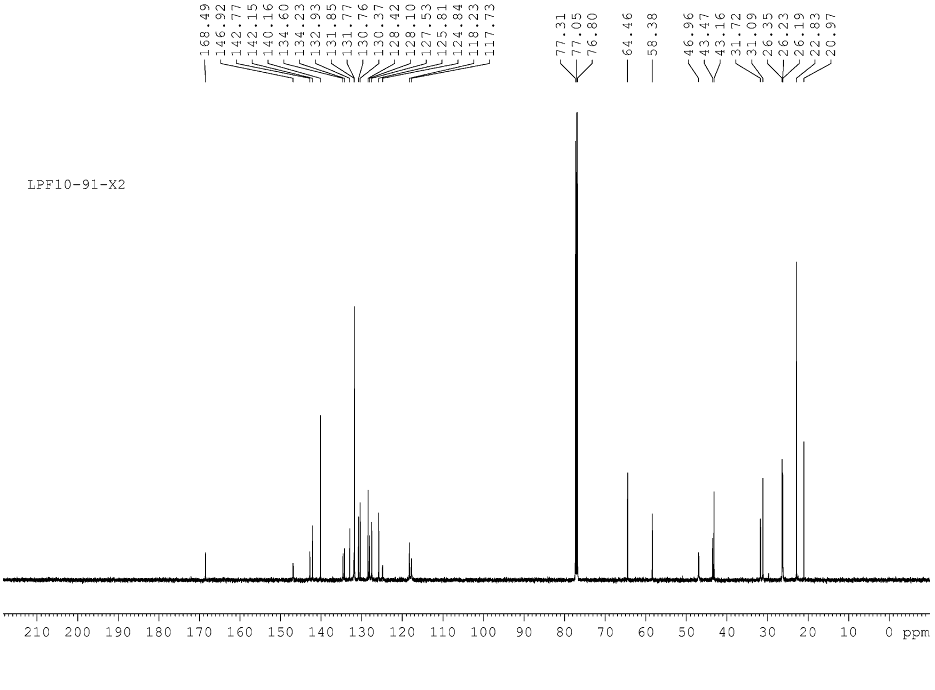

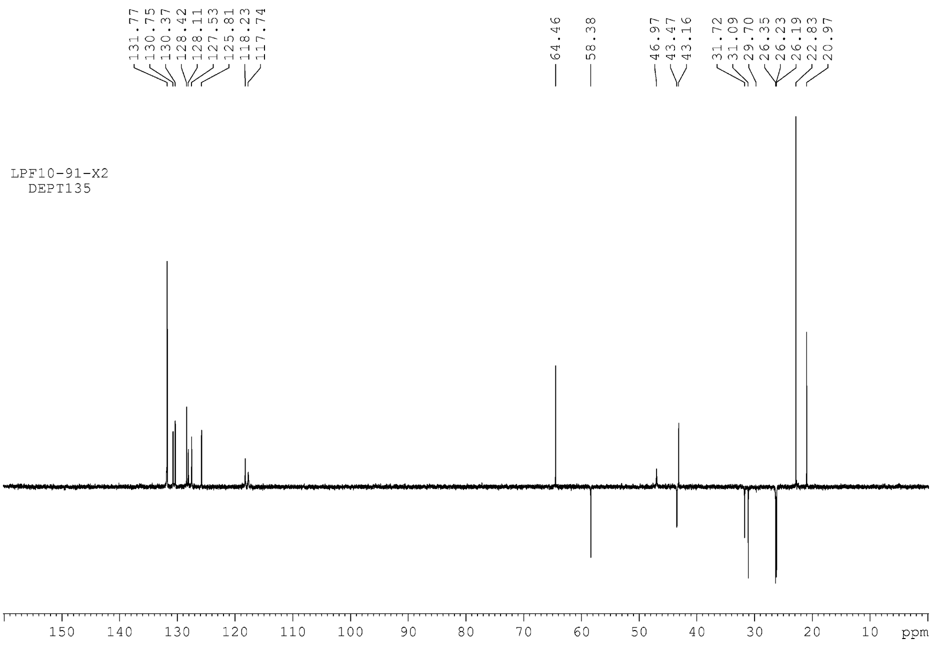
131.77
130.75
130.37
128.42
128.11
127.53
125.81
118.23
117.74
64.46
58.38
46.97
43.47
43.16
31.72
31.09
29.70
26.35
26.23
26.19
22.83
20.97
LPF10-91-X2
DEPT135
150
140
130
120
110
100
90
80
70
60
50
40
30
20
10
ppm

NMR spectra of 15

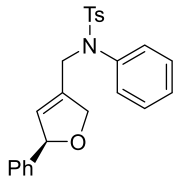

(*R*)-**15**

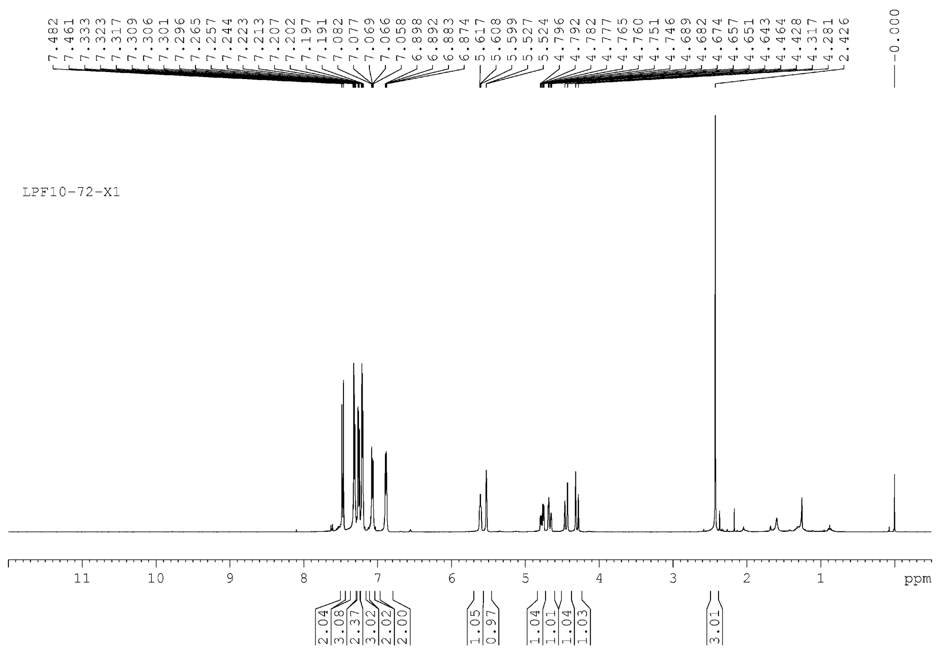

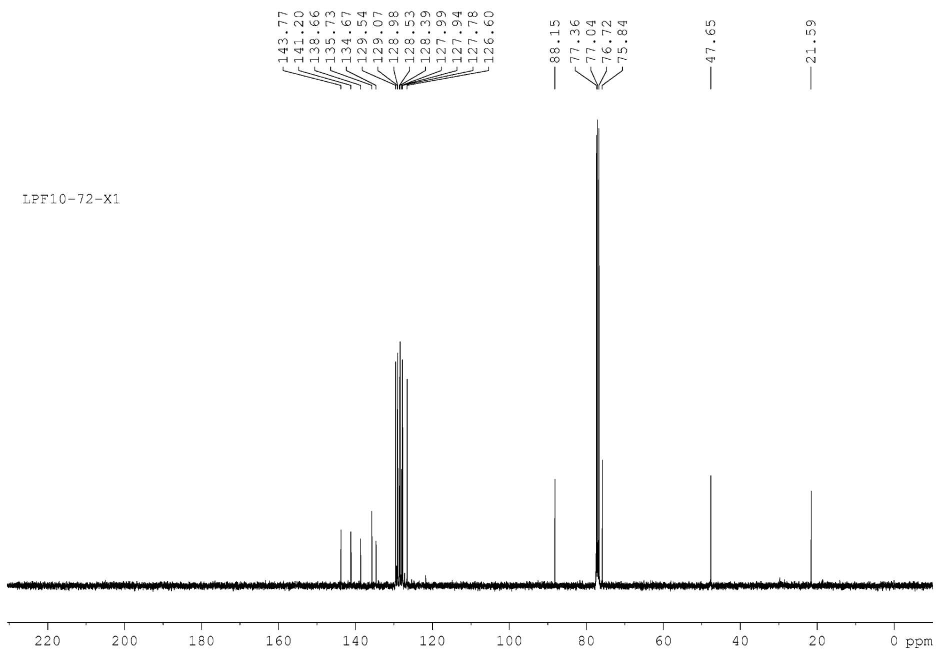

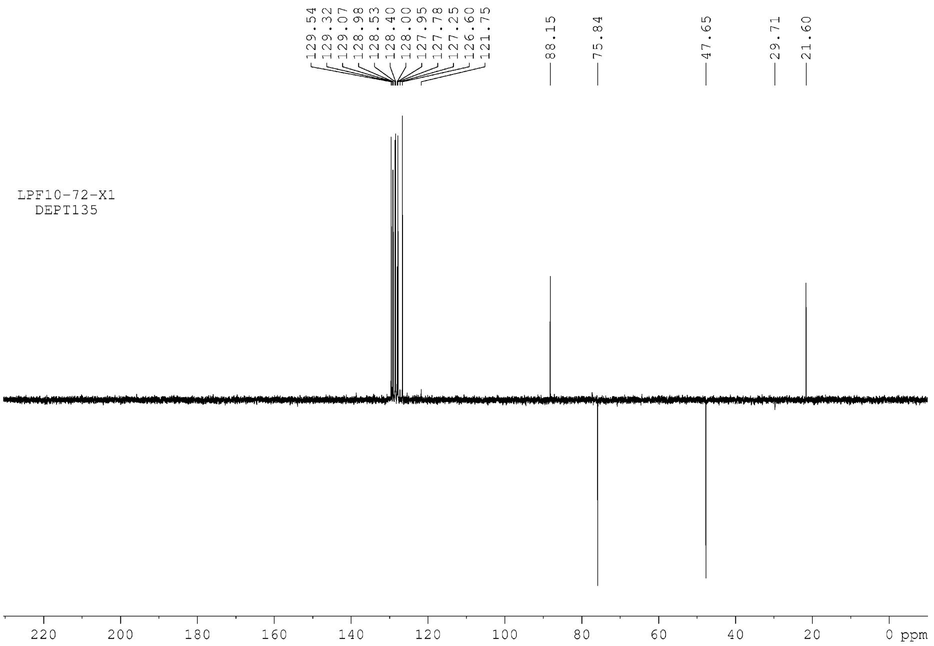

129.54
129.32
129.07
128.98
128.53
128.40
128.00
127.95
127.78
127.25
126.60
121.75
88.15
75.84
47.65
29.71
21.60
LPF10-72-X1
DEPT135
220
200
180
160
140
120
100
80
60
40
20
0 ppm

11. HPLC spectra

HPLC spectra of 3

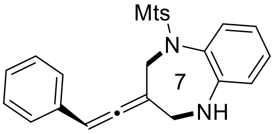

3a

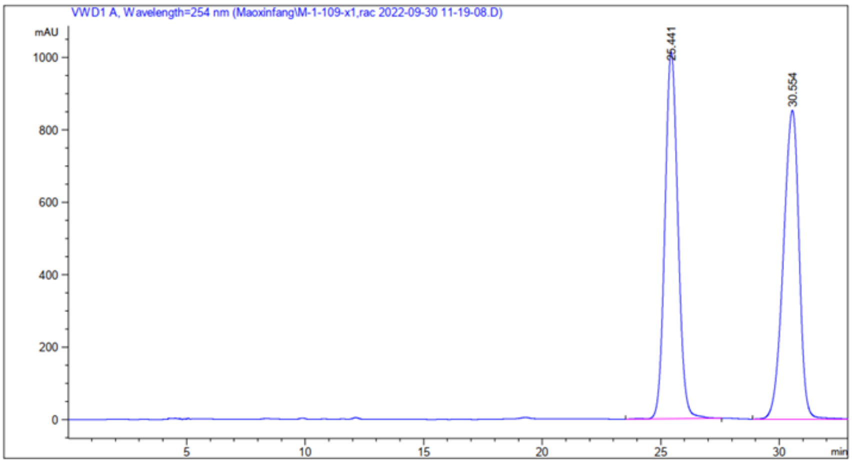

Peak #	RetTime [min]	Type	Width [min]	Area [mAU*s]	Height [mAU]	Area %
1	25.441	VB R	0.5935	3.86090e4	1013.44830	49.5690
2	30.554	BB	0.7243	3.92803e4	852.95270	50.4310

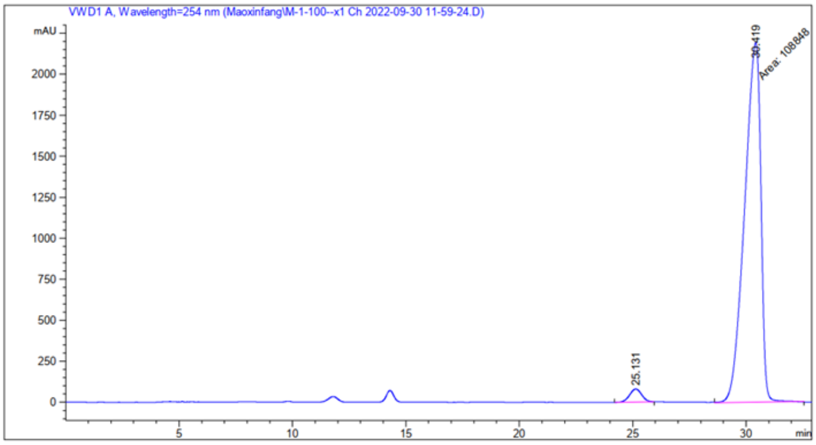

Peak #	RetTime [min]	Type	Width [min]	Area [mAU*s]	Height [mAU]	Area %
1	25.131	BB	0.5749	2900.77759	79.45480	2.5958
2	30.419	MM	0.8273	1.08848e5	2192.87207	97.4042

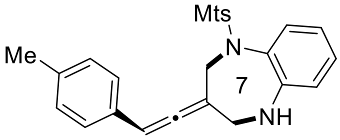

3b

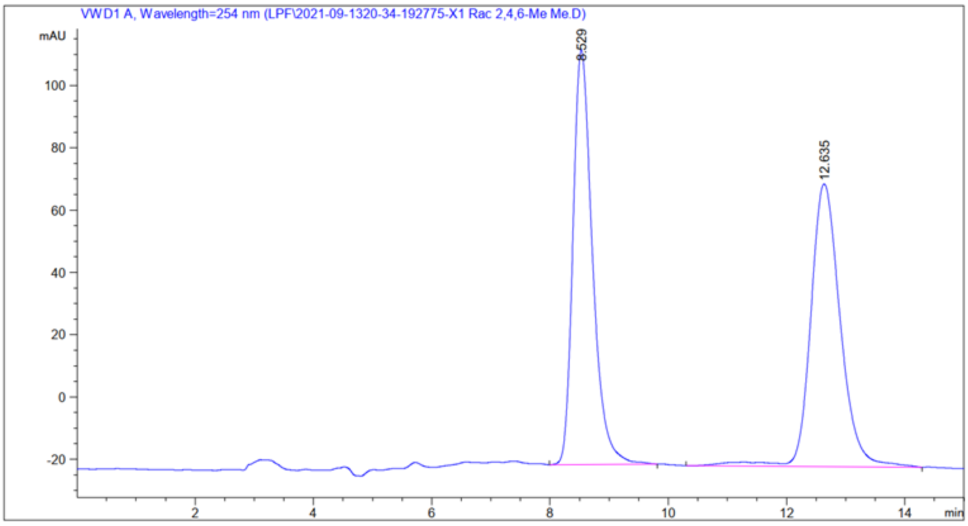

Peak #	RetTime [min]	Type	Width [min]	Area [mAU*s]	Height [mAU]	Area %
1	8.529	BB	0.3495	3053.55713	133.17072	48.9491
2	12.635	VB R	0.5344	3184.66870	90.75445	51.0509

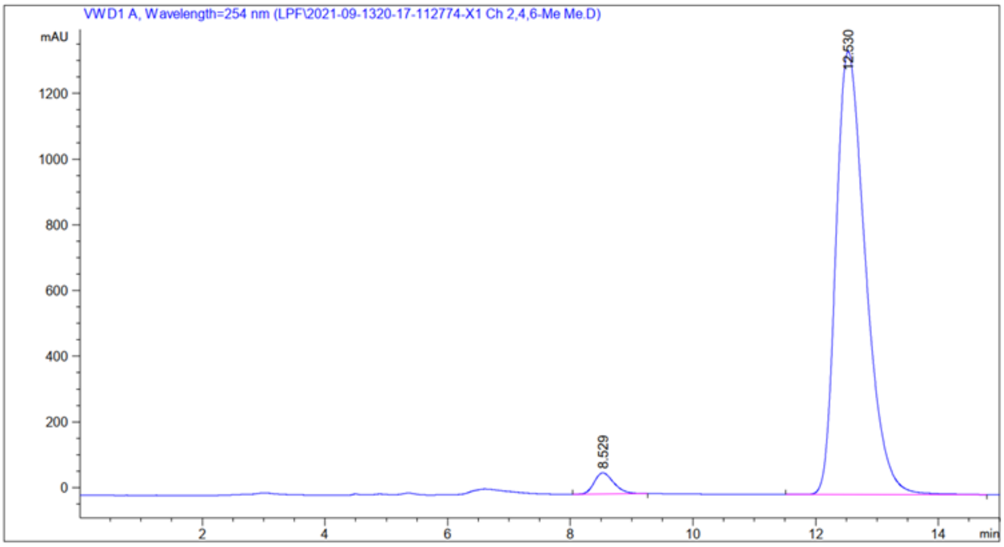

Peak #	RetTime [min]	Type	Width [min]	Area [mAU*s]	Height [mAU]	Area %
1	8.529	BB	0.3410	1434.49182	64.61596	3.0780
2	12.530	BB	0.5145	4.51694e4	1349.35278	96.9220

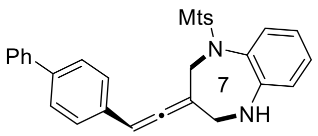

3c

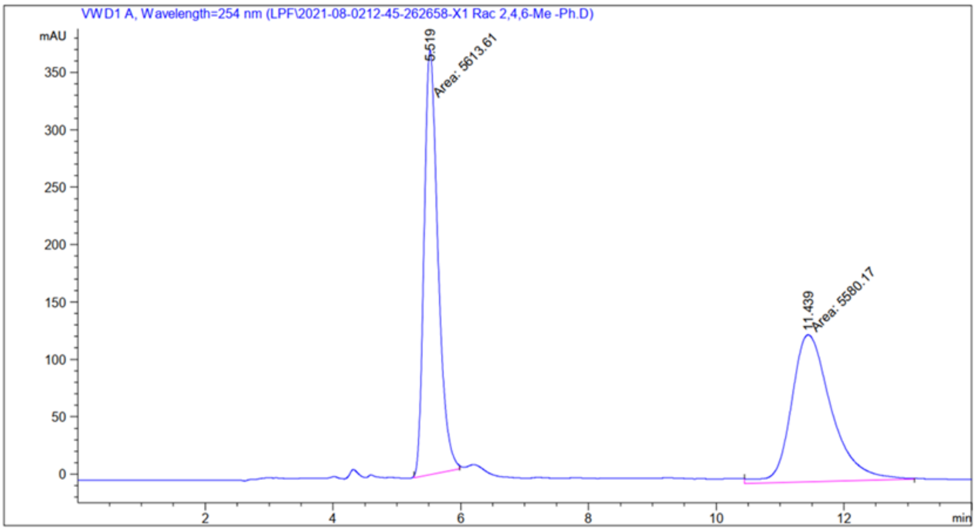

Peak #	RetTime [min]	Type	Width [min]	Area [mAU*s]	Height [mAU]	Area %
1	5.519	MM	0.2528	5613.60596	370.07678	50.1493
2	11.439	MM	0.7262	5580.17480	128.07329	49.8507

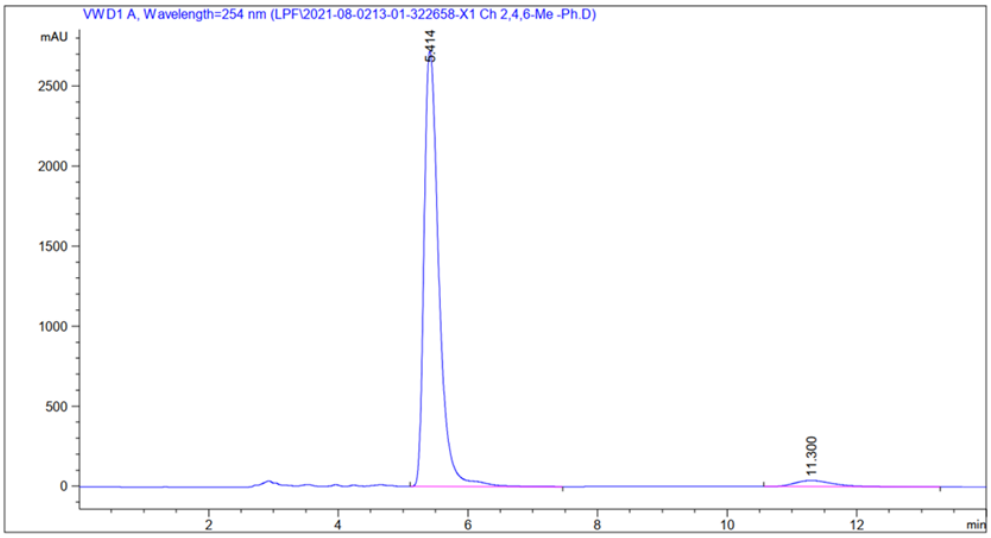

Peak #	RetTime [min]	Type	Width [min]	Area [mAU*s]	Height [mAU]	Area %
1	5.414	BB	0.2432	4.32033e4	2721.83691	96.4589
2	11.300	BV R	0.6364	1586.05066	38.27554	3.5411

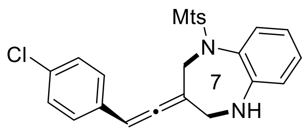

3d

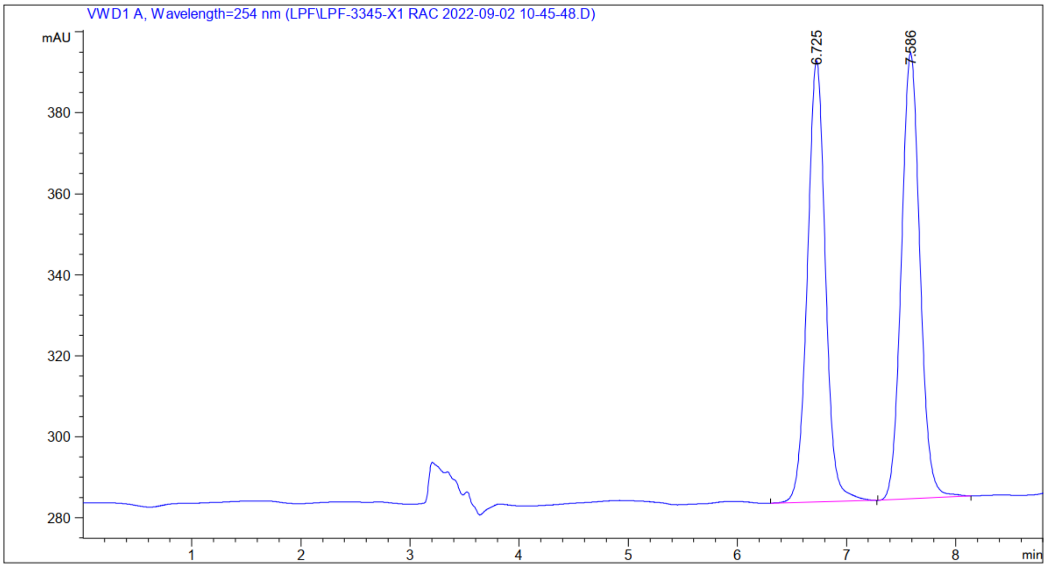

Peak #	RetTime [min]	Type	Width [min]	Area [mAU*s]	Height [mAU]	Area %
1	6.725	BB	0.1771	1253.70667	109.08978	49.0479
2	7.586	BB	0.1829	1302.37878	110.17856	50.9521

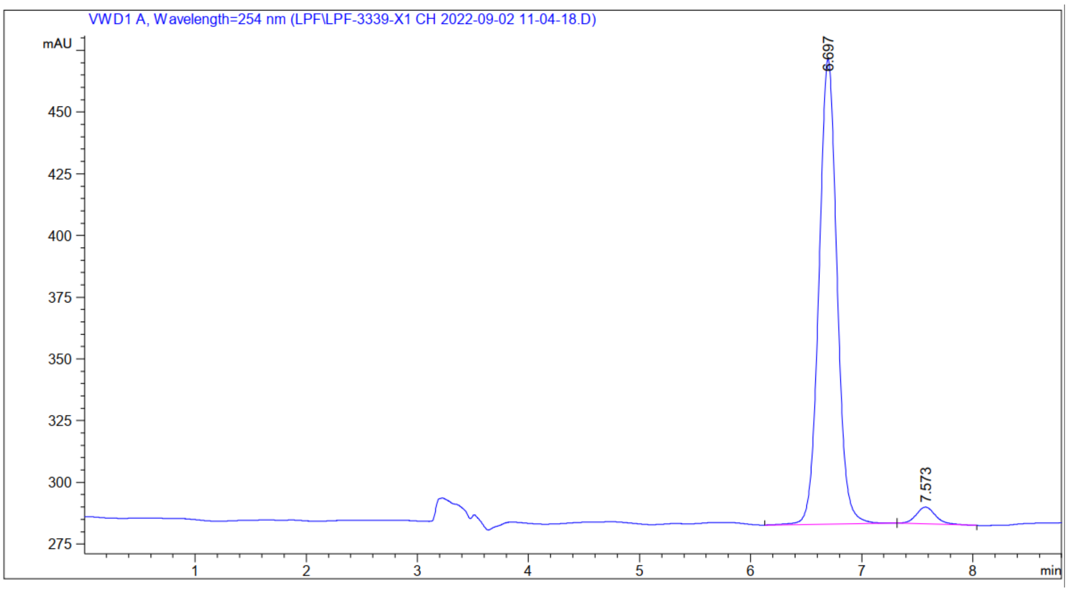

Peak #	RetTime [min]	Type	Width [min]	Area [mAU*s]	Height [mAU]	Area %
1	6.697	BB	0.1732	2120.05249	188.45082	96.2423
2	7.573	BB	0.1871	82.77594	6.79612	3.7577

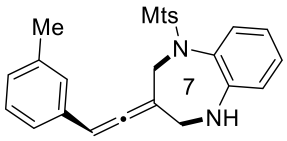

3e

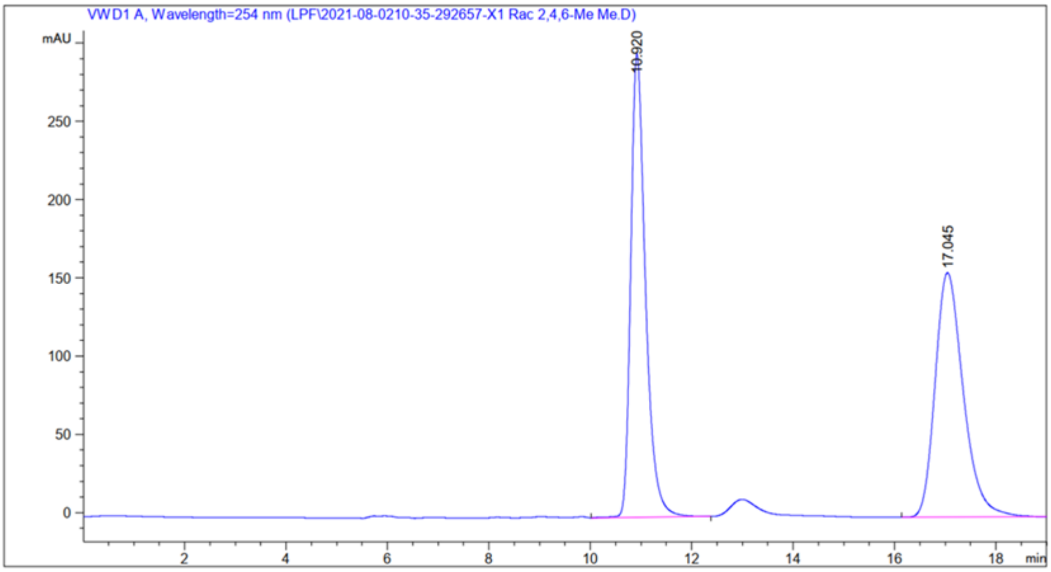

Peak #	RetTime [min]	Type	Width [min]	Area [mAU*s]	Height [mAU]	Area %
1	10.920	BB	0.3060	5993.66943	296.26468	49.8202
2	17.045	BB	0.5910	6036.92236	156.16983	50.1798

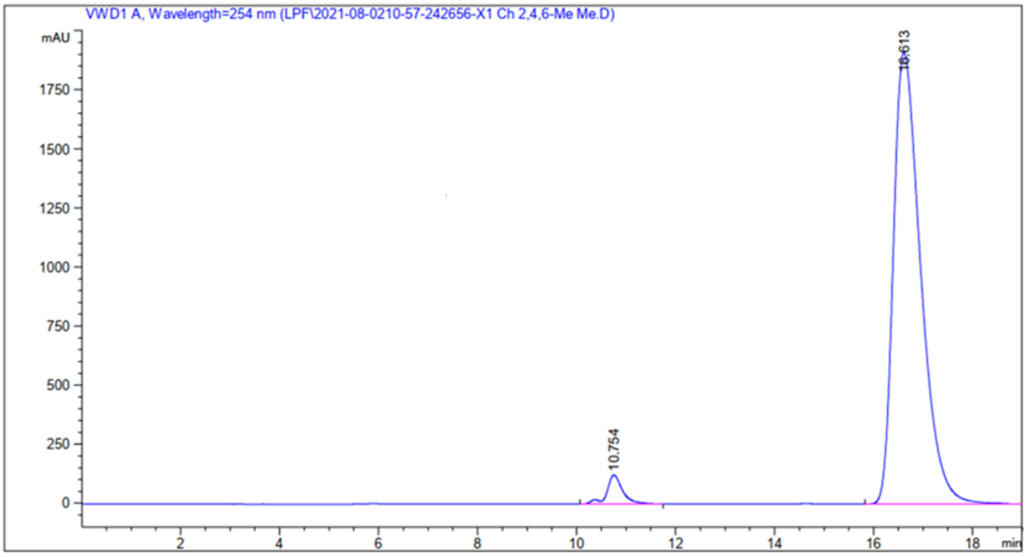

Peak #	RetTime [min]	Type	Width [min]	Area [mAU*s]	Height [mAU]	Area %
1	10.754	VB R	0.3031	2694.65112	122.75300	3.5508
2	16.613	BBA	0.5869	7.31944e4	1910.63049	96.4492

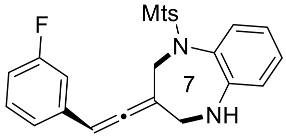

3f

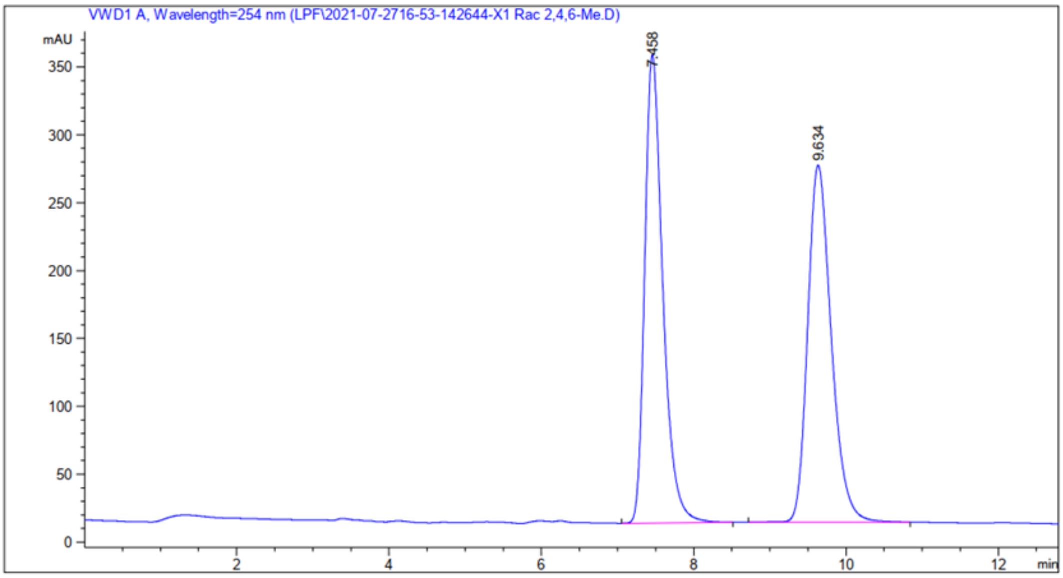

Peak #	RetTime [min]	Type	Width [min]	Area [mAU*s]	Height [mAU]	Area %
1	7.458	BB	0.2505	5691.47607	345.00690	50.1586
2	9.634	BB	0.3289	5655.47656	262.94412	49.8414

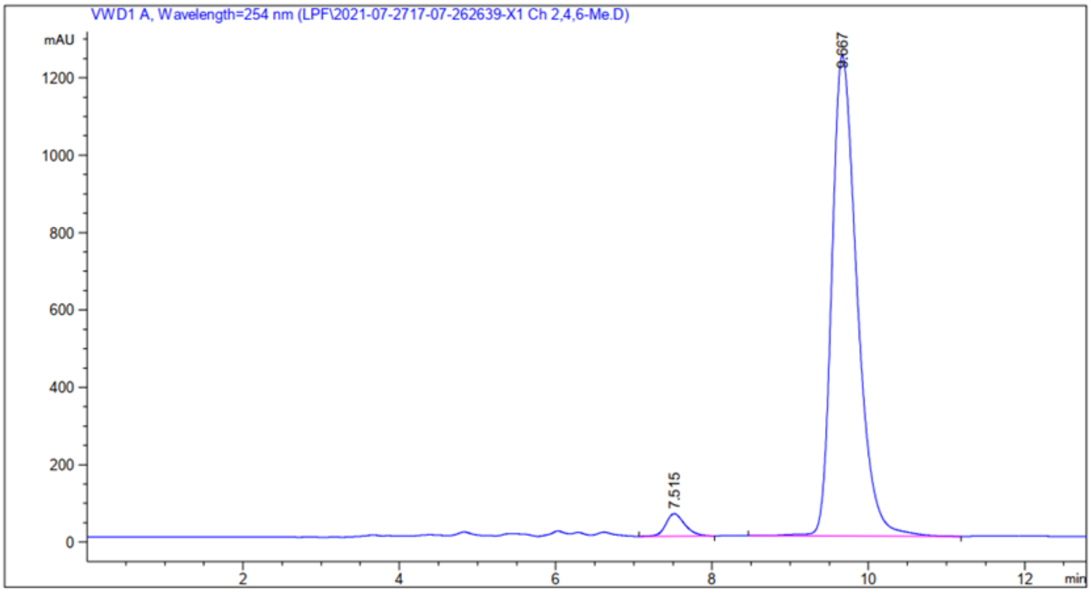

Peak #	RetTime [min]	Type	Width [min]	Area [mAU*s]	Height [mAU]	Area %
1	7.515	BB	0.2566	976.17487	57.92714	3.3925
2	9.667	BB	0.3432	2.77980e4	1241.66382	96.6075

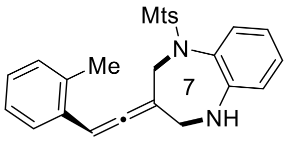

3g

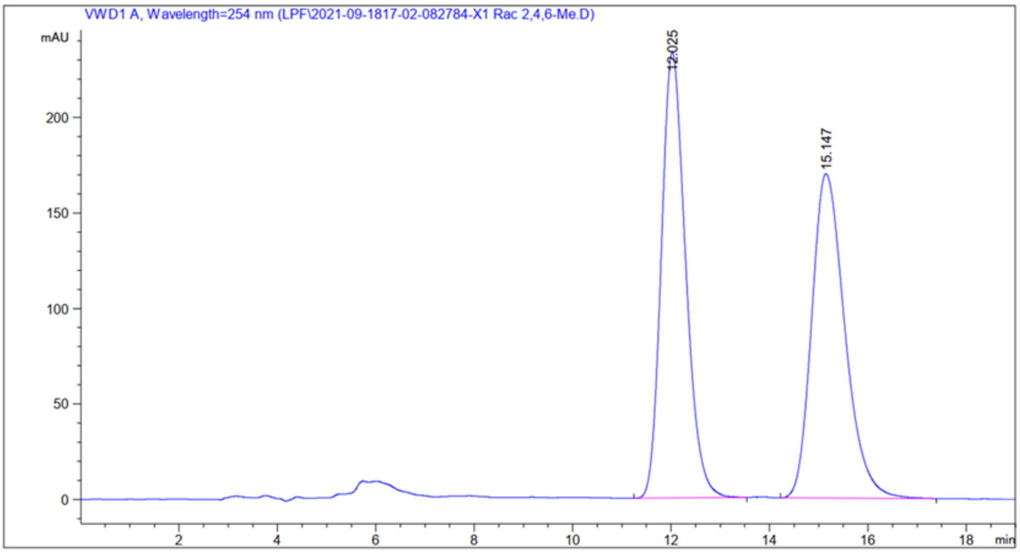

Peak #	RetTime [min]	Type	Width [min]	Area [mAU*s]	Height [mAU]	Area %
1	12.025	BB	0.5270	7981.79102	233.37532	50.1239
2	15.147	BB	0.7270	7942.33008	169.69722	49.8761

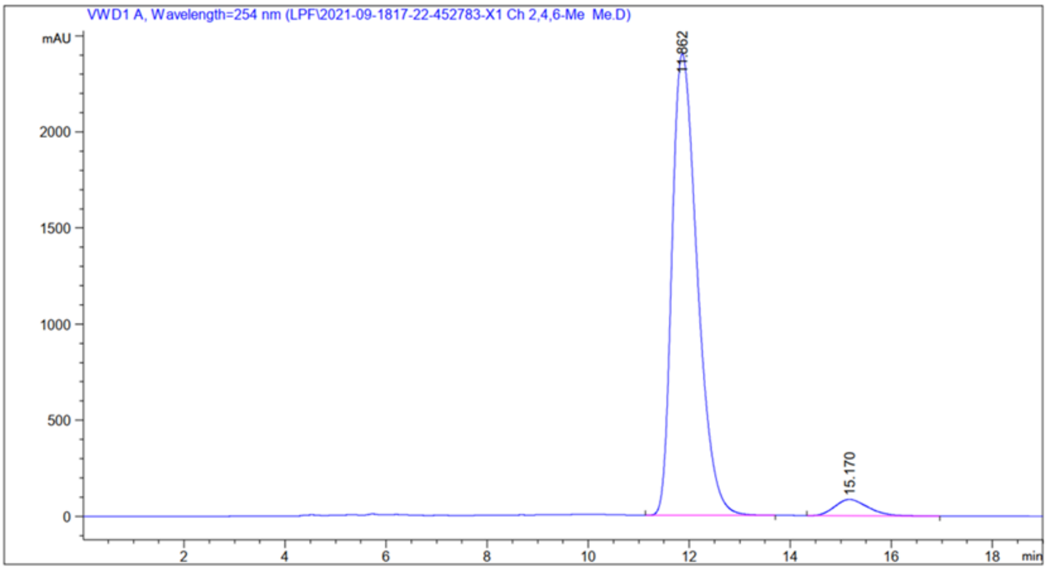

Peak #	RetTime [min]	Type	Width [min]	Area [mAU*s]	Height [mAU]	Area %
1	11.862	BB	0.5407	8.35248e4	2401.84180	95.5438
2	15.170	BB	0.7054	3895.65356	85.21037	4.4562

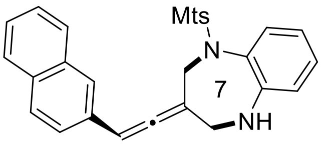

3h

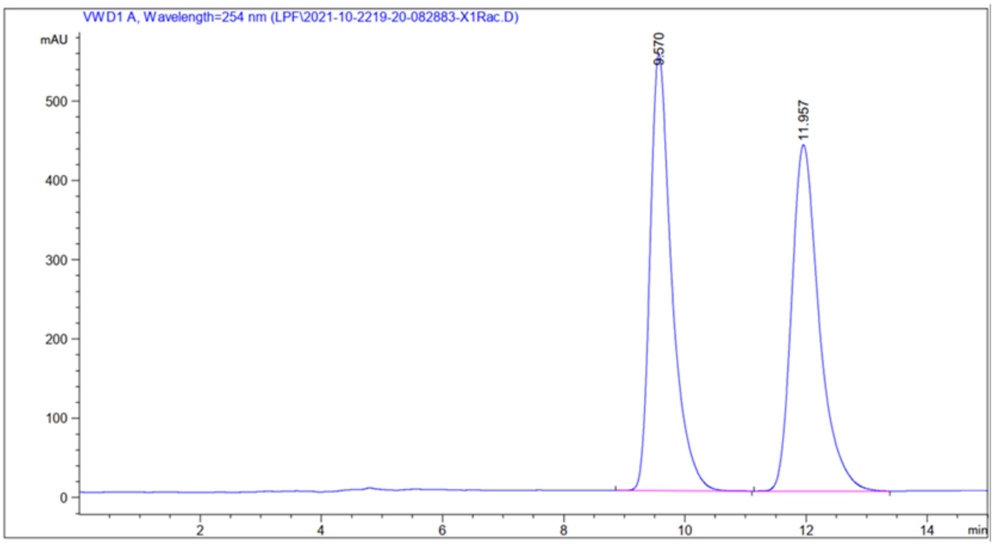

Peak #	RetTime [min]	Type	Width [min]	Area [mAU*s]	Height [mAU]	Area %
1	9.570	BB	0.3715	1.37183e4	550.85828	49.8358
2	11.957	BB	0.4786	1.38087e4	437.00702	50.1642

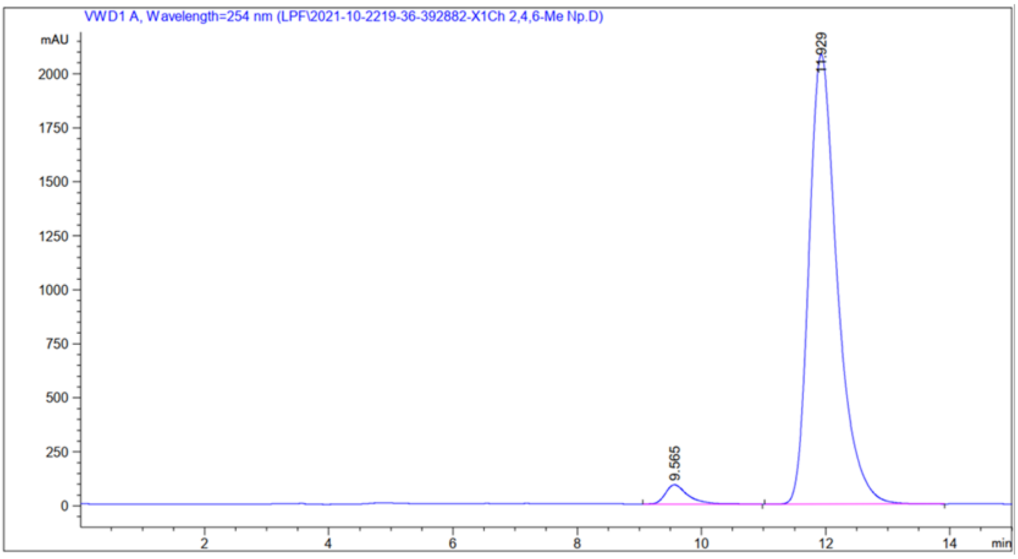

Peak #	RetTime [min]	Type	Width [min]	Area [mAU*s]	Height [mAU]	Area %
1	9.565	BB	0.3720	2235.71338	89.33067	3.2733
2	11.929	BB	0.4823	6.60662e4	2081.21777	96.7267

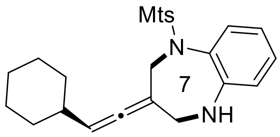

3i

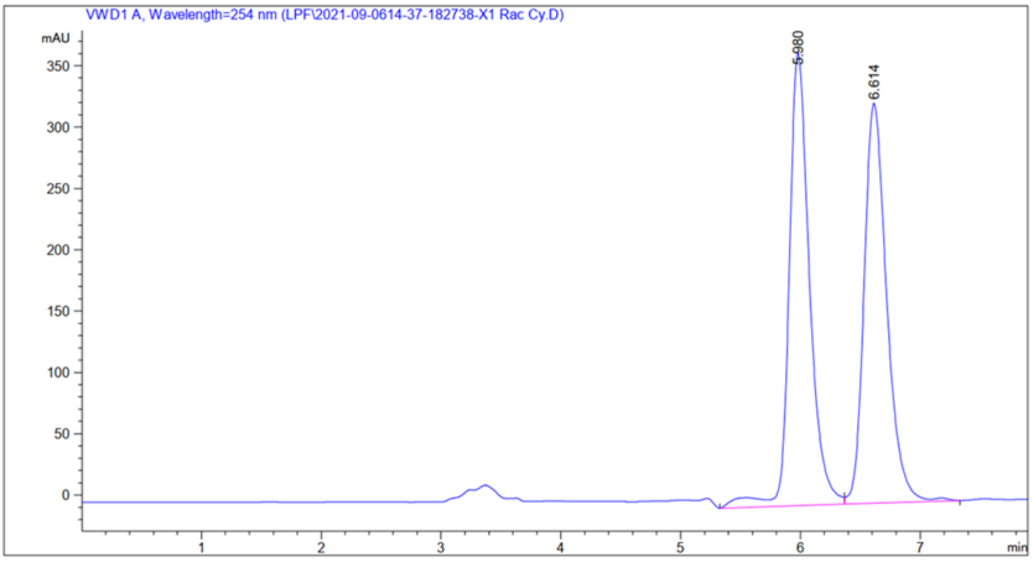

Peak #	RetTime [min]	Type	Width [min]	Area [mAU*s]	Height [mAU]	Area %
1	5.980	VV R	0.1743	4405.71484	369.13498	50.7219
2	6.614	VV R	0.1996	4280.31201	325.97656	49.2781

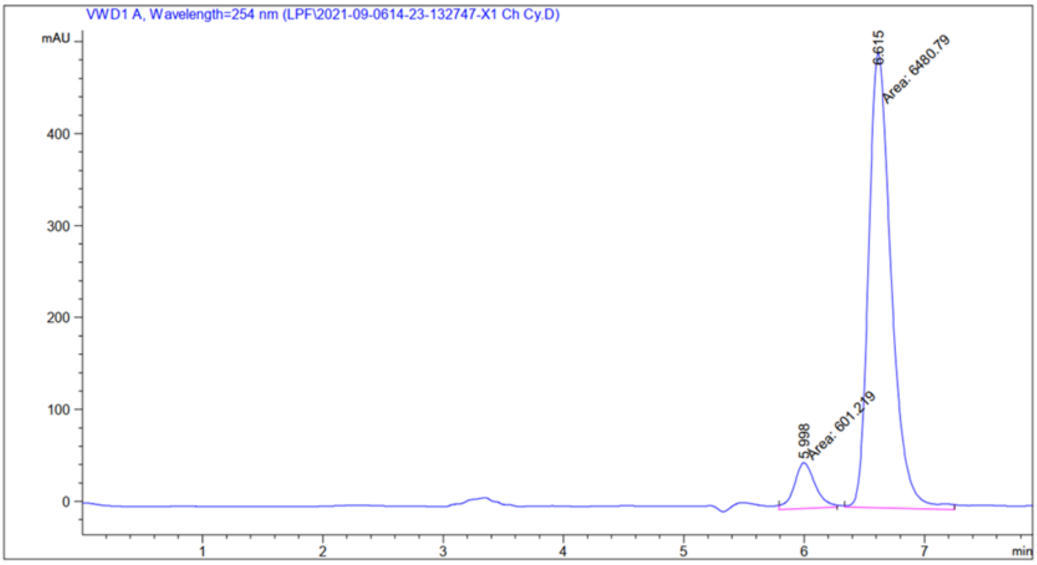

Peak #	RetTime [min]	Type	Width [min]	Area [mAU*s]	Height [mAU]	Area %
1	5.998	MM	0.2021	601.21899	49.58737	8.4894
2	6.615	MM	0.2184	6480.79150	494.63123	91.5106

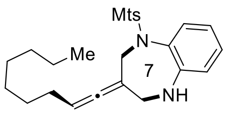

3j

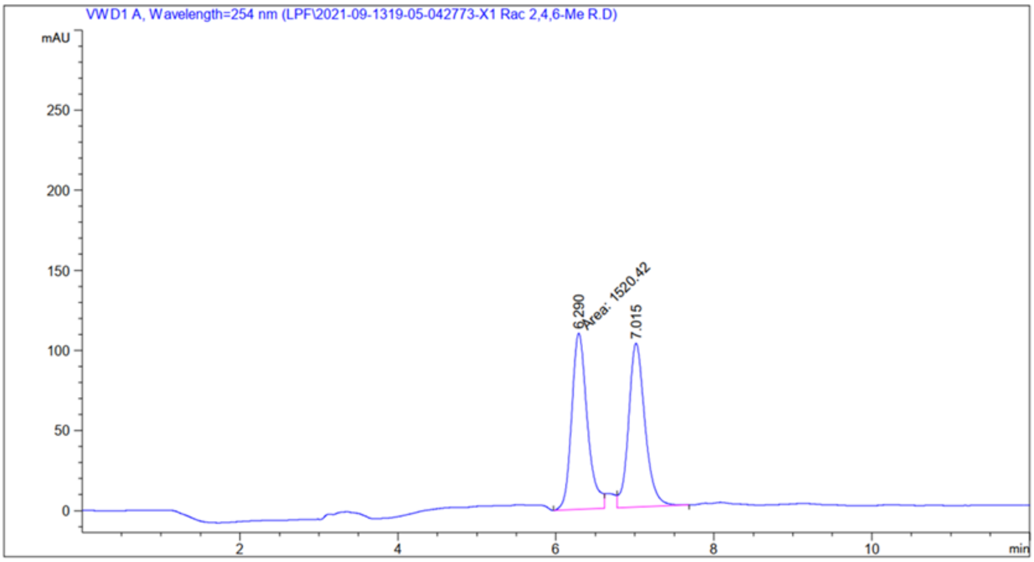

Peak #	RetTime [min]	Type	Width [min]	Area [mAU*s]	Height [mAU]	Area %
1	6.290	MF	0.2301	1520.42053	110.11102	50.6877
2	7.015	VB	0.2186	1479.16589	102.35013	49.3123

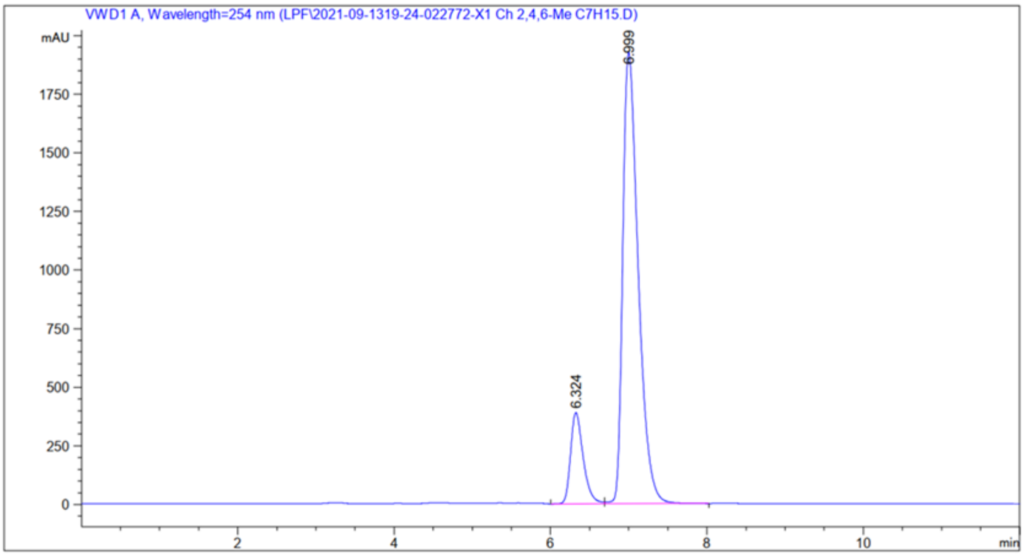

Peak #	RetTime [min]	Type	Width [min]	Area [mAU*s]	Height [mAU]	Area %
1	6.324	BV	0.1718	4387.98486	388.44748	14.2375
2	6.999	VB	0.2102	2.64319e4	1925.04089	85.7625

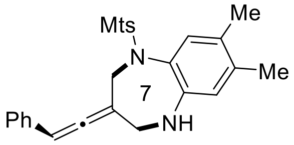

3k

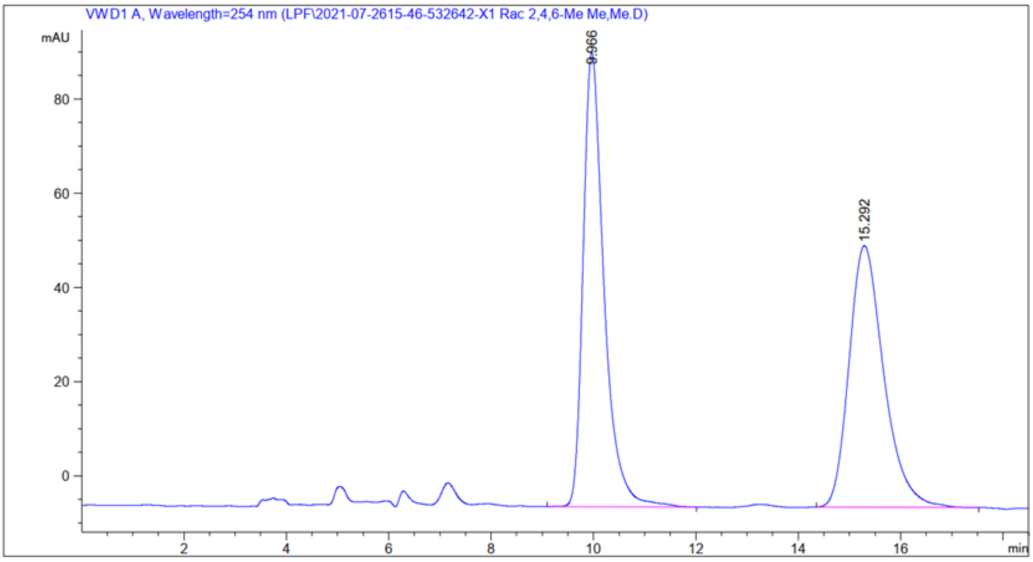

Peak #	RetTime [min]	Type	Width [min]	Area [mAU*s]	Height [mAU]	Area %
1	9.966	BB	0.4269	2734.41064	96.41417	51.0732
2	15.292	BB	0.7152	2619.49463	55.54849	48.9268

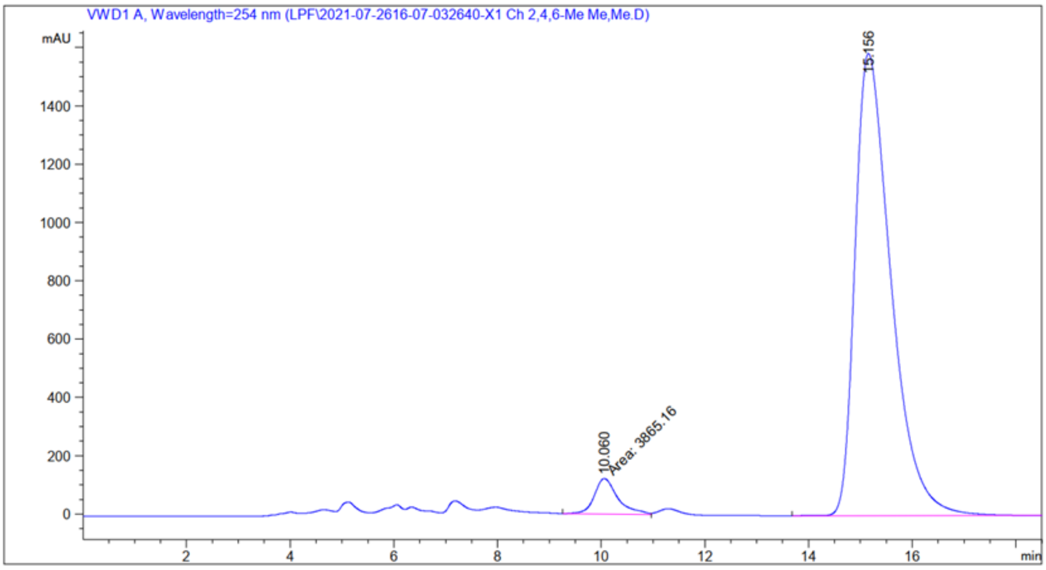

Peak #	RetTime [min]	Type	Width [min]	Area [mAU*s]	Height [mAU]	Area %
1	10.060	MF	0.5281	3865.15723	121.97577	4.8182
2	15.156	BB	0.7412	7.63555e4	1584.41638	95.1818

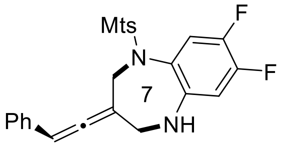

3l

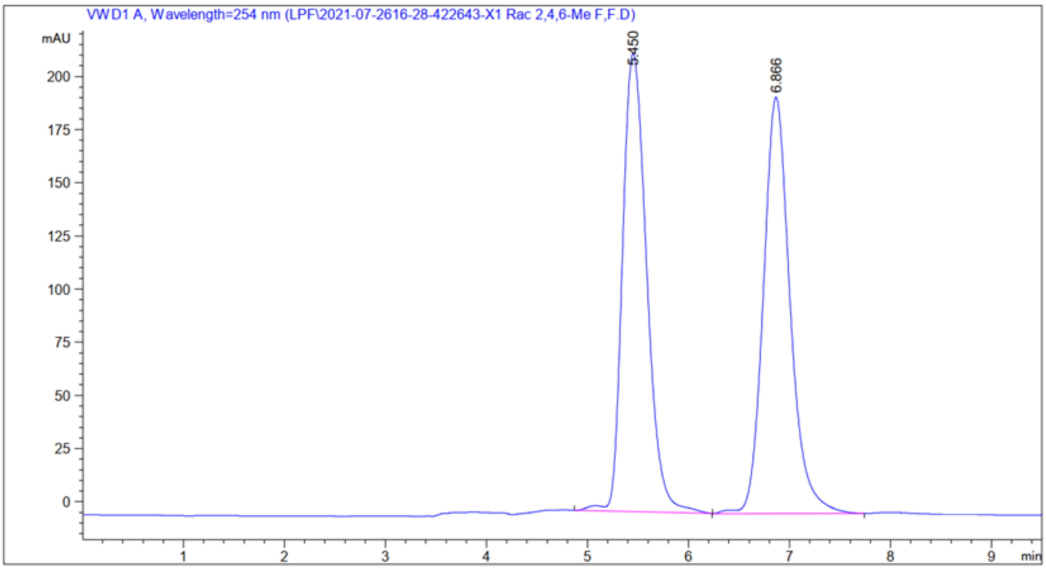

Peak #	RetTime [min]	Type	Width [min]	Area [mAU*s]	Height [mAU]	Area %
1	5.450	VB R	0.2616	3583.56494	215.46179	49.8099
2	6.866	VB R	0.2791	3610.91577	195.83600	50.1901

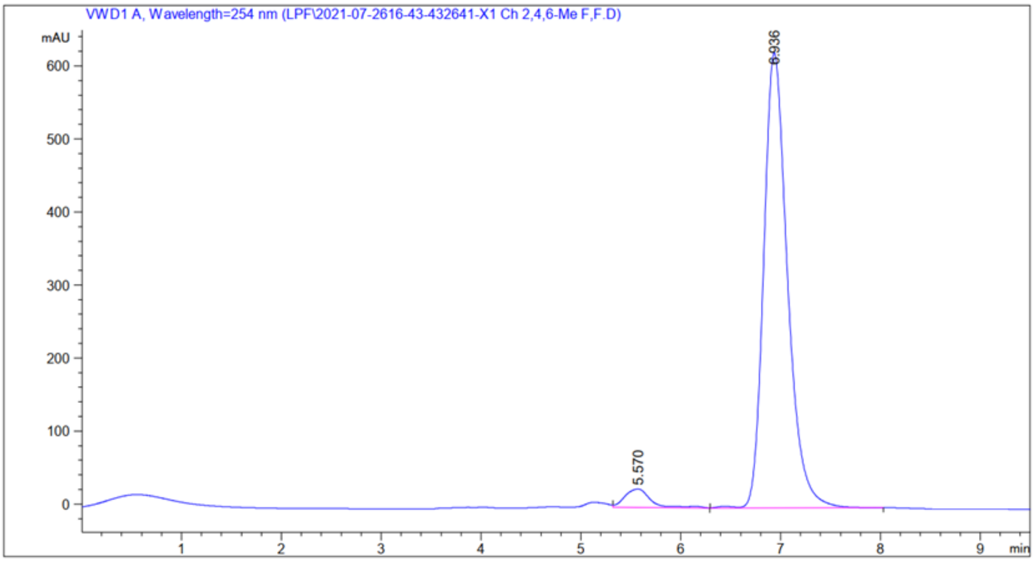

Peak #	RetTime [min]	Type	Width [min]	Area [mAU*s]	Height [mAU]	Area %
1	5.570	VV R	0.2827	457.58356	25.33353	4.2468
2	6.936	VB R	0.2516	1.03173e4	623.37823	95.7532

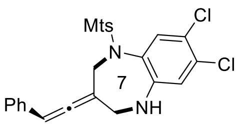

3m

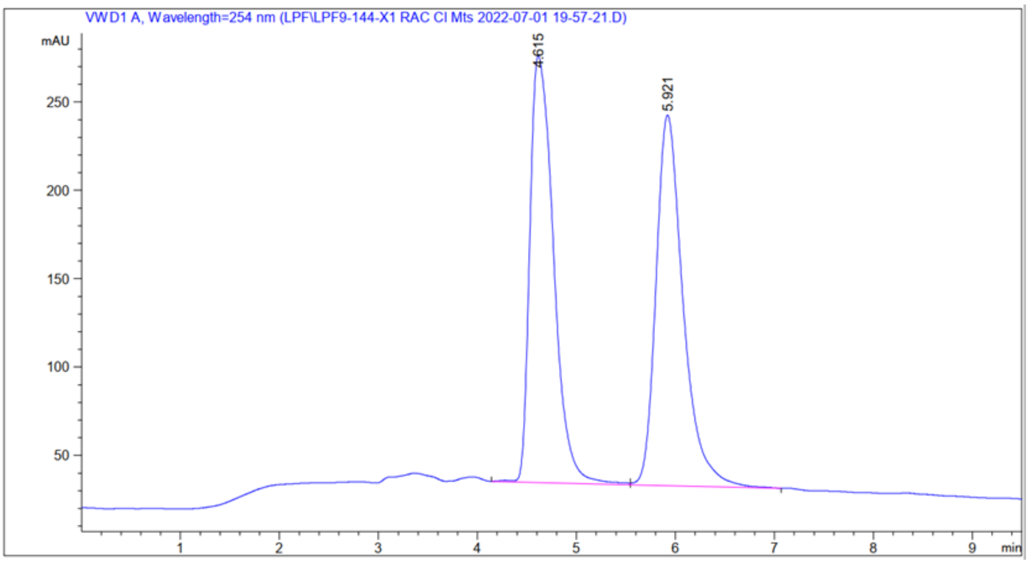

Peak #	RetTime [min]	Type	Width [min]	Area [mAU*s]	Height [mAU]	Area %
1	4.615	VV R	0.2657	4009.13647	241.49821	50.0804
2	5.921	VB	0.2885	3996.27075	209.58128	49.9196

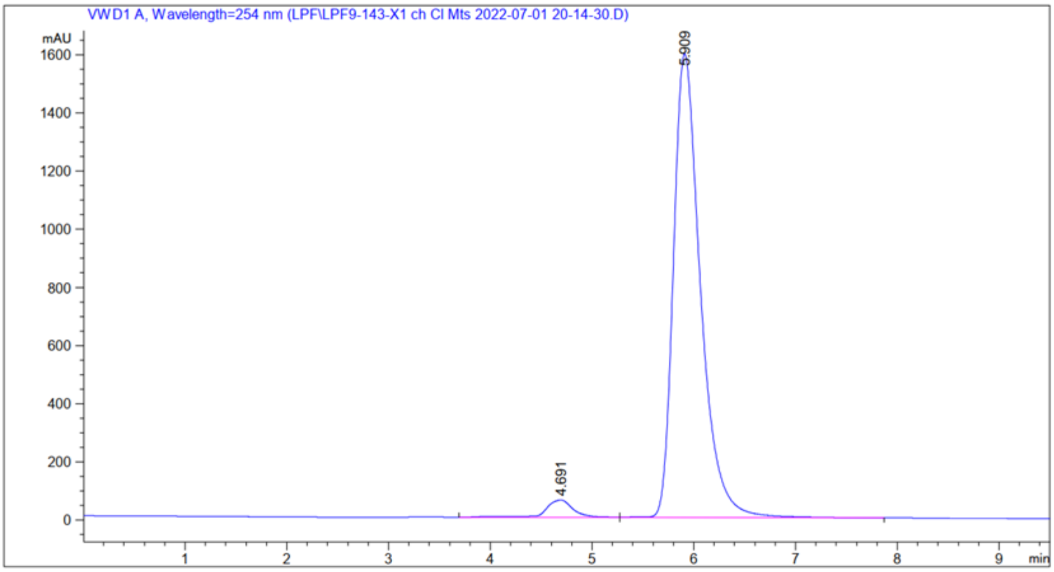

Peak #	RetTime [min]	Type	Width [min]	Area [mAU*s]	Height [mAU]	Area %
1	4.691	VB R	0.2684	1121.33350	59.30951	3.6698
2	5.909	VB R	0.2801	2.94340e4	1596.75903	96.3302

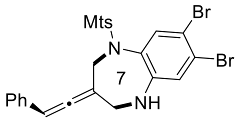

3n

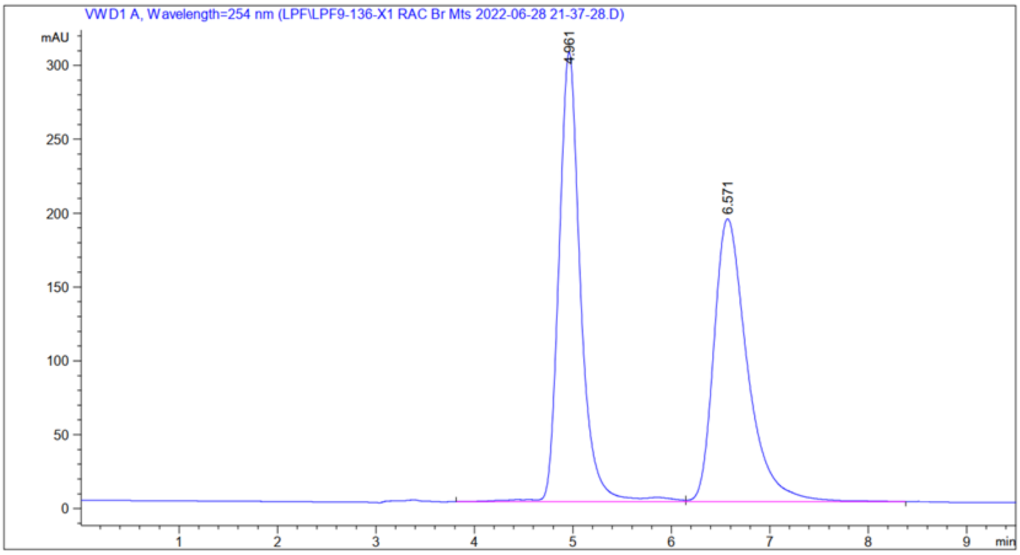

Peak #	RetTime [min]	Type	Width [min]	Area [mAU*s]	Height [mAU]	Area %
1	4.961	VV R	0.2283	4658.89990	304.34665	50.7893
2	6.571	VB	0.3558	4514.10254	191.63524	49.2107

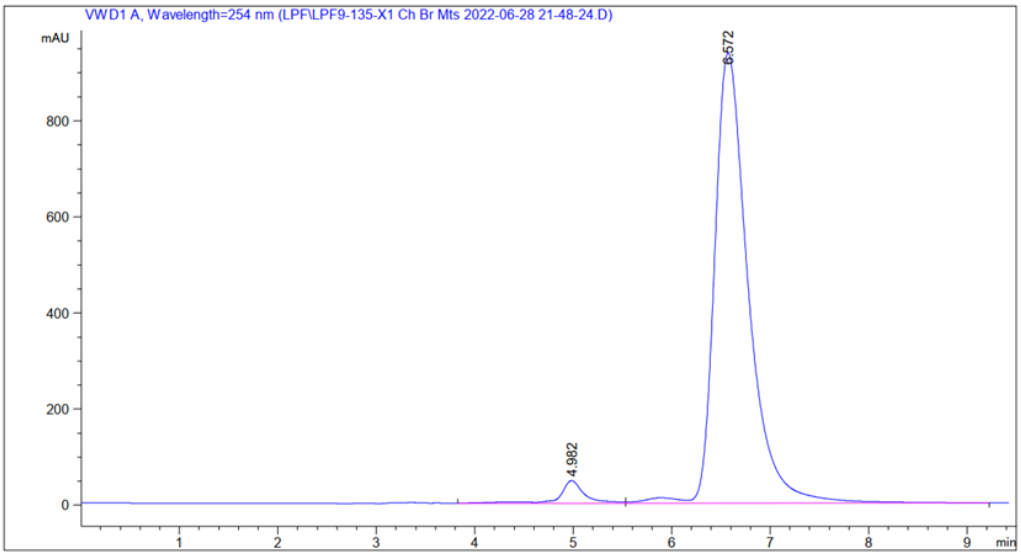

Peak #	RetTime [min]	Type	Width [min]	Area [mAU*s]	Height [mAU]	Area %
1	4.982	VV R	0.2261	794.97290	47.00899	3.4427
2	6.572	VB R	0.3542	2.22966e4	937.35022	96.5573

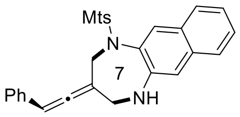

3o

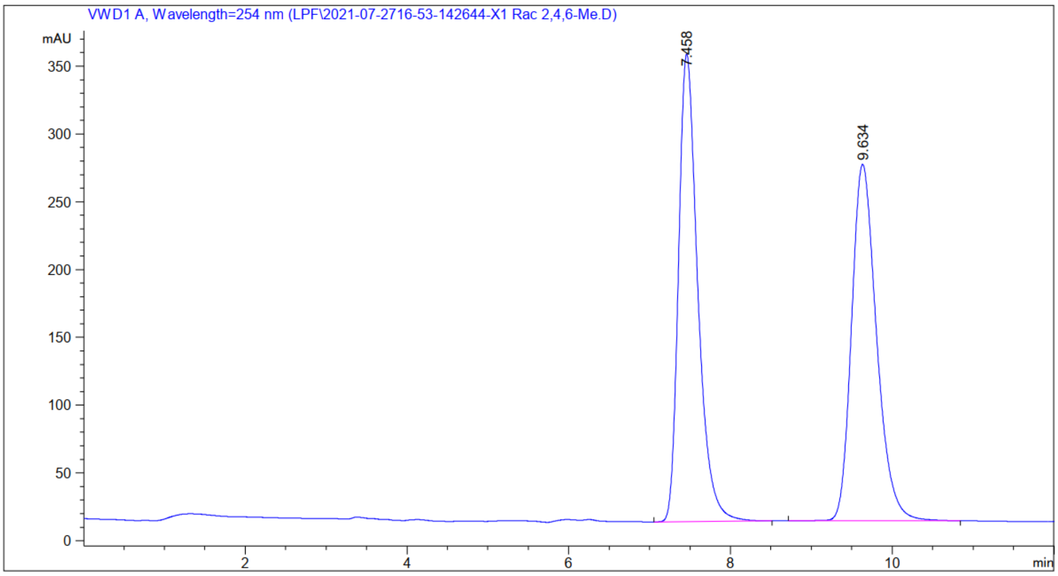

Peak #	RetTime [min]	Type	Width [min]	Area [mAU*s]	Height [mAU]	Area %
1	7.458	BB	0.2505	5691.47607	345.00690	50.1586
2	9.634	BB	0.3289	5655.47656	262.94412	49.8414

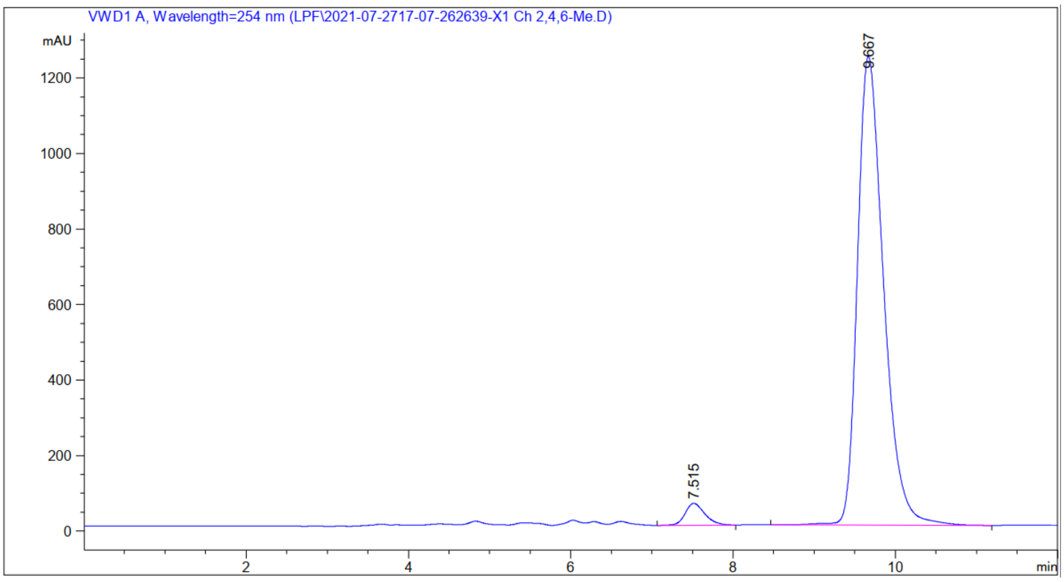

Peak #	RetTime [min]	Type	Width [min]	Area [mAU*s]	Height [mAU]	Area %
1	7.515	BB	0.2566	976.17487	57.92714	3.3925
2	9.667	BB	0.3432	2.77980e4	1241.66382	96.6075

HPLC spectra of 4

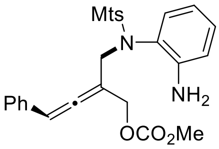

4a

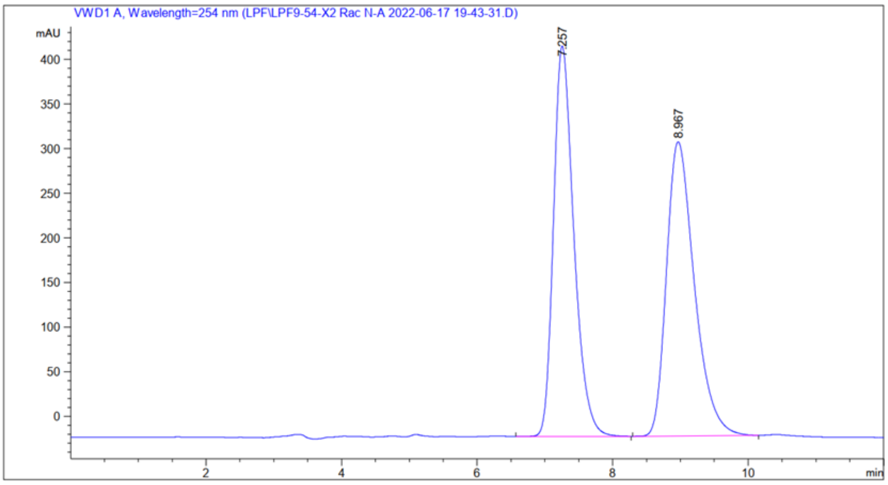

Peak #	RetTime [min]	Type	Width [min]	Area [mAU*s]	Height [mAU]	Area %
1	7.257	BB	0.3176	9124.85547	437.07202	49.1833
2	8.967	BB	0.4369	9427.89648	329.37833	50.8167

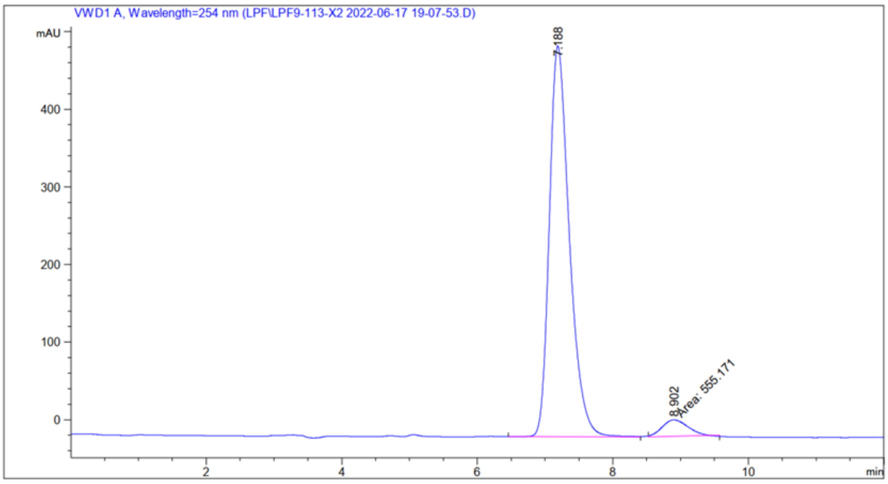

Peak #	RetTime [min]	Type	Width [min]	Area [mAU*s]	Height [mAU]	Area %
1	7.188	BV	0.3125	1.03275e4	503.14160	94.8986
2	8.902	MM	0.4392	555.17096	21.06831	5.1014

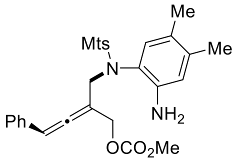

4b

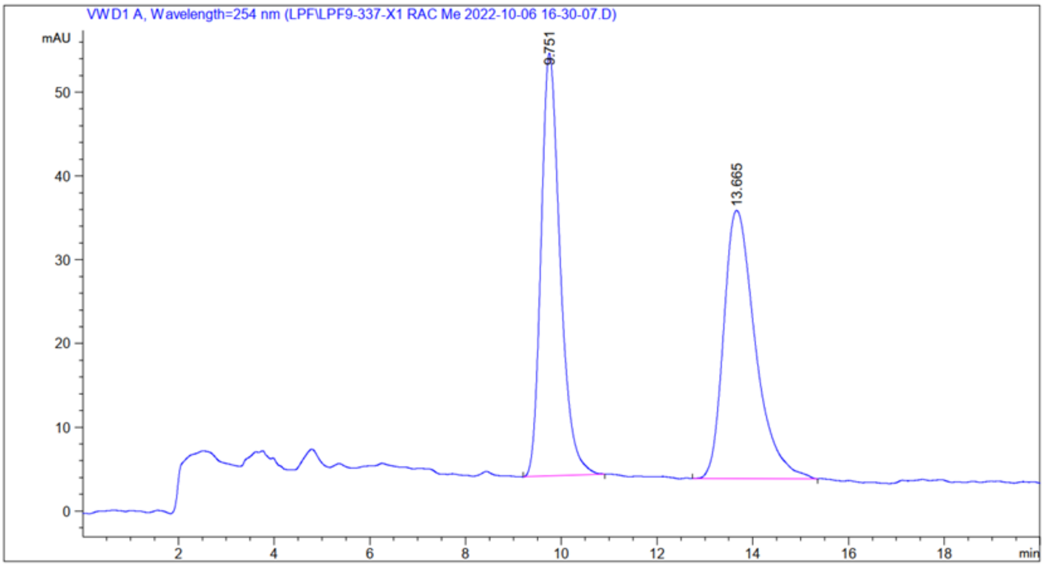

Peak #	RetTime [min]	Type	Width [min]	Area [mAU*s]	Height [mAU]	Area %
1	9.751	BB	0.4245	1399.94214	50.48568	48.7418
2	13.665	BB	0.7023	1472.21765	32.02613	51.2582

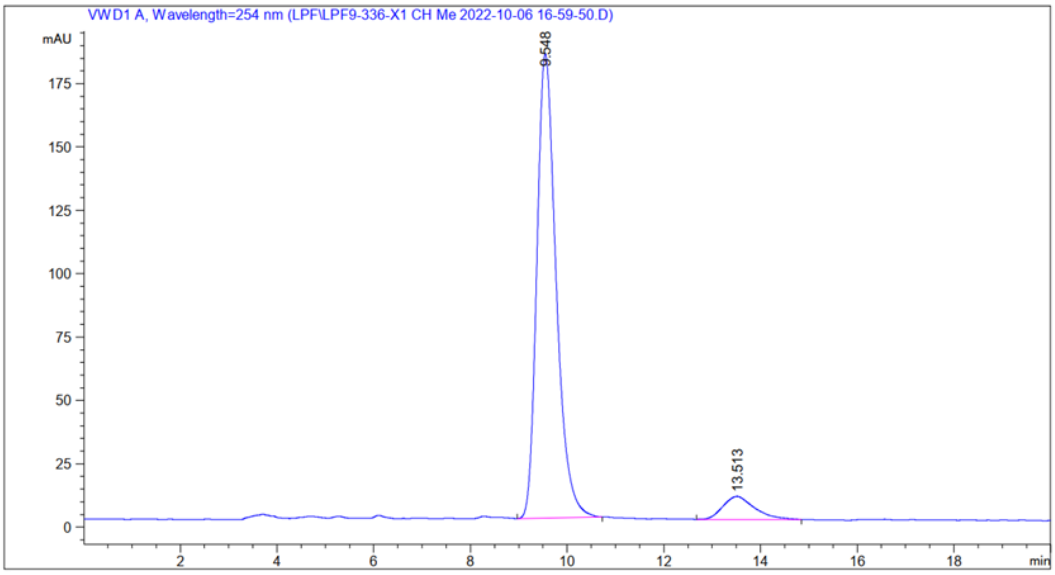

Peak #	RetTime [min]	Type	Width [min]	Area [mAU*s]	Height [mAU]	Area %
1	9.548	BB	0.4219	5064.64404	183.00911	92.4774
2	13.513	BB	0.6643	411.98242	9.09832	7.5226

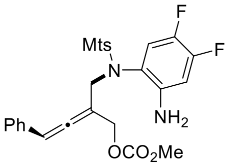

4c

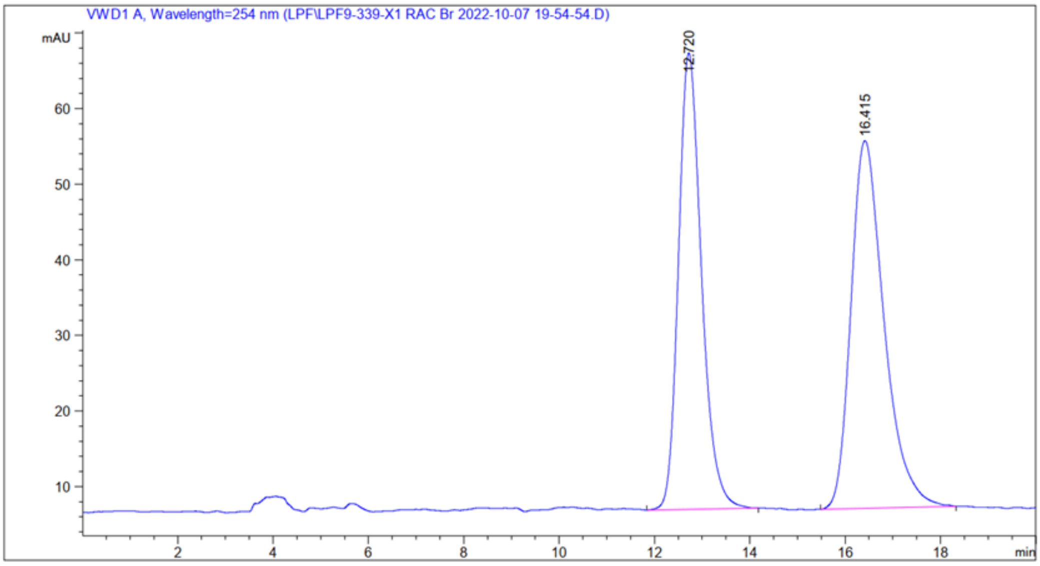

Peak #	RetTime [min]	Type	Width [min]	Area [mAU*s]	Height [mAU]	Area %
1	12.720	BB	0.5170	2027.60803	60.34974	46.3352
2	16.415	BB	0.7404	2348.34448	48.62693	53.6648

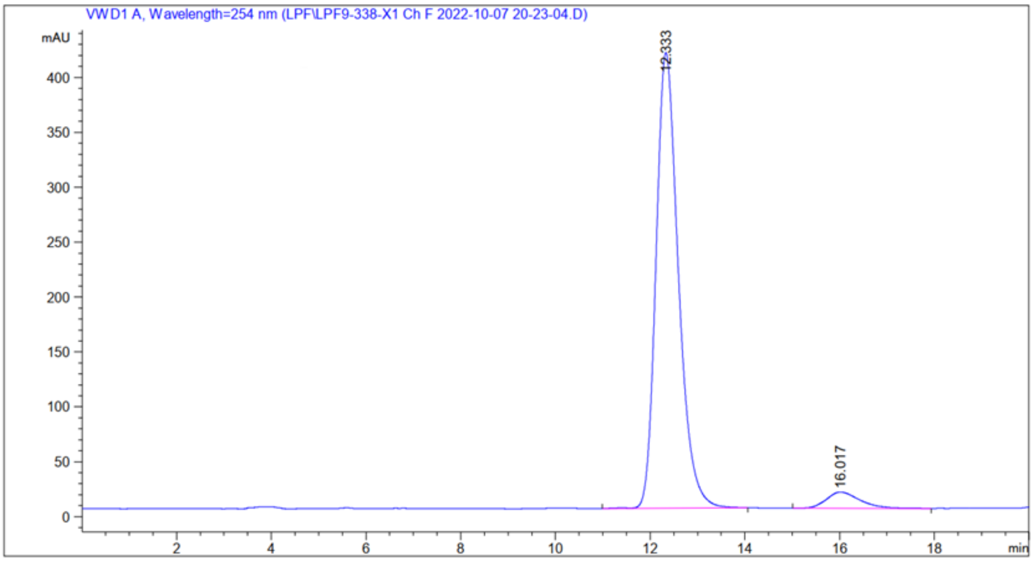

Peak #	RetTime [min]	Type	Width [min]	Area [mAU*s]	Height [mAU]	Area %
1	12.333	BB	0.5025	1.36032e4	414.88126	94.9208
2	16.017	BB	0.7274	727.90375	14.75849	5.0792

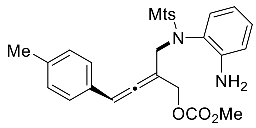

4d

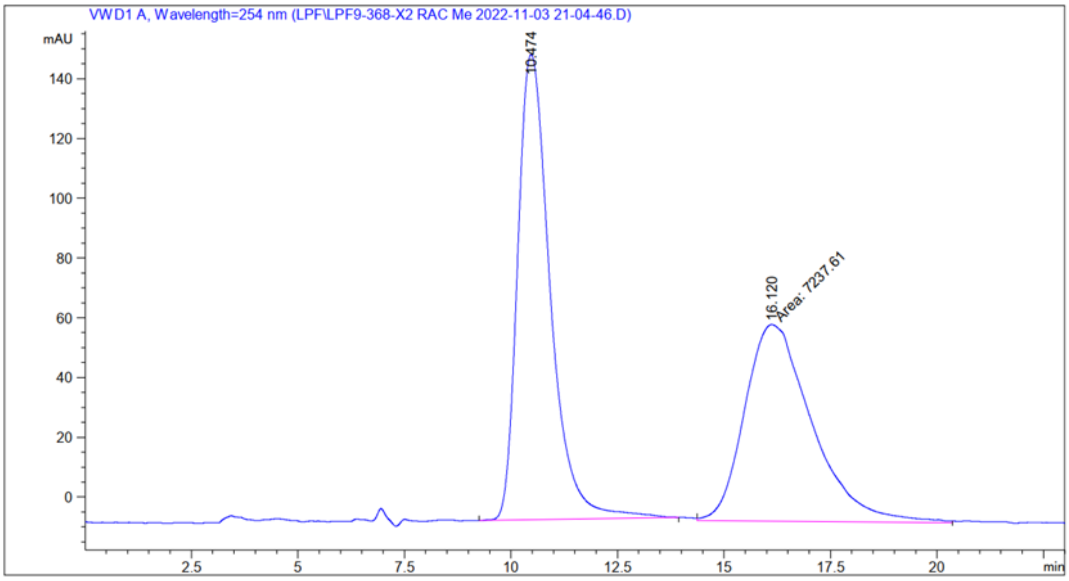

Peak #	RetTime [min]	Type	Width [min]	Area [mAU*s]	Height [mAU]	Area %
1	10.474	BB	0.8311	8291.98242	155.76305	53.3947
2	16.120	MM	1.8315	7237.61133	65.86270	46.6053

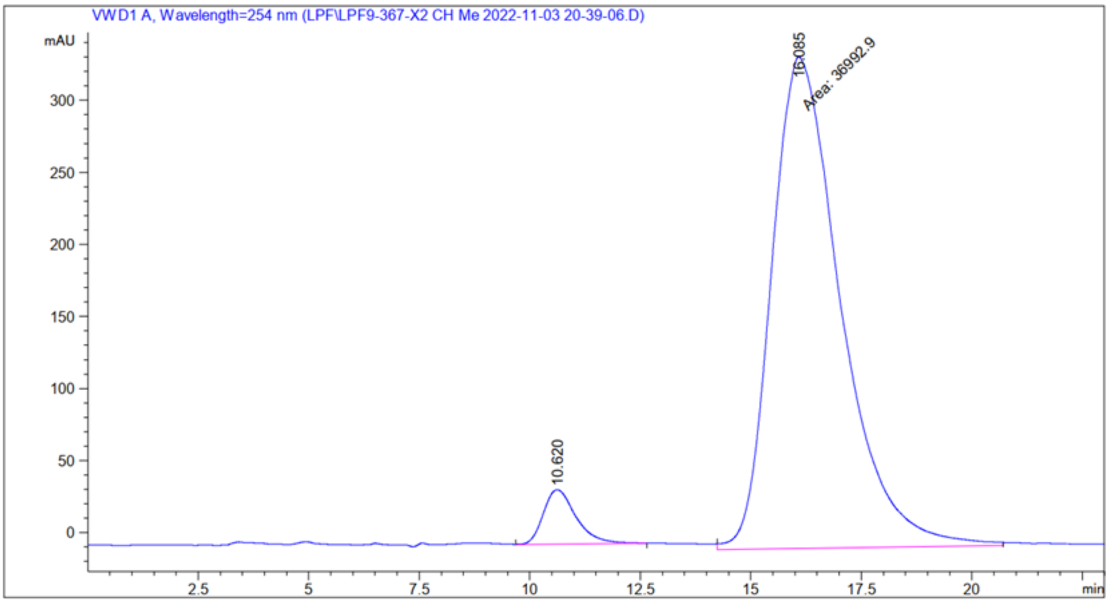

Peak #	RetTime [min]	Type	Width [min]	Area [mAU*s]	Height [mAU]	Area %
1	10.620	BB	0.7868	1925.47168	37.57972	4.9475
2	16.085	MM	1.8070	3.69929e4	341.20490	95.0525

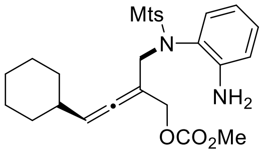

4e

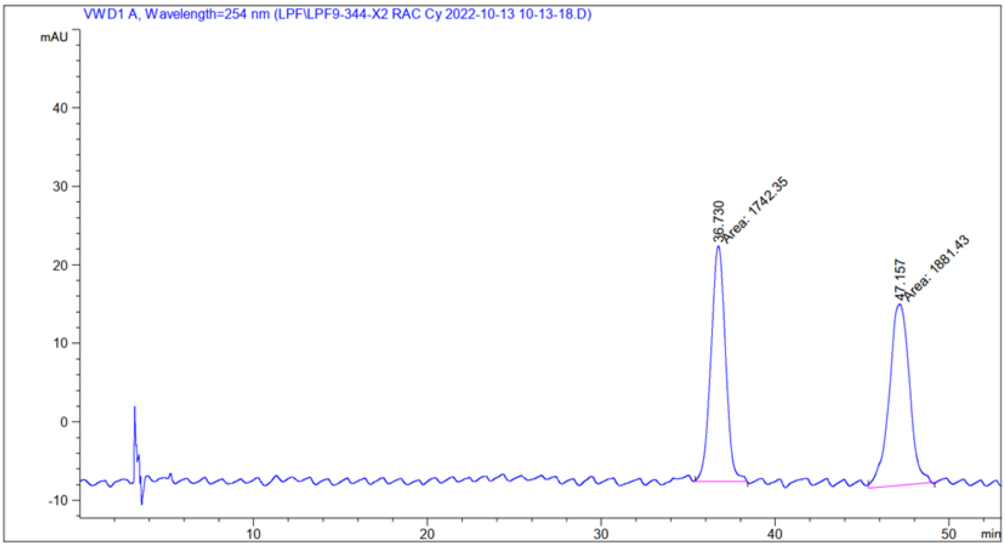

Peak #	RetTime [min]	Type	Width [min]	Area [mAU*s]	Height [mAU]	Area %
1	36.730	MM	0.9682	1742.34827	29.99313	48.0810
2	47.157	MM	1.3575	1881.42603	23.09932	51.9190

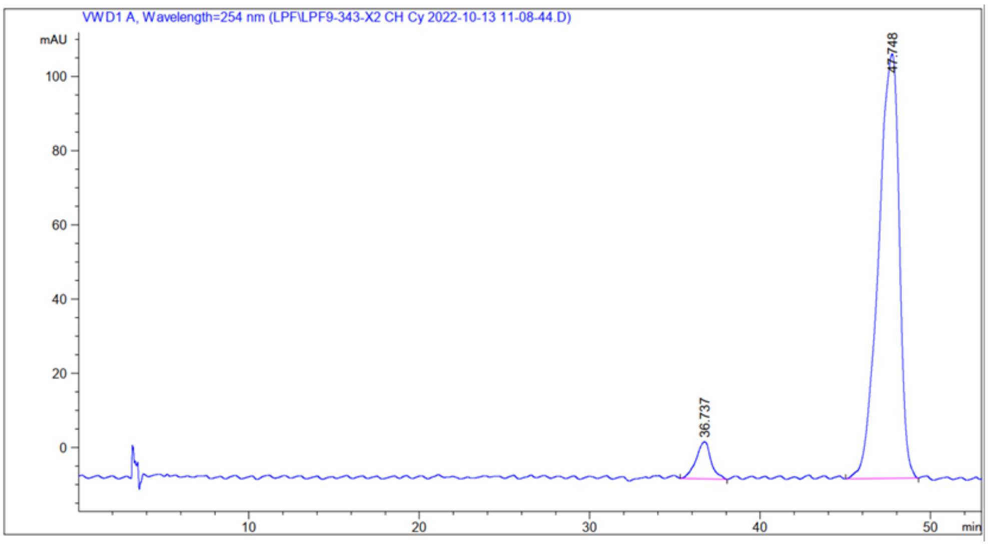

Peak #	RetTime [min]	Type	Width [min]	Area [mAU*s]	Height [mAU]	Area %
1	36.737	BB	0.8925	606.13983	9.93844	5.8849
2	47.748	BB	1.2213	9693.84863	114.32286	94.1151

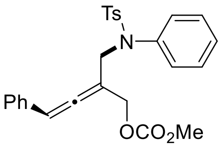

4f

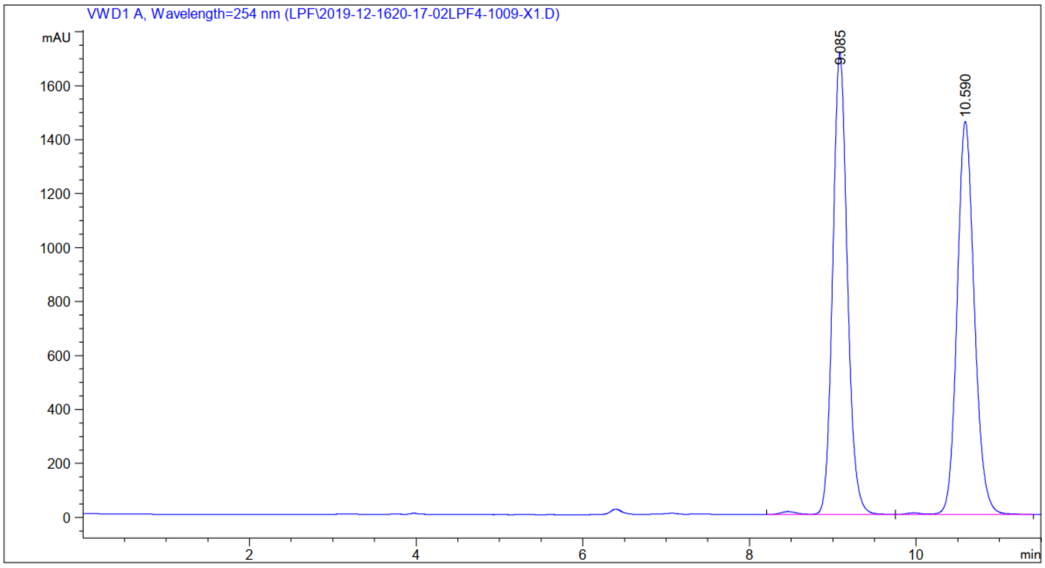

Peak #	RetTime [min]	Type	Width [min]	Area [mAU*s]	Height [mAU]	Area %
1	9.085	VB R	0.1872	2.10058e4	1711.10156	49.9772
2	10.590	VB R	0.2208	2.10250e4	1456.85413	50.0228

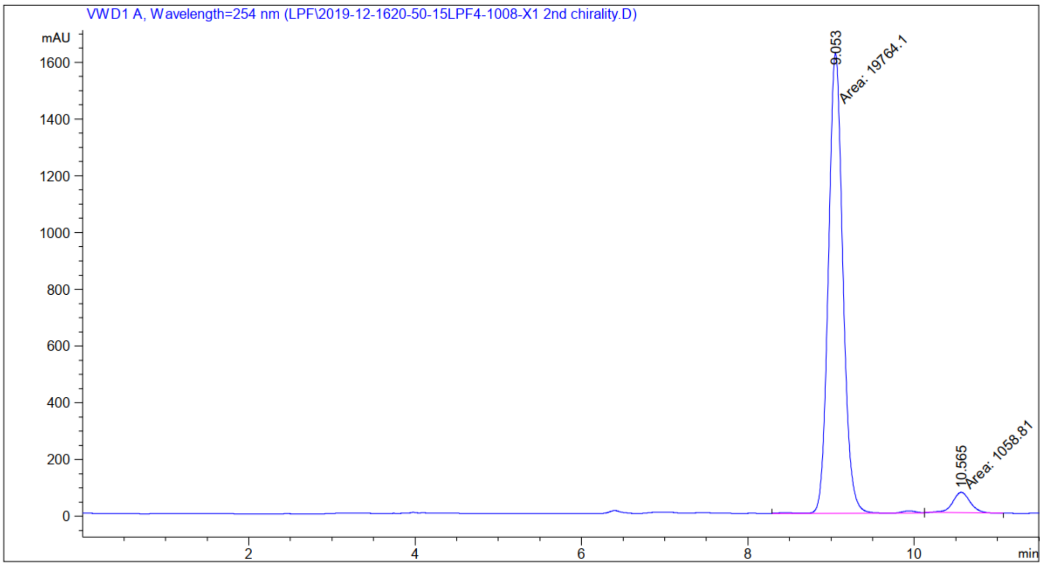

Peak #	RetTime [min]	Type	Width [min]	Area [mAU*s]	Height [mAU]	Area %
1	9.053	MM	0.2030	1.97641e4	1622.75562	94.9152
2	10.565	MM	0.2448	1058.81433	72.07870	5.0848

HPLC spectra of 9

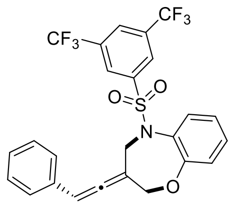

9a

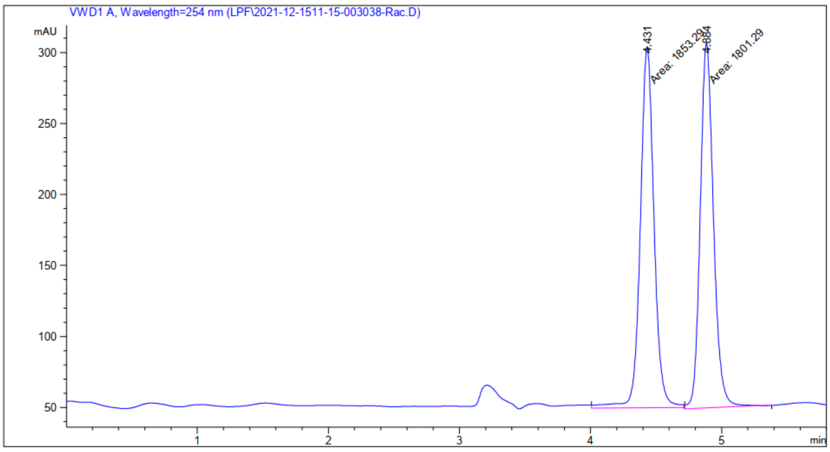

Peak #	RetTime [min]	Type	Width [min]	Area [mAU*s]	Height [mAU]	Area %
1	4.431	FM	0.1215	1853.29468	254.24162	50.7114
2	4.884	MM	0.1169	1801.29443	256.89578	49.2886

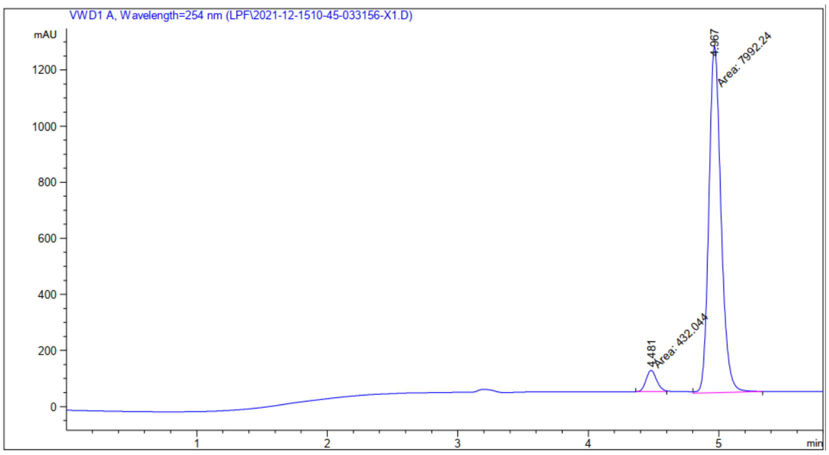

Peak #	RetTime [min]	Type	Width [min]	Area [mAU*s]	Height [mAU]	Area %
1	4.481	MM	0.0946	432.04440	76.11656	5.1286
2	4.967	MM	0.1078	7992.24316	1235.10425	94.8714

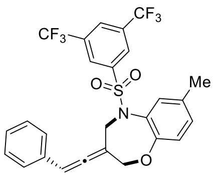

9b

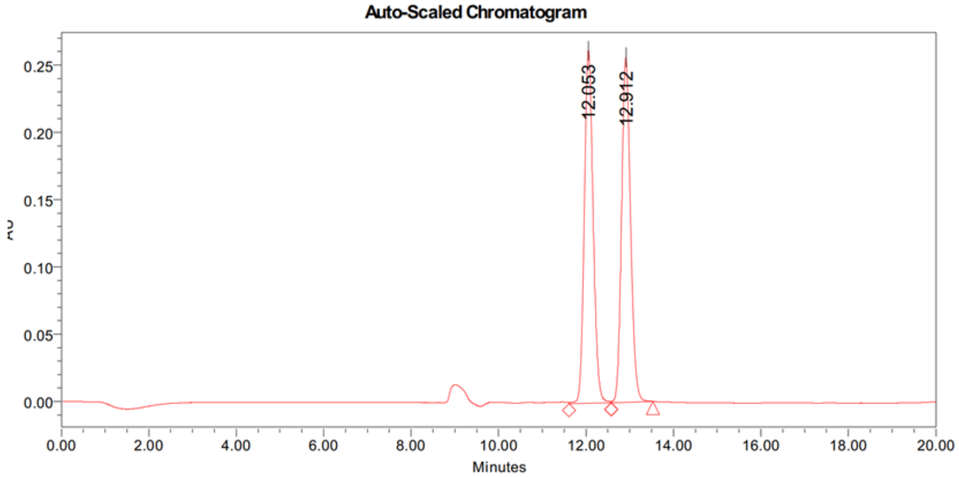

Peak Results

	Name	RT	Area	Height	% Area	Peak Width (sec)
1		12.053	3679966	262041	50.18	30.00
2		12.912	3653694	256223	49.82	30.00

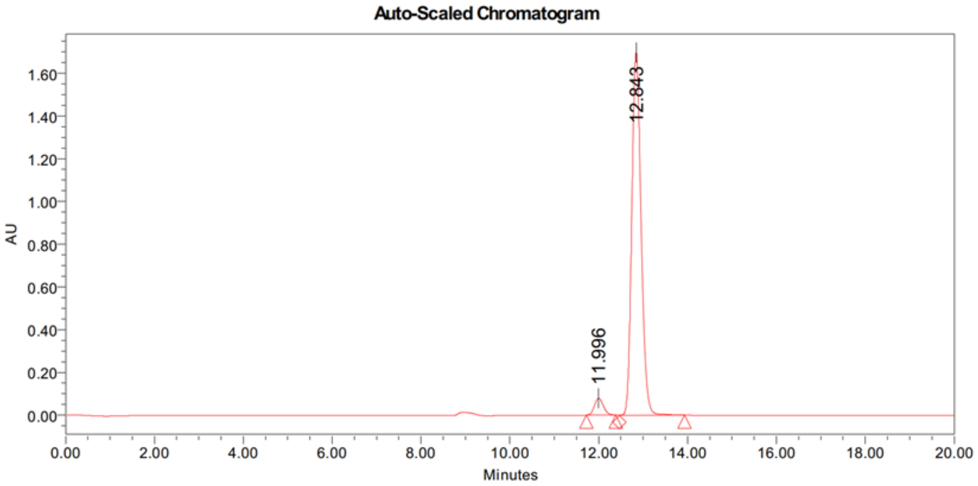

Peak Results

	Name	RT	Area	Height	% Area	Peak Width (sec)
1		11.996	1105519	78805	4.26	30.00
2		12.843	24816484	1699008	95.74	30.00

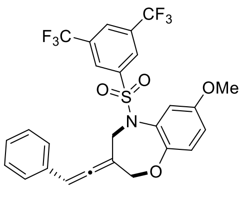

9c

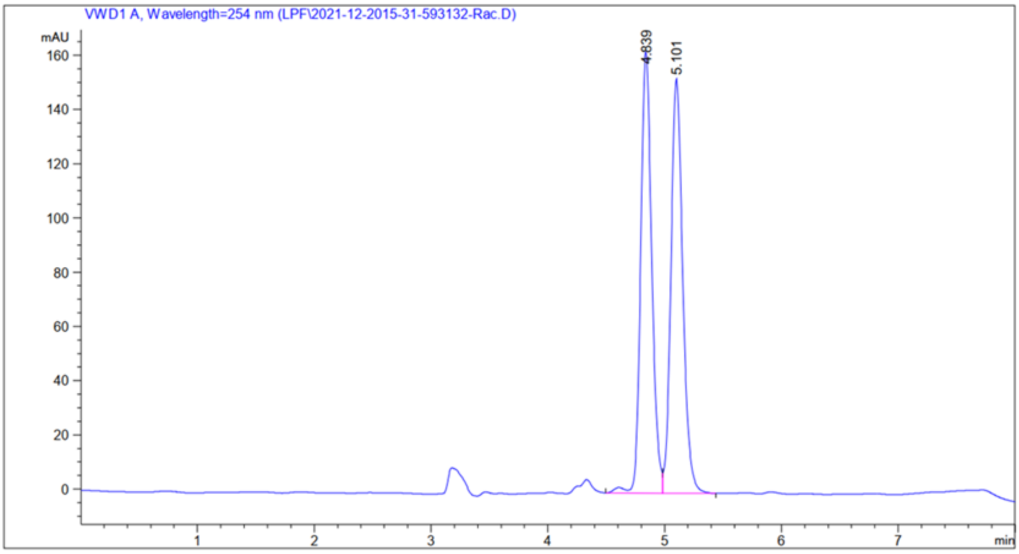

Peak #	RetTime [min]	Type	Width [min]	Area [mAU*s]	Height [mAU]	Area %
1	4.839	VV R	0.1001	1075.15686	162.71704	49.9954
2	5.101	VB	0.1081	1075.35315	152.89691	50.0046

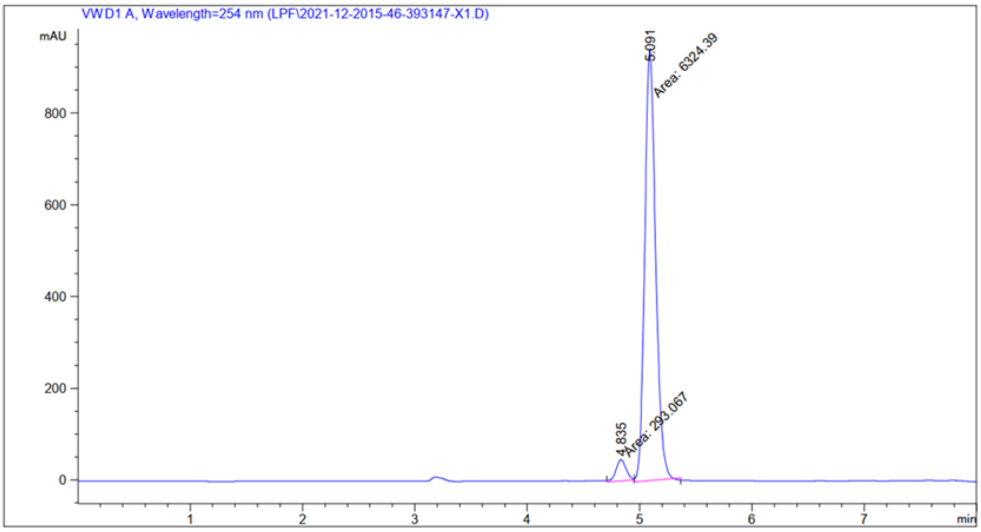

Peak #	RetTime [min]	Type	Width [min]	Area [mAU*s]	Height [mAU]	Area %
1	4.835	MM	0.1050	293.06735	46.51565	4.4287
2	5.091	MM	0.1123	6324.39355	938.66296	95.5713

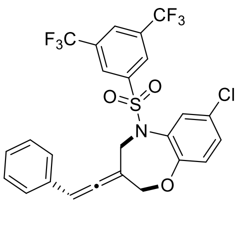

9d

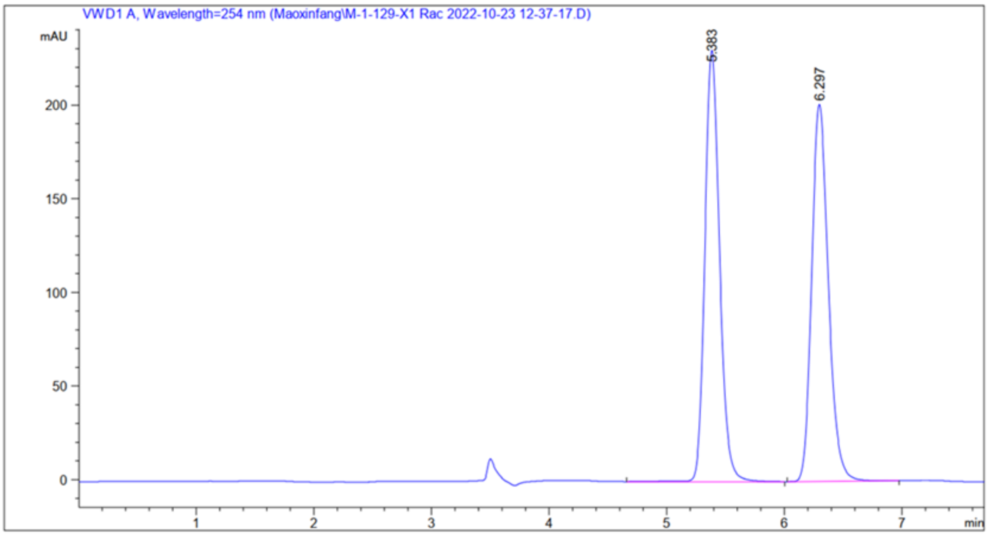

Peak #	RetTime [min]	Type	Width [min]	Area [mAU*s]	Height [mAU]	Area %
1	5.383	BB	0.1347	2005.86682	230.18884	50.3140
2	6.297	BB	0.1519	1980.83411	201.19957	49.6860

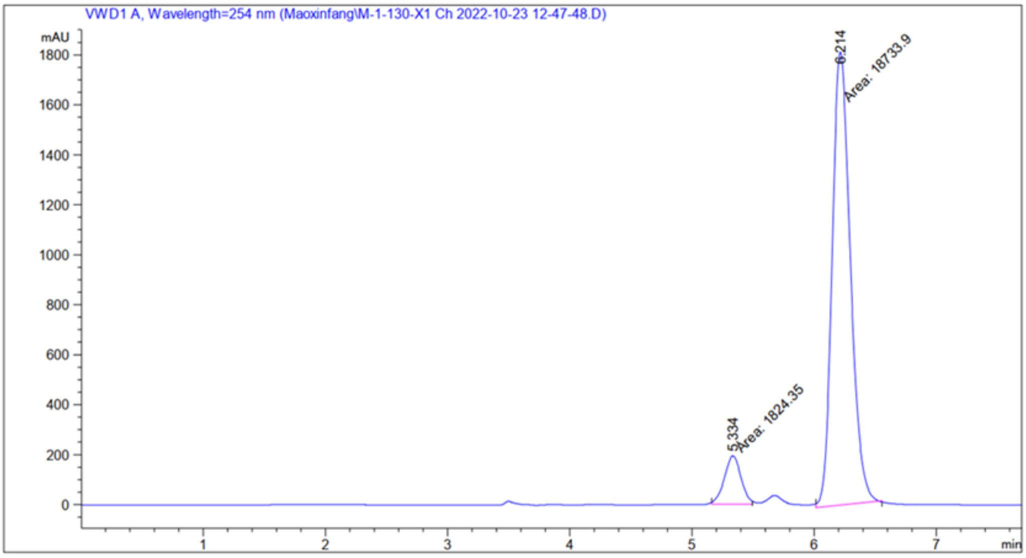

Peak #	RetTime [min]	Type	Width [min]	Area [mAU*s]	Height [mAU]	Area %
1	5.334	MM	0.1575	1824.34619	193.05765	8.8740
2	6.214	MM	0.1723	1.87339e4	1812.40552	91.1260

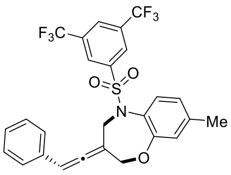

9e

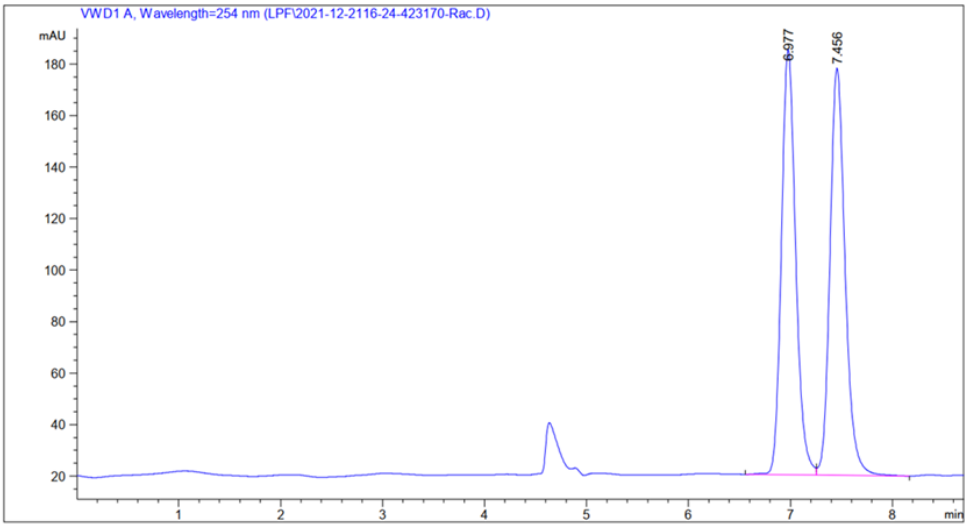

Peak #	RetTime [min]	Type	Width [min]	Area [mAU*s]	Height [mAU]	Area %
1	6.977	BV	0.1496	1607.15771	165.08972	49.6581
2	7.456	VB	0.1583	1629.28650	158.07056	50.3419

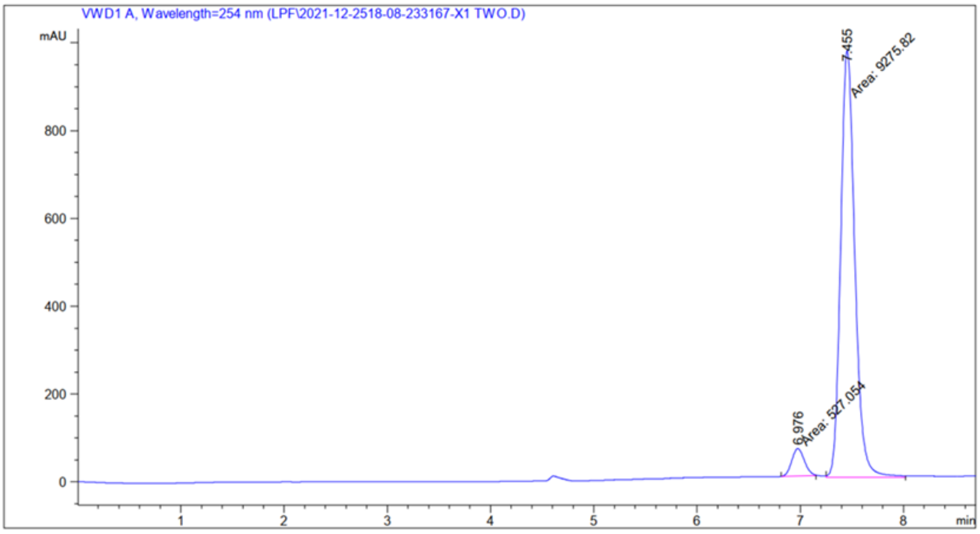

Peak #	RetTime [min]	Type	Width [min]	Area [mAU*s]	Height [mAU]	Area %
1	6.976	MM	0.1409	527.05396	62.35249	5.3765
2	7.455	MM	0.1588	9275.82031	973.22699	94.6235

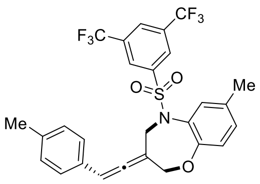

9f

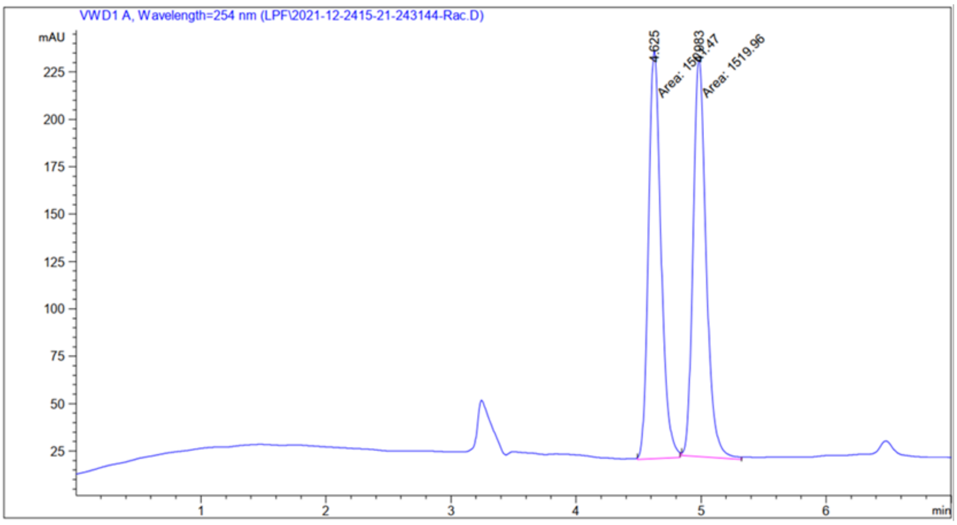

Peak #	RetTime [min]	Type	Width [min]	Area [mAU*s]	Height [mAU]	Area %
1	4.625	MM	0.1163	1501.47388	215.16077	49.6940
2	4.983	MM	0.1211	1519.96387	209.26945	50.3060

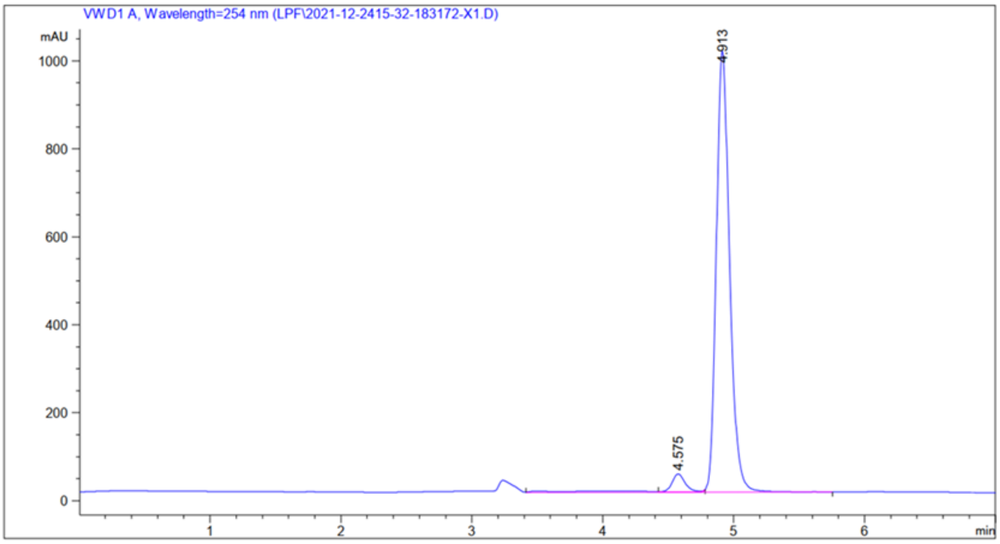

Peak #	RetTime [min]	Type	Width [min]	Area [mAU*s]	Height [mAU]	Area %
1	4.575	VV E	0.1076	289.37299	40.89060	3.8588
2	4.913	VV R	0.1075	7209.61621	1002.60059	96.1412

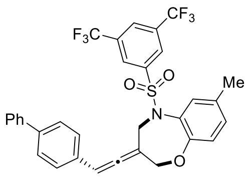

9g

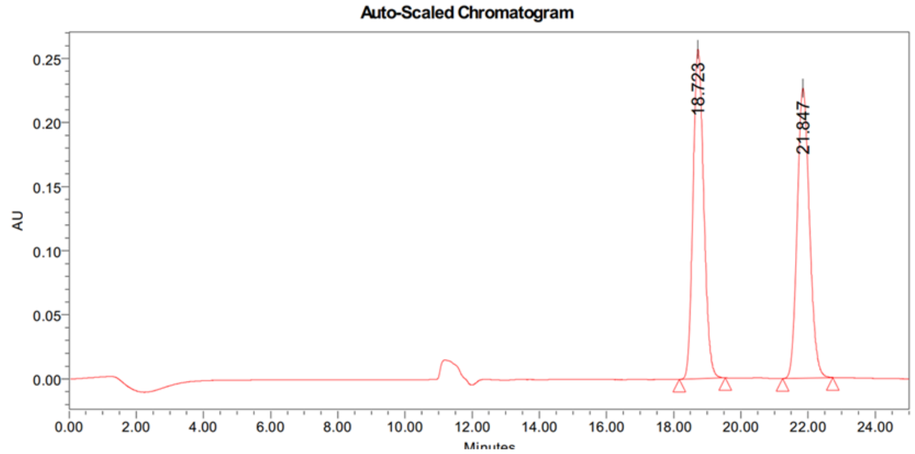

Peak Results

	Name	RT	Area	Height	% Area	Peak Width (sec)
1		18.723	5722919	256949	50.04	30.00
2		21.847	5714336	226318	49.96	30.00

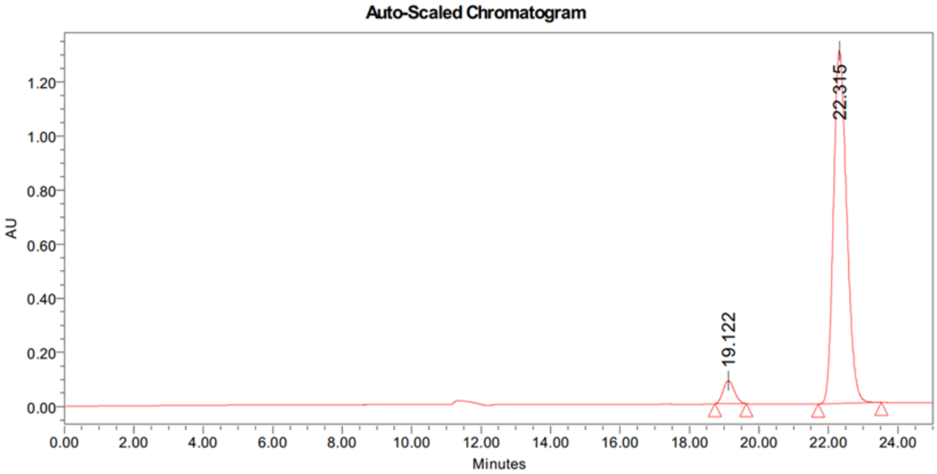

Peak Results

	Name	RT	Area	Height	% Area	Peak Width (sec)
1		19.122	1903027	84607	5.19	30.00
2		22.315	34742560	1303980	94.81	30.00

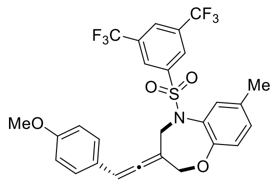

9h

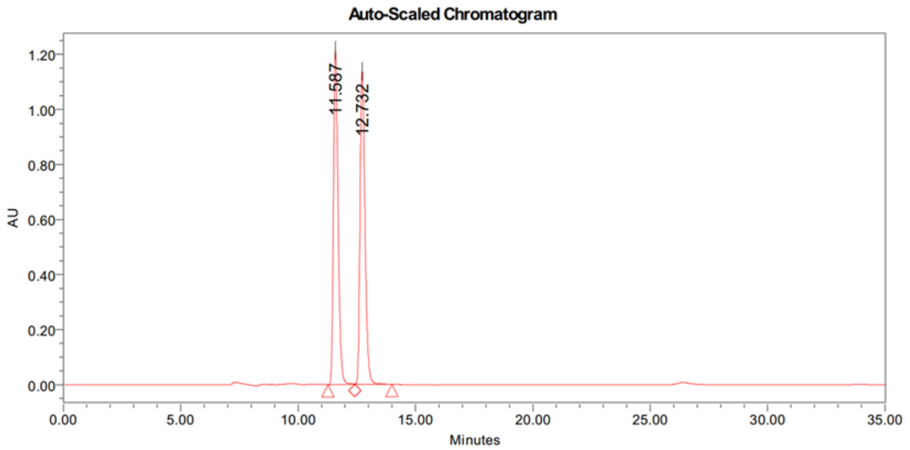

Peak Results

	Name	RT	Area	Height	% Area	Peak Width (sec)
1		11.587	16696297	1214710	49.86	30.00
2		12.732	16791455	1138425	50.14	30.00

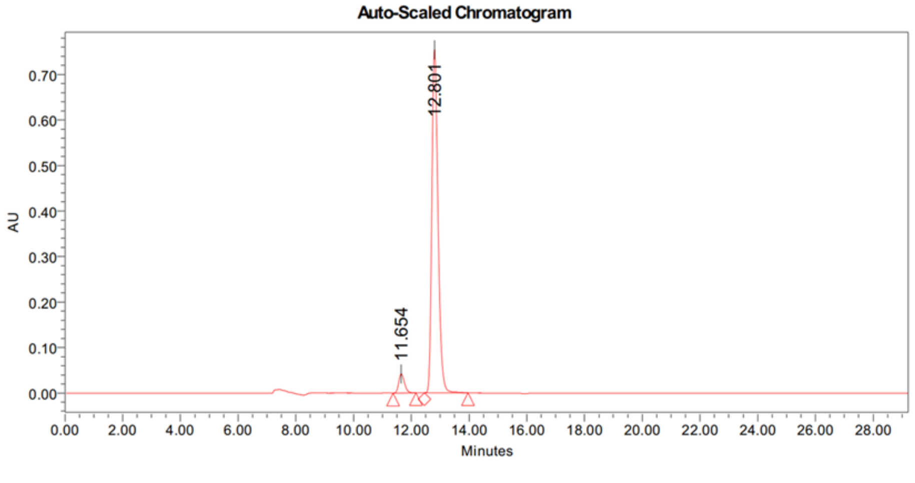

Peak Results

	Name	RT	Area	Height	% Area	Peak Width (sec)
1		11.654	577413	42080	4.93	30.00
2		12.801	11136586	754047	95.07	30.00

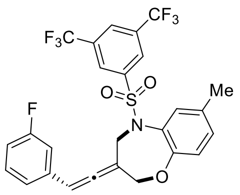

9i

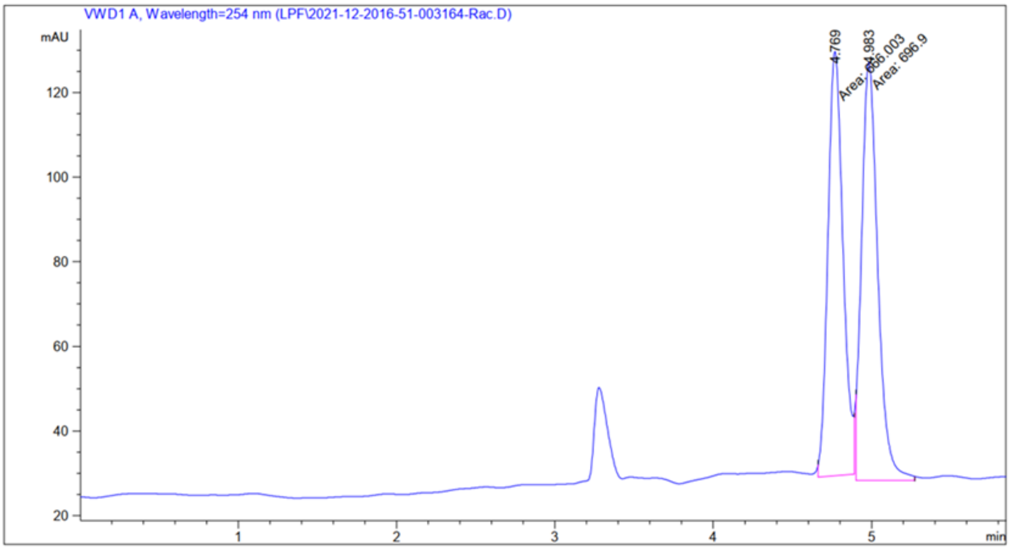

Peak #	RetTime [min]	Type	Width [min]	Area [mAU*s]	Height [mAU]	Area %
1	4.769	MM	0.1107	666.00250	100.28242	48.8665
2	4.983	MM	0.1174	696.90002	98.92054	51.1335

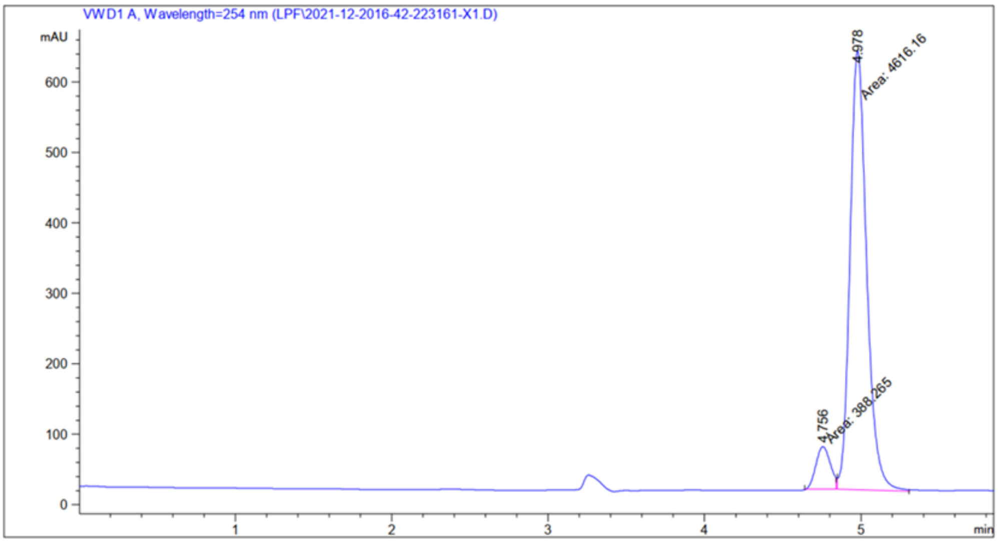

Peak #	RetTime [min]	Type	Width [min]	Area [mAU*s]	Height [mAU]	Area %
1	4.756	MM	0.1070	388.26517	60.49119	7.7584
2	4.978	MM	0.1235	4616.16260	622.82001	92.2416

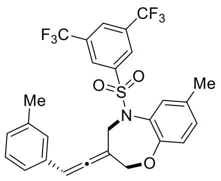

9j

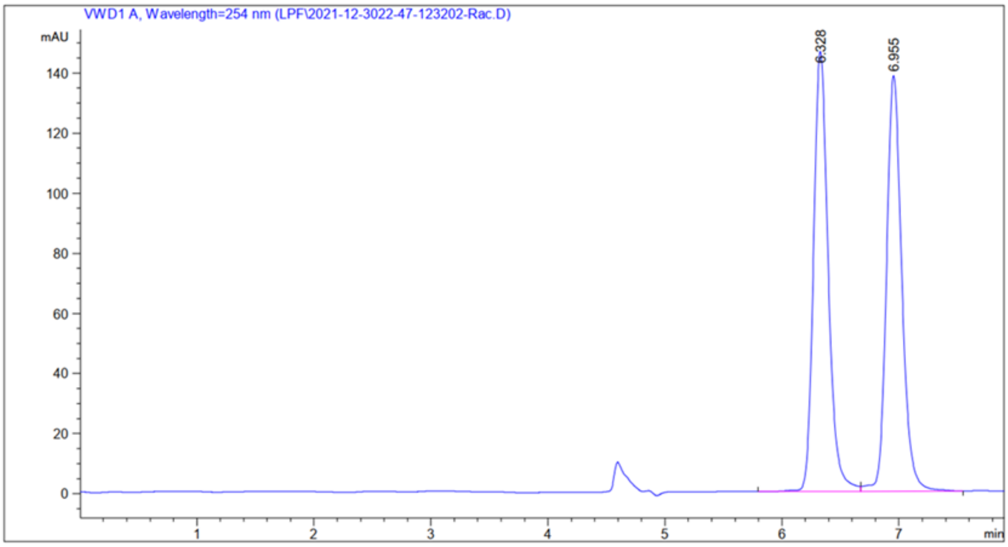

Peak #	RetTime [min]	Type	Width [min]	Area [mAU*s]	Height [mAU]	Area %
1	6.328	BV	0.1339	1278.91284	146.42386	50.3070
2	6.955	VB	0.1406	1263.30139	138.25479	49.6930

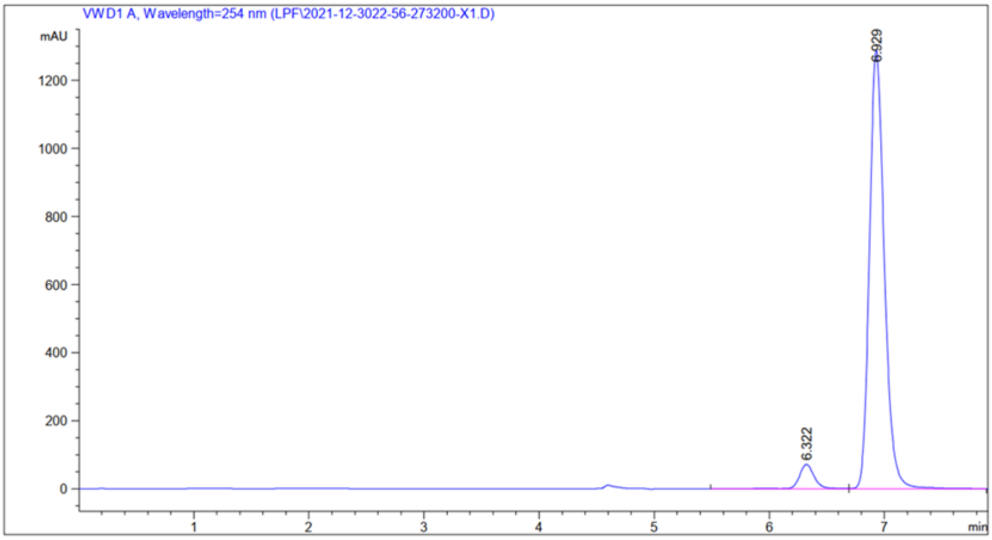

Peak #	RetTime [min]	Type	Width [min]	Area [mAU*s]	Height [mAU]	Area %
1	6.322	VV R	0.1367	640.42316	71.34164	5.2404
2	6.929	VB	0.1380	1.15805e4	1286.33838	94.7596

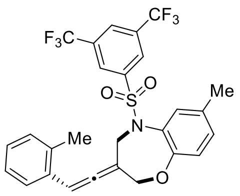

9k

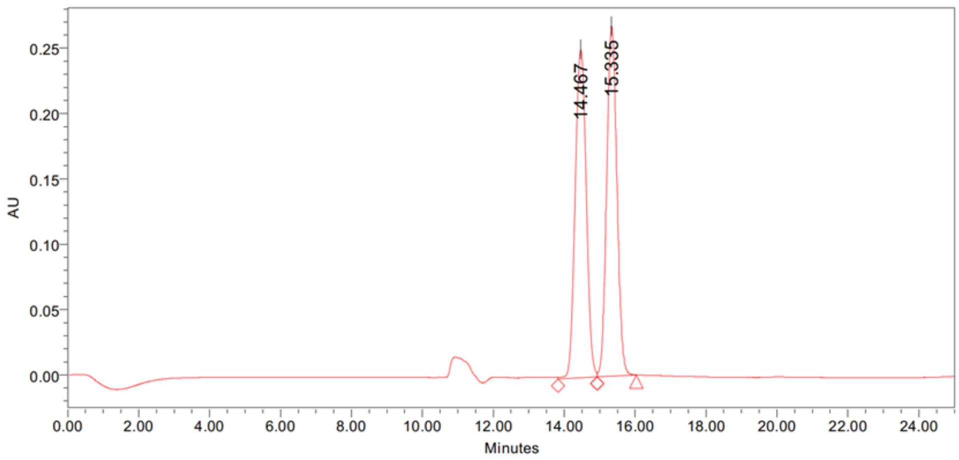

Peak Results

	Name	RT	Area	Height	% Area	Peak Width (sec)
1		14.467	5427895	251067	50.10	30.00
2		15.335	5405150	267660	49.90	30.00

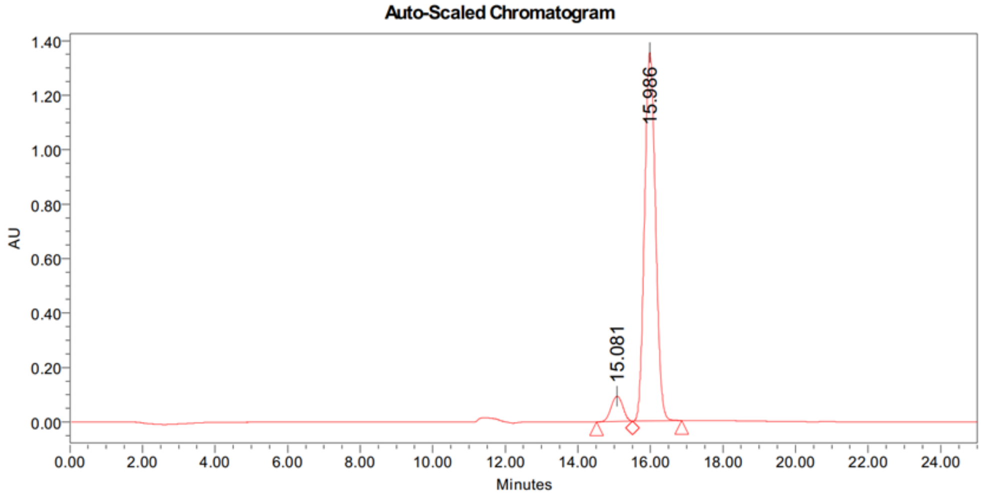

Peak Results

	Name	RT	Area	Height	% Area	Peak Width (sec)
1		15.081	2091211	92624	6.80	30.00
2		15.986	28672914	1353476	93.20	30.00

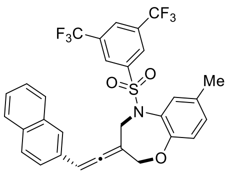

9l

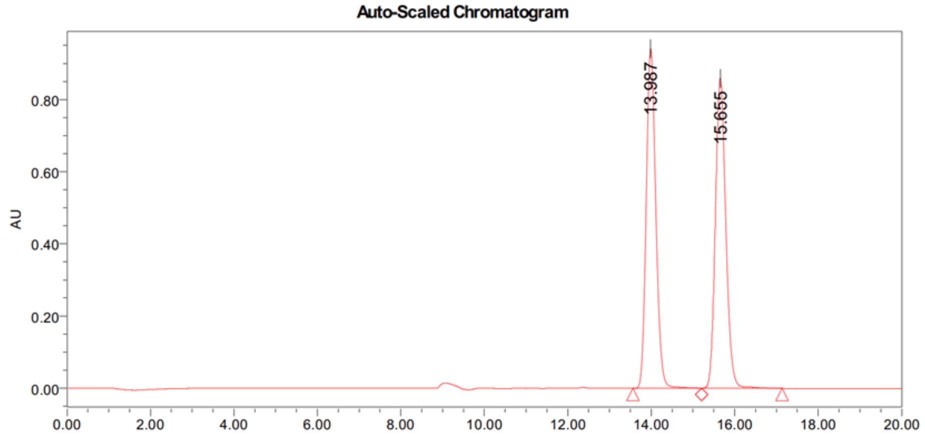

Peak Results

	Name	RT	Area	Height	% Area	Peak Width (sec)
1		13.987	14983032	941678	49.92	30.00
2		15.655	15030228	859736	50.08	30.00

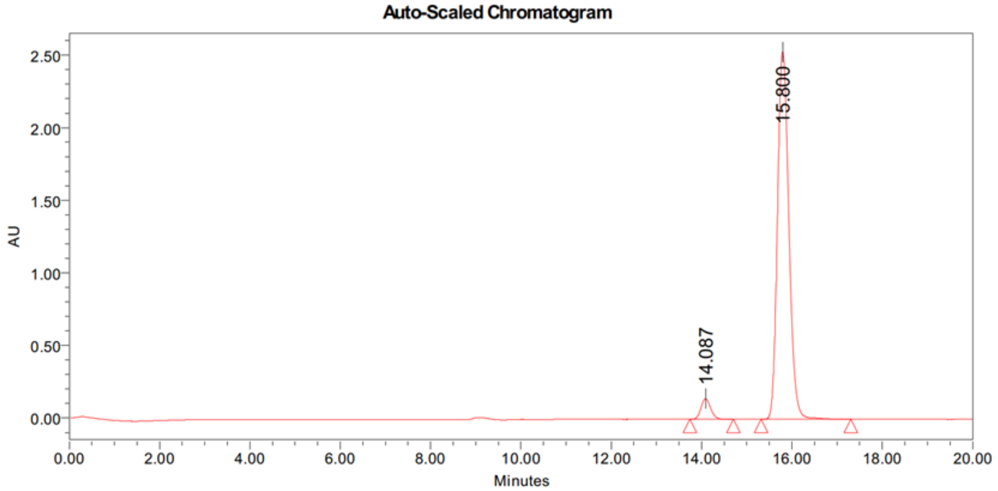

Peak Results

	Name	RT	Area	Height	% Area	Peak Width (sec)
1		14.087	2131628	143106	4.57	30.00
2		15.800	44464551	2531056	95.43	30.00

HPLC spectra of 13

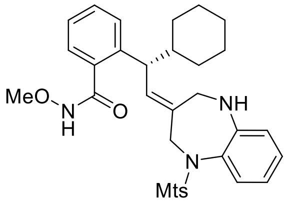

(*R*,*E*)-**13**

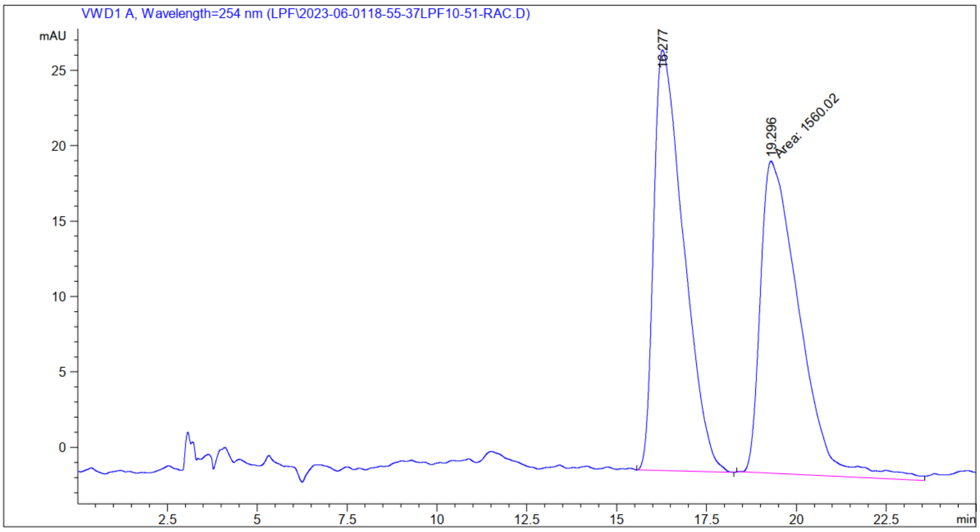

Peak #	RetTime [min]	Type	Width [min]	Area [mAU*s]	Height [mAU]	Area %
1	16.277	BB	0.8636	1612.15063	27.88209	50.8218
2	19.296	MM	1.2573	1560.01599	20.67946	49.1782

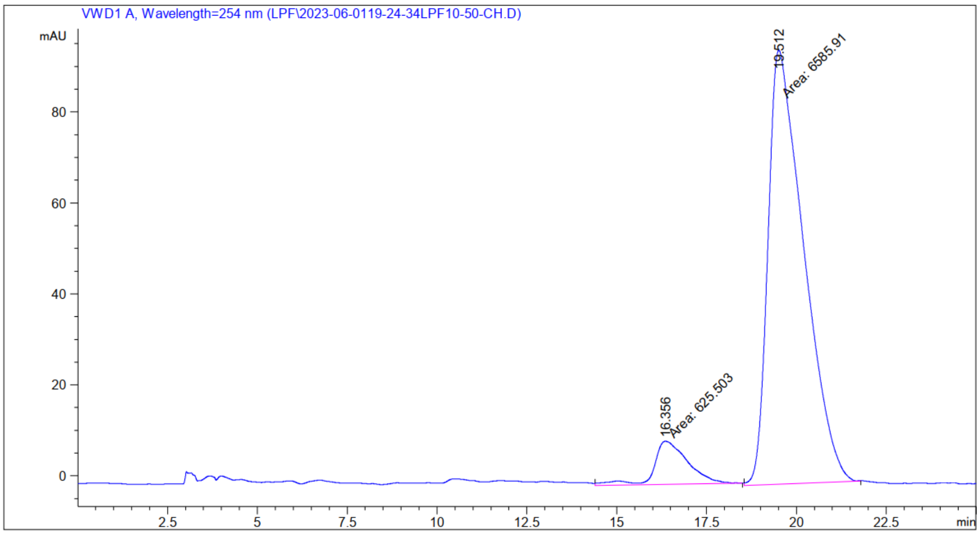

Peak #	RetTime [min]	Type	Width [min]	Area [mAU*s]	Height [mAU]	Area %
1	16.356	MM	1.0986	625.50269	9.48952	8.6738
2	19.512	MM	1.1508	6585.91455	95.38136	91.3262

HPLC spectra of 15

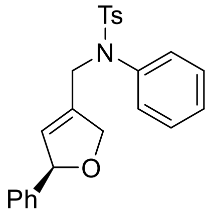

(*R*)-**15**

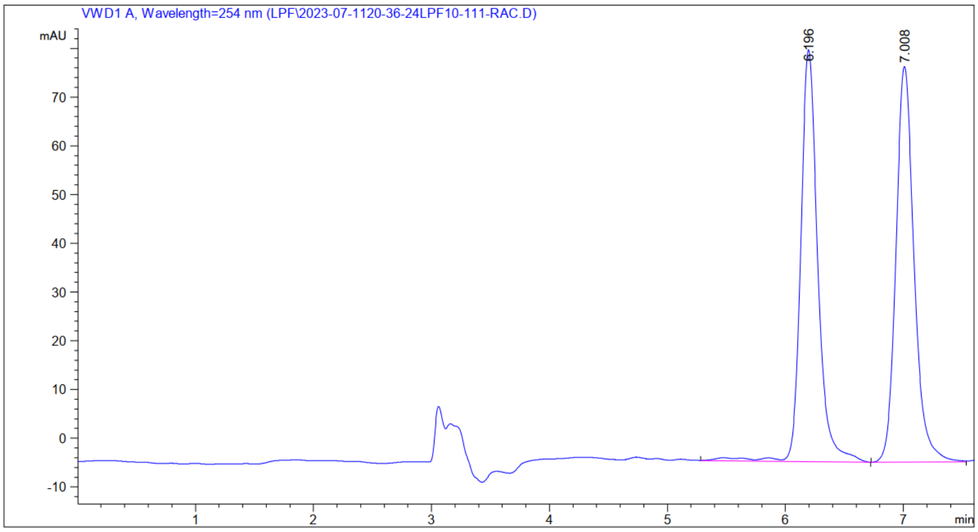

Peak #	RetTime [min]	Type	Width [min]	Area [mAU*s]	Height [mAU]	Area %
1	6.196	VB R	0.1529	853.60492	84.47184	49.5763
2	7.008	BV	0.1627	868.19446	81.20306	50.4237

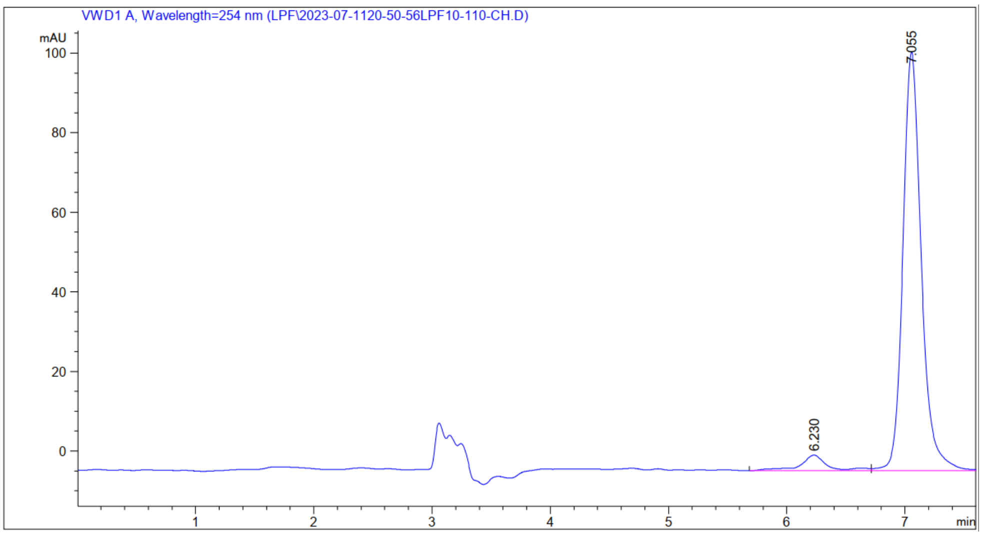

Peak #	RetTime [min]	Type	Width [min]	Area [mAU*s]	Height [mAU]	Area %
1	6.230	BV R	0.2232	62.24430	3.90099	5.1968
2	7.055	VV	0.1639	1135.50537	105.23139	94.8032
